# LncRNA TINCR favors tumorigenesis via STAT3–TINCR–EGFR-feedback loop by recruiting DNMT1 and acting as a competing endogenous RNA in human breast cancer

**DOI:** 10.1038/s41419-020-03188-0

**Published:** 2021-01-14

**Authors:** Qin Wang, Jiena Liu, Zilong You, Yanling Yin, Lei Liu, Yujuan Kang, Siwei Li, Shipeng Ning, Hui Li, Yajie Gong, Shouping Xu, Da Pang

**Affiliations:** 1 Department of Breast Surgery, Harbin Medical University Cancer Hospital, Harbin, China; 2 Heilongjiang Academy of Medical Sciences, Harbin, China

**Keywords:** Breast cancer, Long non-coding RNAs

## Abstract

The long noncoding RNA (lncRNA) TINCR has recently been found to be associated with the progression of human malignancies, but the molecular mechanism of TINCR action remains elusive, particularly in breast cancer. The oncogenic role of TINCR was examined in vitro and in vivo in breast cancer. Next, the interaction between TINCR, DNMT1, and miR-503-5p methylation was explored. Moreover, the mechanism by which TINCR enhances EGFR expression and downstream signaling via an RNA–RNA interaction was comprehensively investigated. Furthermore, upstream transcriptional regulation of TINCR expression by STAT3 was examined by performing chromatin immunoprecipitation. Finally, feedback signaling in the STAT3–TINCR–EGFR downstream cascade was also investigated. TINCR is upregulated in human breast cancer tissues, and TINCR knockdown suppresses tumorigenesis in vitro and in vivo. Mechanistically, TINCR recruits DNMT1 to the miR-503-5p locus promoter, which increases the methylation and suppresses the transcriptional expression of miR-503-5p. Furthermore, TINCR also functions as a competing endogenous RNA to upregulate EGFR expression by sponging miR-503-5p. In addition, TINCR stimulates JAK2–STAT3 signaling downstream from EGFR, and STAT3 reciprocally enhances the transcriptional expression of TINCR. Our findings broaden the current understanding of the diverse manners in which TINCR functions in cancer biology. The newly identified STAT3–TINCR–EGFR-feedback loop could serve as a potential therapeutic target for human cancer.

## Introduction

Long noncoding RNAs (lncRNAs) are >200-nt-long RNA transcripts encoded by the genome that are mostly not translated into proteins, but play key roles in regulating gene expression, chromatin dynamics, differentiation, growth, and development^1,2^. Several lncRNAs are also aberrantly expressed in cancers and can serve as diagnostic biomarkers and potential targets for cancer therapeutics^3–5^, and recent examination of increasing numbers of cancer transcriptomes by using next-generation sequencing has identified thousands of lncRNAs whose aberrant expression is associated with different cancer types, including breast, pancreatic, lung, liver, gastric, head and neck, and colon cancers^6–12^. Although lncRNAs have now been recognized as fundamental regulators of gene expression, most lncRNAs remain functionally uncharacterized in cancer.

Breast cancer (BC) is the most common female malignant tumor worldwide. A major feature is its challenging heterogeneity at the clinical and molecular level^13^. Over the past few decades, BC has revealed that it is quite complex and has no longer been considered a single disease, but rather a set of distinct subtypes^14^. BC subtypes have been classified according to immunohistochemical markers, clinicopathologic features, genomic alterations, and gene-expression profiling^15,16^. Aberrant expression of EGFR, a receptor tyrosine kinase of the ERBB family^17^, can result in unregulated growth stimulation and tumorigenesis in various types of cancer^18–22^. Moreover, inappropriate EGFR activation can occur through a range of complex mechanisms, including gene amplification, autocrine ligand–receptor stimulation, and epigenetic modulation^23–25^. However, to determine how intrinsic and acquired resistance to EGFR inhibitors in cancer treatments can be avoided, it is essential to comprehensively elucidate the regulatory landscape of the ERBB family and the interaction and crosstalk between lncRNAs and ERBB-family members.

Here, we uncovered the diverse manners in which the lncRNA TINCR functions in breast cancer biology. TINCR was found to be upregulated and positively correlated with EGFR expression in human breast cancer. Mechanistic analyses revealed that TINCR recruits DNMT1 to the miR-503-5p locus promoter and thereby increases miR-503-5p methylation and suppresses its transcriptional expression, and further that TINCR also functions as a competing endogenous RNA (ceRNA) to upregulate EGFR expression by sponging miR-503-5p. Moreover, TINCR stimulates JAK2–STAT3 signaling downstream from EGFR, and STAT3, in turn, increases the transcriptional expression of TINCR. Our findings enhance the current understanding of the diverse roles of TINCR in cancer biology, and the newly identified STAT3–TINCR–EGFR-feedback loop might represent a potential therapeutic target for human cancer.

## Results

### TINCR expression is upregulated and correlated with poor prognosis in breast cancer

**Fig. 1:**
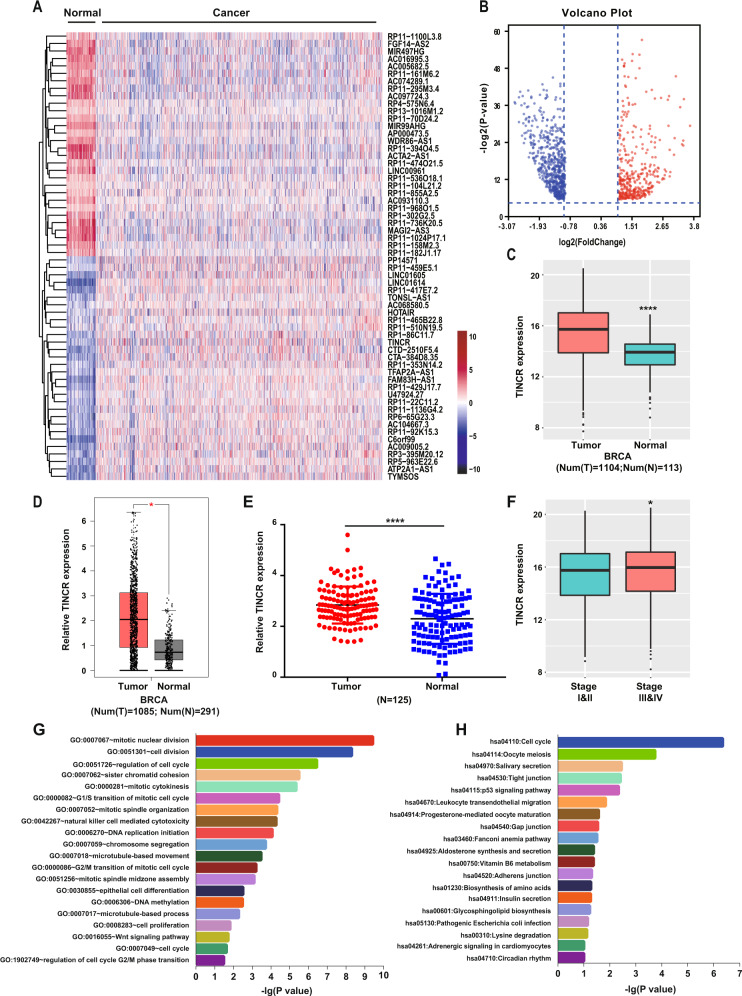
**TINCR is highly expressed in human breast cancer**. **a** Hierarchical-clustering heatmap of differentially expressed lncRNAs in breast-cancer and adjacent normal tissues, generated from RNA-seq data from TCGA. Colors: expression levels indicated by log_2_-transformed scale bar below the matrix; red and blue: max and min expression levels, respectively. **b** Volcano plot of lncRNAs differentially expressed between breast-cancer tissues normal tissues. *P* < 0.001, fold change >1.2 or fold change < –1.2. **c**–**e** TINCR expression in cancer and normal tissues from 1104 breast cancer patients in TCGA cohort (**c**), 1,085 breast-cancer patients in GEPIA cohort (**d**), and 125 breast-cancer patients in HMUCC cohort (**e**). **f** TINCR expression in breast cancers of different TNM stages in TCGA database. (**g**, **h**) GO and KEGG analyses of TINCR. *P* < 0.05 indicated statistical significance. Vertical axis: biological process or pathway category; horizontal axis: −log_10_ (*P* value) of significant biological process or pathway. *P < 0.05, **P < 0.01, ***P < 0.001, ****P < 0.0001.

By using the large-scale cancer-genome RNA-seq expression data from TCGA database, we examined the potential association of carcinogenesis with the lncRNAs differentially expressed between breast-cancer and adjacent normal tissues (Fig. 1a, b). Here, we focused on TINCR, which was found to be expressed at higher levels in human breast-cancer than in normal tissues (Fig. 1c, d). To validate the result, we analyzed 250 tissue specimens—125 breast-cancer and 125 adjacent normal tissues—from Harbin Medical University Cancer Center (HMUCC), and we found that TINCR was upregulated in breast-cancer tissues relative to the expression in normal tissues (Fig. 1e and Supplementary Table S1). Moreover, TINCR expression was higher in advanced tumor-node metastasis (TNM) stages III and IV than in TNM stages I and II (Fig. 1f). Next, GO and KEGG pathway analyses were performed to identify the potential functions of TINCR (Fig. 1g, h and Supplementary Table S2). The results indicated TINCR involvement in the biological processes of mitotic nuclear division, cell division, regulation of the cell cycle, DNA-replication initiation, DNA methylation, chromosome segregation, cell proliferation, and microtubule-based movement (Fig. 1g and Supplementary Table S2). Moreover, our results suggested potential TINCR participation in cell- cycle pathway, oocyte meiosis pathway, salivary-secretion pathway, p53 signaling pathway, progesterone-mediated oocyte-maturation pathway, aldosterone synthesis, and secretion pathway, and other pathways related to tumorigenesis (Fig. 1h and Supplementary Table S2). The association of TINCR overexpression with patient survival was analyzed in 125 patients with breast cancer in the HMUCC cohort. Patients harboring tumors with upregulated TINCR expression were associated with poor overall survival (OS) (Supplementary Fig. S1).

**Fig. 2:**
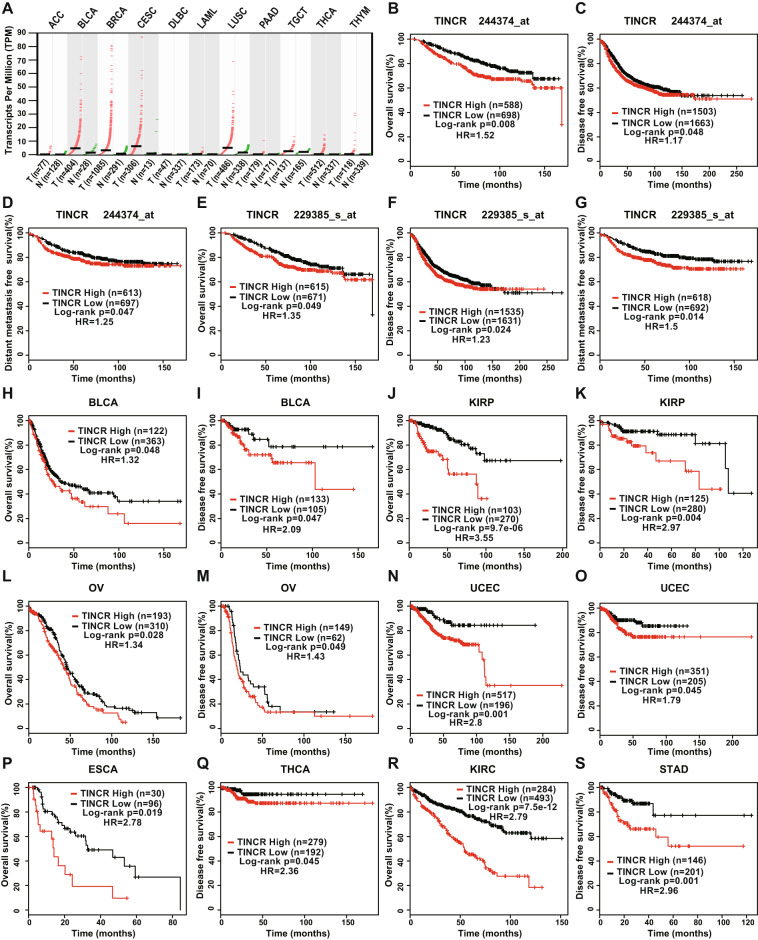
**High expression of TINCR is correlated with poor prognosis in human cancer**. **a** Scatter diagram depicting TINCR expression in multiple cancer and adjacent normal tissues, generated from GEPIA database. **b**–**g** Kaplan–Meier analysis of OS (**b**, **e**), DFS (**c**, **f**), and DMFS (**d**, **g**) of breast- cancer patients according to TINCR expression, generated from GEO database. **h**–**s** Kaplan–Meier analysis of OS and DFS in BLCA (**h**, **i**), KIRP (**j**, **k**), OV (**l**, **m**), UCEC (**n**, **o**), ESCA (**p**), THCA (**q**), KIRC (**r**), and STAD (**s**) patients according to TINCR expression, generated from TCGA database. Data are shown as means ± standard deviation (SD); Student’s *t* test, *P < 0.05, **P < 0.01, ***P < 0.001, ****P < 0.0001.

Besides breast cancer, multiple other cancers exhibited TINCR upregulation, including adrenocortical carcinoma (ACC), bladder urothelial carcinoma (BLCA), cervical and endocervical cancers (CESC), lymphoid neoplasm-diffuse large B-cell lymphoma (DLBC), acute myeloid leukemia (LAML), lung squamous-cell carcinoma (LUSC), pancreatic adenocarcinoma (PAAD), testicular germ-cell tumors (TGCT), thyroid carcinoma (THCA), and thymoma (THYM), according to an analysis of the GEPIA database (Fig. 2a). To investigate the relationship between TINCR expression and the prognosis of cancer patients, Kaplan–Meier survival analysis and log-rank test were performed to assess the effects of TINCR expression and clinical outcomes on the survival of patients with breast cancer in the GEO database (two TINCR probes were used here, 244374-at/229385-at) (Supplementary Table S3) and other cancers in TCGA database. High TINCR expression indicated markedly poorer prognosis than low TINCR expression did in BRCA patients (Fig. 2b–g) and BLCA, kidney renal papillary-cell carcinoma (KIRP), ovarian serous cystadenocarcinoma (OV), uterine corpus endometrial carcinoma (UCEC), esophageal carcinoma (ESCA), THCA, kidney renal clear-cell carcinoma (KIRC), and stomach adenocarcinoma (STAD) patients (Fig. 2h–s). EGFR showed higher expression in the 4T1, MDA-MB-231, and UACC-812 cell lines than the other tested cell lines (Supplementary Fig. S2). Thus, the following experiments were performed with these three cell lines to examine the regulatory effects on EGFR expression.

### TINCR promotes tumor growth in vivo and in vitro

**Fig. 3:**
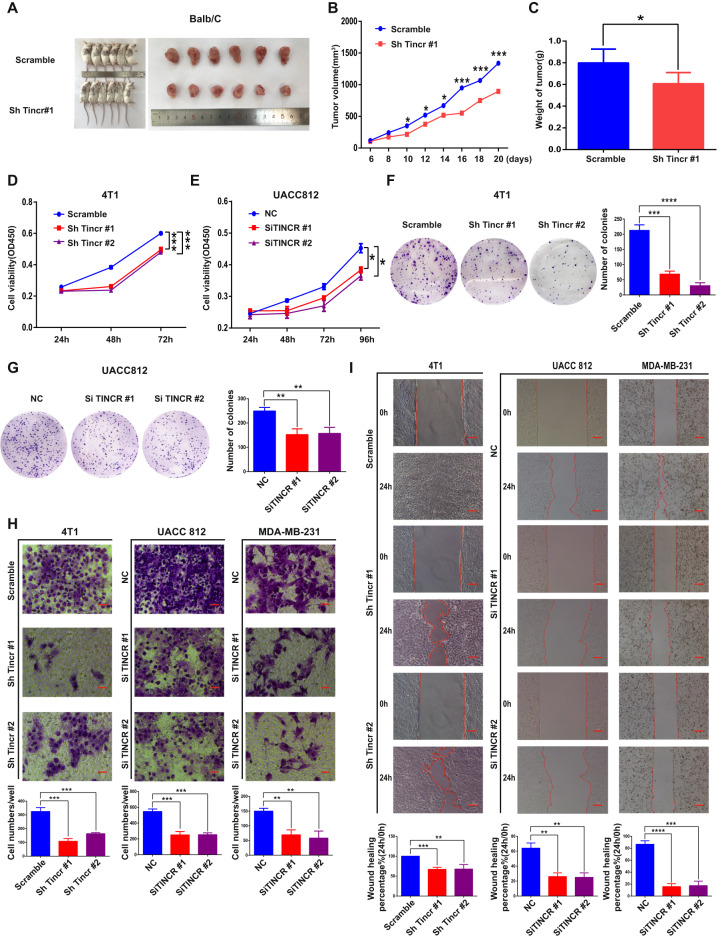
**TINCR promotes tumor growth in vivo and in vitro**. **a** Tumorigenesis and tumor tissue of each group of Balb/c mice (*n* = 6/group). **b**, **c** Tumor volume and weight measured in Balb/c mice after injection of 4T1 cells transfected with Tincr-specific or scrambled-control shRNA. Tumor tissues were measured at the indicated time points and dissected at the endpoint. **d**–**i** Knockdown of Tincr/TINCR expression inhibits proliferation (**d**, **e**), colony formation (**f**, **g**), and migration (**h**, **i**) of 4T1, UACC 812, and MDA-MB-231 cells. Scale bar, 100 μm. Data are presented as means from three independent experiments ± S.D. *P < 0.05, **P < 0.01, ***P < 0.001, ****P < 0.0001.

We next investigated the potential biological functions of TINCR: we used shRNAs specifically designed to target the mouse homolog of the gene, Tincr, and injected Balb/c mice with 4T1 cells in vivo. Tumor growth was markedly inhibited in mice injected with TINCR-knockdown cells as compared with the growth in mice injected with control cells (Fig. 3a, b), and the median tumor weight was also lower in the knockdown than in the control group (Fig. 3c). Furthermore, the results of in vitro assays showed that proliferation and colony formation were lower in TINCR-knockdown cells relative to control (Fig. 3d–g), and TINCR knockdown also decreased the invasiveness of these cells (Fig. 3h, i).

### TINCR upregulates EGFR expression and downstream signaling in human breast cancer

**Fig. 4:**
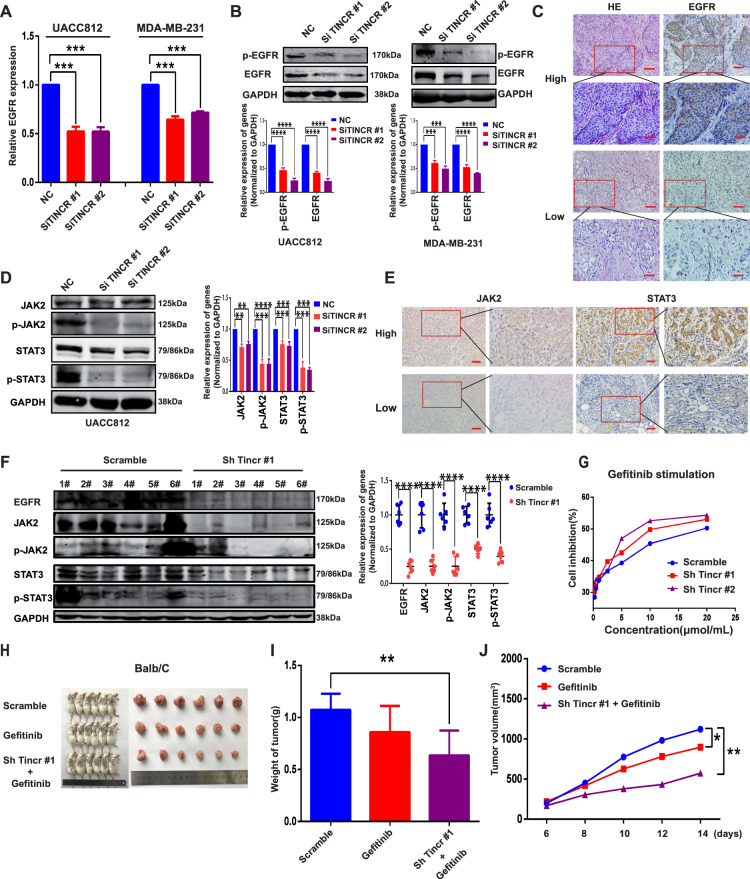
**TINCR regulates EGFR expression and downstream signaling in human cancer**. **a**, **b** TINCR knockdown decreases expression of EGFR mRNA (**a**) and protein (**b**) in UACC 812 and MBA-MB-231 cells. **c** EGFR protein expression is higher in TINCR^-high^ breast-cancer tissues than in TINCR^-low^ tissues and vice versa in 122 patients in the HMUCC cohort. Scale bar, 400 μm. **d** TINCR knockdown decreases the levels of p-JAK2, STAT3, and p-STAT3 in UACC 812 cells. **e** JAK2 and STAT3 protein expression is higher in TINCR^-high^ breast-cancer tissues than in TINCR^-low^ tissues and vice versa in 125 patients in the HMUCC cohort. Scale bar, 400 μm. **f** TINCR knockdown decreases the levels of EGFR, JAK2, p-JAK2, STAT3, and p-STAT3 in vivo (*n* = 6/group). **g** Gefitinib IC_50_ analysis after Tincr knockdown. **h** Tumorigenesis and tumor tissue of each group of Balb/c mice (*n* = 6/group). **i**, **j** Tumor growth and weight measured in Balb/c mice in each group. Tumors were measured at the indicated time points and dissected at the endpoint. Data are presented as means from three independent experiments ± S.D. *P < 0.05, **P < 0.01, ***P < 0.001, ****P < 0.0001.

The aforementioned guilt-by-association analysis and our previous study^26^ suggested that TINCR could perform its potential oncogenic function through EGFR and the EGFR downstream signaling pathway. Here, TINCR knockdown decreased EGFR mRNA and protein expression relative to control in UACC-812 and MDA-MB-231 cells (Fig. 4a, b). We also examined the relationship between TINCR and EGFR protein expression in 120 breast-cancer samples from HMUCC, and the results showed that the EGFR level was higher in TINCR high-expression tissues than in TINCR low-expression tissues (Fig. 4c, Supplementary Fig.S3, and Supplementary Table S4). Next, we tested how TINCR knockdown affects EGFR downstream signaling. As per our expectation, TINCR knockdown led to a decrease in p-JAK2, STAT3, and p-STAT3 relative to their levels in control cells (Fig. 4d). To gain insights into the role of TINCR in EGFR downstream signaling in a clinical context, we performed immunohistochemical analysis on samples from the HMUCC cohort, which revealed that JAK2 and STAT3 were more abundant in TINCR high-expression tissues than TINCR low-expression tissues (Fig. 4e, Supplementary Fig. S4, and Supplementary Table S4). Furthermore, in Balb/c mice injected with 4T1 cells in vivo, EGFR and its downstream-signaling molecules were downregulated in the TINCR-knockdown group relative to the control group (Fig. 4f). To further validate the result, we analyzed tissue specimens from HMUCC, and we found that EGFR, JAK2, and STAT3 were upregulated in breast-cancer tissues relative to the expression in normal tissues (Supplementary Fig. S5).

Considering the aforementioned results, we sought to ascertain whether TINCR knockdown and EGFR-inhibitor treatment used together would produce a synergistic anticancer effect. When TINCR knockdown was combined with treatment with the EGFR inhibitor gefitinib, cell-inhibition activity was higher than in the gefitinib group (IC50 = 13.29 and 10.02 μmol/L vs. 26.3 μmol/L) (Fig. 4g). Furthermore, this combination treatment more effectively inhibited tumor growth than the single treatments in vivo (Fig. 4h, i), and the median tumor weight was lower in the combination- treatment group than in the gefitinib or control group (Fig. 4j).

### TINCR upregulates EGFR expression by acting as a ceRNA to sponge miR-503-5p

**Fig. 5:**
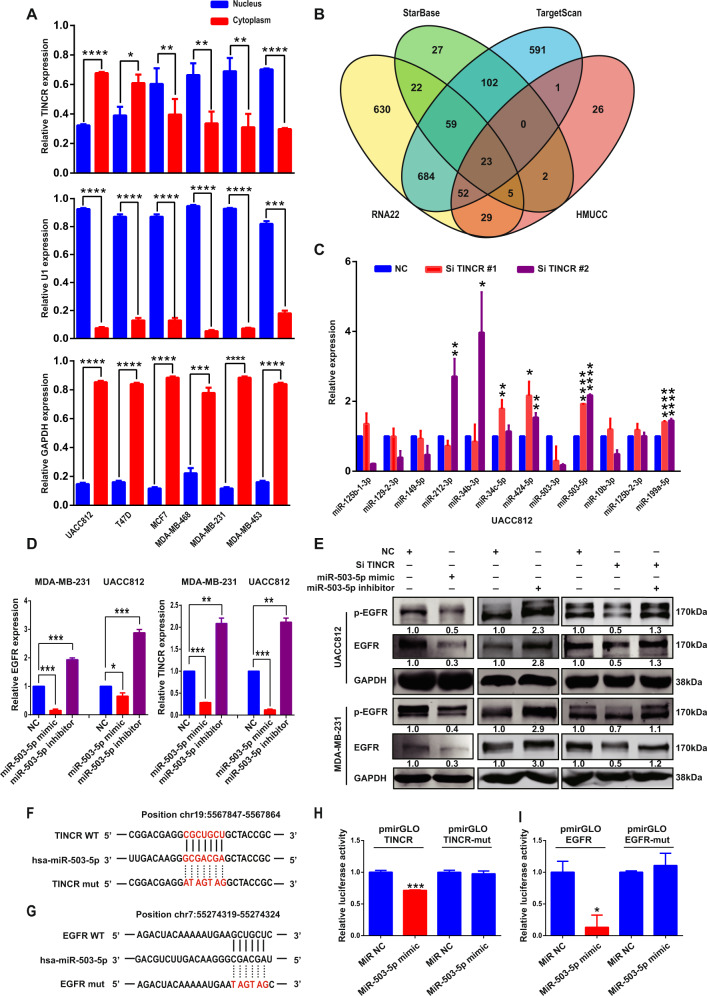
**TINCR promotes EGFR expression by acting as a ceRNA**. **a** Subcellular localization of TINCR in breast-cancer cell lines, assessed using nuclear/cytoplasmic extract isolation assay. **b** Overlapping miRNAs in transcriptome miRNA sequencing data from HMUCC cohort and TargetScan, RNA22, and starBase databases. Venn diagrams were generated using Venny online software (http://bioinfogp.cnb.csic.es/tools/ venny/). **c** qRT-PCR examination of the expression of miRNAs in UACC 812 cells transfected with a TINCR-specific siRNA or scrambled siRNA. **d**, **e** Expression of TINCR, EGFR mRNA (**d**), and EGFR protein (**e**) in UACC 812 and MDA-MB-231 cells treated with miR-503-5p mimic or inhibitor. **f**, **g** Complementarity between miR-503-5p seed sequence and 3ʹ-UTRs of TINCR (**f**) and EGFR (**g**) predicted through a computational and bioinformatics-based approach by using TargetScan and StarBase online databases. Watson–Crick complementarity is connected by “|.” Nucleotide-replacement mutations made to the genes are underlined. **h**, **i** Luciferase-reporter assay for assessing interactions between miR-503-5p and its binding sites or mutated binding sites in 3ʹ-UTRs of TINCR (**h**) and EGFR (**i**) in HEK293T cells. Data are presented as means from three independent experiments ± S.D. *P < 0.05, **P < 0.01, ***P < 0.001, ****P < 0.0001.

Next, we investigated the potential mechanism by which TINCR regulates EGFR expression. First, the subcellular localization of TINCR was examined, which revealed TINCR localization in both the cytoplasm and the nucleus in breast-cancer cells (Fig. 5a and Supplementary Fig. S6). LncRNAs localized in the cytoplasm typically act as ceRNAs to influence the expression of their target genes^27^. To identify miRNA candidates that might be affected in TINCR-knockdown and scrambled-control groups, transcriptome miRNA sequencing was performed to identify microRNAs that potentially bind TINCR transcript on the HMUCC cohort samples. We identified 138 potential microRNA candidates in TINCR-knockdown compared to the control groups (Supplementary Table S5). Furthermore, the public databases TargetScan, RNA22, and StarBase were used to predict potential miRNAs that bind to EGFR 3ʹ-UTR (Supplementary Table S5), and overlapping miRNAs were examined among the transcriptome-sequencing data from the HMUCC cohort and the TargetScan, RNA22, and StarBase databases (Fig. 5b and Supplementary Table S5). Ultimately, miR-503-5p was selected for the following experiments. The knockdown of TINCR increased the expression of miR-503-5p (Fig. 5c). Moreover, the levels of TINCR, EGFR mRNA, and EGFR protein were decreased and increased, respectively, after transfection of miR-503-5p mimic and its corresponding inhibitor (Fig. 5d, e). Notably, combining TINCR knockdown with miR-503-5p-inhibitor treatment attenuated the decrease in EGFR protein levels (Fig. 5e). Last, the results of luciferase-reporter assays to investigate physical interactions showed that both EGFR and TINCR with their wild-type 3ʹ-UTRs were regulated by miR-503-5p, and that this effect could be abolished by mutating their miRNA-binding sites (Fig. 5f–i). These results indicate that EGFR and TINCR are bona fide direct targets of miR-503-5p.

### TINCR recruits DNMT1 to miR-503-5p locus and suppresses its expression via DNA methylation

**Fig. 6:**
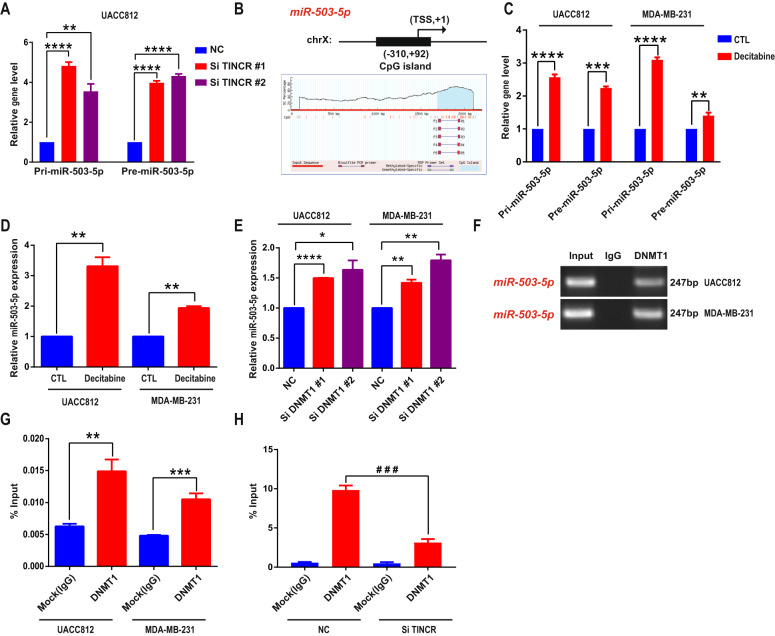
**TINCR recruits DNMT1 to miR-503-5p promoter to regulate miR-503-5p gene methylation**. **a** TINCR knockdown increases Pri-MiR-503-5p and Pre-MiR-503-5p expression in UACC 812 cells. **b** Schematic representation of predicted CpG island in miR-503-5p locus. CpG island located in the promoter region of miR-503-5p locus around the transcriptional start site (TSS, −310 to +92 bases; “−”: upstream of TSS; “+”: downstream of TSS). **c** Pri-MiR-503-5p and Pre-MiR-503-5p expression is upregulated in UACC 812 and MDA-MB-231 cells treated with decitabine (1 μM) for 10 h. **d** MiR-503-5p expression is upregulated in UACC-812 and MDA-MB-231 cells treated with decitabine (1 μM) for 10 h. **e** miR-503-5p expression is upregulated in UACC 812 and MDA-MB-231 cells following DNMT1 knockdown. **f** ChIP assay results indicating that DNMT1 is enriched at miR-503-5p locus CpG islands in UACC 812 and MDA-MB-231 cells. **g** RIP-assay results showing higher enrichment of TINCR in DNMT1 group than IgG group in UACC 812 and MDA-MB-231 cells. IgG: negative control. **h** ChIP assay results indicating diminished enrichment of DNMT1 at the promoter of miR-503-5p locus after TINCR knockdown. Data are presented as means from three independent experiments ± S.D. *P < 0.05, **P < 0.01, ***P < 0.001, ****P < 0.0001, ^#^^#^^#^*P* < 0.001.

As mentioned in the preceding section, TINCR was also localized in the nucleus (Fig. 5a and Supplementary Fig. S6), which suggested that TINCR could exert its regulatory effect through epigenetic modification of target genes, and knockdown of TINCR expression was found to upregulate miR-503-5p (Fig. 5c). First, TINCR knockdown increased pri-mir-503-5p and pre-mir-503-5p level relative to control in UACC 812 cells (Fig. 6a). Because DNA methylation is a major epigenetic-modification mechanism that results in the downregulation of gene expression^11^, we tested whether methylation of the miR-503 locus was regulated through TINCR. Next, we obtained information on the region around the transcriptional start site (TSS, −2000 to +200 bases; “−”: upstream of TSS; “+”: downstream of TSS) by using the University of California at Santa Cruz (UCSC) database (http://genome.ucsc.edu/cgi-bin/hgGateway), and we then located a CpG island in the promoter region of miR-503 locus from the public data of the Li Lab (http://www.urogene.org/methprimer/index.html) (Fig. 6b). Bisulfite sequencing was performed to investigate the role of TINCR in CpG-island methylation in the miR-503 locus. As expected, TINCR knockdown led to a decrease in miR-503-5p CpG-island methylation at positions ChrX.133680280 and ChrX.133680391 (Supplementary Table S6), and pri-miR-503-5p, pre-miR-503-5p, and miR-503-5p expression was increased following treatment with the DNA methyltransferase inhibitor decitabine (1 μM) (Fig. 6c, d). Because DNMT1 is a maintenance methyltransferase that preserves the methylation state across mitotic divisions^28^, we tested whether miR-503-5p expression is regulated by DNMT1. Accordingly, miR-503-5p was upregulated when DNMT1 expression was knocked down (Fig. 6e), and the results of ChIP-PCR assays indicated DNMT1 enrichment at the miR-503-5p promoter (Fig. 6f). Notably, RIP-assay results indicated markedly higher TINCR enrichment in the DNMT1 group than the IgG group (Fig. 6g), and DNMT1 enrichment at the miR-503-5p promoter was decreased relative to control after knockdown of TINCR (Fig. 6h).

### Identification of positive-feedback loop in STAT3–TINCR–EGFR axis

**Fig. 7:**
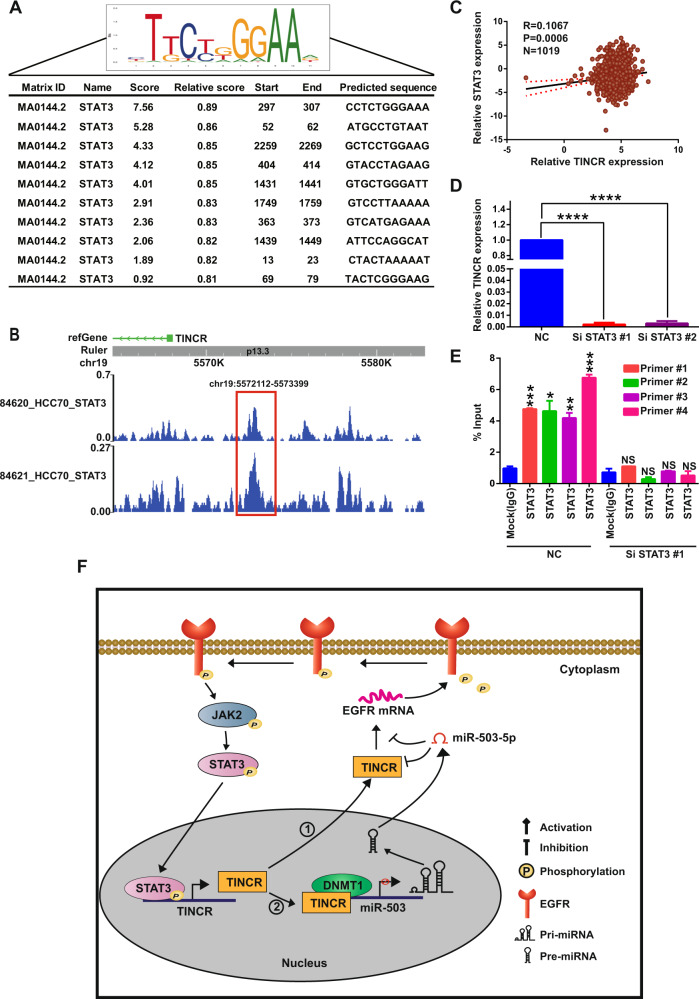
**Identification of the positive-feedback loop in STAT3–TINCR–EGFR signaling axis**. **a** Upper corner of picture: STAT3-binding motif; lower table: prediction of STAT3-binding sites within TINCR promoter region, from JASPAR database. **b** Enrichment peak of STAT3 in the promoter of TINCR in ChIP-seq of HCC70 cells in ENCODE database. **c** TINCR expression is positively correlated with STAT3 mRNA expression in 1019 cancer cell lines in CCLE cohort. **d** STAT3 knockdown decreases TINCR expression in MBA-MB-231 cells. **e** ChIP assay results indicating STAT3 enrichment at TINCR promoter and diminished STAT3 enrichment at the promoter after STAT3 knockdown. **f** Molecular mechanism of dual regulation by TINCR of EGFR and its downstream genes. Data are presented as means from three independent experiments ± S.D. *P < 0.05, **P < 0.01, ***P < 0.001, ****P < 0.0001.

To investigate the transcriptional regulation upstream of TINCR, we used the online database JASPAR (http://jaspar.genereg.net/) to search for transcription factors that might potentially be enriched at the promoter of the TINCR locus. The analysis yielded putative STAT3-binding sites in the region upstream of the TSS of TINCR (Fig. 7a). Moreover, there were obvious enrichment peaks of STAT3 in the promotor region (chr19-5572112–5573399) of TINCR in HCC70 cells in ENCODE database (Fig. 7b). Next, from the CCLE database, TINCR expression was found to be positively correlated with STAT3 expression in human cancer cells (Fig. 7c). Last, STAT3 knockdown decreased the expression of TINCR (Fig. 7d), and the results of ChIP-PCR assays indicated that STAT3 was highly enriched at the TINCR promoter relative to mock control (Fig. 7e). Collectively, our results indicate that TINCR stimulates the activation of EGFR and its key downstream-signaling effectors, including STAT3, which, reciprocally, promotes the transcriptional expression of TINCR.

## Discussion

LncRNAs have been found to be associated with tumorigenesis and drug resistance in various types of cancer^29–33^. Identification of novel onco-lncRNAs and their regulatory mechanisms, and development of novel lncRNA-based targeted strategies for combating cancer, appears promising and may introduce a new paradigm in cancer research. Moreover, EGFR activity has been reported to be modulated by diverse signaling pathways^34–40^. However, the comprehensive regulation of EGFR by lncRNAs remains poorly understood. Our study revealed that TINCR upregulated EGFR expression through a ceRNA interaction to trigger enhanced JAK2–STAT3 downstream signaling, and that STAT3, in turn, increased TINCR transcriptional expression. Our study uncovers a distinctive facet of tumorigenesis by identifying a previously unknown positive-feedback loop in the STAT3–TINCR–EGFR signaling pathway; this suggests that the feedback loop could represent a new therapeutic target in pharmacological strategies developed for human cancers. In Fig. 7f, we present a schematic model of the molecular mechanism underlying the dual regulation by TINCR of EGFR and its downstream genes through miR-503-5p.

Generally, lncRNAs interact with chromatin DNA, RNA, or protein, thereby regulating chromatin accessibility, RNA stability, and protein activity or stability in *cis*- or *trans*-manner^41,42^. Unlike the classical theory, the identification of TINCR ceRNA and RNA–protein-mediated dual regulation in this study reflects the lncRNA-mediated complex regulation of its target genes. In terms of their regulatory mechanism, lncRNAs localized in the cytoplasm have been widely reported to potentially compete for miRNA-response elements (MREs) with target genes closely related to cancer occurrence and development by acting as ceRNAs; this would weaken the inhibitory effect of miRNAs on target genes and indirectly regulate the expression levels of the target genes^43–46^. Our study suggests that TINCR functions as a ceRNA to upregulate EGFR expression by acting as a sponge for miR-503-5p. Conversely, lncRNAs localized in the nucleus can regulate gene expression by acting as epigenetic modulators^47,48^. However, no previous study has reported lncRNA regulation of miRNAs through the modification of methylation. We have shown here for the first time that TINCR can recruit DNMT1 to the miR-503-5p promoter to regulate miR-503-5p locus methylation and thus transcription. Thus, this study fills in the theoretical gap in the lncRNA- regulation model and provides a comprehensive understanding regarding the above-mentioned dual-regulatory network in human breast cancer.

In conclusion, this study has revealed that TINCR promotes tumorigenesis through a STAT3–TINCR–EGFR-feedback loop by recruiting DNMT1 and acting as a ceRNA in human breast cancer. We have demonstrated for the first time that TINCR upregulates EGFR expression by means of a dual molecular mechanism through the aforementioned RNA–protein and RNA–RNA interactions to suppress miR-503-5p expression. These findings broaden the ceRNA landscape of TINCR and enhance our understanding of the complex regulatory network through which lncRNAs influence their target genes. Moreover, the study has uncovered a previously unrecognized positive-feedback loop in the STAT3–TINCR–EGFR signaling axis in tumorigenesis, and this feedback loop could thus represent a new target in pharmacological strategies for treating human breast cancer.

## Materials and methods

### Public data access and analysis

Genome-wide expression profiles of TINCR and clinical pathology information were downloaded from the databases of The Cancer Genome Atlas (TCGA) (https://tcga-data.nci.nih.gov/), Gene Expression Profiling Interactive Analysis (GEPIA) (http://gepia.cancer-pku.cn/), Cancer Cell Line Encyclopedia (CCLE) (https://portals.broadinstitute.org/ccle/about), and Gene Expression Omnibus (GEO) (https://www.ncbi.nlm.nih.gov/geo/). All transcripts were normalized by the log_2_ method. Wilcoxon rank-sum statistical analysis was used to detect significant differences among tumors or between tumor and normal samples. The correlations between genes were assessed by Spearman’s correlation coefficients. To calculate Spearman’s correlation coefficient, we rank and compare data sets to find Σd^2^, then plug the value into the standard version of Spearman’s rank- correlation coefficient formula according to previous study^49^ Guilt-by-association analysis was performed to identify coding genes that were positively or negatively correlated with TINCR expression. JASPAR (http://jaspar.genereg.net/) was used to examine the transcription factors that might potentially be enriched at the TINCR locus promoter. Correlations between TINCR and EGFR/JAK2 mRNA levels were assessed using Pearson correlation coefficients. Unpaired Student’s *t* tests were used to detect significant differences among tumors or between tumor and normal samples. Survival was calculated by the Kaplan–Meier method, with the log-rank test applied for comparison. Overall survival (OS) was calculated as the time from surgery until death; disease-free survival (DFS) was measured as the time for which patients were disease-free, and distant metastasis-free survival (DMFS) was calculated as the survival time specifically free of distant metastasis. The log-rank test was used to examine the survival difference between distinct patient groups. All statistical tests were two-sided, and *P* < 0.05 was considered statistically significant. Gene ontology (GO)-term enrichment and Kyoto Encyclopedia of Genes and Genomes (KEGG) pathway analyses of these genes were performed using DAVID as previously described^50,51^

### Breast-tissue specimens and clinical assessments

Eligible patients with a histological diagnosis of breast cancer who had received neither chemotherapy nor radiotherapy before surgical resection were recruited to this study. Informed consent was obtained from all patients. In total, 125 breast-cancer tissues and 125 normal tissues were obtained from Harbin Medical University Cancer Center (HMUCC). For RNA extraction, fresh tissue was collected from patients with breast cancer and normal controls and stored at −80 °C immediately after resection. Two independent senior pathologists confirmed the pathological diagnosis and molecular subtype of each cancer tissue. This study conformed to the clinical research guidelines and was approved by the research ethics committee of Harbin Medical University Cancer Hospital. We obtained written informed consent from all patients.

### Cell culture, plasmid construction, and transfection

Breast-cancer cell lines (UACC-812, MDA-MB-231, and 4T1) were obtained from the Chinese Academy of Sciences Cell Bank and Cellbio (China) and cultured and stored according to their instructions. Mycoplasma testing was performed before the experiments. For transfection, cells were seeded in six-well dishes the night before to obtain 60–70% confluence for plasmid transfection and 80% confluence for microRNA (miRNA) or siRNA transfection; on the following day, cells were transfected using Lipofectamine 2000 (Invitrogen), according to the manufacturer’s instructions. At 48 h post transfection, cells were harvested for quantitative reverse-transcription-PCR (qRT-PCR) analysis. For lentiviral transduction, lentiviruses were used to infect 5 × 10^5^ cells seeded in six-well plates by using 4–6 μg/mL polybrene (107689, Sigma-Aldrich), after which the infected cells were selected using 1 μg/mL puromycin (Catalog Number 540411, Calbiochem, USA). Stable knockdown cell lines were identified using qRT-PCR.

### qRT-PCR

The total RNA was isolated from cells and tissue samples by using Trizol reagent (Invitrogen) according to the manufacturer’s protocols, and 0.5 μg of the RNA was reverse-transcribed into cDNA by using a High-Capacity cDNA Reverse Transcription Kit (Applied Biosystems, USA). The SYBR Green PCR Master Mix Kit (Applied Biosystems) was used to quantify RNA levels, with GAPDH or U6 serving as an internal control. qRT-PCR was performed on a 7500 FAST Real-Time PCR System (Applied Biosystems).

### Cell-viability assays

The viability of treated cells was quantified using the Cell Counting Kit-8 (CCK-8, Dojindo Laboratories, Kumamoto, Japan) assay, according to the manufacturer’s instructions. Briefly, cells were seeded in 96-well microtiter plates at a density of 3–5 × 10^3^ cells/well with 100 μL of the medium, CCK-8 solution was added to each well, and the plates were incubated at 37 °C for 60 min. Next, the 450-nm absorbance of each cell suspension was measured using a microplate reader, with a medium containing 10% CCK-8 serving as a control.

### Colony-formation assays

In total, 1000× cells were plated in a six-well plate and cultured in RPMI-1640 or DMEM medium containing 10% FBS for 14 days. Colonies were fixed with 4% methanol for 30 min, and 500 μL of 0.5% crystal violet (Catalog #: 332488, Sigma-Aldrich, St. Louis, MO, USA) was added to each well for 30 min to visualize the colonies for counting.

### Invasion assays

Cells in serum-free RPMI-1640 or DMEM medium were placed in the BRAND^®^ Insert with Matrigel (Catalog #: BR782806, Sigma-Aldrich, USA). RPMI-1640 or DMEM medium containing 10% FBS was added to the lower chamber. After incubating at 37 °C for 24 h, the noninvading cells that remained in the top chamber were removed with a cotton swab, and the cells that had migrated to the underside of the membrane were fixed with 100% methanol for 30 min, air-dried, stained with 0.5% crystal violet, imaged, and counted under a light microscope.

### Wound-healing assay

Cells were plated in six-well culture plates and cultivated to achieve over 90% confluence in RPMI-1640 or DMEM medium containing 5% FBS. A vertical wound per well was created using a 10-µL pipette tip. After two washes with PBS to eliminate cell debris, the cells were reincubated in RPMI-1640 or DMEM medium containing 0.1% FBS. Images were captured at the indicated time to measure the size of the remaining wound.

### Western blotting

Cells were lysed in lysis buffer containing 150 mmol/L NaCl, 1% Triton X-100, 5 mmol/L EDTA, 5000 U/mL aprotinin, 20 mg/mL leupeptin, 1 mmol/L phenylmethylsulfonylfluoride, 2 mmol/L sodium orthovanadate, 50 mmol/L NaF, 5% glycerol, 10 mmol/L Tris-HCl (pH 7.4), and 2% SDS. Next, protein concentrations were measured using a protein assay kit (Catalog #: 5000001, Bio-Rad, Richmond, CA), and then equal amounts of protein were separated using SDS-PAGE and electroblotted onto nitrocellulose membranes, which were blocked (overnight at 4 °C) with 5% nonfat milk in 0.1% Tween-20/TBST. The membranes were immunoblotted with primary antibodies from Cell Signaling Technology (CST) against EGFR (#2085), p-EGFR (#11862), JAK2 (Catalog #: 3230), p-JAK2 (Catalog #: 3771), STAT3 (Catalog #: 9139), and p-STAT3 (Catalog #: 9145), washed with Tween-20/PBS, and incubated with horseradish peroxidase-conjugated secondary antibodies for 1 h. After washing with Tween-20/PBS, protein bands on the membranes were visualized using an enhanced chemiluminescence detection system (Western Lightning, Perkin-Elmer, Norwalk, CT, USA).

### Animal experiments

Animal experiments were approved by the Medical Experimental Animal Care Commission of Harbin Medical University. Six-to-eight-week female Balb/c mice were obtained from Beijing Vital River Laboratory Animal Technology Company. Approximately 5 × 10^4^ cells transfected with 4T1-Scramble or 4T1-Sh Tincr shRNA were suspended in 200 μL of serum-free medium and injected directly into the right mammary fat pad. Gefitinib (Catalog #: S1025, Selleck) was administered orally for 7 days at a dose of 50 mg/kg^52^. Tumor growth was measured using calipers once every 3 days, and tumor volume was calculated as tumor volume = 1/2 (length × width^2^). After the mice were euthanized at the endpoint, the tumors induced by the injections were dissected out and weighed.

### Immunohistochemistry

Paraffin-embedded tissue sections of 100 breast-cancer tissues were deparaffinized in xylene, rehydrated in a graded series of ethanol solutions, and incubated for 20 min in 3% H_2_O_2_ to block endogenous peroxidase activity. Next, the sections were heated in target-retrieval solution (Dako) for 15 min in a microwave oven (Oriental Rotor) for antigen retrieval. After blocking nonspecific binding by incubating sections with 10% goat serum for 2 h at room temperature, the sections were incubated overnight at 4 °C with anti-EGFR, anti-JAK2, or anti-STAT3 primary antibody. Subsequently, sections were incubated with an appropriate secondary antibody for 20 min at 37 °C, and binding was visualized using 3,3’-diaminobenzidine tetrahydrochloride. After each treatment, sections were washed thrice with TBST for 5 min.

### RNA-FISH assay

ViewRNA^®^Probe (Catalogue Number VA1-3016120, Santa Clara) was purchased to perform FISH assay according to the manufacturer’s protocol. TINCR hybridization was carried out in a moist chamber. After digestion with a working protease solution, slides were incubated with RNase III (AM2290, Life Technologies, USA) or RNase A (AM2272, Life Technologies) for 2 h. Standard immunofluorescence and imaging was performed by confocal microscopy.

### RNA-immunoprecipitation (RIP) assay

RIP was performed using a Magna RNA-binding protein immunoprecipitation kit (Catalog #: 17-700 Millipore, Bedford, MA, USA), according to the manufacturer’s instructions. Briefly, cell lysates were incubated with RIP buffer containing magnetic beads conjugated with negative-control normal mouse IgG or human anti-DNMT1 antibody. The samples were then incubated with Proteinase K to isolate the immunoprecipitated RNA. Last, the purified RNAs were extracted and analyzed using real-time PCR to confirm the presence of the binding targets.

### Bisulfite-sequencing PCR (BSP)

To measure the methylation levels on the miR-503 locus promoter, BSP was conducted as described in our previous study^53^. Briefly, online tools were used to analyze the CpG islands in the miR-503 locus promoter, and CpG sites at the 5ʹ end of the promoter, within the sequence ranging from nucleotide −310 to 92, were selected for BSP analysis. Genomic DNA was isolated and modified with bisulfite, and the bisulfite-treated DNA was PCR-amplified. Subsequently, the PCR products were separated on gels, and the bands of the correct size were excised, and the PCR products were purified and subcloned into pTG19-T vector; positive clones were obtained through ampicillin antibiotic selection, and ten positive clones were subject to DNA sequencing at Generay Biotech Co., Ltd. (Shanghai, China).

### Chromatin-immunoprecipitation (ChIP) assay

ChIP assays were performed using a ChIP Assay Kit (Catalog #: P2078, Beyotime, Shanghai, China), according to the manufacturer’s protocol with slight modifications. Cells were cross-linked for 10 min with 1% formaldehyde, and after terminating the reaction by adding 0.125 mol/L glycine (final concentration), sonicated cell lysates were prepared, and DNA was immunoprecipitated using an anti-STAT3 antibody (Catalog #: 9139, CST) or anti-DNMT1 antibody (Catalog #: GTX116011, GeneTex); IgG (BD Biosciences, San Diego, CA, USA) served as the negative control. PCR was performed on the immunoprecipitated DNA to amplify the binding sites, and the amplified fragments were analyzed on agarose gels. Chromatin (10%) from before the immunoprecipitation was used as the input control.

### Dual-luciferase-reporter assay

The full-length 3ʹ-untranslated regions (UTRs) of human TINCR and EGFR were PCR-amplified and the fragments were cloned separately into the multiple-cloning sites in psi-CHECK-2 luciferase miRNA-expression reporter vector. HEK293T cells (plated at ~40–50% confluence) were used for the dual-luciferase-reporter assays, and Lipofectamine 2000 (Catalog #: 11668500, Invitrogen) was used to transfect the cells with 20 μmol/L hsa-miR-503-5p mimic or negative-control (NC) mimic and 0.5 mg of the plasmid. Luciferase activities were measured at 48 h post transfection by using a dual-luciferase-reporter assay kit (Catalog #: E1910, Promega, USA) and a luminometer (GloMax^TM^ 20/20, Promega, USA).

### Statistical analyses

The expression of each lncRNA was dichotomized by using the median expression as the cutoff to define high values (at or above the median) versus low values (below the median). The differences in the results of the in vitro and in vivo experiments between groups were analyzed using Student’s *t* test. Spearman’s correlation coefficients were calculated for correlation analyses. All experiments were performed independently in triplicate. All statistical tests were two-sided, and *P* < 0.05 was considered statistically significant.

## Supplementary information

Supplementary legends

Supplementary Figure. S1

Supplementary Figure. S2

Supplementary Figure. S3

Supplementary Figure. S4

Supplementary Figure. S5

Supplementary Figure. S6

Supplementary Table S1

Supplementary Table S2

Supplementary Table S3

Supplementary Table S4

Supplementary Table S5

Supplementary Table S6
