# Supplementary material for: LncRNA TINCR favors tumorigenesis via STAT3–TINCR–EGFR-feedback loop by recruiting DNMT1 and acting as a competing endogenous RNA in human breast cancer
Source: Cell Death Dis. 2021 Jan 14;12(1):83. doi: 10.1038/s41419-020-03188-0 (PMC7809450; doi:10.1038/s41419-020-03188-0)
Supplement: Supplementary file 1 — Supplementary legends [file 41419_2020_3188_MOESM1_ESM.docx]

**LncRNA TINCR favors tumorigenesis via STAT3-TINCR-EGFR feedback loop by recruiting DNMT1 and acting as a competing endogenous RNA in human breast cancer**

Qin Wang^1^, Jiena Liu^1^, Zilong You^1^, Yanling Yin^1^, Lei Liu^1^, Yujuan Kang^1^, Siwei Li^1^, Shipeng Ning^1^, Hui Li^1^, Yajie Gong^1^, Shouping Xu^1,*^ and Da Pang^1,^ ^2,*^

**Supplementary legends**

**Supplementary Table S1**. **Clinical information of 250 tissue specimens—125 breast cancer tissues and 125 adjacent normal tissues—from HMUCC.**

**Supplementary Table S2**. **GO and KEGG analysis of potential function of TINCR. *P* < 0.05 indicated statistical significance.**

**Supplementary Table S3**. **GEO database with two TINCR probes 244374-at /229385-at.**

**Supplementary Table S4**. **Immunohistochemistry assays of EGFR protein and JAK2 protein expression in TINCR^-high^ breast cancer tissues and in TINCR^-low^ ones in HMUCC cohort.**

**Supplementary Table S5.** **Public databases including TargetScan, RNA22, starBase databases and data of transcriptome microRNA sequencing performing in HMUCC cohort.**

**Supplementary Table S6.** **Bisulfite sequencing methylation sites of TINCR on CpG island of miR-503-5p locus.**

**Supplementary Fig. S1 High expression of TINCR was likely associated with poor OS in the HMUCC cohort.**

**Supplementary Fig. S2 The basal expression of EGFR in cell lines from CCLE database (a) and our chort (b).**

**Supplementary Fig. S3** **EGFR protein expression is higher in TINCR^-high^ breast cancer tissues than in TINCR^-low^ ones and vice versa in 122 patients in HMUCC cohort.**

**Supplementary Fig. S4 Positive correlation between expression of TINCR and JAK2 score in breast cancer samples in HMUCC cohort (n = 52).**

**Supplementary Fig. S5 EGFR, JAK2 and STAT3 expression in cancer tissues and normal tissues from HMUCC cohort.**

**Supplementary Fig. S6 Subcellular localization of TINCR in UACC812 cell lines, assessed using RNA-FISH assay.**
