# Supplementary figures and images for: LncRNA TINCR favors tumorigenesis via STAT3–TINCR–EGFR-feedback loop by recruiting DNMT1 and acting as a competing endogenous RNA in human breast cancer
Source: Cell Death Dis. 2021 Jan 14;12(1):83. doi: 10.1038/s41419-020-03188-0 (PMC7809450; doi:10.1038/s41419-020-03188-0)

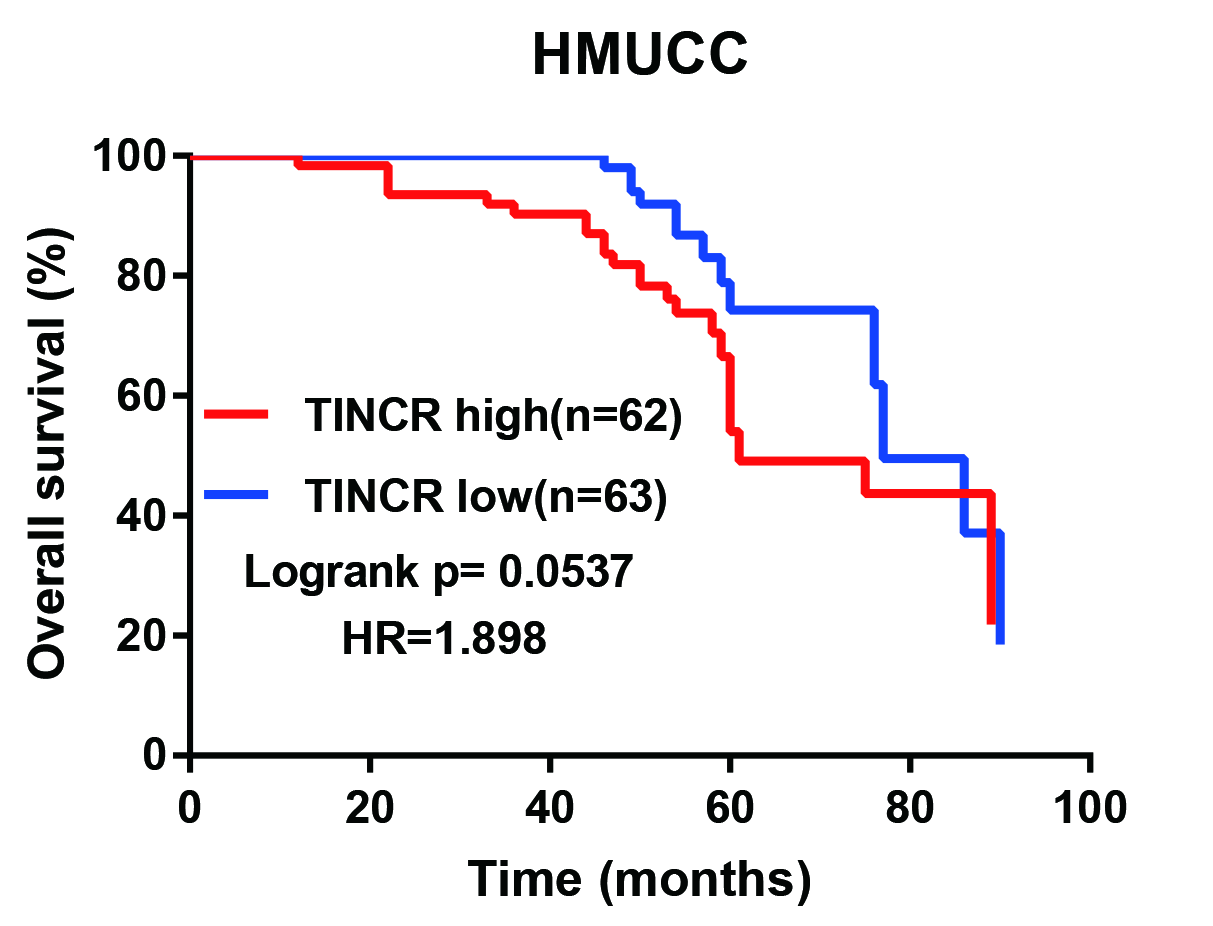

Supplement: Supplementary file 2 — Supplementary Figure. S1 [file 41419_2020_3188_MOESM2_ESM.tif]

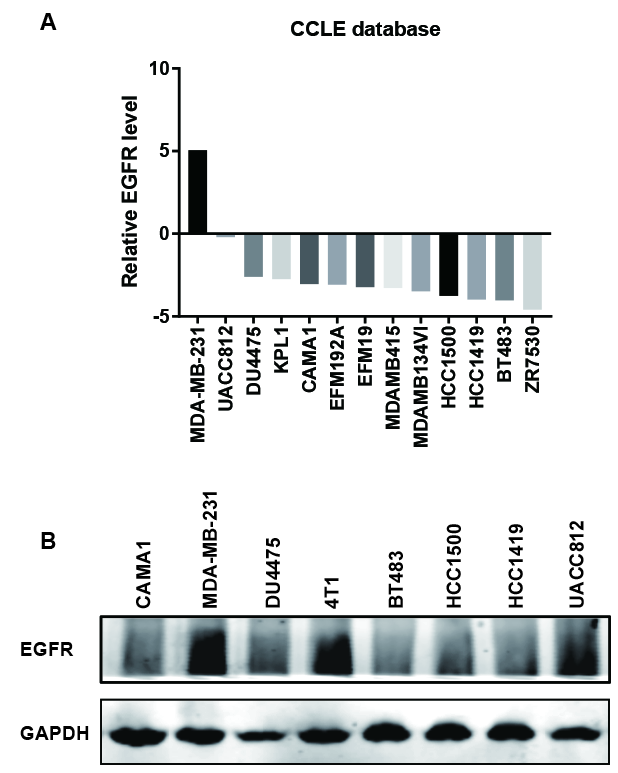

Supplement: Supplementary file 3 — Supplementary Figure. S2 [file 41419_2020_3188_MOESM3_ESM.tif]

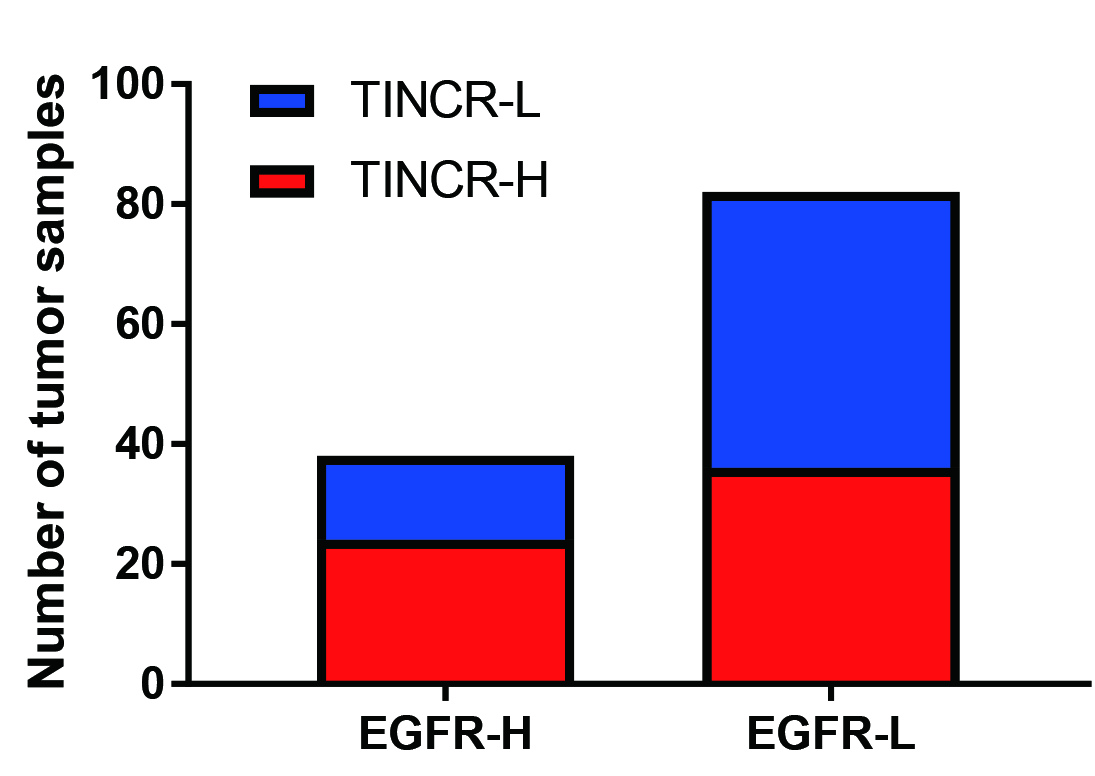

Supplement: Supplementary file 4 — Supplementary Figure. S3 [file 41419_2020_3188_MOESM4_ESM.tif]

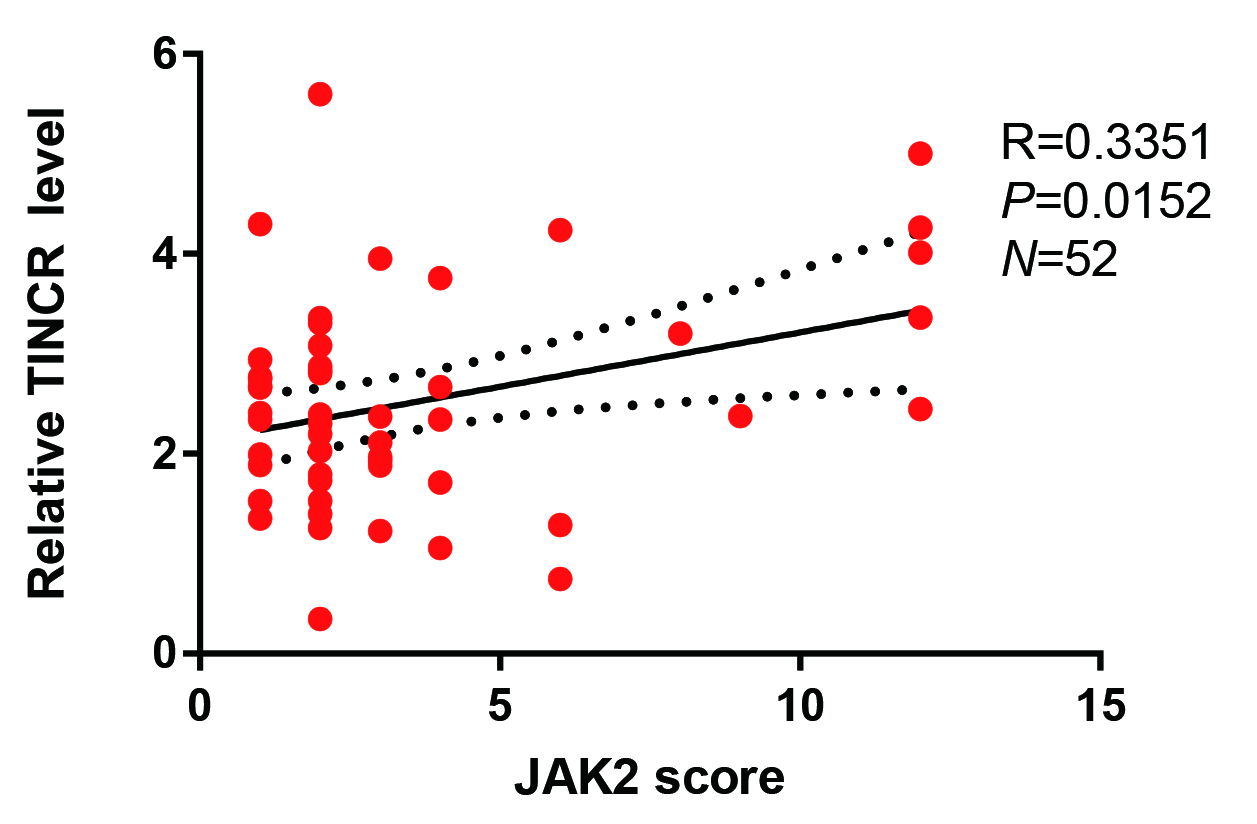

Supplement: Supplementary file 5 — Supplementary Figure. S4 [file 41419_2020_3188_MOESM5_ESM.tif]

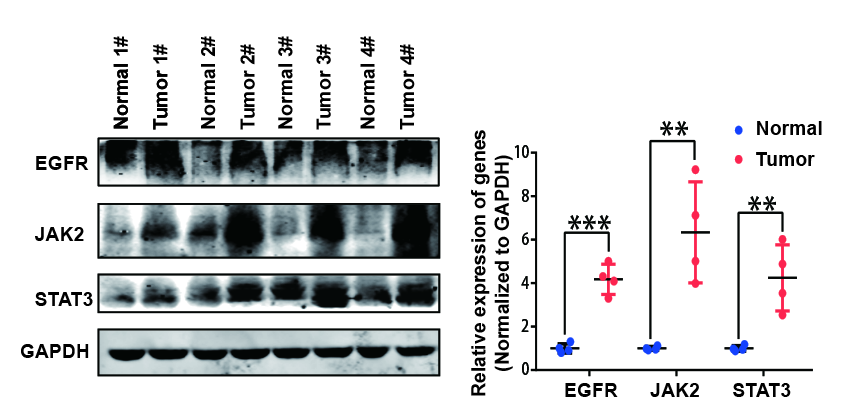

Supplement: Supplementary file 6 — Supplementary Figure. S5 [file 41419_2020_3188_MOESM6_ESM.tif]

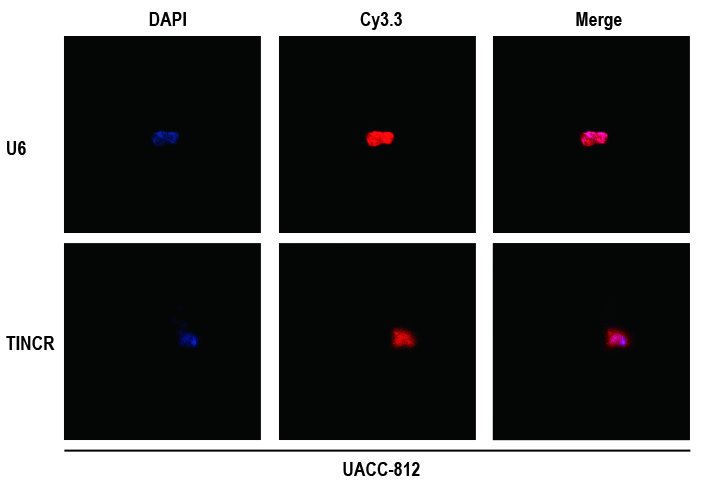

Supplement: Supplementary file 7 — Supplementary Figure. S6 [file 41419_2020_3188_MOESM7_ESM.tif]
