# Supplementary material for: LncRNA TINCR favors tumorigenesis via STAT3–TINCR–EGFR-feedback loop by recruiting DNMT1 and acting as a competing endogenous RNA in human breast cancer
Source: Cell Death Dis. 2021 Jan 14;12(1):83. doi: 10.1038/s41419-020-03188-0 (PMC7809450; doi:10.1038/s41419-020-03188-0)
Supplement: Supplementary file 9 — Supplementary Table S2 [file 41419_2020_3188_MOESM9_ESM.pdf]

a.COR

Correlated Gene	Spearman's Correlation	p-Value	Correlated Gene	Spearman's Correlation	p-Value	Correlated Gene	Spearman's Correlation	p-Value
MEAK7	0.444397	1.38E-53	SYT12	0.226356	4.88E-14	CDON	-0.20814	4.70E-12
DBNDD1	0.399647	9.35E-43	PSORS1C2	0.226316	4.93E-14	SUSD3	-0.20824	4.58E-12
HR	0.393644	2.01E-41	DDX19A	0.226261	5.00E-14	BCAT2	-0.20857	4.23E-12
TMEM40	0.380388	1.41E-38	CALB2	0.226248	5.02E-14	ZNF407	-0.20862	4.19E-12
FUT3	0.37779	4.90E-38	DNAJC9	0.225716	5.77E-14	FRMPD2	-0.20874	4.07E-12
B3GNT3	0.370716	1.39E-36	PLOD1	0.225583	5.97E-14	SELENOP	-0.20886	3.95E-12
NCCRP1	0.369515	2.43E-36	MYO1E	0.225514	6.08E-14	PRDX3	-0.20887	3.94E-12
CLDN23	0.362023	7.55E-35	GTPBP4	0.224958	7.03E-14	MTX3	-0.20942	3.45E-12
SDC1	0.361878	8.06E-35	FADS6	0.224802	7.32E-14	TRIM8	-0.20945	3.42E-12
KPNA7	0.353579	3.26E-33	C11ORF80	0.224408	8.11E-14	ZNF839	-0.20955	3.34E-12
KRT80	0.350523	1.24E-32	LPAR2	0.22438	8.17E-14	CDKL3	-0.20966	3.26E-12
SLC15A1	0.348151	3.46E-32	KLK6	0.22435	8.23E-14	MEGF8	-0.20981	3.14E-12
ZNRF1	0.347917	3.82E-32	PPIF	0.2241	8.78E-14	CYP2A7	-0.20991	3.06E-12
NPW	0.347675	4.24E-32	MIS18A	0.223969	9.09E-14	ARMT1	-0.20996	3.03E-12
TFAP2C	0.344179	1.89E-31	ACTR3	0.223968	9.09E-14	ABLIM3	-0.21	3.00E-12
TUBB3	0.342043	4.66E-31	TGFA	0.223767	9.57E-14	GALNT16	-0.21004	2.98E-12
SLC6A9	0.342039	4.67E-31	STAU1	0.223741	9.64E-14	MROH8	-0.21008	2.94E-12
NUP93	0.336492	4.72E-30	GINS1	0.223598	1.00E-13	SKAP1	-0.2101	2.93E-12
MUC16	0.334942	8.93E-30	DNER	0.223355	1.06E-13	CCNI	-0.21019	2.87E-12
CCNE1	0.327643	1.72E-28	MPZL2	0.223166	1.12E-13	IMPDH2	-0.21025	2.82E-12
KRT7	0.326656	2.54E-28	LY6E-DT	0.223134	1.13E-13	ABCG2	-0.21028	2.81E-12

GJB3	0.325285	4.38E-28	SPIRE2	0.223072	1.15E-13	CAPS2	-0.21065	2.57E-12
ADGRG1	0.324247	6.60E-28	CEBPG	0.22297	1.18E-13	TSPAN3	-0.21065	2.57E-12
TUBA1C	0.324134	6.90E-28	MEX3D	0.222944	1.18E-13	KCNJ11	-0.21065	2.56E-12
DUSP9	0.323312	9.54E-28	MTHFD2	0.222804	1.23E-13	IQGAP2	-0.21083	2.46E-12
S100A7	0.322292	1.42E-27	KARS	0.22273	1.25E-13	FSIP1	-0.21091	2.41E-12
HS3ST6	0.321236	2.15E-27	ABRACL	0.222568	1.30E-13	DBNDD2	-0.21103	2.34E-12
KDM7A-DT	0.320288	3.11E-27	TRPV6	0.222538	1.31E-13	UFSP2	-0.21108	2.31E-12
GRHL3	0.319913	3.60E-27	CORO1C	0.222419	1.36E-13	TMED8	-0.21111	2.29E-12
SBSN	0.319379	4.42E-27	PIMREG	0.222337	1.38E-13	TMEM25	-0.2114	2.14E-12
MAB21L4	0.318341	6.61E-27	GRHL1	0.222313	1.39E-13	N4BP2L2	-0.21152	2.07E-12
KRT81	0.317284	9.92E-27	CENPW	0.22208	1.48E-13	GASK1B	-0.21155	2.06E-12
MUC21	0.315917	1.68E-26	UTP4	0.222055	1.49E-13	MYOZ3	-0.2118	1.94E-12
ANLN	0.315608	1.89E-26	PADI1	0.221983	1.52E-13	TCTN1	-0.21207	1.81E-12
USB1	0.313459	4.27E-26	DBF4	0.221703	1.63E-13	PHF21A	-0.21218	1.77E-12
SLC2A1	0.313455	4.27E-26	SMIM13	0.22149	1.72E-13	ZNF25	-0.21221	1.75E-12
CBX2	0.31312	4.85E-26	SLC22A20P	0.221418	1.75E-13	FAM81B	-0.21225	1.74E-12
PTTG1IP	0.313029	5.02E-26	SUV39H1	0.221416	1.75E-13	BMPR1B	-0.21236	1.69E-12
INAVA	0.312572	5.97E-26	RHCG	0.221343	1.79E-13	RETREG1	-0.21284	1.50E-12
NRBP1	0.311945	7.56E-26	FAM171A2	0.221286	1.81E-13	FHIT	-0.21298	1.45E-12
SLURP1	0.310927	1.11E-25	PPM1G	0.221279	1.82E-13	SLITRK6	-0.21301	1.44E-12
ULBP2	0.310602	1.25E-25	NUF2	0.221231	1.84E-13	RAB17	-0.21327	1.35E-12
SLC6A17	0.309904	1.62E-25	KRT6A	0.221203	1.85E-13	CCDC91	-0.21351	1.27E-12
KCNG1	0.309536	1.86E-25	VSTM2L	0.220948	1.98E-13	IGBP1	-0.21356	1.26E-12
LAD1	0.309383	1.97E-25	NRBF2	0.220912	1.99E-13	CHN2	-0.2136	1.25E-12
TMEM189	0.308315	2.93E-25	C10ORF55	0.22091	1.99E-13	CCNDBP1	-0.21367	1.23E-12

B3GNT4	0.307321	4.24E-25	MND1	0.220822	2.04E-13	KYAT1	-0.21367	1.23E-12
PTPRU	0.307094	4.61E-25	DDX39A	0.220811	2.05E-13	TRMO	-0.2137	1.22E-12
MMP1	0.307004	4.77E-25	GPR87	0.220759	2.07E-13	STRADB	-0.21374	1.21E-12
AUNIP	0.306741	5.25E-25	NDC80	0.220716	2.10E-13	BECN1	-0.21375	1.20E-12
ADGRF4	0.305603	7.99E-25	CDH3	0.220622	2.15E-13	PCM1	-0.21394	1.15E-12
S100A11	0.305366	8.71E-25	DESI2	0.220549	2.19E-13	HPN	-0.214	1.13E-12
PSMD7	0.304877	1.04E-24	TAF7L	0.22051	2.21E-13	LYPD6B	-0.21406	1.11E-12
TPD52L2	0.304389	1.25E-24	SLPI	0.220412	2.26E-13	THAP6	-0.21427	1.06E-12
KCTD5	0.303257	1.88E-24	TAF4	0.220351	2.30E-13	DEFB132	-0.21454	9.88E-13
ATP6V1C2	0.303245	1.89E-24	TMEM54	0.220341	2.31E-13	METTL25	-0.21455	9.87E-13
CALML5	0.302273	2.69E-24	MRPL15	0.220297	2.33E-13	RAPGEF3	-0.21463	9.66E-13
ORC6	0.301677	3.34E-24	VGF	0.220173	2.41E-13	ZBTB25	-0.21464	9.65E-13
SLC7A5	0.299955	6.21E-24	GPI	0.220136	2.43E-13	LINC00957	-0.21464	9.65E-13
UPK2	0.299895	6.35E-24	LRRC59	0.220042	2.49E-13	TMEM9B	-0.21495	8.93E-13
KRT83	0.298666	9.86E-24	AGO2	0.22003	2.50E-13	FBXL5	-0.21503	8.76E-13
CLIC3	0.297897	1.30E-23	SRPK3	0.219786	2.66E-13	CPB1	-0.21527	8.25E-13
PLEKHN1	0.296856	1.88E-23	TBC1D7	0.219757	2.67E-13	PHYHD1	-0.21528	8.24E-13
MYBL2	0.296656	2.02E-23	CCNE2	0.219748	2.68E-13	ABHD10	-0.21529	8.21E-13
RAET1L	0.29615	2.41E-23	SCAF4	0.219585	2.79E-13	KLHDC2	-0.21543	7.93E-13
B3GNT7	0.295867	2.67E-23	FAM72B	0.219584	2.79E-13	EPHX2	-0.21573	7.35E-13
S100A7A	0.29529	3.27E-23	TAS1R3	0.219493	2.86E-13	NXPE3	-0.2158	7.23E-13
DNMT3B	0.294154	4.88E-23	GGH	0.219482	2.87E-13	RPGR	-0.21591	7.03E-13
MYH14	0.29368	5.76E-23	RAET1G	0.21947	2.88E-13	ATP6V1G2	-0.21596	6.94E-13
RCC1	0.293256	6.68E-23	C1QL4	0.219357	2.96E-13	CCDC30	-0.21602	6.85E-13
IVL	0.293057	7.16E-23	SGO1	0.219305	3.00E-13	C6ORF201	-0.21602	6.85E-13

PFKP	0.293018	7.26E-23	FAM47C	0.219264	3.03E-13	HPS4	-0.2162	6.55E-13
PPP2R2C	0.292879	7.62E-23	CSNK2A2	0.219245	3.05E-13	MAOA	-0.21621	6.53E-13
UNC13D	0.292831	7.75E-23	TONSL	0.219205	3.08E-13	PIH1D2	-0.21623	6.51E-13
DONSON	0.292705	8.10E-23	ZNF217	0.219196	3.08E-13	ANKS1B	-0.21636	6.29E-13
KCTD15	0.292657	8.24E-23	FUT6	0.219053	3.20E-13	ZNF763	-0.21638	6.25E-13
NCS1	0.292447	8.86E-23	TMEM65	0.218945	3.29E-13	FAN1	-0.2164	6.22E-13
ZNF488	0.290959	1.49E-22	KIF4B	0.218898	3.33E-13	ARL6	-0.21647	6.11E-13
PGLYRP4	0.290403	1.80E-22	LMO1	0.21875	3.45E-13	PCBD2	-0.21654	6.02E-13
TICRR	0.290303	1.87E-22	MYL12A	0.218727	3.47E-13	DYNC2H1	-0.21662	5.90E-13
DGCR5	0.290297	1.87E-22	SLC28A1	0.218674	3.52E-13	MYL5	-0.21668	5.81E-13
LSR	0.289995	2.08E-22	FZD9	0.218586	3.60E-13	SAMD15	-0.2167	5.77E-13
L1CAM	0.289539	2.43E-22	EFNA2	0.218527	3.65E-13	HEMK1	-0.21676	5.69E-13
CPA4	0.289537	2.43E-22	AVEN	0.218399	3.77E-13	WDR35	-0.21692	5.46E-13
EPOP	0.288649	3.30E-22	CDC25A	0.218276	3.89E-13	CENPP	-0.21694	5.45E-13
MCM4	0.287936	4.21E-22	NCAPH	0.21827	3.90E-13	NAT1	-0.21727	5.01E-13
AURKA	0.287528	4.85E-22	ULBP3	0.218178	3.99E-13	CCDC173	-0.21728	5.00E-13
CABLES2	0.287264	5.30E-22	RAD54L	0.218131	4.03E-13	PTCD2	-0.21777	4.42E-13
SHCBP1	0.287101	5.61E-22	CDCA3	0.217893	4.28E-13	GATA3	-0.2182	3.96E-13
SLC44A2	0.286525	6.82E-22	RTKN2	0.217822	4.36E-13	ZNF620	-0.21824	3.92E-13
CAVIN4	0.286323	7.31E-22	RAB22A	0.21749	4.74E-13	DUSP28	-0.2183	3.87E-13
AIF1L	0.285935	8.34E-22	GPR37L1	0.217412	4.83E-13	NUDT16P1	-0.21837	3.80E-13
CENPN	0.285817	8.68E-22	ANKRD34B	0.21731	4.96E-13	AUH	-0.21838	3.79E-13
A2ML1	0.285591	9.37E-22	H2BFXP	0.21728	5.00E-13	RASA4CP	-0.2186	3.59E-13
ASS1	0.284967	1.16E-21	HCCS	0.217272	5.01E-13	GRIA1	-0.2187	3.49E-13
C15ORF39	0.284865	1.20E-21	P2RY6	0.21716	5.15E-13	SIAH2	-0.2187	3.49E-13

ULBP1	0.284313	1.44E-21	TMEM158	0.217118	5.20E-13	GLCCI1	-0.21881	3.40E-13
CDCA8	0.283874	1.67E-21	ACTB	0.216793	5.65E-13	DMAC2L	-0.21883	3.38E-13
GABBR2	0.283495	1.90E-21	SH2B2	0.216587	5.94E-13	MEIS3P1	-0.21885	3.37E-13
RBM38	0.282767	2.43E-21	MYD88	0.216566	5.97E-13	ZCWPW2	-0.21904	3.21E-13
TMEM79	0.282108	3.03E-21	YWHAZ	0.216562	5.98E-13	AP1AR	-0.21929	3.01E-13
RFWD3	0.282053	3.09E-21	PLAAT1	0.216507	6.06E-13	TBC1D14	-0.21943	2.91E-13
TMEM105	0.280096	5.92E-21	KLHDC7B	0.216464	6.13E-13	CFAP44	-0.21945	2.89E-13
S100A9	0.280045	6.02E-21	CLDN7	0.216455	6.14E-13	LANCL1	-0.21965	2.75E-13
ABCD1	0.279597	6.99E-21	CALU	0.216352	6.30E-13	FBP1	-0.21991	2.57E-13
GINS3	0.279389	7.49E-21	CCK	0.216272	6.43E-13	FAM227B	-0.22017	2.41E-13
GAL	0.27921	7.94E-21	GJB4	0.21626	6.45E-13	BBS4	-0.22059	2.17E-13
SLC16A13	0.279165	8.06E-21	NECTIN4	0.216151	6.63E-13	USP30	-0.22059	2.17E-13
S100P	0.279059	8.35E-21	RRP1B	0.216145	6.64E-13	CAMK1	-0.22061	2.15E-13
GATA5	0.278924	8.73E-21	GTF2IRD1	0.215965	6.94E-13	AGTR1	-0.22085	2.03E-13
NUTF2	0.278676	9.47E-21	TUBA1B	0.215852	7.14E-13	KCNE4	-0.22092	1.99E-13
TLE1	0.278237	1.09E-20	ALYREF	0.215821	7.19E-13	MAP3K12	-0.22108	1.91E-13
GALNS	0.278229	1.10E-20	ASCL2	0.215365	8.06E-13	TMEM161B	-0.22115	1.88E-13
MMP15	0.277828	1.25E-20	MCU	0.215297	8.19E-13	ARL17A	-0.22115	1.88E-13
FAM83D	0.277797	1.27E-20	AADAT	0.215211	8.37E-13	SNX1	-0.22163	1.66E-13
STIL	0.277603	1.35E-20	TUBB6	0.214995	8.83E-13	MAML3	-0.22187	1.56E-13
RASGRF1	0.277597	1.35E-20	SPINDOC	0.214807	9.25E-13	LIAS	-0.22203	1.50E-13
S100A8	0.277567	1.36E-20	DUS2	0.214714	9.47E-13	FAM172A	-0.22213	1.46E-13
CLDN9	0.276091	2.21E-20	CPPED1	0.214512	9.96E-13	RBM5	-0.22214	1.45E-13
KRT86	0.275703	2.51E-20	GJB5	0.214398	1.02E-12	TMEM128	-0.22247	1.34E-13
KREMEN2	0.27517	2.99E-20	SPNS2	0.214384	1.03E-12	ZNF540	-0.22251	1.32E-13

NXPH4	0.275098	3.06E-20	TUBB	0.214067	1.11E-12	C2ORF73	-0.22258	1.30E-13
STK38	0.275055	3.10E-20	RNF222	0.21404	1.12E-12	FBXO4	-0.2226	1.29E-13
PLK1	0.274987	3.17E-20	PSME4	0.213985	1.13E-12	CLDN12	-0.22336	1.06E-13
TMEM51	0.274472	3.75E-20	OLFM2	0.213954	1.14E-12	SYT9	-0.22347	1.03E-13
MTSS2	0.273965	4.41E-20	FIBCD1	0.213925	1.15E-12	ZNF493	-0.22353	1.02E-13
NTAN1	0.2737	4.81E-20	LINC01096	0.213899	1.16E-12	PATZ1	-0.22362	9.94E-14
UBE2C	0.273576	5.00E-20	DDX28	0.213673	1.22E-12	HEBP1	-0.2239	9.24E-14
CIB2	0.273364	5.36E-20	YWHAQ	0.213634	1.24E-12	LINC02381	-0.22401	8.98E-14
ERCC6L	0.273184	5.68E-20	CTNNBIP1	0.213633	1.24E-12	SNRPN	-0.22406	8.88E-14
NXN	0.27317	5.70E-20	CLDN4	0.213541	1.27E-12	ARHGEF3	-0.22417	8.62E-14
MESP2	0.273074	5.88E-20	SLC35A2	0.213474	1.29E-12	NAPB	-0.2242	8.55E-14
DIRAS1	0.272701	6.63E-20	DTNA	0.213425	1.30E-12	DTX3	-0.22421	8.54E-14
CFAP20	0.271796	8.87E-20	CHFR	0.213309	1.34E-12	RAB30	-0.22426	8.42E-14
SUMO3	0.271517	9.70E-20	PXDC1	0.213167	1.39E-12	ZNF214	-0.22439	8.15E-14
RAP2B	0.270809	1.22E-19	ADARB1	0.213116	1.40E-12	PWARSN	-0.22454	7.83E-14
GAN	0.270406	1.38E-19	CCNA2	0.213107	1.41E-12	DCAF16	-0.22483	7.26E-14
PCP4L1	0.27025	1.45E-19	RASGEF1C	0.213093	1.41E-12	ANGEL1	-0.22489	7.16E-14
CPNE2	0.269977	1.59E-19	DEPDC1	0.213065	1.42E-12	NME5	-0.22497	7.00E-14
S100A10	0.269964	1.59E-19	EBP	0.213008	1.44E-12	N4BP2L1	-0.22505	6.87E-14
ACTN4	0.269927	1.61E-19	SYT16	0.212703	1.55E-12	AFF3	-0.22505	6.87E-14
MELK	0.269633	1.77E-19	CARM1	0.212649	1.57E-12	WDR6	-0.22549	6.13E-14
RASAL1	0.269529	1.83E-19	UHRF1	0.21225	1.74E-12	TLE3	-0.2257	5.79E-14
SOX11	0.269469	1.86E-19	ANKRD27	0.211951	1.87E-12	ATP6AP1L	-0.22575	5.71E-14
SPSB1	0.269124	2.08E-19	REEP4	0.211836	1.92E-12	NMNAT3	-0.22575	5.71E-14
PSCA	0.268993	2.17E-19	SRD5A1	0.211772	1.95E-12	AGR3	-0.22606	5.27E-14

CDC42BPG	0.268589	2.46E-19	RAE1	0.211769	1.95E-12	NEK9	-0.22614	5.17E-14
CIAPIN1	0.268515	2.52E-19	NUDT8	0.211654	2.01E-12	LINC02447	-0.22619	5.09E-14
RRM2	0.268506	2.53E-19	MGC2889	0.211648	2.01E-12	CCNG1	-0.2262	5.09E-14
MTFR2	0.268186	2.80E-19	PTPRH	0.211638	2.02E-12	ANOS1	-0.22635	4.89E-14
FANCA	0.267041	4.01E-19	TTYH3	0.211489	2.09E-12	ALKBH8	-0.22659	4.59E-14
EMC8	0.266949	4.13E-19	FXYD5	0.211467	2.10E-12	DEGS2	-0.22687	4.27E-14
SLC66A3	0.26693	4.16E-19	ABTB2	0.211341	2.17E-12	CDK17	-0.22717	3.94E-14
PERP	0.266787	4.35E-19	TRIB3	0.211231	2.23E-12	POLL	-0.2275	3.62E-14
JPT1	0.266394	4.92E-19	FAM110A	0.211106	2.30E-12	POLN	-0.22784	3.30E-14
CDCA2	0.266048	5.48E-19	SERPINH1	0.210907	2.41E-12	METTL15	-0.22787	3.28E-14
MGAT5B	0.265844	5.84E-19	USP6NL	0.210822	2.46E-12	C2CD5	-0.22799	3.17E-14
RSU1	0.265707	6.09E-19	CDH16	0.2108	2.47E-12	LYPD6	-0.22815	3.05E-14
WNT3A	0.265017	7.56E-19	SHMT2	0.210769	2.49E-12	TMBIM4	-0.22817	3.03E-14
TH	0.264912	7.81E-19	USP39	0.210694	2.54E-12	BBS12	-0.22823	2.98E-14
IQGAP3	0.264162	9.86E-19	KDELR3	0.210584	2.61E-12	CLGN	-0.22826	2.96E-14
CBFB	0.263795	1.10E-18	CDK16	0.210583	2.61E-12	CYP21A2	-0.22826	2.95E-14
OVOL1	0.263659	1.15E-18	HASPIN	0.21051	2.65E-12	RNASE4	-0.22886	2.52E-14
VPS35	0.263502	1.21E-18	SEPTIN3	0.210499	2.66E-12	TXNDC15	-0.22922	2.29E-14
P4HA2	0.263195	1.33E-18	CARHSP1	0.210461	2.68E-12	CYFIP2	-0.22926	2.27E-14
ZSWIM4	0.263193	1.33E-18	EFS	0.210452	2.69E-12	CRY2	-0.22936	2.21E-14
CDC20	0.263018	1.41E-18	ARID3C	0.210424	2.71E-12	SLC25A12	-0.22937	2.20E-14
ANXA4	0.262821	1.49E-18	MPHOSPH10	0.210421	2.71E-12	ETNPPL	-0.22945	2.16E-14
CLDN14	0.262638	1.58E-18	LMNB2	0.210363	2.75E-12	XBP1	-0.22958	2.08E-14
GUCA1A	0.262635	1.58E-18	FOSL1	0.21035	2.76E-12	BBS5	-0.22993	1.90E-14
PBDC1	0.262604	1.60E-18	ATP11A	0.210334	2.77E-12	ABHD14A	-0.22994	1.89E-14

CDC25B	0.262537	1.63E-18	EHD1	0.210322	2.78E-12	PJA2	-0.23036	1.69E-14
RAD51	0.262463	1.67E-18	RBM17	0.210168	2.88E-12	POMT2	-0.2305	1.63E-14
CDR2	0.26238	1.71E-18	CEBPB	0.21013	2.91E-12	LONRF2	-0.23072	1.54E-14
PSMA7	0.262223	1.80E-18	CASP14	0.210021	2.99E-12	OCIAD1	-0.23088	1.47E-14
PLCB3	0.261821	2.03E-18	HSPA14	0.20995	3.04E-12	DOK1	-0.23089	1.47E-14
GNAS	0.261605	2.17E-18	THEG	0.209885	3.09E-12	DCLK1	-0.23107	1.40E-14
TEX30	0.261508	2.24E-18	BORA	0.209792	3.16E-12	ZNF141	-0.23111	1.39E-14
NSDHL	0.26148	2.26E-18	PITPNM3	0.209564	3.34E-12	ASB16	-0.23111	1.39E-14
MPHOSPH6	0.26146	2.27E-18	OPN3	0.209563	3.34E-12	ZNF516	-0.2313	1.32E-14
UNC93A	0.260923	2.68E-18	FAM72D	0.209562	3.34E-12	PARP3	-0.23158	1.22E-14
TTK	0.260066	3.48E-18	ACTL8	0.209562	3.34E-12	LZTFL1	-0.23181	1.15E-14
KIF4A	0.260062	3.48E-18	RAB5IF	0.209547	3.35E-12	LRRC49	-0.23186	1.13E-14
NDRG4	0.25981	3.76E-18	ADAM9	0.209518	3.37E-12	CGRRF1	-0.2319	1.12E-14
TMSB10	0.259679	3.91E-18	CCT5	0.209397	3.47E-12	THSD4	-0.23213	1.05E-14
LINC01588	0.259523	4.10E-18	ABCA12	0.209022	3.80E-12	MDH1B	-0.23214	1.05E-14
CHRM1	0.259431	4.22E-18	DHX38	0.208938	3.88E-12	RAMP2	-0.23251	9.50E-15
BUB1	0.258843	5.04E-18	UBE2T	0.208854	3.96E-12	ANKAR	-0.23265	9.16E-15
SUSD2	0.258817	5.09E-18	S1PR5	0.208832	3.98E-12	GLRB	-0.23272	8.98E-15
MESP1	0.258801	5.11E-18	DMRT1	0.208751	4.06E-12	RAD17	-0.23274	8.94E-15
KIF1A	0.258656	5.34E-18	PCSK1N	0.208615	4.19E-12	NAP1L5	-0.2332	7.89E-15
HJURP	0.258246	6.04E-18	MACC1	0.208613	4.19E-12	KBTBD3	-0.23334	7.61E-15
MFAP3L	0.258169	6.19E-18	MYPN	0.208538	4.27E-12	TRIM66	-0.23338	7.53E-15
CCNB2	0.25811	6.30E-18	AMER1	0.208494	4.31E-12	NADK2	-0.23341	7.46E-15
SAPCD2	0.258096	6.33E-18	TMEM151A	0.208445	4.37E-12	ECI2	-0.23355	7.18E-15
SF3B3	0.257893	6.73E-18	ITPR3	0.208443	4.37E-12	NTN4	-0.23359	7.10E-15

FRMD8	0.25717	8.36E-18	CFDP1	0.208192	4.64E-12	CYP4X1	-0.23365	6.99E-15
KLRG2	0.257097	8.55E-18	TMC6	0.208055	4.79E-12	DNAH7	-0.23382	6.66E-15
HES2	0.25675	9.49E-18	CCDC168	0.207998	4.86E-12	SPAG8	-0.23407	6.23E-15
PADI2	0.256412	1.05E-17	MSANTD3	0.207986	4.87E-12	NBEA	-0.23421	6.01E-15
SLC52A2	0.256005	1.19E-17	POC1A	0.207959	4.90E-12	CYB5A	-0.23427	5.90E-15
MUC5B	0.255972	1.20E-17	CDK1	0.207952	4.91E-12	DYNC2LI1	-0.23458	5.42E-15
FAM83B	0.255829	1.25E-17	FAM131C	0.207946	4.92E-12	8-Mar	-0.23495	4.91E-15
C11ORF86	0.255668	1.31E-17	GTSE1	0.207924	4.95E-12	TUBG2	-0.23514	4.66E-15
PKMYT1	0.25561	1.34E-17	SH3GL3	0.20786	5.02E-12	BHLHE40	-0.23534	4.41E-15
PERM1	0.255542	1.36E-17	BAK1	0.207779	5.12E-12	CST9	-0.23545	4.27E-15
CHMP1A	0.255501	1.38E-17	DNAJB1	0.207776	5.12E-12	PLA2G12A	-0.23559	4.12E-15
CENPA	0.255383	1.43E-17	DIAPH3	0.20764	5.29E-12	LRTOMT	-0.23595	3.73E-15
NHSL1	0.255284	1.47E-17	CIAO2B	0.207591	5.36E-12	SOX2-OT	-0.23596	3.73E-15
KIF2C	0.255079	1.57E-17	ZC3H18	0.207577	5.37E-12	CYBRD1	-0.23597	3.71E-15
COL13A1	0.25471	1.75E-17	ZNF185	0.207555	5.40E-12	C3ORF18	-0.23618	3.51E-15
HEBP2	0.253676	2.38E-17	EDC4	0.207534	5.43E-12	INTU	-0.23622	3.47E-15
ADGRG6	0.253319	2.64E-17	CLTCL1	0.207519	5.45E-12	CCDC170	-0.23624	3.45E-15
NOTCH1	0.2533	2.66E-17	RHBDF2	0.207505	5.47E-12	DNAJC19	-0.23631	3.38E-15
PGBD5	0.25312	2.80E-17	PRDX1	0.207329	5.70E-12	CCDC158	-0.23646	3.25E-15
ELOVL1	0.252967	2.93E-17	SAE1	0.207283	5.76E-12	ZNF182	-0.2369	2.88E-15
DLX3	0.252939	2.96E-17	DSCC1	0.207277	5.77E-12	GRIK1-AS1	-0.23697	2.82E-15
UBXN2A	0.252921	2.97E-17	CHD5	0.20716	5.94E-12	KANSL3	-0.23709	2.73E-15
CNIH2	0.252366	3.50E-17	MSL3P1	0.207124	5.99E-12	CPLX1	-0.23719	2.66E-15
GPRIN1	0.252305	3.57E-17	RSPO4	0.206992	6.18E-12	CASD1	-0.23724	2.62E-15
KRT78	0.251799	4.14E-17	CA5BP1	0.206844	6.40E-12	ZNF483	-0.23757	2.39E-15

CDCA4	0.251416	4.63E-17	CLSPN	0.206704	6.62E-12	MBLAC2	-0.23772	2.29E-15
TPM3	0.251392	4.67E-17	PRC1	0.206601	6.78E-12	IGIP	-0.23777	2.26E-15
CDT1	0.251292	4.80E-17	KIF18A	0.206492	6.96E-12	DLG4	-0.23782	2.23E-15
LINC00592	0.25117	4.98E-17	DNMT3A	0.206399	7.11E-12	PSD4	-0.23811	2.06E-15
VAC14	0.251146	5.01E-17	MSL3	0.206358	7.18E-12	NUDT16	-0.2382	2.01E-15
SNAI1	0.25086	5.45E-17	CCZ1P-OR7E38P	0.206352	7.19E-12	CD302	-0.23833	1.94E-15
CRYBG2	0.250265	6.49E-17	GINS4	0.206212	7.44E-12	ARHGAP35	-0.23837	1.91E-15
CSTB	0.250192	6.63E-17	PRIM2	0.206176	7.50E-12	DPY19L2P4	-0.23862	1.79E-15
CCNF	0.249813	7.40E-17	FLVCR2	0.206057	7.72E-12	FBXL17	-0.23877	1.71E-15
VGLL1	0.2498	7.43E-17	PPP4C	0.205965	7.89E-12	ANKRD42	-0.23895	1.63E-15
SRC	0.24977	7.50E-17	RTN4R	0.205949	7.91E-12	NICN1	-0.23992	1.25E-15
TMEM132A	0.249707	7.64E-17	KLK5	0.20592	7.97E-12	ACBD4	-0.24078	9.80E-16
LRRC3	0.249673	7.71E-17	MFGE8	0.205845	8.11E-12	CFAP61	-0.24081	9.72E-16
TES	0.249581	7.92E-17	NRTN	0.205833	8.14E-12	PCAT18	-0.24136	8.33E-16
MCM10	0.249432	8.27E-17	SECTM1	0.205689	8.42E-12	LTA4H	-0.24139	8.25E-16
E2F2	0.249387	8.39E-17	UBE2I	0.20565	8.50E-12	GLYATL1	-0.2414	8.23E-16
DDX27	0.249191	8.87E-17	MRPS6	0.205624	8.55E-12	ZBTB4	-0.24159	7.81E-16
CHAC1	0.248977	9.45E-17	SLC9A2	0.205546	8.71E-12	POLI	-0.24201	6.93E-16
OSBPL2	0.24892	9.60E-17	ESYT3	0.205453	8.90E-12	DNAAF4	-0.24217	6.63E-16
RIPK4	0.248878	9.72E-17	PWP2	0.20541	8.99E-12	ATP1A1-AS1	-0.24267	5.75E-16
KIF23	0.248758	1.01E-16	MARVELD3	0.205145	9.57E-12	NEDD4L	-0.24276	5.61E-16
CSMD2	0.248265	1.16E-16	PAQR4	0.205056	9.78E-12	TRIM52	-0.24329	4.83E-16
DLL3	0.248206	1.18E-16	UBE2S	0.205052	9.79E-12	RNF157	-0.24333	4.77E-16
HTR1D	0.247965	1.27E-16	SPRR1B	0.205024	9.85E-12	SLC39A6	-0.24374	4.26E-16
PADI3	0.24785	1.31E-16	POLQ	0.204862	1.02E-11	NBR1	-0.24385	4.12E-16

DHTKD1	0.247625	1.40E-16	ETFA	0.204853	1.03E-11	CYB5D1	-0.24392	4.04E-16
TNFSF9	0.247557	1.43E-16	ACOT7	0.204828	1.03E-11	TMEM232	-0.24396	4.00E-16
SLC5A6	0.247552	1.43E-16	PDXK	0.204818	1.03E-11	CPEB2	-0.24401	3.94E-16
TMEFF1	0.247534	1.43E-16	GEN1	0.204807	1.04E-11	DRC3	-0.24413	3.81E-16
TPX2	0.247453	1.47E-16	IMPA2	0.204797	1.04E-11	EZH1	-0.24464	3.29E-16
ST14	0.247369	1.50E-16	TMEM51-AS1	0.204672	1.07E-11	ICE2	-0.24467	3.26E-16
LCN2	0.247195	1.58E-16	OGFOD1	0.20448	1.12E-11	ESR1	-0.24492	3.04E-16
RGS9BP	0.247151	1.60E-16	PSPH	0.204468	1.12E-11	GMPR2	-0.24551	2.57E-16
TRIM47	0.247057	1.65E-16	FANCI	0.204462	1.12E-11	CBX7	-0.24597	2.25E-16
SEMA7A	0.246993	1.68E-16	PDIA6	0.204427	1.13E-11	COQ10A	-0.24617	2.12E-16
APOBEC3B	0.246474	1.95E-16	SUV39H2	0.204225	1.19E-11	TBC1D19	-0.24623	2.09E-16
C1ORF198	0.245988	2.24E-16	AWAT2	0.204172	1.20E-11	DNAJC24	-0.24652	1.92E-16
ELF4	0.245651	2.47E-16	DTX2	0.204126	1.22E-11	FUCA1	-0.24676	1.79E-16
PKP1	0.245607	2.50E-16	TYRO3	0.203859	1.30E-11	CCNG2	-0.24694	1.70E-16
TCHHL1	0.245451	2.61E-16	TRIP13	0.20383	1.30E-11	ARL14EP	-0.24708	1.63E-16
SKA3	0.245273	2.75E-16	TREM1	0.203821	1.31E-11	CST3	-0.24728	1.54E-16
ADRM1	0.245199	2.81E-16	NRDC	0.203584	1.38E-11	DNAL1	-0.24767	1.38E-16
PSMB2	0.244889	3.07E-16	KLHL30	0.203545	1.39E-11	SPOP	-0.24771	1.36E-16
EZH2	0.244781	3.16E-16	HSPH1	0.203447	1.43E-11	MYCBPAP	-0.24778	1.34E-16
POR	0.244688	3.25E-16	LYPD3	0.203436	1.43E-11	EPB41L4A-AS1	-0.24789	1.30E-16
SDCBP2	0.244442	3.48E-16	BRD7	0.203394	1.44E-11	SPATA6L	-0.24791	1.29E-16
TNFRSF12A	0.244377	3.55E-16	KLK7	0.203349	1.46E-11	UNC119B	-0.24857	1.06E-16
NECTIN1	0.244245	3.68E-16	KRT3	0.203078	1.56E-11	ZNF280D	-0.24867	1.03E-16
LRP8	0.244202	3.73E-16	APEX2	0.203023	1.58E-11	IMPACT	-0.24914	9.01E-17
KRT16	0.243822	4.15E-16	CCNB1	0.202951	1.60E-11	SLC45A1	-0.24918	8.91E-17

G6PD	0.243712	4.28E-16	NANOS1	0.202833	1.65E-11	LIFR	-0.24918	8.90E-17
PLAUR	0.243433	4.64E-16	FER1L4	0.202783	1.67E-11	LINC00908	-0.24924	8.76E-17
ABCC2	0.243288	4.83E-16	RDH13	0.202721	1.69E-11	DNAJC12	-0.24955	7.99E-17
KPNA2	0.243136	5.04E-16	EXOSC6	0.202571	1.75E-11	IGFALS	-0.24964	7.78E-17
NTSR1	0.243113	5.08E-16	TSPYL5	0.202471	1.79E-11	TCEAL1	-0.24979	7.46E-17
KCNK12	0.242851	5.47E-16	ATP2B2	0.202329	1.85E-11	METAP1D	-0.24987	7.28E-17
CWH43	0.242833	5.50E-16	KIFC3	0.202115	1.95E-11	CHIC1	-0.24992	7.18E-17
ACOT9	0.242752	5.62E-16	CRABP1	0.202035	1.98E-11	ETFRF1	-0.2501	6.80E-17
CALHM3	0.242533	5.98E-16	DLGAP3	0.202013	1.99E-11	MRPS30	-0.25046	6.13E-17
NIPAL1	0.242528	5.99E-16	FOXM1	0.201985	2.01E-11	LINC00324	-0.25046	6.13E-17
KCNN4	0.24217	6.63E-16	HTATSF1	0.201867	2.06E-11	EFCAB12	-0.25111	5.06E-17
AKR1B15	0.242166	6.63E-16	BARX1	0.20183	2.08E-11	MLEC	-0.25115	5.01E-17
DLGAP5	0.242018	6.92E-16	NUDT5	0.201753	2.12E-11	GNPDA2	-0.25146	4.58E-17
IGF2BP1	0.241828	7.30E-16	SLC35C1	0.201649	2.17E-11	ZNF226	-0.25165	4.33E-17
PHLDA2	0.241771	7.42E-16	SPRR2D	0.201628	2.18E-11	TTC14	-0.25165	4.32E-17
NELFCD	0.241722	7.52E-16	TTC7A	0.201578	2.20E-11	ZC3H6	-0.25182	4.11E-17
SLC30A3	0.241583	7.82E-16	CCT6A	0.201573	2.21E-11	GIN1	-0.25192	4.00E-17
NIP7	0.241514	7.97E-16	PSAPL1	0.201332	2.33E-11	JADE2	-0.25195	3.95E-17
TMC7	0.241456	8.10E-16	PRR11	0.201249	2.38E-11	GLIPR1L2	-0.25258	3.29E-17
ENTPD2	0.241389	8.26E-16	HIST1H2BJ	0.201203	2.40E-11	SETBP1	-0.25341	2.57E-17
DPM1	0.240947	9.35E-16	SNRPA1	0.201202	2.40E-11	MAGI2	-0.25402	2.15E-17
CASP3	0.240839	9.63E-16	SLC6A19	0.201162	2.43E-11	OSGEPL1	-0.2542	2.04E-17
RNF114	0.2408	9.74E-16	TXN	0.201147	2.44E-11	KIZ	-0.2542	2.03E-17
PCBP3	0.240715	9.97E-16	OPRK1	0.201089	2.47E-11	EGOT	-0.2545	1.86E-17
USH1G	0.240232	1.14E-15	MOCS3	0.201073	2.48E-11	GLI3	-0.25479	1.71E-17

DLGAP4	0.240132	1.17E-15	LINC01634	0.201067	2.48E-11	FRY	-0.25481	1.70E-17
MROH6	0.240106	1.18E-15	PKM	0.201015	2.51E-11	MCC	-0.25483	1.69E-17
SPTBN2	0.239879	1.26E-15	TMCC2	0.200982	2.53E-11	SEZ6L	-0.25533	1.45E-17
EFNA3	0.239865	1.26E-15	DKC1	0.200975	2.53E-11	SPATA4	-0.25592	1.22E-17
WDR62	0.23984	1.27E-15	UMODL1	0.200967	2.54E-11	SLC25A45	-0.25659	9.97E-18
KIF20A	0.2398	1.29E-15	SKA1	0.200963	2.54E-11	CTC1	-0.25697	8.89E-18
PPP1R14C	0.239707	1.32E-15	STMN1	0.200923	2.56E-11	CNTD1	-0.25709	8.56E-18
CHAF1B	0.239671	1.34E-15	IGF2BP2	0.200713	2.69E-11	SERPINA11	-0.25713	8.47E-18
ACP7	0.239419	1.43E-15	HSP90AA1	0.200653	2.73E-11	TCTN2	-0.25715	8.42E-18
CD24	0.23897	1.62E-15	RACGAP1	0.200545	2.80E-11	NDFIP1	-0.25728	8.09E-18
KRT9	0.238815	1.69E-15	TPM4	0.200501	2.83E-11	CCNH	-0.25781	6.89E-18
GIPC1	0.238706	1.75E-15	PIEZO1	0.200474	2.84E-11	TNS2	-0.25801	6.49E-18
MTHFD1L	0.238702	1.75E-15	OLIG1	0.200267	2.98E-11	NUDT6	-0.25842	5.74E-18
VSIG10L	0.238573	1.81E-15	PGLYRP3	0.200193	3.03E-11	TMEM144	-0.25844	5.70E-18
SMYD2	0.238527	1.83E-15	HNRNPAB	0.200184	3.04E-11	FAM110B	-0.25887	5.00E-18
HAUS8	0.238512	1.84E-15	NMU	0.200091	3.11E-11	BTRC	-0.25922	4.51E-18
MYCN	0.238482	1.86E-15	TEAD3	0.200088	3.11E-11	VWA2	-0.25963	3.97E-18
KIFC1	0.23824	1.99E-15	LINC00938	-0.20003	3.15E-11	KIF13B	-0.25979	3.78E-18
CMC2	0.238106	2.06E-15	PHGR1	-0.2001	3.10E-11	TTC41P	-0.25981	3.76E-18
PGRMC1	0.238057	2.09E-15	IVD	-0.20035	2.93E-11	C5ORF63	-0.25983	3.73E-18
PLP2	0.237802	2.24E-15	C12ORF76	-0.20044	2.87E-11	ERBB4	-0.25991	3.64E-18
PNP	0.237723	2.29E-15	MZF1	-0.20052	2.81E-11	TRAK1	-0.26005	3.49E-18
NAE1	0.237663	2.33E-15	ZNF227	-0.20057	2.78E-11	CASC1	-0.26015	3.39E-18
CAMK1D	0.237544	2.41E-15	MTMR10	-0.20057	2.78E-11	ARSG	-0.26028	3.26E-18
GALNT3	0.237269	2.60E-15	FBXO36	-0.20073	2.68E-11	NUMA1	-0.26174	2.08E-18

FGFR4	0.23702	2.78E-15	EDEM1	-0.20076	2.67E-11	EFHC1	-0.2618	2.04E-18
RYR1	0.236589	3.13E-15	TRIM45	-0.2008	2.64E-11	MLLT3	-0.26241	1.70E-18
LY6E	0.236514	3.20E-15	HSD17B8	-0.20083	2.62E-11	ZSCAN26	-0.26268	1.56E-18
IL36RN	0.236507	3.20E-15	C20ORF96	-0.20087	2.60E-11	THTPA	-0.26277	1.52E-18
CAMP	0.236459	3.25E-15	C5AR2	-0.20093	2.56E-11	RABEP1	-0.2628	1.50E-18
SCEL	0.236407	3.29E-15	PRKAB1	-0.20116	2.43E-11	EWSAT1	-0.26287	1.47E-18
KLF5	0.236156	3.53E-15	C9ORF64	-0.20122	2.40E-11	CCDC183	-0.26302	1.41E-18
PDSS1	0.236122	3.56E-15	ENPP4	-0.2013	2.35E-11	ZNF546	-0.26302	1.40E-18
ZNF695	0.236024	3.66E-15	CEP120	-0.20134	2.33E-11	SNHG8	-0.26315	1.35E-18
SMOX	0.235999	3.68E-15	PACRGL	-0.20142	2.28E-11	SEPSECS	-0.26322	1.32E-18
RAET1K	0.235803	3.89E-15	CFAP43	-0.20158	2.21E-11	TBC1D9	-0.26368	1.14E-18
RHBDL2	0.235676	4.02E-15	FGD3	-0.20175	2.12E-11	STK32B	-0.26377	1.11E-18
SLC9A4	0.235555	4.16E-15	PPM1M	-0.20181	2.09E-11	NPHP1	-0.26473	8.26E-19
TMSB15B	0.235483	4.24E-15	IDUA	-0.20188	2.05E-11	MYO18B	-0.26518	7.19E-19
RCC2	0.235432	4.30E-15	KIAA1328	-0.20198	2.01E-11	GRK3	-0.26532	6.89E-19
CHD7	0.235344	4.41E-15	KIF12	-0.20202	1.99E-11	POLK	-0.26559	6.33E-19
AMMECR1	0.235169	4.62E-15	KLHL32	-0.20206	1.97E-11	LYRM9	-0.26564	6.22E-19
HOMER3	0.235153	4.64E-15	ZNF721	-0.20209	1.96E-11	MAATS1	-0.26583	5.87E-19
COLGALT1	0.235053	4.77E-15	ZBTB14	-0.2021	1.95E-11	SUGT1P3	-0.26598	5.59E-19
GPT2	0.234872	5.01E-15	KIAA1324	-0.20234	1.85E-11	BBS1	-0.26678	4.35E-19
TGM1	0.234772	5.15E-15	SPATA24	-0.2025	1.78E-11	CIRBP	-0.26738	3.61E-19
EXO1	0.234724	5.22E-15	ARFIP2	-0.20257	1.75E-11	CALCOCO1	-0.26753	3.44E-19
GSDMC	0.234531	5.50E-15	RAPGEF4	-0.20258	1.75E-11	ZSCAN30	-0.26797	3.00E-19
CYP27C1	0.234449	5.62E-15	SRSF5	-0.20267	1.71E-11	ELP2	-0.26822	2.77E-19
BIRC5	0.234335	5.80E-15	LRP2	-0.20269	1.70E-11	NOSTRIN	-0.26882	2.29E-19

PLA2G15	0.23433	5.81E-15	CLASP2	-0.2027	1.70E-11	NEK10	-0.26888	2.24E-19
EN1	0.234271	5.90E-15	FAM174A	-0.20278	1.67E-11	SALL2	-0.27044	1.37E-19
SMURF1	0.233924	6.48E-15	CST5	-0.20283	1.65E-11	DUBR	-0.27161	9.43E-20
KCNF1	0.233488	7.30E-15	LINC00663	-0.20287	1.63E-11	BORCS7	-0.27172	9.11E-20
DPYSL5	0.233377	7.52E-15	PEX12	-0.20291	1.62E-11	BTF3	-0.27272	6.60E-20
MVD	0.233322	7.63E-15	NSA2	-0.20311	1.55E-11	PCSK6	-0.27273	6.58E-20
ORC1	0.23323	7.83E-15	HDAC7	-0.20312	1.54E-11	BTG2	-0.27303	5.98E-20
VASN	0.233154	7.99E-15	TTC8	-0.20314	1.53E-11	CCDC28A	-0.27336	5.37E-20
ABHD11	0.233103	8.10E-15	TBC1D15	-0.20316	1.52E-11	SERPINI1	-0.27372	4.78E-20
RGS20	0.233078	8.16E-15	SLC27A1	-0.20317	1.52E-11	WDR19	-0.27391	4.49E-20
PSAT1	0.23284	8.70E-15	ABHD1	-0.20331	1.47E-11	TPRG1	-0.2744	3.83E-20
UBE2A	0.232802	8.79E-15	CFB	-0.20333	1.47E-11	NUDT12	-0.27445	3.77E-20
AURKB	0.232792	8.81E-15	DCTN4	-0.20336	1.45E-11	ZNF740	-0.27464	3.55E-20
CTSV	0.232678	9.09E-15	PPP1R3E	-0.20337	1.45E-11	DNAH5	-0.2747	3.47E-20
SMIM5	0.232627	9.21E-15	ZCCHC4	-0.20342	1.44E-11	MOAP1	-0.27489	3.27E-20
F11R	0.232553	9.40E-15	BRD8	-0.20346	1.42E-11	GATA3-AS1	-0.2757	2.51E-20
APBA2	0.2325	9.53E-15	FAM161B	-0.20351	1.41E-11	VAMP2	-0.2759	2.35E-20
C1ORF105	0.232316	1.00E-14	CA14	-0.20352	1.40E-11	FMO5	-0.27597	2.30E-20
CLCN5	0.232091	1.06E-14	HTR7P1	-0.20358	1.38E-11	CYP2B7P	-0.27623	2.12E-20
TROAP	0.232079	1.07E-14	TMED7	-0.20358	1.38E-11	FAM47E	-0.2763	2.06E-20
ARHGEF4	0.231945	1.11E-14	CCDC191	-0.2036	1.38E-11	CAMLG	-0.27641	2.00E-20
YTHDF1	0.23192	1.11E-14	ATPAF1	-0.20373	1.34E-11	QDPR	-0.27656	1.90E-20
TCP1	0.231704	1.18E-14	CACNA1D	-0.20385	1.30E-11	TAPT1-AS1	-0.27736	1.46E-20
C16ORF87	0.23157	1.22E-14	PCED1A	-0.20388	1.29E-11	IKZF5	-0.27836	1.05E-20
TCF3	0.231363	1.29E-14	MED13L	-0.2039	1.28E-11	RERG	-0.27842	1.03E-20

SLC10A3	0.231322	1.31E-14	CHRD	-0.20397	1.26E-11	C4A	-0.27873	9.30E-21
STIP1	0.231205	1.35E-14	PHYKPL	-0.20409	1.23E-11	SLC46A1	-0.27959	6.99E-21
CDH1	0.231193	1.35E-14	TRMT10A	-0.2041	1.23E-11	TTC39C	-0.28034	5.46E-21
SLCO4A1	0.231071	1.40E-14	PTGR2	-0.20414	1.21E-11	CHD3	-0.28176	3.40E-21
HPCAL1	0.230682	1.55E-14	CFAP36	-0.20426	1.18E-11	EVL	-0.28242	2.73E-21
DSC2	0.230071	1.83E-14	SIRT3	-0.20427	1.18E-11	TTC23L	-0.28269	2.49E-21
MSI1	0.229687	2.03E-14	NEAT1	-0.20431	1.17E-11	FAM114A2	-0.28307	2.20E-21
DHCR7	0.229614	2.06E-14	SLC30A9	-0.20442	1.14E-11	HSD17B4	-0.28355	1.87E-21
CLDN3	0.229499	2.13E-14	EFCAB6	-0.20461	1.09E-11	PIGH	-0.28392	1.65E-21
E2F8	0.229461	2.15E-14	LTBP3	-0.20473	1.05E-11	GSAP	-0.28394	1.64E-21
GARS	0.229289	2.25E-14	DRAIC	-0.20489	1.02E-11	SPEF2	-0.28422	1.49E-21
UCA1	0.22928	2.26E-14	FBXW4	-0.20503	9.83E-12	B4GAT1	-0.28506	1.12E-21
KHSRP	0.229242	2.28E-14	FAAH2	-0.20505	9.80E-12	CREBL2	-0.28509	1.11E-21
CENPI	0.22913	2.35E-14	RSBN1L	-0.20506	9.76E-12	PGPEP1	-0.28532	1.03E-21
HENMT1	0.22899	2.44E-14	SERF1A	-0.20551	8.79E-12	SYBU	-0.28547	9.76E-22
MNX1	0.228975	2.45E-14	ICA1	-0.2056	8.60E-12	MRPS27	-0.28554	9.54E-22
CEP55	0.2289	2.50E-14	PGGHG	-0.20561	8.59E-12	INPP5J	-0.28622	7.57E-22
PLAU	0.228806	2.56E-14	ABCC8	-0.20566	8.48E-12	JADE1	-0.28841	3.58E-22
CDCA5	0.228743	2.60E-14	TYW3	-0.20568	8.43E-12	CFAP69	-0.28844	3.55E-22
PALM2	0.228575	2.72E-14	SMAP2	-0.20578	8.23E-12	TRAM1L1	-0.28856	3.40E-22
BEND3	0.228557	2.73E-14	CEP126	-0.20607	7.69E-12	DCDC1	-0.28892	3.01E-22
PGK1	0.228195	3.01E-14	TMEM26	-0.20617	7.52E-12	XPA	-0.2894	2.55E-22
IL4R	0.228037	3.14E-14	SYNGAP1	-0.20624	7.39E-12	LRIG1	-0.2901	2.00E-22
COG4	0.227965	3.20E-14	EIF2A	-0.20643	7.06E-12	ACADSB	-0.29154	1.22E-22
CHRNA5	0.227908	3.24E-14	ZNF568	-0.20649	6.97E-12	ZNF396	-0.29217	9.76E-23

MCM6	0.227839	3.30E-14	CSNK1G3	-0.20652	6.91E-12	STEAP2	-0.29668	2.00E-23
MKI67	0.227692	3.44E-14	CARF	-0.20656	6.84E-12	TSHZ1	-0.29803	1.24E-23
CENPO	0.227622	3.50E-14	DNAJC18	-0.20667	6.67E-12	PHOSPHO2	-0.30017	5.75E-24
TAGLN2	0.227587	3.53E-14	ATP9B	-0.2067	6.63E-12	ITPR1	-0.30123	3.92E-24
KRT4	0.227313	3.80E-14	PAXIP1-AS2	-0.20672	6.59E-12	LINC00173	-0.30152	3.53E-24
SLC6A11	0.227296	3.81E-14	RCOR3	-0.20675	6.55E-12	LETMD1	-0.30196	3.01E-24
FAM178B	0.227154	3.96E-14	ABCD3	-0.20691	6.30E-12	NOVA1	-0.30273	2.28E-24
CDC45	0.227137	3.98E-14	SMAD4	-0.20709	6.04E-12	APH1B	-0.30291	2.13E-24
GRAMD2A	0.22713	3.98E-14	C5ORF15	-0.20714	5.96E-12	EIF4B	-0.30599	6.93E-25
RTL8A	0.22711	4.00E-14	ANKRD30B	-0.20732	5.72E-12	KLHDC1	-0.30672	5.30E-25
MRGBP	0.227064	4.05E-14	BCDIN3D-AS1	-0.20735	5.67E-12	CYB5D2	-0.31064	1.23E-25
MTFR1	0.226995	4.13E-14	FGF10	-0.2074	5.60E-12	DELE1	-0.31065	1.23E-25
DYNC1LI2	0.22696	4.16E-14	SLC4A7	-0.20741	5.59E-12	NHLRC4	-0.3116	8.60E-26
C10ORF91	0.226955	4.17E-14	PHF1	-0.20743	5.57E-12	RAMP2-AS1	-0.31462	2.75E-26
LGALS3	0.226831	4.31E-14	RERE	-0.20743	5.57E-12	BCL2	-0.31578	1.77E-26
PRKAG3	0.226731	4.42E-14	CEP63	-0.20749	5.49E-12	RGS22	-0.32021	3.21E-27
B4GALT5	0.22647	4.74E-14	ZNF345	-0.20755	5.41E-12	HDHD2	-0.33106	4.34E-29
CPAMD8	0.226457	4.75E-14	SPATA6	-0.20758	5.37E-12	TAPT1	-0.33165	3.42E-29
ACAT2	0.226445	4.77E-14	SLC14A1	-0.20769	5.23E-12	ZADH2	-0.333	1.97E-29
TNNT2	0.226415	4.80E-14	PRRT3	-0.20799	4.86E-12	ZFAND4	-0.33488	9.16E-30
KCMF1	0.226377	4.85E-14	LPAR6	-0.20801	4.85E-12	PLCD4	-0.3481	3.54E-32
SPART	-0.20809	4.75E-12	PIEZO2	-0.20813	4.71E-12	LINC00472	-0.36496	1.98E-35

b. GO

Category	Term	Count	%	PValue	Genes	List Total	Pop Hits	Pop Total	Fold Enrichment	Bonferroni	Benjamini	FDR
GOTERM_BP_DIRECT	GO:0007067~mitotic nuclear division	44	3.600655	3.75E-10	BORA, PKMYT1, ANLN, AURKA, CEP55, AURKB, CCNG1, RCC1, CCNG2, FAM83D, KIF2C, NUMA1, MIS18A, CDCA2, BUB1, SKA3, SKA1, CDCA5, CCNA2, TUBB3, CDCA3,	1020	248	16792	2.92081	1.28E-06	1.28E-06	6.85E-07

					ERCC6L,<br>CDK1,<br>CENPN,<br>CCNF,<br>NUF2,<br>TPX2,<br>NDC80,<br>CDC20,<br>UBE2I,<br>BIRC5,<br>CEP63,<br>REEP4,<br>CDC25A,<br>CDC25B,<br>CCNB2,<br>RCC2,<br>SGO1,<br>PLK1,<br>CENPW,<br>NEK9,<br>HAUS8,<br>CHFR,<br>CLTCL1							
GOTERM_BP_DIRECT	GO:0051301~c		52	4.255319	4.95E-09	KIFC1,<br>BORA,<br>AURKA,	1020	350	16792	2.445894	1.69E-05	8.46E-06	9.04E-06

	ell divi sion				FAM83D, CCNE2, KIF2C, CCNE1, CDCA8, MIS18A, CDCA2, CABLES2, CDCA5, CCNA2, TUBA1B, CDCA4, TUBA1C, CDCA3, CDK1, CCNF, TPX2, UBE2I, UBE2C, CHMP1A, RCC2, SGO1, NEK9, HAUS8, CHFR, UBE2S,							

					RCC1, CCNG1, CCNG2, TUBB, NUMA1, NCAPH, USP39, BUB1, SKA3, CLASP2, SKA1, ERCC6L, NUF2, NDC80, BIRC5, CDC20, CEP63, REEP4, CDC25A, CDC25B, CCNB1, CCNB2, CENPW							
GOTERM_BP_DIRECT	GO:0051726~r	25	2.045827	3.61E-07	E2F2, CDK17, ADARB1,	1020	124	16792	3.319102	0.001233	4.11E-04	6.60E-04

	egulation of cell cycle				CCNI, BTRC, FOXM1, CCNF, PRR11, PKMYT1, CCNG1, CDKL3, MYBL2, CCNG2, CDC25A, SRC, CCNB1, CCNE2, CCNE1, BAK1, SRSF5, CCNB2, PLK1, CABLES2, CDK16, CCNDBP1							
GOTERM_BP_DIRECT	GO:0007062~siste	21	1.718494	3.18E-06	CENPO, CENPN, CENPP, KIF18A,	1020	103	16792	3.356482	0.010809	0.002713	0.005808

	r chromatid cohesion				NUF2, NDC80, BIRC5, CDC20, AURKB, CENPI, KIF2C, CDCA8, RCC2, SGO1, PLK1, CENPA, BUB1, SKA1, CLASP2, CDCA5, ERCC6L							
GOTERM_BP_DIRECT	GO:0000281~mitotic cytokinesis	11	0.900164	4.49E-06	KIF23, BBS4, KIF4B, KIF4A, CENPA, PLK1, ANLN, STMN1, CEP55,	1020	29	16792	6.24449	0.015237	0.003066	0.008206

					RACGAP1, KIF20A							
GOTERM_BP_DIRECT	GO:0000082~G1/S transition of mitotic cell cycle	19	1.554828	3.80E-05	CDK1, CCNH, DBF4, IQGAP3, PKMYT1, RCC1, MCM10, MCM4, CDC25A, MCM6, CDT1, CCNE2, CCNE1, CDC45, RRM2, PRIM2, ORC6, CDCA5, ORC1	1020	102	16792	3.06659	0.121745	0.021404	0.069361
GOTERM_BP_DIRECT	GO:0070052~mitotic	10	0.818331	4.83E-05	CCNB1, STIL, CEP126, WDR62, TTK,	1020	30	16792	5.487582	0.152141	0.023302	0.088171

	spindle organization				NDC80, AURKA, STMN1, CLASP2, RCC1							
GOTERM_BP_DIRECT	GO:0042267~natural killer cell mediated cytotoxicity	8	0.654664	5.22E-05	TUBB, RAET1G, ULBP3, CEBPG, ULBP1, ULBP2, RAET1L, PRDX1	1020	18	16792	7.316776	0.163545	0.022075	0.095402
GOTERM_BP_DIRECT	GO:0006270~DNA replication init	10	0.818331	8.48E-05	CCNE2, CCNE1, CDC45, GINS4, PRIM2, ORC6, MCM10, MCM4,	1020	32	16792	5.144608	0.251645	0.031695	0.154812

	iation				ORC1, MCM6						
GOTERM_BP_DIRECT	GO:0008544~epidermis development	16	1.309329	1.65E-04	KLK7, S100A7, KLK5, GJB5, GRHL3, GRHL1, SCEL, KRT9, PLOD1, CASP14, KRT16, SPRR2D, SPRR1B, OVOL1, CALML5, KRT83	1020	85	16792	3.09887	0.430667	0.054772	0.30061
GOTERM_BP_DIRECT	GO:0007059~chromosome segregat	14	1.145663	1.94E-04	CENPN, NUF2, NEK10, NDC80, UBE2I, RCC1, SGO1, HJURP,	1020	68	16792	3.389389	0.484772	0.058505	0.353806

	ion				MIS18A, CDCA2, SKA3, CENPW, NEK9, SKA1							
GOTERM_BP_DIRECT	GO:0060271~cilium morphogenesis	21	1.718494	2.04E-04	BBS4, BBS5, RPGR, CEP126, UNC119B, TTC8, PCM1, ARL6, DNAH5, BBS1, ACTR3, NOTCH1, WDR19, RAB17, CFAP20, DYNC2H1, TCTN1, TCTN2, WDR35, EHD1, INTU	1020	136	16792	2.542042	0.501765	0.056404	0.371666
GOTERM_B	GO:0	15	1.2	3.37E	KIF23,	1020	81	16792	3.048	0.684037	0.084812	0.61

P_DIRECT	0070 18~microtubule-based movement		274 96	-04	KIF4B, KIFC1, KIF12, KIF4A, DYNC1LI2, KIF18A, RACGAP1, DNAH5, KIFC3, KIF2C, KIF1A, DYNC2H1, KIF20A, KIF13B				656			3891
GOTERM_BP_DIRECT	GO:0 0062 60~DNA replication	22	1.8 003 27	4.50E -04	EXO1, POLL, CLSPN, CDK1, POLK, POLI, TICRR, DBF4, GINS3, GINS4, MCM10, MCM4,	1020	155	16792	2.336 648	0.785152	0.104027	0.81 8557

					CDC25A, CDT1, MCM6, CDC45, RRM2, ORC6, ORC1, CHAF1B, RAD17, DSCC1							
GOTERM_BP_DIRECT	GO:0042384~cilium assembly	19	1.554828	4.88E-04	BBS4, BBS5, RPGR, CEP126, ABLIM3, DYNC2LI1, TTC8, ARL6, PCM1, DNAH5, BBS1, NME5, WDR19, RAB17, DYNC2H1, TCTN2,	1020	124	16792	2.522517	0.811569	0.105301	0.888081

					WDR35, EHD1, INTU							
GOTERM_BP_DIRECT	GO:0000086~G2/M transition of mitotic cell cycle	20	1.636661	6.16E-04	CDK1, HSP90AA1, CCNH, BTRC, FOXM1, BORA, TPX2, PKMYT1, AURKA, BIRC5, CEP63, PCM1, CDC25A, CDC25B, CCNB1, TUBB, CCNB2, PLK1, HAUS8, MELK	1020	137	16792	2.40332	0.878443	0.123406	1.120015
GOTERM_BP_DIRECT	GO:0051256~mitot	5	0.409165	7.75E-04	KIF23, KIF4B, KIF4A, AURKB,	1020	8	16792	10.28922	0.929274	0.144287	1.405813

	ic spindle midzone assembly				RACGAP1							
GOTERM_BP_DIRECT	GO:0042493~response to drug	34	2.782324	8.69E-04	ASS1, CDH1, CDH3, PNP, CBX7, KCNJ11, SRC, BAK1, CASP3, BCL2, GATA3, TGFA, ABCD3, SRD5A1, HTR1D, FOSL1, DNMT3B, DNMT3A, CDK1, HSP90AA1, BECN1,	1020	304	16792	1.841228	0.94877	0.152174	1.575607

					SLC6A11, GGH, CST3, GAL, RAD54L, POR, RAD51, ABCG2, LCN2, CCNB1, GNAS, LRP8, ABCC8							
GOTERM_BP_DIRECT	GO:0016338~calcium-independent cell-cell adhesion via	7	0.572831	0.001213	CLDN7, CLDN9, CLDN4, CLDN3, CLDN12, CLDN23, CLDN14	1020	21	16792	5.487582	0.984197	0.196109	2.192357

	plasma membrane cell-adhesion molecules											
GOTERM_BP_DIRECT	GO:0007080~mitotic metaphase plate congression	9	0.736498	0.001426	CCNB1, KIFC1, KIF2C, CHMP1A, CDCA8, BECN1, KIF18A, CEP55, CDCA5	1020	37	16792	4.004452	0.992381	0.2164	2.573013
GOTERM_BP_DIRECT	GO:00070	6	0.4909	0.002027	KIZ, TTK, AURKA,	1020	16	16792	6.173529	0.999027	0.281237	3.638554

	51~spindle organization		98		AURKB, CHD3, AUNIP							
GOTERM_BP_DIRECT	GO:0030855~epithelial cell differentiation	12	0.981997	0.003119	CDK1, F11R, BARX1, LGALS3, RHCG, KRT3, KRT4, TAGLN2, PGK1, ANXA4, UPK2, ACTL8	1020	70	16792	2.822185	0.999977	0.384482	5.546489
GOTERM_BP_DIRECT	GO:0035108~limb morphogenesis	5	0.409165	0.003153	MEGF8, BAK1, ZNF141, BMPR1B, GLI3	1020	11	16792	7.483066	0.999979	0.374517	5.604982

GOTERM_BP_DIRECT	GO:0006306~DNA methylation	7	0.572831	0.003214	DNMT3A, BEND3, HENMT1, EZH2, GNAS, DNMT3B, HEMK1	1020	25	16792	4.609569	0.999983	0.367742	5.711215
GOTERM_BP_DIRECT	GO:0061436~establishment of skin barrier	6	0.490998	0.003592	CLDN4, KRT16, GRHL3, GRHL1, TMEM79, ABCA12	1020	18	16792	5.487582	0.999995	0.388611	6.362864
GOTERM_BP_DIRECT	GO:0006281~DNA repair	26	2.12766	0.004522	CLSPN, APEX2, TICRR, FOXM1, FAN1, FANCI, POLQ, FANCA, POLL,	1020	235	16792	1.82141	1	0.448861	7.945525

					EXO1, CDK1, POLK, UBE2A, POLI, GEN1, RAD54L, RAD51, XPA, UHRF1, RFWD3, BTG2, PARP3, PSME4, CHAF1B, RAD17, UBE2T							
GOTERM_BP_DIRECT	GO:0045862~positive regulation of	6	0.490998	0.004635	BAK1, FGFR4, PLK1, BTRC, CYFIP2, PERP	1020	19	16792	5.198762	1	0.444619	8.136588

	proteolysis											
GOTERM_BP_DIRECT	GO:0031069~hair follicle morphogenesis	7	0.572831	0.004843	NOTCH1, BCL2, FGF10, SNAI1, INTU, TMEM79, CTSV	1020	27	16792	4.268119	1	0.447118	8.486665
GOTERM_BP_DIRECT	GO:0007017~microtubule-based process	8	0.654664	0.00526	TUBB, NEK10, NEK9, TUBB6, TUBA1B, TUBB3, TUBA1C, GTSE1	1020	36	16792	3.658388	1	0.462934	9.18553
GOTERM_BP_DIRECT	GO:0030216~kerat	12	0.981997	0.005936	NOTCH1, CASP3, S100A7, SPRR2D,	1020	76	16792	2.599381	1	0.492531	10.3057

	inocyte differentiation				KRT16, SPRR1B, TGM1, ST14, IVL, INTU, SCEL, ADAM9						
GOTERM_BP_DIRECT	GO:0050796~regulation of insulin secretion	11	0.900164	0.006857	ICA1, SLC2A1, SYT9, RAPGEF4, GNAS, RAPGEF3, ITPR3, ABCC8, CACNA1D, KCNJ11, ITPR1	1020	67	16792	2.702839	1	0.531682	11.81127
GOTERM_BP_DIRECT	GO:0007411~axon guidance	19	1.554828	0.007904	ZNF280D, B4GAT1, WNT3A, EFNA2, EFNA3, NECTIN1, SMAD4, DPYSL5,	1020	159	16792	1.967246	1	0.571536	13.49409

					NTN4, ARHGAP35, EVL, L1CAM, TTC8, GLI3, GATA3, SPTBN2, ANOS1, SIAH2, TUBB3							
GOTERM_BP_DIRECT	GO:0031145~anaphase-promoting complex-dependent catabol i	12	0.981997	0.007947	CCNB1, CDK1, PLK1, PSMB2, AURKA, CDC20, PSME4, AURKB, UBE2C, PSMA7, PSMD7, UBE2S	1020	79	16792	2.50067	1	0.562369	13.56294

	c process											
GOTERM_BP_DIRECT	GO:0032355~response to estradiol	13	1.06383	0.008778	RAMP2, DNMT3A, ASS1, TH, EZH2, ESR1, CST3, FGF10, MMP15, KCNJ11, GPI, CASP3, DNMT3B	1020	91	16792	2.351821	1	0.587842	14.87656
GOTERM_BP_DIRECT	GO:0051983~regulation of chromosome segregat	4	0.327332	0.009908	KIF2C, MKI67, BUB1, AURKB	1020	8	16792	8.231373	1	0.621834	16.63206

	ion											
GOTERM_BP_DIRECT	GO:0010046~response to mycotoxin	3	0.245499	0.010592	LCN2, BAK1, ASS1	1020	3	16792	16.46275	1	0.636138	17.67727
GOTERM_BP_DIRECT	GO:0000083~regulation of transcription involved in G1/S transiti	6	0.490998	0.010966	CCNE1, CDK1, CDC45, RRM2, ORC1, CDT1	1020	23	16792	4.294629	1	0.638902	18.24428

	on of mitotic cell cycle											
GOTERM_BP_DIRECT	GO:0031146~SCF-dependent proteasomal ubiquitin-dependent protein catabolic		6	0.490998	0.013175	BTRC, CCNF, FBXW4, FBXL5, FBXO4, FBXO36	1020	24	16792	4.115686	1	0.696658	21.51608

	process											
GOTERM_BP_DIRECT	GO:0070059~intrinsic apoptotic signaling pathway in response to endoplasmic reticulum stre	7	0.572831	0.0133	BAK1, CEBPB, XBP1, CHAC1, BCL2, TRIB3, ITPR1	1020	33	16792	3.492097	1	0.690706	21.69838

	ss											
GOTERM_BP_DIRECT	GO:0034080~CENP-A containing nucleosome assembly	8	0.654664	0.014066	CENPO, CENPN, CENPA, HJURP, MIS18A, CENPP, CENPW, CENPI	1020	43	16792	3.062836	1	0.701942	22.80109
GOTERM_BP_DIRECT	GO:0033314~mitotic DNA replication checkpoint	4	0.327332	0.014198	CLSPN, TICRR, RAD17, NAE1	1020	9	16792	7.316776	1	0.696429	22.99026

GOTERM_BP_DIRECT	GO:1904851~positive regulation of establishment of protein localization to telomere	4	0.327332	0.014198	CCT5, TCP1, DKC1, CCT6A	1020	9	16792	7.316776	1	0.696429	22.99026
GOTERM_BP_DIRECT	GO:0031110~regulatio	4	0.327332	0.014198	SKA3, STMN1, SKA1, CLASP2	1020	9	16792	7.316776	1	0.696429	22.99026

	n of microtubule polymerization or depolymerization											
GOTERM_BP_DIRECT	GO:1904668~positive regulation of ubiquitin prot	4	0.327332	0.014198	PLK1, CDC20, UBE2C, UBE2S	1020	9	16792	7.316776	1	0.696429	22.99026

	ein ligase activity											
GOTERM_BP_DIRECT	GO:0008283~cell proliferation	34	2.782324	0.014983	STIL, ERBB4, E2F8, AURKB, MCM10, PRDX1, SRC, FAM83D, KIF2C, BAK1, FAM83B, DKC1, BCL2, BUB1, TGFA, RAPGEF3, NRDC, CDK1, PDXK, MKI67, DLGAP5,	1020	366	16792	1.529326	1	0.707278	24.10191

					TPX2, SMAD4, TNFSF9, CDC25A, OGFOD1, UHRF1, RASGRF1, KRT16, PLK1, CHRM1, TXN, LRP2, MELK							
GOTERM_BP_DIRECT	GO:0043627~response to estrogen	10	0.818331	0.016132	HSP90AA1, IL4R, OPRK1, GATA3, ESR1, SRD5A1, ABCC2, CD24, GAL, GLI3	1020	65	16792	2.53273	1	0.725478	25.7029
GOTERM_BP_DIRECT	GO:0016055~Wnt sign	20	1.636661	0.019136	FZD9, BTRC, WNT3A, TLE3, TLE1,	1020	187	16792	1.760721	1	0.77708	29.74039

	aling pathway				ARL6, CALCOCO1, CTNNBIP1, CSNK2A2, CCNE1, RSPO4, AMER1, NXN, KREMEN2, FBXW4, BRD7, VPS35, MCC, CSNK1G3, CD24							
GOTERM_BP_DIRECT	GO:0001701~in utero embryonic developm	20	1.636661	0.019136	STIL, TAPT1, UBE2A, WNT3A, MYO1E, SMAD4, GJB3, GLI3, CCNB1, GPI, NOTCH1,	1020	187	16792	1.760721	1	0.77708	29.74039

	ent				WDR19, MYO18B, CHD7, CCNB2, GATA3, BTF3, APBA2, TCTN1, FOSL1							
GOTERM_BP_DIRECT	GO:0045724~positive regulation of cilium assembly	4	0.327332	0.01938	BBS4, TAPT1, CEP120, ARHGAP35	1020	10	16792	6.585098	1	0.773838	30.05936
GOTERM_BP_DIRECT	GO:0060009~Serto	4	0.327332	0.01938	SDC1, CST3, DMRT1, HSD17B4	1020	10	16792	6.585098	1	0.773838	30.05936

	li cell development											
GOTERM_BP_DIRECT	GO:0072015~glomerular visceral epithelial cell development	4	0.327332	0.01938	MAGI2, MYO1E, NUP93, GLCCI1	1020	10	16792	6.585098	1	0.773838	30.05936
GOTERM_BP_DIRECT	GO:0030900~forebrain deve	8	0.654664	0.019981	STIL, NOTCH1, DYNC2H1, ARHGAP35, LRP2, CHRD, SRC,	1020	46	16792	2.863086	1	0.776811	30.83816

	lopment				DCLK1							
GOTERM_BP_DIRECT	GO:0006564~L-serine biosynthetic process	3	0.245499	0.020332	SHMT2, PSAT1, PSPH	1020	4	16792	12.34706	1	0.775499	31.28926
GOTERM_BP_DIRECT	GO:0061512~protein localization to cilium	5	0.409165	0.020822	BBS1, BBS4, ARL6, EHD1, WDR35	1020	18	16792	4.572985	1	0.776495	31.9134
GOTERM_BP_DIRECT	GO:0035264~m	11	0.900164	0.022531	STIL, XPA, TNS2, DHCR7,	1020	80	16792	2.263627	1	0.796001	34.05265

	ulticellular organism growth				SPTBN2, APBA2, GNAS, EN1, TTC8, SLITRK6, CTC1							
GOTERM_BP_DIRECT	GO:0019886~antigen processing and presentation of exogenous peptide anti	12	0.981997	0.02328	KIF23, KIF2C, KIF4B, DYNC1LI2, KIF4A, DYNC2LI1, KIF18A, SPTBN2, DYNC2H1, RACGAP1, DCTN4, CTSV	1020	92	16792	2.147315	1	0.800155	34.96906

	gen via MHC class II											
GOTERM_BP_DIRECT	GO:0007049~cell cycle	22	1.800327	0.023366	E2F2, APEX2, FOXM1, SUV39H1, AURKA, CDC20, AURKB, SRC, SUV39H2, SPAG8, UHRF1, HJURP, USP39, BRD7, CABLES2, CAMK1, RBM38, SIAH2, CHAF1B, CCNDBP1, RAD17,	1020	217	16792	1.669034	1	0.794959	35.07372

					CREBL2							
GOTERM_BP_DIRECT	GO:0000122~negative regulation of transcription from RNA polymerase II promoter	58	4.746318	0.023523	LMO1, IMPACT, E2F8, EZH2, CBX2, AURKB, ZNF345, TCEAL1, GLI3, CBX7, N4BP2L2, CRY2, PCBP3, GATA3, ELP2, BEND3, ZNF280D, RCOR3, SOX11, ESR1, DMRT1, TLE1, UBE2I, ASCL2, UHRF1,	1020	720	16792	1.326166	1	0.790842	35.26452

					BTG2,<br>IGBP1,<br>TXN,<br>TFAP2C,<br>TSHZ1,<br>FOXM1,<br>TRIB3,<br>ZBTB14,<br>ARHGAP35,<br>XBP1,<br>OVOL1,<br>NEDD4L,<br>BHLHE40,<br>DNMT3B,<br>TCF3,<br>KLF5,<br>DNMT3A,<br>SMAD4,<br>SUV39H1,<br>EN1,<br>SMYD2,<br>SNAI1,<br>SUV39H2,<br>SALL2,<br>NOTCH1,<br>ZNF217,							

					PLK1, MZF1, ZBTB4, PHF21A, LRP8, RERE, HDAC7							
GOTERM_BP_DIRECT	GO:0031424~keratinization	8	0.654664	0.024774	SPRR2D, KRT16, CASP14, SPRR1B, TGM1, CDH3, IVL, ABCA12	1020	48	16792	2.743791	1	0.801663	36.7624
GOTERM_BP_DIRECT	GO:1903364~positive regulation of cellular prot	4	0.327332	0.025466	AMER1, PTTG1IP, FBXL5, VPS35	1020	11	16792	5.986453	1	0.804614	37.57753

	ein catabolic process											
GOTERM_BP_DIRECT	GO:0006605~protein targeting	7	0.572831	0.028838	YWHAZ, HOMER3, TRAK1, HPS4, YWHAQ, GIPC1, KIF13B	1020	39	16792	2.954852	1	0.837728	41.40731
GOTERM_BP_DIRECT	GO:1902749~regulation of cell cycle G2/M phase	3	0.245499	0.032533	PLK1, NEK10, GTSE1	1020	5	16792	9.877647	1	0.867172	45.34923

	transition											
GOTERM_BP_DIRECT	GO:0030311~poly-N-acetyllactosamine biosynthetic process	3	0.245499	0.032533	B4GAT1, B3GNT4, B3GNT3	1020	5	16792	9.877647	1	0.867172	45.34923
GOTERM_BP_DIRECT	GO:0036124~histone H3-K9 trimethy	3	0.245499	0.032533	BEND3, SUV39H1, SUV39H2	1020	5	16792	9.877647	1	0.867172	45.34923

	lation											
GOTERM_BP_DIRECT	GO:1901985~positive regulation of protein acetylation	3	0.245499	0.032533	XBP1, CAMK1, RAPGEF3	1020	5	16792	9.877647	1	0.867172	45.34923
GOTERM_BP_DIRECT	GO:0051902~negative regulation of mito	3	0.245499	0.032533	FZD9, BCL2, SRC	1020	5	16792	9.877647	1	0.867172	45.34923

	chondrial depolarization											
GOTERM_BP_DIRECT	GO:0018101~protein citrullination	3	0.245499	0.032533	PADI3, PADI2, PADI1	1020	5	16792	9.877647	1	0.867172	45.34923
GOTERM_BP_DIRECT	GO:0002934~desmosome organization	3	0.245499	0.032533	NECTIN1, PERP, GRHL1	1020	5	16792	9.877647	1	0.867172	45.34923
GOTERM_BP_DIRECT	GO:00096	11	0.9001	0.032739	DNMT3A, CDK1, XPA,	1020	85	16792	2.130473	1	0.864128	45.5612

	36~response to toxic substance		64		SDC1, BCL2, CST3, EPHX2, CDH1, HTR1D, DNMT3B, RAD51							
GOTERM_BP_DIRECT	GO:0042542~response to hydrogen peroxide	8	0.654664	0.033349	BAK1, CASP3, SDC1, BCL2, PRDX3, FOSL1, SRC, ADAM9	1020	51	16792	2.582391	1	0.864505	46.1852
GOTERM_BP_DIRECT	GO:2000378~negative regulati	5	0.409165	0.035276	G6PD, BECN1, BCL2, SIRT3, MYCN	1020	21	16792	3.919701	1	0.875138	48.11168

	on of reactive oxygen species metabolic process											
GOTERM_BP_DIRECT	GO:1900182~positive regulation of protein localiza	5	0.409165	0.035276	CDK1, TRIM8, PLK1, SRC, GTSE1	1020	21	16792	3.919701	1	0.875138	48.11168

	tion to nucleus											
GOTERM_BP_DIRECT	GO:0051437~positive regulation of ubiquitin-protein ligase activity involved in regu	10	0.818331	0.040041	CCNB1, CDK1, PLK1, BTRC, PSMB2, CDC20, PSME4, UBE2C, PSMA7, PSMD7	1020	76	16792	2.166151	1	0.9025	52.59868

	lation of mitotic cell cycle transition											
GOTERM_BP_DIRECT	GO:0043161~proteasome-mediated ubiquitin-dependent protein	20	1.636661	0.040078	CDK1, UBE2A, BTRC, ABTB2, CDC20, UBE2C, ZNRF1, PSMA7, KCTD5, RNF222, GTSE1, AMER1, UBXN2A, PSMB2, NEDD4L,	1020	203	16792	1.621945	1	0.898927	52.63223

	catabolic process				SMURF1, PSME4, SIAH2, PSMD7, SPOP							
GOTERM_BP_DIRECT	GO:1900025~negative regulation of substrate adhesion-dependent cell spreading	4	0.327332	0.040331	CORO1C, RCC2, ACTN4, AP1AR	1020	13	16792	5.06546	1	0.896633	52.8597

GOTERM_BP_DIRECT	GO:0098609~cell-cell adhesion	25	2.045827	0.040746	VASN, F11R, YWHAZ, S100P, STK38, LAD1, DIAPH3, S100A11, PFKP, ARFIP2, SNX1, GIPC1, ANLN, EIF2A, TAGLN2, PRDX1, PKM, PLCB3, CCNB2, LRRC59, SPTBN2, DNAJB1, PERP, EHD1, TES	1020	271	16792	1.518703	1	0.895329	53.23104
GOTERM_BP_DIRECT	GO:00708	6	0.4909	0.041947	F11R, RAMP2,	1020	32	16792	3.086765	1	0.898591	54.2895

	30~bicellular tight junction assembly		98		ACTN4, CLDN3, MARVELD3, FRMPD2							
GOTERM_BP_DIRECT	GO:0071168~protein localization to chromatin	3	0.245499	0.04686	PLK1, EZH2, ESR1	1020	6	16792	8.231373	1	0.91984	58.38719
GOTERM_BP_DIRECT	GO:0046578~regul	3	0.245499	0.04686	RASGRF1, FOXM1, SH2B2	1020	6	16792	8.231373	1	0.91984	58.38719

	ation of Ras protein signal transduction											
GOTERM_BP_DIRECT	GO:0031536~positive regulation of exit from mitosis	3	0.245499	0.04686	BIRC5, UBE2C, CDCA5	1020	6	16792	8.231373	1	0.91984	58.38719
GOTERM_BP_DIRECT	GO:2000347~p	3	0.245499	0.04686	HPN, XBP1, WNT3A	1020	6	16792	8.231373	1	0.91984	58.38719

	ositive regulation of hepatocyte proliferation											
GOTERM_BP_DIRECT	GO:0023052~signaling	3	0.245499	0.04686	DLGAP3, DLGAP5, DLGAP4	1020	6	16792	8.231373	1	0.91984	58.38719
GOTERM_BP_DIRECT	GO:0098532~histone H3-K27 trim	3	0.245499	0.04686	BEND3, EZH2, CHD5	1020	6	16792	8.231373	1	0.91984	58.38719

	ethylation											
GOTERM_BP_DIRECT	GO:0051297~centrosome organization	6	0.490998	0.047063	BBS4, CEP120, PLK1, HAUS8, PCM1, CHD3	1020	33	16792	2.993226	1	0.917628	58.54857
GOTERM_BP_DIRECT	GO:0051439~regulation of ubiquitin-protein ligase acti	5	0.409165	0.047456	CCNB1, CDK1, PLK1, CDC20, UBE2C	1020	23	16792	3.578858	1	0.916282	58.85996

	vity involved in mitotic cell cycle											
GOTERM_BP_DIRECT	GO:0007368~determination of left/right symmetry	8	0.654664	0.047565	STIL, NOTCH1, DYNC2LI1, DYNC2H1, FGF10, PCSK6, ARL6, DNAH5	1020	55	16792	2.394581	1	0.913668	58.94557
GOTERM_BP_DIRECT	GO:0006879~cellular	7	0.572831	0.048441	LCN2, TTC7A, CYBRD1, NDFIP1, SMAD4,	1020	44	16792	2.619073	1	0.914533	59.62973

	iron ion homeostasis				SLC46A1, ABCG2							
GOTERM_BP_DIRECT	GO:0007077~mitotic nuclear envelope disassembly	7	0.572831	0.048441	CCNB1, CDK1, CCNB2, PLK1, RAE1, NUP93, NEK9	1020	44	16792	2.619073	1	0.914533	59.62973
GOTERM_BP_DIRECT	GO:0060065~uterus development	4	0.327332	0.049077	GATA3, SMAD4, ESR1, SRC	1020	14	16792	4.703641	1	0.91432	60.11982
GOTERM_B	GO:0	5	0.4	0.054	BBS4,	1020	24	16792	3.429	1	0.932006	63.9

P_DIRECT	046907~intracellular transport		09165	312	BBS5, GNAS, SPIRE2, SDCBP2				739			4618
GOTERM_BP_DIRECT	GO:0001890~placenta development	6	0.490998	0.058355	ASCL2, DLX3, E2F8, CCNF, GJB3, PHLDA2	1020	35	16792	2.822185	1	0.942407	66.66008
GOTERM_BP_DIRECT	GO:0031648~protein destabilization	6	0.490998	0.058355	XBP1, PLK1, BTRC, FBXO4, CHFR, SRC	1020	35	16792	2.822185	1	0.942407	66.66008
GOTERM_B	GO:0	4	0.3	0.058	GINS1,	1020	15	16792	4.390	1	0.941019	66.8

P_DIRECT	006271~DNA strand elongation involved in DNA replication		27332	662	GINS3, GINS4, PRIM2				065			5819
GOTERM_BP_DIRECT	GO:0010633~negative regulation of epitheli	4	0.327332	0.058662	CORO1C, MARVELD3, MCC, EVL	1020	15	16792	4.390065	1	0.941019	66.85819

	al cell migration											
GOTERM_BP_DIRECT	GO:1904874~positive regulation of telomerase RNA localization to Cajal body	4	0.327332	0.058662	CCT5, TCP1, DKC1, CCT6A	1020	15	16792	4.390065	1	0.941019	66.85819
GOTERM_B	GO:0	10	0.8	0.060	KIF23,	1020	82	16792	2.007	1	0.942676	67.7

P_DIRECT	006890~retrograde vesicle-mediated transport, Golgi to ER		18331	021	KDELR3, COG4, KIF2C, KIF4B, TMED7, KIF4A, ATP9B, KIF18A, RACGAP1				652			2167
GOTERM_BP_DIRECT	GO:0042035~regulation of cytokine biosynthetic	3	0.245499	0.063011	GATA3, IGF2BP1, IGF2BP2	1020	7	16792	7.055462	1	0.948495	69.54649

	process											
GOTERM_BP_DIRECT	GO:0031100~organ regeneration	7	0.572831	0.063366	PKM, CDK1, BAK1, NOTCH1, MKI67, PTPRU, CCNA2	1020	47	16792	2.451898	1	0.947351	69.75669
GOTERM_BP_DIRECT	GO:0010043~response to zinc ion	6	0.490998	0.064532	S100A8, ASS1, TH, GGH, SLC30A3, ABCC8	1020	36	16792	2.743791	1	0.94824	70.43677
GOTERM_BP_DIRECT	GO:0009409~response to cold	6	0.490998	0.064532	SLC27A1, ZNF516, HSP90AA1, CIRBP, PCSK1N, VGF	1020	36	16792	2.743791	1	0.94824	70.43677
GOTERM_BP_DIRECT	GO:00714	8	0.6546	0.065145	KLF5, CCNB1,	1020	59	16792	2.232237	1	0.94776	70.78865

	07~cellular response to organic cyclic compound		64		P2RY6, CASP3, RGS20, CEBPB, RAE1, BTRC							
GOTERM_BP_DIRECT	GO:0051895~negative regulation of focal adhesion asse	4	0.327332	0.069053	CORO1C, RCC2, CLASP2, SRC	1020	16	16792	4.115686	1	0.95476	72.94074

	mbly											
GOTERM_BP_DIRECT	GO:2000179~positive regulation of neural precursor cell proliferation	4	0.327332	0.069053	FZD9, CDON, WNT3A, ADGRG1	1020	16	16792	4.115686	1	0.95476	72.94074
GOTERM_BP_DIRECT	GO:0050768~negative regu	4	0.327332	0.069053	NOTCH1, WNT3A, DLL3, PCM1	1020	16	16792	4.115686	1	0.95476	72.94074

	lation of neurogenesis											
GOTERM_BP_DIRECT	GO:0042755~eating behavior	5	0.409165	0.069533	CCK, OPRK1, TH, BBS12, NMU	1020	26	16792	3.165913	1	0.954001	73.19464
GOTERM_BP_DIRECT	GO:0006836~neurotransmitter transport	5	0.409165	0.069533	ICA1, CPLX1, SLC6A11, SLC6A17, SLC6A19	1020	26	16792	3.165913	1	0.954001	73.19464
GOTERM_BP_DIRECT	GO:0016266~O	8	0.654664	0.070078	GALNT3, MUC21, B3GNT4,	1020	60	16792	2.195033	1	0.953386	73.48024

	-glycan processing				B3GNT7, B3GNT3, MUC5B, B4GALT5, MUC16						
GOTERM_BP_DIRECT	GO:0014070~response to organic cyclic compound	7	0.572831	0.074661	CDK1, BAK1, G6PD, BTG2, MKI67, PSMB2, ABCD3	1020	49	16792	2.351821	1	0.960617	75.76902
GOTERM_BP_DIRECT	GO:0001889~liver development	9	0.736498	0.078912	PKM, NOTCH1, ASS1, XBP1, CEBPG, PTCD2, QDPR, SRD5A1, LSR	1020	74	16792	2.002226	1	0.966124	77.72378

GOTERM_BP_DIRECT	GO:2000811~negative regulation of anoikis	4	0.327332	0.080208	NOTCH1, BCL2, TLE1, SRC	1020	17	16792	3.873587	1	0.966695	78.2895
GOTERM_BP_DIRECT	GO:1904871~positive regulation of protein localization to	3	0.245499	0.080712	CCT5, TCP1, CCT6A	1020	8	16792	6.173529	1	0.96609	78.50571

	Cajal body											
GOTERM_BP_DIRECT	GO:0000076~DNA replication checkpoint	3	0.245499	0.080712	CDC45, RAD17, CDT1	1020	8	16792	6.173529	1	0.96609	78.50571
GOTERM_BP_DIRECT	GO:0043497~regulation of protein heterodimerization	3	0.245499	0.080712	BAK1, CDON, BCL2	1020	8	16792	6.173529	1	0.96609	78.50571

	activity											
GOTERM_BP_DIRECT	GO:0060236~regulation of mitotic spindle organization	3	0.245499	0.080712	BORA, TPX2, PARP3	1020	8	16792	6.173529	1	0.96609	78.50571
GOTERM_BP_DIRECT	GO:0033146~regulation of intracellular estr	3	0.245499	0.080712	UFSP2, CARM1, SRC	1020	8	16792	6.173529	1	0.96609	78.50571

	ogen receptor signaling pathway											
GOTERM_BP_DIRECT	GO:0045056~transcytosis	3	0.245499	0.080712	RAB17, VPS35, SRC	1020	8	16792	6.173529	1	0.96609	78.50571
GOTERM_BP_DIRECT	GO:0050773~regulation of dendrite development	3	0.245499	0.080712	RAB17, CDC20, CAMK1D	1020	8	16792	6.173529	1	0.96609	78.50571
GOTERM_B	GO:0	15	1.2	0.081	CDK1,	1020	153	16792	1.613	1	0.965995	78.8

P_DIRECT	042787~protein ubiquitination involved in ubiquitin-dependent protein catabolic process		27496	557	ABTB2, CDC20, GAN, AURKA, AURKB, UBE2C, CCNB1, UHRF1, PLK1, NEDD4L, SMURF1, SIAH2, SPOP, KLHL32				995			6376
GOTERM_BP_DIRECT	GO:000079~r	6	0.490998	0.085157	CCNE2, GTPBP4, PKMYT1,	1020	39	16792	2.53273	1	0.969702	80.327

	egulation of cyclin-dependent protein serine/threonine kinase activity				CCNG1, CCNA2, CDC25A							
GOTERM_BP_DIRECT	GO:0016337~single organismal cell	11	0.900164	0.085867	MPZL2, PKP1, COL13A1, NECTIN1, CYFIP2, CDH1, PTPRU, CD24, CDH3, SRC,	1020	101	16792	1.792972	1	0.969411	80.60428

	-cell adhesion				NPHP1							
GOTERM_BP_DIRECT	GO:0045931~positive regulation of mitotic cell cycle	5	0.409165	0.0867	CCNB1, CDK1, FGF10, BIRC5, CDC25B	1020	28	16792	2.939776	1	0.969283	80.92452
GOTERM_BP_DIRECT	GO:0009314~response to radiatio	5	0.409165	0.0867	F11R, OPRK1, BCL2, TXN, CDC25A	1020	28	16792	2.939776	1	0.969283	80.92452

	n											
GOTERM_BP_DIRECT	GO:0018146~keratan sulfate biosynthetic process	5	0.409165	0.0867	B4GAT1, B3GNT4, B3GNT7, B3GNT3, B4GALT5	1020	28	16792	2.939776	1	0.969283	80.92452
GOTERM_BP_DIRECT	GO:0007219~Notch signaling pathway	12	0.981997	0.089621	NOTCH1, CHAC1, DTX2, DNER, DTX3, APH1B, DLL3, MAML3, PERP, MESP1, MESP2, ANXA4	1020	115	16792	1.717852	1	0.971728	82.00864
GOTERM_B	GO:0	8	0.6	0.091	AMER1,	1020	64	16792	2.057	1	0.973322	82.8

P_DIRECT	031398~positive regulation of protein ubiquitination		54664	974	FANCI, NDFIP1, PTTG1IP, TBC1D7, FBXO4, CHFR, TSPYL5				843			3973
GOTERM_BP_DIRECT	GO:0030705~cytoskeleton-dependent intracellula	4	0.327332	0.092086	TUBB, TUBA1B, TUBA1C, KIF13B	1020	18	16792	3.658388	1	0.972377	82.87807

	r transport											
GOTERM_BP_DIRECT	GO:0046655~folic acid metabolic process	4	0.327332	0.092086	MTHFD2, SHMT2, SLC46A1, MTHFD1L	1020	18	16792	3.658388	1	0.972377	82.87807
GOTERM_BP_DIRECT	GO:0071539~protein localization to centrosome	4	0.327332	0.092086	STIL, BBS4, AURKA, PCM1	1020	18	16792	3.658388	1	0.972377	82.87807

GOTERM_BP_DIRECT	GO:0045184~establishment of protein localization	6	0.490998	0.092715	CORO1C, RCC2, PLK1, DLG4, MCC, CEP55	1020	40	16792	2.469412	1	0.972013	83.09373
GOTERM_BP_DIRECT	GO:0006633~fatty acid biosynthetic process	7	0.572831	0.093603	PRKAG3, ELOVL1, XBP1, PRKAB1, ABCD3, FADS6, HSD17B8	1020	52	16792	2.216139	1	0.971944	83.39351
GOTERM_BP_DIRECT	GO:0042149~cellu	5	0.409165	0.095981	BECN1, XBP1, IMPACT, BCL2,	1020	29	16792	2.838404	1	0.973496	84.17162

	lar response to glucose starvation				SLC2A1							
GOTERM_BP_DIRECT	GO:0060068~vagina development	3	0.245499	0.099716	TYRO3, BAK1, ESR1	1020	9	16792	5.487582	1	0.976247	85.3245
GOTERM_BP_DIRECT	GO:0010838~positive regulation of	3	0.245499	0.099716	TGM1, FGF10, CDH3	1020	9	16792	5.487582	1	0.976247	85.3245

	keratinocyte proliferation											
GOTERM_BP_DIRECT	GO:0015886~heme transport	3	0.245499	0.099716	SLC46A1, FLVCR2, ABCG2	1020	9	16792	5.487582	1	0.976247	85.3245
GOTERM_BP_DIRECT	GO:0008608~attachment of spindle microtubules to	3	0.245499	0.099716	SGO1, NDC80, AURKB	1020	9	16792	5.487582	1	0.976247	85.3245

	kinetochore											
GOTERM_BP_DIRECT	GO:0000075~cell cycle checkpoint	3	0.245499	0.099716	CCNE2, TICRR, CCNG2	1020	9	16792	5.487582	1	0.976247	85.3245
GOTERM_BP_DIRECT	GO:0042754~negative regulation of circadian rhythm	3	0.245499	0.099716	CRY2, SUV39H1, SUV39H2	1020	9	16792	5.487582	1	0.976247	85.3245

c. KEGG

Category	Term	Count	%	PValue	Genes	List Total	Pop Hits	Pop Total	Fold Enrichment	Bonferroni	Benjamini	FDR
KEGG_PATHWAY	hsa04110:Cell cycle	24	1.963993	4.53E-07	E2F2, CDK1, YWHAZ, CCNH, DBF4, SMAD4, PKMYT1, TTK, CDC20, MCM4, CDC25A, CDC25B, MCM6, CCNB1, CCNE2, CCNE1, CDC45, CCNB2, PLK1, BUB1, YWHAQ,	397	124	6879	3.353701	1.19E-04	1.19E-04	5.93E-04

					ORC6, ORC1, CCNA2							
KEGG_PATHWAY	hsa04114:Oocyte meiosis	18	1.472995	1.82E-04	CDK1, YWHAZ, CPEB2, BTRC, PKMYT1, AURKA, CDC20, ITPR3, ITPR1, CCNB1, CCNE2, CCNE1, CCNB2, SGO1, PLK1, BUB1, YWHAQ, CALML5	397	111	6879	2.809858	0.046474	0.023513	0.237419
KEGG_PATHWAY	hsa04970:Salivary secre	13	1.06383	0.003553	CAMP, CST3, ITPR3, ITPR1, ATP2B2,	397	86	6879	2.619267	0.606486	0.267198	4.551634

	tion				KCNN4, PLCB3, CST5, GNAS, TRPV6, CALML5, VAMP2, MUC5B							
KEGG_PATHWAY	hsa04530:Tight junction	13	1.06383	0.003914	ACTB, F11R, CLDN7, CLDN9, ACTN4, CLDN4, CLDN3, MYH14, MYL12A, PPP2R2C, CLDN14, SRC, CLDN23	397	87	6879	2.58916	0.642075	0.226522	5.002497
KEGG_PATHWAY	hsa04115:p53 signaling	11	0.900164	0.004632	CCNB1, CCNE2, CCNE1, CDK1, CASP3,	397	67	6879	2.844806	0.703713	0.215952	5.895077

	pathway				CCNB2, RRM2, PERP, CCNG1, CCNG2, GTSE1							
KEGG_PATHWAY	hsa04670:Leukocyte transendothelial migration	14	1.145663	0.014492	ACTB, CLDN7, F11R, CLDN9, MYL5, CLDN4, ACTN4, CLDN3, ARHGAP35, MYL12A, CLDN23, CLDN14, RAPGEF4, RAPGEF3	397	115	6879	2.109429	0.978175	0.47136	17.39009
KEGG_PATHWAY	hsa04914:Progesterone-mediated	11	0.900164	0.027066	CCNB1, CDK1, HSP90AA1, CCNB2, CPEB2, PLK1,	397	87	6879	2.190828	0.999245	0.641928	30.16911

	oocyte maturation				BUB1, PKMYT1, CCNA2, CDC25A, CDC25B							
KEGG_PATHWAY	hsa04540:Gap junction	11	0.900164	0.029036	CDK1, TUBB, PLCB3, TUBB6, GNAS, ITPR3, TUBA1B, SRC, ITPR1, TUBB3, TUBA1C	397	88	6879	2.165932	0.999556	0.619015	31.99624
KEGG_PATHWAY	hsa03460:Fanconi anemia pathway	8	0.654664	0.031031	POLK, FAN1, POLI, FANCI, POLN, FANCA, UBE2T, RAD51	397	53	6879	2.615465	0.999741	0.600545	33.80243
KEGG_PATHWAY	hsa04925:A	10	0.8183	0.042199	AGTR1, PLCB3,	397	81	6879	2.139192	0.999988	0.67684	43.12035

	ldosterone synthesis and secretion		31		CYP21A2, CAMK1, GNAS, CALML5, ITPR3, CACNA1D, ITPR1, CAMK1D							
KEGG_PATHWAY	hsa00750:Vitamin B6 metabolism	3	0.245499	0.042486	PDXK, PHOSPHO2, PSAT1	397	6	6879	8.663728	0.999989	0.644444	43.34358
KEGG_PATHWAY	hsa04520:Adherens junction	9	0.736498	0.050137	ACTB, CSNK2A2, ACTN4, NECTIN1, SMAD4, CDH1, NECTIN4, SNAI1, SRC	397	71	6879	2.196438	0.999999	0.674712	48.98982
KEGG_PATHWAY	hsa01230:Biosyn	9	0.736498	0.053707	PKM, SHMT2, BCAT2,	397	72	6879	2.165932	0.999999	0.671284	51.44306

	thesis of amino acids				ASS1, PFKP, PSAT1, PSPH, PGK1, GPT2						
KEGG_PATHWAY	hsa04911:Insulin secretion	10	0.818331	0.054612	KCNN4, PLCB3, SLC2A1, RAPGEF4, GNAS, VAMP2, ITPR3, ABCC8, CACNA1D, KCNJ11	397	85	6879	2.038524	1	0.650411	52.04723
KEGG_PATHWAY	hsa00601:Glycosphingolipid biosynthesis - lacto	5	0.409165	0.05925	B4GAT1, B3GNT4, FUT6, FUT3, B3GNT3	397	26	6879	3.332203	1	0.655902	55.03617

	and neolacto series										
KEGG_PATHWAY	hsa05130:Pathogenic Escherichia coli infection	7	0.572831	0.07093	ACTB, TUBB, TUBB6, CDH1, TUBA1B, TUBB3, TUBA1C	397	51	6879	2.378278	1	0.700225	61.81788
KEGG_PATHWAY	hsa00310:Lysine degradation	7	0.572831	0.076493	AADAT, PLOD1, SUV39H1, ACAT2, COLGALT1, PHYKPL, SUV39H2	397	52	6879	2.332542	1	0.70666	64.70415
KEGG_PATHWAY	hsa04261:Adrenergic signa	13	1.06383	0.099323	TPM4, TPM3, TNNT2, AGTR1, ATP2B2,	397	138	6879	1.632297	1	0.781864	74.56356

	ling in cardiomyocytes				PLCB3, BCL2, GNAS, RAPGEF4, CALML5, RAPGEF3, CACNA1D, PPP2R2C						
KEGG_PATHWAY	hsa04710:Circadian rhythm	5	0.409165	0.099951	PRKAG3, CRY2, BTRC, PRKAB1, BHLHE40	397	31	6879	2.794751	1	0.765924	74.79452
