# Supplementary material for: LncRNA TINCR favors tumorigenesis via STAT3–TINCR–EGFR-feedback loop by recruiting DNMT1 and acting as a competing endogenous RNA in human breast cancer
Source: Cell Death Dis. 2021 Jan 14;12(1):83. doi: 10.1038/s41419-020-03188-0 (PMC7809450; doi:10.1038/s41419-020-03188-0)
Supplement: Supplementary file 11 — Supplementary Table S4 [file 41419_2020_3188_MOESM11_ESM.pdf]

a.EGFR

sample	log10	EGFR score
1319425	4.235336	8
1331476	4.188858	2
1422760	4.160707	8
1006332	4.106602	12
1113530	4.01452	3
1101010	4.012318	12
1411157	3.752647	6
1114897	3.712677	12
1402764	3.619948	4
1304866	3.609275	9
1334997	3.575331	1
1305400	3.418161	2
1111801	3.375327	8
1222763	3.229772	2
1413331	3.22791	6
1231366	3.147992	4
1010941	3.106809	6
1319857	3.100735	1
1331865	3.080815	6
1231086	3.053581	12
1229029	2.984331	0
1100998	2.964912	4
1224273	2.944464	2
1114912	2.944085	4
1414861	2.940908	6
1319895	2.909496	6
1014369	2.812029	12
1322979	2.715006	12
1302137	2.714629	0
1305920	2.696829	8
1324261	2.680333	0
1221498	2.678212	0
1221872	2.665233	8
1333208	2.662272	1
1321489	2.654116	8
1317374	2.649349	0
1324120	2.627135	0
1330963	2.623596	0
1222512	2.556238	6
1005573	2.54667	3

1010193	2.528288	12
1104421	2.375029	12
1222712	2.370355	3
1417455	2.3661	6
1223843	2.36087	0
1302537	2.344287	2
1228588	2.342565	0
1228435	2.342565	4
1312297	2.320961	2
1225842	2.31667	4
1221806	2.300805	4
1223730	2.296636	0
1221524	2.288681	3
1318194	2.260621	0
1309689	2.260546	0
1309275	2.258356	9
1302604	2.25264	1
1414094	2.246231	4
1229998	2.228796	0
1228142	2.207883	4
1224951	2.204038	2
1317173	2.172913	0
1334965	2.168213	0
1335910	2.160279	2
1316236	2.125018	0
1104967	2.114797	4
1005270	2.106705	6
1319091	2.045553	0
1108722	2.026182	4
1310933	1.990687	1
1419416	1.968707	0
1318108	1.943468	3
1403387	1.934773	6
1310072	1.934773	2
1103528	1.918897	0
1005339	1.918398	9
1223850	1.888242	1
1310062	1.883188	2
1303779	1.856002	0
1223558	1.826592	2
1317357	1.793502	0
1308315	1.758147	6
1304437	1.73311	0

1221890	1.730224	8
1013279	1.711659	2
1304713	1.702653	2
1305566	1.681781	0
1006634	1.669088	2
1227317	1.624651	0
1303717	1.576485	0
1223842	1.560643	2
1406585	1.529653	2
1405667	1.529653	9
1333641	1.508583	0
1324768	1.456733	0
1223846	1.426417	0
1317784	1.403181	4
1026118	1.398392	2
1222885	1.353575	8
1006235	1.348889	3
1111087	1.286628	6
1112341	1.259024	6
1325379	1.189616	2
1013941	1.188413	6
1100942	1.16274	0
1012927	1.062311	6
1226813	1.056301	8
1221805	0.989328	2
1307793	0.98852	1
1401862	0.927407	0
1408382	0.747242	4
1305613	0.682654	6
1225541	0.619174	0
1104887	0.566315	0
1417729	0.474064	4
1413933	0.427118	0
1026143	0.345171	6
1322103	0.112424	0
1221887	-0.59892	0
1322864	-3.19005	0

b.TINCR-EGFR

		TINCR			
		High expression	Low expression	X^2	P
EGFR	High Score	24	14	3.8511	0.0497
	Low Score	36	46		

c.JAK2

sample	log10	JAK2 score
1318160	5.595403589	2
1224420	5.000413277	12
1312896	4.29591651	1
1111264	4.261224585	12
1319425	4.235335558	6
1101010	4.012317501	12
1321034	3.952746179	3
1319366	3.758115251	4
1223387	3.360331755	12
1413183	3.359757481	2
1106031	3.309891271	2
1305917	3.202670269	8
1331865	3.080815037	2
1224273	2.944464202	1
1221830	2.870013685	2
1314381	2.83915726	2
1222911	2.808282399	2
1336548	2.766424542	1
1323700	2.747425115	1
1221498	2.67821228	1
1221872	2.665233008	4
1333208	2.662271963	1
1112424	2.445763302	12
1224287	2.409028105	1
1310923	2.390770782	2
1104421	2.375028553	9
1222712	2.370355261	3
1228588	2.34256537	1

1228435	2.34256537	4
1221806	2.300804907	2
1221524	2.28868084	2
1420952	2.19749729	2
1104967	2.114797017	3
1108722	2.02618191	2
1310933	1.99068704	1
1419416	1.968706812	3
1403387	1.934773213	3
1223850	1.888242333	1
1310062	1.883188097	3
1317357	1.793501864	2
1221890	1.730224239	2
1013279	1.71165851	4
1406585	1.5296534	2
1405667	1.5296534	1
1026118	1.398392048	2
1222885	1.353574785	1
1111087	1.286627899	6
1112341	1.259024028	2
1232875	1.228348898	3
1226813	1.05630122	4
1408382	0.747241783	6
1026143	0.345171213	2
