# Supplementary material for: LncRNA TINCR favors tumorigenesis via STAT3–TINCR–EGFR-feedback loop by recruiting DNMT1 and acting as a competing endogenous RNA in human breast cancer
Source: Cell Death Dis. 2021 Jan 14;12(1):83. doi: 10.1038/s41419-020-03188-0 (PMC7809450; doi:10.1038/s41419-020-03188-0)
Supplement: Supplementary file 12 — Supplementary Table S5 [file 41419_2020_3188_MOESM12_ESM.pdf]

a. TargetScan

miRNA	Position in the UTR	seed match	context++ score	context++ score percentile	weighted context++ score	conserved branch length	Pct
Conserved sites							
hsa-miR-133a-3p.2	50-56	7mer-m8	-0.07	60	-0.07	3.39	0.57
hsa-miR-133b	50-56	7mer-m8	-0.07	60	-0.07	3.39	0.57
hsa-miR-875-5p	270-277	8mer	-0.2	91	-0.2	2.267	N/A
hsa-miR-7-5p	457-464	8mer	-0.08	80	-0.08	2.831	0.19
hsa-miR-138-5p	3765-3772	8mer	-0.12	69	-0.11	2.142	0.16
hsa-miR-222-3p	4161-4168	8mer	-0.14	76	-0.12	3.613	0.52
hsa-miR-221-3p	4161-4168	8mer	-0.13	75	-0.11	3.613	0.52
hsa-miR-152-3p	4166-4172	7mer-1A	-0.24	90	-0.2	4.132	0.55
hsa-miR-148b-3p	4166-4172	7mer-1A	-0.24	90	-0.2	4.132	0.55
hsa-miR-148a-3p	4166-4172	7mer-1A	-0.24	90	-0.2	4.132	0.55
hsa-miR-411-5p.2	4206-4213	8mer	-0.03	32	-0.03	2.613	N/A
hsa-miR-455-3p.2	4238-4245	8mer	-0.09	86	-0.08	1.846	<

							0.1
hsa-miR-137	4563-4570	8mer	-0.03	38	-0.03	2.254	<0.1
hsa-miR-302b-3p	4574-4580	7mer-m8	-0.14	87	-0.12	2.957	0.17
hsa-miR-302e	4574-4580	7mer-m8	-0.14	87	-0.12	2.957	0.17
hsa-miR-302a-3p	4574-4580	7mer-m8	-0.14	87	-0.12	2.957	0.17
hsa-miR-302d-3p	4574-4580	7mer-m8	-0.14	87	-0.12	2.957	0.17
hsa-miR-302c-3p.1	4574-4580	7mer-m8	-0.14	87	-0.12	2.957	0.17
hsa-miR-520d-3p	4574-4580	7mer-m8	-0.11	83	-0.09	2.957	0.17
hsa-miR-520a-3p	4574-4580	7mer-m8	-0.11	83	-0.09	2.957	0.17
hsa-miR-372-3p	4574-4580	7mer-m8	-0.11	82	-0.09	2.957	0.17
hsa-miR-520b	4574-4580	7mer-m8	-0.11	82	-0.09	2.957	0.17
hsa-miR-520c-3p	4574-4580	7mer-m8	-0.11	82	-0.09	2.957	0.17
hsa-miR-520e	4574-4580	7mer-m8	-0.1	82	-0.08	2.957	0.17
hsa-miR-373-3p	4574-4580	7mer-m8	-0.05	80	-0.04	2.957	0.1

							7
hsa-miR-106b-5p	4575-4581	7mer-m8	-0.05	82	-0.04	3.005	＜0.1
hsa-miR-20a-5p	4575-4581	7mer-m8	-0.05	81	-0.04	3.005	＜0.1
hsa-miR-93-5p	4575-4581	7mer-m8	-0.03	80	-0.03	3.005	＜0.1
hsa-miR-20b-5p	4575-4581	7mer-m8	-0.03	79	-0.03	3.005	＜0.1
hsa-miR-17-5p	4575-4581	7mer-m8	-0.03	78	-0.03	3.005	＜0.1
hsa-miR-106a-5p	4575-4581	7mer-m8	-0.04	78	-0.03	3.005	＜0.1
hsa-miR-519d-3p	4575-4581	7mer-m8	-0.03	78	-0.03	3.005	＜0.1
hsa-miR-526b-3p	4575-4581	7mer-m8	-0.02	62	-0.02	3.005	＜0.1
hsa-miR-137	5756-5763	8mer	-0.03	38	-0.03	2.464	＜0.1
hsa-miR-137	5784-5790	7mer-m8	-0.14	71	-0.12	4.91	0.81
hsa-miR-493-5p	5856-5862	7mer-1A	-0.08	80	-0.07	3.906	N/A
hsa-miR-144-3p	5894-5900	7mer-m8	-0.17	91	-0.15	4.7	0.91
hsa-miR-421	5897-5903	7mer-m8	-0.11	80	-0.1	3.185	N/A
Poorly conserved							

sites							
hsa-miR-4437	16-22	7mer-m8	-0.24	87	-0.24	0	N/A
hsa-miR-5088-5p	17-24	8mer	-0.46	99	-0.46	0	N/A
hsa-miR-3158-3p	18-24	7mer-1A	-0.14	84	-0.14	0	N/A
hsa-miR-4446-3p	18-24	7mer-1A	-0.14	76	-0.14	0.024	N/A
hsa-miR-2909	19-26	8mer	-0.27	96	-0.27	0	N/A
hsa-miR-1296-5p	19-25	7mer-1A	-0.17	72	-0.17	0.024	N/A
hsa-miR-1290	25-32	8mer	-0.03	54	-0.03	0.024	N/A
hsa-miR-3167	26-32	7mer-1A	-0.08	76	-0.08	0.221	N/A
hsa-miR-876-5p	26-32	7mer-1A	-0.01	30	-0.01	0.221	N/A
hsa-miR-378j	27-34	8mer	-0.19	91	-0.19	0.099	N/A
hsa-miR-6839-5p	27-34	8mer	-0.18	90	-0.18	0.099	N/A
hsa-miR-5706	28-34	7mer-m8	-0.15	89	-0.15	0	N/A
hsa-miR-4782-5p	28-34	7mer-m8	-0.13	86	-0.13	0	N/A
hsa-miR-5007-5p	32-38	7mer-m8	-0.13	83	-0.13	0	N/A
hsa-miR-503-3p	43-49	7mer-1A	-0.05	48	-0.05	0	N/A
hsa-miR-6861-5p	44-50	7mer-m8	-0.02	24	-0.02	0	N/A
hsa-miR-6742-3p	45-52	8mer	-0.07	77	-0.07	0	N/A
hsa-miR-6852-5p	46-52	7mer-1A	-0.06	66	-0.06	0.437	N/A
hsa-miR-939-3p	46-52	7mer-1A	-0.01	37	-0.01	0	N/A
hsa-miR-1343-3p	46-52	7mer-m8	-0.02	33	-0.02	0.335	N/A
hsa-miR-6783-3p	46-52	7mer-m8	-0.02	32	-0.02	0.335	N/A
hsa-miR-133a-3p.1	50-56	7mer-1A	-0.02	31	-0.02	3.39	0.68
hsa-miR-4474-3p	56-62	7mer-m8	-0.02	24	-0.02	0	N/A

hsa-miR-6893-3p	61-67	7mer-m8	-0.09	68	-0.09	0.145	N/A
hsa-miR-370-3p	61-67	7mer-m8	-0.05	67	-0.05	0.145	N/A
hsa-miR-492	64-70	7mer-m8	-0.09	53	-0.09	0.024	N/A
hsa-miR-4751	66-72	7mer-m8	-0.15	80	-0.15	0	N/A
hsa-miR-7154-3p	67-74	8mer	-0.12	83	-0.12	0	N/A
hsa-miR-766-5p	68-74	7mer-1A	-0.11	71	-0.11	0	N/A
hsa-miR-6894-5p	68-74	7mer-1A	-0.03	42	-0.03	0	N/A
hsa-miR-765	68-74	7mer-1A	-0.04	40	-0.04	0.099	N/A
hsa-miR-4802-5p	69-75	7mer-m8	-0.02	34	-0.02	0	N/A
hsa-miR-4534	70-76	7mer-m8	-0.02	38	-0.02	0	N/A
hsa-miR-8082	70-76	7mer-m8	-0.02	26	-0.02	0	N/A
hsa-miR-6740-5p	70-81	non-canonical	N/A	N/A	N/A	0	N/A
hsa-miR-6740-5p	70-81	non-canonical	N/A	N/A	N/A	0	N/A
hsa-miR-6728-5p	73-80	8mer	-0.09	76	-0.09	0	N/A
hsa-miR-5187-5p	73-79	7mer-1A	-0.12	70	-0.12	0	N/A
hsa-miR-6513-5p	74-80	7mer-m8	-0.02	52	-0.02	0	N/A
hsa-miR-769-3p	74-80	7mer-1A	-0.01	51	-0.01	0.099	N/A
hsa-miR-450b-3p	74-80	7mer-1A	-0.01	47	-0.01	0.099	N/A
hsa-miR-5089-5p	74-80	7mer-1A	-0.01	28	-0.01	0	N/A
hsa-miR-4425	75-82	8mer	-0.14	65	-0.14	0	N/A
hsa-miR-3147	76-82	7mer-1A	-0.07	73	-0.07	0	N/A
hsa-miR-4687-3p	79-86	8mer	-0.11	79	-0.11	0	N/A
hsa-miR-7974	80-86	7mer-1A	-0.12	64	-0.12	0	N/A

hsa-miR-889-5p	81-87	7mer-m8	-0.07	63	-0.07	0.085	N/A
hsa-miR-3622b-5p	84-90	7mer-m8	-0.07	56	-0.07	0	N/A
hsa-miR-4253	85-91	7mer-m8	-0.11	54	-0.11	0	N/A
hsa-miR-6862-5p	85-91	7mer-m8	-0.09	50	-0.09	0	N/A
hsa-miR-4767	88-94	7mer-1A	-0.3	87	-0.3	0	N/A
hsa-miR-610	95-101	7mer-m8	-0.09	54	-0.09	0.024	N/A
hsa-miR-193a-5p	104-110	7mer-1A	-0.1	73	-0.1	0.338	< 0.1
hsa-miR-6759-5p	106-112	7mer-m8	-0.12	79	-0.12	0	N/A
hsa-miR-6793-5p	106-112	7mer-1A	-0.06	52	-0.06	0.335	N/A
hsa-miR-6748-5p	106-112	7mer-1A	-0.06	38	-0.06	0.335	N/A
hsa-miR-134-3p	107-114	8mer	-0.24	94	-0.24	0.085	N/A
hsa-miR-7114-5p	108-114	7mer-1A	-0.07	64	-0.07	0	N/A
hsa-miR-622	111-117	7mer-m8	-0.06	66	-0.06	0.504	N/A
hsa-miR-548ap-3p	116-122	7mer-m8	-0.02	37	-0.02	0	N/A
hsa-miR-548t-3p	116-122	7mer-m8	-0.02	36	-0.02	0	N/A
hsa-miR-548aa	116-122	7mer-m8	-0.02	36	-0.02	0	N/A
hsa-miR-548aw	119-126	8mer	-0.03	73	-0.03	0	N/A
hsa-miR-548f-5p	119-125	7mer-1A	-0.01	35	-0.01	0	N/A
hsa-miR-548g-5p	119-125	7mer-1A	-0.01	35	-0.01	0	N/A
hsa-miR-548x-5p	119-125	7mer-1A	-0.01	35	-0.01	0	N/A
hsa-miR-548aj-5p	119-125	7mer-1A	-0.01	35	-0.01	0	N/A
hsa-miR-1468-3p	119-125	7mer-1A	-0.01	26	-0.01	0	N/A
hsa-miR-527	120-126	7mer-1A	-0.01	44	-0.01	0.024	N/A
hsa-miR-518a-5p	120-126	7mer-1A	-0.01	44	-0.01	0.024	N/A

hsa-miR-4684-3p	121-127	7mer-m8	-0.03	36	-0.03	0	N/A
hsa-miR-30b-5p	128-134	7mer-1A	-0.01	9	-0.01	0.122	<0.1
hsa-miR-30c-5p	128-134	7mer-1A	-0.01	9	-0.01	0.122	<0.1
hsa-miR-30a-5p	128-134	7mer-1A	-0.01	9	-0.01	0.122	<0.1
hsa-miR-30e-5p	128-134	7mer-1A	-0.01	9	-0.01	0.122	<0.1
hsa-miR-30d-5p	128-134	7mer-1A	-0.01	9	-0.01	0.122	<0.1
hsa-miR-8086	137-143	7mer-m8	-0.13	71	-0.13	0	N/A
hsa-miR-6069	139-145	7mer-1A	-0.06	52	-0.06	0	N/A
hsa-miR-4794	140-146	7mer-m8	-0.02	35	-0.02	0	N/A
hsa-miR-664a-5p	140-146	7mer-m8	-0.02	32	-0.02	0	N/A
hsa-miR-221-5p	142-148	7mer-1A	-0.19	90	-0.19	0.085	N/A
hsa-miR-8073	142-148	7mer-1A	-0.15	85	-0.15	0.085	N/A
hsa-miR-1343-3p	143-149	7mer-1A	-0.01	27	-0.01	0	N/A
hsa-miR-6783-3p	143-149	7mer-1A	-0.01	24	-0.01	0	N/A
hsa-miR-5585-5p	150-156	7mer-m8	-0.03	54	-0.03	0	N/A
hsa-miR-3190-3p	153-159	7mer-m8	-0.08	65	-0.08	0	N/A
hsa-miR-6134	156-162	7mer-m8	-0.06	47	-0.06	0.024	N/A
hsa-miR-455-5p	164-170	7mer-m8	-0.17	84	-0.17	1.349	<0.1
hsa-miR-4698	168-174	7mer-m8	-0.02	59	-0.02	0	N/A

hsa-miR-186-3p	171-177	7mer-1A	-0.01	38	-0.01	0.442	N/A
hsa-miR-891b	177-184	8mer	-0.03	36	-0.03	0.099	N/A
hsa-miR-4709-3p	193-199	7mer-1A	-0.02	44	-0.02	0.312	N/A
hsa-miR-27b-3p	200-207	8mer	-0.05	67	-0.05	1.696	＜0.1
hsa-miR-27a-3p	200-207	8mer	-0.05	67	-0.05	1.696	＜0.1
hsa-miR-3681-3p	200-206	7mer-1A	-0.02	48	-0.02	2.853	0.25
hsa-miR-128-3p	200-206	7mer-1A	-0.01	31	-0.01	2.853	0.25
hsa-miR-216a-3p	200-206	7mer-1A	-0.01	30	-0.01	2.853	0.25
hsa-miR-513a-5p	201-207	7mer-1A	-0.01	34	-0.01	0.099	N/A
hsa-miR-3977	204-210	7mer-m8	-0.02	39	-0.02	0	N/A
hsa-miR-3607-3p	211-218	8mer	-0.03	59	-0.03	0	N/A
hsa-miR-3686	212-219	8mer	-0.03	37	-0.03	0	N/A
hsa-miR-4743-3p	214-220	7mer-1A	-0.01	39	-0.01	0	N/A
hsa-miR-4652-3p	214-220	7mer-1A	-0.01	36	-0.01	0	N/A
hsa-miR-6854-3p	217-224	8mer	-0.36	91	-0.36	0	N/A
hsa-miR-1287-5p	224-231	8mer	-0.19	93	-0.19	0	N/A
hsa-miR-3135b	225-231	7mer-1A	-0.05	59	-0.05	0	N/A
hsa-miR-195-3p	234-240	7mer-m8	-0.02	41	-0.02	0.085	N/A
hsa-miR-16-2-3p	234-240	7mer-m8	-0.02	40	-0.02	0.085	N/A
hsa-miR-338-5p	235-241	7mer-m8	-0.02	51	-0.02	0.085	N/A

hsa-miR-7845-5p	239-245	7mer-m8	-0.17	81	-0.17	0	N/A
hsa-miR-4475	240-246	7mer-m8	-0.22	94	-0.22	0	N/A
hsa-miR-7158-5p	246-252	7mer-1A	-0.03	61	-0.03	0	N/A
hsa-miR-767-3p	247-254	8mer	-0.19	91	-0.19	0.085	N/A
hsa-miR-6828-3p	248-255	8mer	-0.22	93	-0.22	0	N/A
hsa-miR-6071	249-255	7mer-1A	-0.12	78	-0.12	0	N/A
hsa-miR-544a	250-256	7mer-1A	-0.14	80	-0.14	0.024	N/A
hsa-miR-6738-3p	250-256	7mer-1A	-0.01	32	-0.01	0	N/A
hsa-miR-3606-3p	253-260	8mer	-0.03	89	-0.03	0	N/A
hsa-miR-513c-3p	253-260	8mer	-0.03	89	-0.03	0	N/A
hsa-miR-513a-3p	253-260	8mer	-0.03	89	-0.03	0	N/A
hsa-miR-577	257-263	7mer-m8	-0.02	38	-0.02	0.024	N/A
hsa-miR-488-3p	262-268	7mer-1A	-0.07	77	-0.07	0.638	N/A
hsa-miR-510-3p	263-269	7mer-1A	-0.01	43	-0.01	0	N/A
hsa-miR-6800-3p	269-275	7mer-1A	-0.05	67	-0.05	0	N/A
hsa-miR-3144-3p	271-277	7mer-m8	-0.02	19	-0.02	0	N/A
hsa-miR-3671	276-283	8mer	-0.03	59	-0.03	0	N/A
hsa-miR-607	277-284	8mer	-0.03	85	-0.03	0	N/A
hsa-miR-1305	279-285	7mer-1A	-0.01	49	-0.01	0	N/A
hsa-miR-335-3p	280-286	7mer-1A	-0.01	58	-0.01	0.085	N/A
hsa-miR-5003-3p	291-297	7mer-1A	-0.01	46	-0.01	0	N/A
hsa-miR-4668-3p	305-311	7mer-m8	-0.02	74	-0.02	0	N/A
hsa-miR-548c-3p	306-313	8mer	-0.03	93	-0.03	0.024	N/A
hsa-miR-95-5p	310-317	8mer	-0.03	47	-0.03	0.085	N/A
hsa-miR-6793-3p	318-324	7mer-1A	-0.01	53	-0.01	0	N/A

hsa-miR-5695	326-332	7mer-m8	-0.08	72	-0.08	0	N/A
hsa-miR-3145-5p	327-333	7mer-m8	-0.04	53	-0.04	0.085	N/A
hsa-miR-548ah-3p	331-337	7mer-m8	-0.02	67	-0.02	0.085	N/A
hsa-miR-548aj-3p	331-337	7mer-m8	-0.02	67	-0.02	0.085	N/A
hsa-miR-548aq-3p	331-337	7mer-m8	-0.02	67	-0.02	0.085	N/A
hsa-miR-548ae-3p	331-337	7mer-m8	-0.02	67	-0.02	0.085	N/A
hsa-miR-548am-3p	331-337	7mer-m8	-0.02	67	-0.02	0.085	N/A
hsa-miR-548x-3p	331-337	7mer-m8	-0.02	67	-0.02	0.085	N/A
hsa-miR-548j-3p	331-337	7mer-m8	-0.02	67	-0.02	0.085	N/A
hsa-miR-1273c	340-346	7mer-m8	-0.24	89	-0.24	0	N/A
hsa-miR-4670-5p	342-348	7mer-1A	-0.24	78	-0.24	0	N/A
hsa-miR-6826-5p	346-352	7mer-1A	-0.01	24	-0.01	0	N/A
hsa-miR-551b-5p	349-355	7mer-m8	-0.02	62	-0.02	0.504	N/A
hsa-miR-548c-3p	351-358	8mer	-0.03	93	-0.03	0.024	N/A
hsa-miR-548ao-5p	356-363	8mer	-0.07	53	-0.07	0	N/A
hsa-miR-548ax	356-363	8mer	-0.07	53	-0.07	0	N/A
hsa-miR-5585-5p	357-363	7mer-1A	-0.01	28	-0.01	0	N/A
hsa-miR-3616-5p	358-364	7mer-1A	-0.1	79	-0.1	0	N/A
hsa-miR-573	358-364	7mer-1A	-0.09	76	-0.09	0	N/A
hsa-miR-6077	369-376	8mer	-0.03	65	-0.03	0	N/A
hsa-miR-7-5p	370-376	7mer-1A	-0.01	39	-0.01	1.466	0.1
hsa-miR-6079	371-377	7mer-1A	-0.01	41	-0.01	0	N/A
hsa-miR-502-5p	378-384	7mer-1A	-0.01	35	-0.01	0.024	N/A
hsa-miR-6875-3p	381-387	7mer-m8	-0.02	52	-0.02	0	N/A
hsa-miR-4778-3p	382-388	7mer-m8	-0.02	50	-0.02	0	N/A

hsa-miR-103a-2-5p	383-389	7mer-m8	-0.02	33	-0.02	0.494	N/A
hsa-miR-634	391-398	8mer	-0.03	23	-0.03	0.024	N/A
hsa-miR-373-3p	398-404	7mer-m8	-0.02	36	-0.02	1.504	<0.1
hsa-miR-302e	398-404	7mer-m8	-0.02	29	-0.02	1.504	<0.1
hsa-miR-302a-3p	398-404	7mer-m8	-0.02	29	-0.02	1.504	<0.1
hsa-miR-302c-3p.1	398-404	7mer-m8	-0.02	29	-0.02	1.504	<0.1
hsa-miR-302b-3p	398-404	7mer-m8	-0.02	29	-0.02	1.504	<0.1
hsa-miR-302d-3p	398-404	7mer-m8	-0.02	29	-0.02	1.504	<0.1
hsa-miR-520d-3p	398-404	7mer-m8	-0.02	28	-0.02	1.504	<0.1
hsa-miR-520e	398-404	7mer-m8	-0.02	28	-0.02	1.504	<0.1
hsa-miR-520c-3p	398-404	7mer-m8	-0.02	27	-0.02	1.504	<0.1
hsa-miR-372-3p	398-404	7mer-m8	-0.02	27	-0.02	1.504	<0.1
hsa-miR-520a-3p	398-404	7mer-m8	-0.02	27	-0.02	1.504	<0.1
hsa-miR-520b	398-404	7mer-m8	-0.02	27	-0.02	1.504	<

							0.1
hsa-miR-451b	402-408	7mer-1A	-0.01	21	-0.01	0	N/A
hsa-miR-6755-5p	406-412	7mer-m8	-0.02	17	-0.02	0	N/A
hsa-miR-6823-5p	408-414	7mer-1A	-0.04	55	-0.04	0	N/A
hsa-miR-4518	409-415	7mer-m8	-0.02	58	-0.02	0.024	N/A
hsa-miR-1266-5p	409-415	7mer-m8	-0.02	49	-0.02	0.024	N/A
hsa-miR-510-5p	410-416	7mer-m8	-0.02	36	-0.02	0	N/A
hsa-miR-4680-5p	413-419	7mer-1A	-0.01	21	-0.01	0	N/A
hsa-miR-6744-5p	417-423	7mer-1A	-0.01	23	-0.01	0	N/A
hsa-miR-1255b-5p	417-423	7mer-1A	-0.01	18	-0.01	0.099	N/A
hsa-miR-1255a	417-423	7mer-1A	-0.01	15	-0.01	0.099	N/A
hsa-miR-3116	420-426	7mer-m8	-0.07	70	-0.07	0.024	N/A
hsa-miR-1254	420-426	7mer-m8	-0.05	54	-0.05	0.024	N/A
hsa-miR-2861	422-429	8mer	-0.09	66	-0.09	0	N/A
hsa-miR-6724-5p	424-430	7mer-1A	-0.05	54	-0.05	0	N/A
hsa-miR-6773-5p	424-430	7mer-1A	-0.04	46	-0.04	0	N/A
hsa-miR-3147	425-431	7mer-m8	-0.06	70	-0.06	0	N/A
hsa-miR-4259	426-432	7mer-m8	-0.02	33	-0.02	0	N/A
hsa-miR-27a-3p	430-436	7mer-m8	-0.02	47	-0.02	1.913	＜ 0.1
hsa-miR-27b-3p	430-436	7mer-m8	-0.02	46	-0.02	1.913	＜ 0.1
hsa-miR-128-3p	430-436	7mer-1A	-0.01	31	-0.01	1.913	＜ 0.1
hsa-miR-216a-3p	430-436	7mer-1A	-0.01	30	-0.01	1.913	＜

							0.1
hsa-miR-3681-3p	430-436	7mer-1A	-0.01	28	-0.01	1.913	<0.1
hsa-miR-1178-3p	433-440	8mer	-0.04	64	-0.04	0.024	N/A
hsa-miR-6841-3p	437-443	7mer-1A	-0.14	72	-0.14	0	N/A
hsa-miR-502-5p	437-443	7mer-m8	-0.04	62	-0.04	0.099	N/A
hsa-miR-1915-5p	437-443	7mer-1A	-0.01	24	-0.01	0	N/A
hsa-miR-4448	439-446	8mer	-0.03	58	-0.03	0	N/A
hsa-miR-6772-3p	440-446	7mer-m8	-0.05	53	-0.05	0	N/A
hsa-miR-4268	440-446	7mer-1A	-0.01	33	-0.01	0	N/A
hsa-miR-218-5p	443-449	7mer-1A	-0.01	28	-0.01	0.069	<0.1
hsa-miR-4474-3p	449-456	8mer	-0.12	73	-0.12	0	N/A
hsa-miR-7108-5p	450-456	7mer-1A	-0.01	41	-0.01	0	N/A
hsa-miR-8087	455-461	7mer-m8	-0.02	57	-0.02	0	N/A
hsa-miR-6077	458-464	7mer-1A	-0.01	38	-0.01	0	N/A
hsa-miR-875-3p	460-466	7mer-1A	-0.03	49	-0.03	0	N/A
hsa-miR-1299	460-466	7mer-m8	-0.04	47	-0.04	0.024	N/A
hsa-miR-6128	460-466	7mer-1A	-0.01	33	-0.01	0.024	N/A
hsa-miR-518c-5p	461-467	7mer-m8	-0.02	31	-0.02	0	N/A
hsa-miR-6780a-3p	463-469	7mer-m8	-0.11	76	-0.11	0	N/A
hsa-miR-5196-3p	465-471	7mer-m8	-0.08	76	-0.08	0	N/A
hsa-miR-6128	476-482	7mer-m8	-0.02	47	-0.02	0.024	N/A
hsa-miR-7843-3p	490-496	7mer-1A	-0.02	36	-0.02	0	N/A
hsa-miR-127-5p	490-496	7mer-1A	-0.01	26	-0.01	0	N/A

hsa-miR-4635	491-497	7mer-m8	-0.02	58	-0.02	0	N/A
hsa-miR-671-5p	497-504	8mer	-0.15	82	-0.15	0.085	N/A
hsa-miR-6079	498-504	7mer-m8	-0.06	77	-0.06	0	N/A
hsa-miR-6828-5p	498-504	7mer-1A	-0.11	71	-0.11	0.085	N/A
hsa-miR-3190-3p	499-505	7mer-m8	-0.08	62	-0.08	0	N/A
hsa-miR-6499-3p	503-509	7mer-1A	-0.01	23	-0.01	0	N/A
hsa-miR-6516-5p	504-511	8mer	-0.03	60	-0.03	0	N/A
hsa-miR-455-3p.2	504-510	7mer-1A	-0.01	46	-0.01	1.231	﹤ 0.1
hsa-miR-557	506-512	7mer-1A	-0.01	51	-0.01	0.099	N/A
hsa-miR-507	506-512	7mer-1A	-0.01	43	-0.01	0.099	N/A
hsa-miR-3680-3p	506-512	7mer-1A	-0.01	33	-0.01	0	N/A
hsa-miR-450b-5p	506-512	7mer-1A	-0.01	23	-0.01	0.085	N/A
hsa-miR-19a-5p	507-514	8mer	-0.03	63	-0.03	0.085	N/A
hsa-miR-19b-2-5p	507-514	8mer	-0.03	62	-0.03	0.085	N/A
hsa-miR-19b-1-5p	507-514	8mer	-0.03	62	-0.03	0.085	N/A
hsa-miR-2052	508-514	7mer-1A	-0.01	18	-0.01	0	N/A
hsa-miR-4255	511-517	7mer-1A	-0.04	67	-0.04	0	N/A
hsa-miR-142-3p.2	511-518	8mer	-0.03	49	-0.03	0.229	﹤ 0.1
hsa-miR-3591-5p	512-519	8mer	-0.06	63	-0.06	0	N/A
hsa-miR-432-5p	522-528	7mer-1A	-0.03	45	-0.03	0.099	N/A
hsa-miR-1248	525-531	7mer-m8	-0.02	39	-0.02	0.024	N/A
hsa-miR-1245b-5p	528-534	7mer-m8	-0.09	79	-0.09	0	N/A
hsa-miR-3142	528-534	7mer-m8	-0.06	66	-0.06	0	N/A

hsa-miR-4493	530-537	8mer	-0.2	88	-0.2	0	N/A
hsa-miR-6126	531-537	7mer-m8	-0.02	62	-0.02	0	N/A
hsa-miR-605-3p	531-537	7mer-1A	-0.08	55	-0.08	0	N/A
hsa-miR-8060	533-539	7mer-m8	-0.02	49	-0.02	0	N/A
hsa-miR-6508-3p	537-543	7mer-m8	-0.02	28	-0.02	0	N/A
hsa-miR-6762-5p	538-545	8mer	-0.1	50	-0.1	0.085	N/A
hsa-miR-6845-5p	538-545	8mer	-0.09	49	-0.09	0.085	N/A
hsa-miR-128-1-5p	539-545	7mer-1A	-0.07	54	-0.07	0.085	N/A
hsa-miR-1227-5p	539-545	7mer-m8	-0.02	43	-0.02	0.085	N/A
hsa-miR-128-2-5p	539-545	7mer-1A	-0.02	36	-0.02	0.085	N/A
hsa-miR-4731-5p	541-548	8mer	-0.03	45	-0.03	0	N/A
hsa-miR-5589-5p	542-548	7mer-1A	-0.01	37	-0.01	0	N/A
hsa-miR-3692-5p	543-549	7mer-m8	-0.02	41	-0.02	0	N/A
hsa-miR-370-3p	544-550	7mer-m8	-0.02	41	-0.02	1.052	N/A
hsa-miR-6893-3p	544-550	7mer-m8	-0.02	31	-0.02	1.052	N/A
hsa-miR-1234-3p	547-553	7mer-m8	-0.12	63	-0.12	0.099	N/A
hsa-miR-7107-5p	547-553	7mer-m8	-0.11	62	-0.11	0.099	N/A
hsa-miR-6850-3p	549-555	7mer-1A	-0.22	85	-0.22	0	N/A
hsa-miR-101-3p.1	559-566	8mer	-0.03	63	-0.03	0.898	< 0.1
hsa-miR-101-3p.2	560-566	7mer-m8	-0.02	40	-0.02	1.233	< 0.1
hsa-miR-144-3p	560-566	7mer-1A	-0.01	29	-0.01	1.233	< 0.1
hsa-miR-4659b-5p	573-579	7mer-m8	-0.05	56	-0.05	0	N/A

hsa-miR-4659a-5p	573-579	7mer-m8	-0.03	49	-0.03	0	N/A
hsa-miR-4769-3p	574-580	7mer-m8	-0.02	44	-0.02	0	N/A
hsa-miR-6817-5p	574-580	7mer-m8	-0.02	43	-0.02	0	N/A
hsa-miR-6727-3p	575-581	7mer-m8	-0.07	59	-0.07	0	N/A
hsa-miR-4722-3p	575-581	7mer-m8	-0.06	52	-0.06	0	N/A
hsa-miR-1183	582-588	7mer-1A	-0.01	34	-0.01	0.024	N/A
hsa-miR-759	597-603	7mer-m8	-0.02	51	-0.02	0.099	N/A
hsa-miR-4797-5p	598-604	7mer-m8	-0.02	63	-0.02	0	N/A
hsa-miR-7845-5p	602-608	7mer-m8	-0.02	13	-0.02	0	N/A
hsa-miR-4475	603-609	7mer-m8	-0.02	51	-0.02	0	N/A
hsa-miR-7515	604-610	7mer-m8	-0.02	18	-0.02	0	N/A
hsa-miR-3202	605-611	7mer-m8	-0.05	56	-0.05	0	N/A
hsa-miR-6876-5p	606-612	7mer-m8	-0.02	49	-0.02	0	N/A
hsa-miR-4476	606-612	7mer-m8	-0.02	49	-0.02	0	N/A
hsa-miR-6840-3p	609-615	7mer-m8	-0.02	49	-0.02	0	N/A
hsa-miR-6844	616-622	7mer-1A	-0.01	30	-0.01	0	N/A
hsa-miR-4778-3p	618-624	7mer-1A	-0.01	32	-0.01	0	N/A
hsa-miR-4659a-3p	619-626	8mer	-0.03	56	-0.03	0	N/A
hsa-miR-4659b-3p	619-626	8mer	-0.03	56	-0.03	0	N/A
hsa-miR-6875-3p	620-626	7mer-1A	-0.01	31	-0.01	0	N/A
hsa-miR-1468-5p	624-630	7mer-m8	-0.04	16	-0.04	0	N/A
hsa-miR-1470	627-633	7mer-m8	-0.02	61	-0.02	0	N/A
hsa-miR-6887-3p	628-635	8mer	-0.11	62	-0.11	0	N/A
hsa-miR-6795-3p	629-635	7mer-1A	-0.14	82	-0.14	0	N/A
hsa-miR-6826-3p	629-635	7mer-m8	-0.08	63	-0.08	0	N/A

hsa-miR-6729-3p	631-637	7mer-m8	-0.17	84	-0.17	0	N/A
hsa-miR-651-3p	644-650	7mer-1A	-0.01	28	-0.01	0	N/A
hsa-miR-4521	645-652	8mer	-0.03	55	-0.03	0	N/A
hsa-miR-1264	650-656	7mer-1A	-0.05	74	-0.05	0.024	N/A
hsa-miR-3184-3p	650-656	7mer-1A	-0.01	40	-0.01	0	N/A
hsa-miR-548aq-5p	654-660	7mer-m8	-0.02	53	-0.02	0.099	N/A
hsa-miR-559	654-660	7mer-m8	-0.02	44	-0.02	0.099	N/A
hsa-miR-548as-5p	654-660	7mer-m8	-0.02	43	-0.02	0.099	N/A
hsa-miR-548h-5p	654-660	7mer-m8	-0.02	42	-0.02	0.099	N/A
hsa-miR-548j-5p	654-660	7mer-m8	-0.02	42	-0.02	0.099	N/A
hsa-miR-548ap-5p	654-660	7mer-m8	-0.02	42	-0.02	0.099	N/A
hsa-miR-548ak	654-660	7mer-m8	-0.02	41	-0.02	0.099	N/A
hsa-miR-548o-5p	654-660	7mer-m8	-0.02	41	-0.02	0.099	N/A
hsa-miR-548d-5p	654-660	7mer-m8	-0.02	41	-0.02	0.099	N/A
hsa-miR-548ay-5p	654-660	7mer-m8	-0.02	41	-0.02	0.099	N/A
hsa-miR-548w	654-660	7mer-m8	-0.02	41	-0.02	0.099	N/A
hsa-miR-548c-5p	654-660	7mer-m8	-0.02	41	-0.02	0.099	N/A
hsa-miR-548b-5p	654-660	7mer-m8	-0.02	41	-0.02	0.099	N/A
hsa-miR-548ad-5p	654-660	7mer-m8	-0.02	41	-0.02	0.099	N/A
hsa-miR-548au-5p	654-660	7mer-m8	-0.02	41	-0.02	0.099	N/A
hsa-miR-548bb-5p	654-660	7mer-m8	-0.02	41	-0.02	0.099	N/A
hsa-miR-548am-5p	654-660	7mer-m8	-0.02	41	-0.02	0.099	N/A
hsa-miR-548ae-5p	654-660	7mer-m8	-0.02	41	-0.02	0.099	N/A
hsa-miR-548y	654-660	7mer-m8	-0.02	40	-0.02	0.099	N/A
hsa-miR-548i	654-660	7mer-m8	-0.02	40	-0.02	0.099	N/A

hsa-miR-548a-5p	654-660	7mer-m8	-0.02	40	-0.02	0.099	N/A
hsa-miR-548ar-5p	654-660	7mer-m8	-0.02	39	-0.02	0.099	N/A
hsa-miR-548ab	654-660	7mer-m8	-0.02	39	-0.02	0.099	N/A
hsa-miR-548n	655-661	7mer-m8	-0.02	53	-0.02	0.099	N/A
hsa-miR-520d-5p	659-665	7mer-1A	-0.01	52	-0.01	0.085	N/A
hsa-miR-524-5p	659-665	7mer-1A	-0.01	51	-0.01	0.085	N/A
hsa-miR-2681-5p	662-668	7mer-1A	-0.01	53	-0.01	0	N/A
hsa-miR-153-5p	664-670	7mer-m8	-0.02	50	-0.02	0.085	N/A
hsa-miR-450a-2-3p	665-676	non-canonical	N/A	N/A	N/A	0	N/A
hsa-miR-450a-2-3p	665-676	non-canonical	N/A	N/A	N/A	0	N/A
hsa-miR-6810-5p	670-676	7mer-m8	-0.15	84	-0.15	0	N/A
hsa-miR-4652-5p	670-676	7mer-1A	-0.14	71	-0.14	0	N/A
hsa-miR-3191-3p	670-676	7mer-1A	-0.1	62	-0.1	0	N/A
hsa-miR-3144-5p	670-676	7mer-1A	-0.06	47	-0.06	0	N/A
hsa-miR-491-5p	671-677	7mer-m8	-0.06	42	-0.06	0.142	N/A
hsa-miR-126-3p.2	676-682	7mer-m8	-0.23	65	-0.23	1.13	＜0.1
hsa-miR-6763-5p	686-692	7mer-m8	-0.1	66	-0.1	0.085	N/A
hsa-miR-1343-5p	686-692	7mer-1A	-0.05	62	-0.05	0.099	N/A
hsa-miR-3150a-3p	686-692	7mer-m8	-0.07	59	-0.07	0.085	N/A
hsa-miR-3175	686-692	7mer-1A	-0.1	58	-0.1	0	N/A
hsa-miR-939-5p	686-692	7mer-1A	-0.04	51	-0.04	0.099	N/A
hsa-miR-6825-5p	686-692	7mer-1A	-0.03	23	-0.03	0	N/A

hsa-miR-491-5p	687-693	7mer-m8	-0.06	43	-0.06	0.22	N/A
hsa-miR-1296-3p	688-694	7mer-m8	-0.08	69	-0.08	0.085	N/A
hsa-miR-194-3p	689-696	8mer	-0.08	75	-0.08	0	N/A
hsa-miR-5693	690-696	7mer-1A	-0.01	43	-0.01	0	N/A
hsa-miR-5094	691-697	7mer-m8	-0.02	31	-0.02	0	N/A
hsa-miR-6884-3p	694-700	7mer-1A	-0.01	37	-0.01	0	N/A
hsa-miR-7152-3p	698-704	7mer-m8	-0.17	85	-0.17	0	N/A
hsa-miR-497-3p	704-710	7mer-m8	-0.02	68	-0.02	0.085	N/A
hsa-miR-4261	707-713	7mer-1A	-0.01	14	-0.01	0	N/A
hsa-miR-875-3p	708-714	7mer-m8	-0.02	33	-0.02	0.085	N/A
hsa-miR-6847-3p	716-722	7mer-m8	-0.03	53	-0.03	0	N/A
hsa-miR-574-3p	718-724	7mer-m8	-0.23	78	-0.23	0	N/A
hsa-miR-622	726-732	7mer-1A	-0.01	28	-0.01	0.099	N/A
hsa-miR-6882-5p	731-737	7mer-m8	-0.05	73	-0.05	0	N/A
hsa-miR-7-5p	740-747	8mer	-0.16	90	-0.16	0.245	< 0.1
hsa-miR-6077	741-747	7mer-1A	-0.01	38	-0.01	0	N/A
hsa-miR-206	746-752	7mer-1A	-0.03	50	-0.03	2.165	< 0.1
hsa-miR-1-3p	746-752	7mer-1A	-0.03	50	-0.03	2.165	< 0.1
hsa-miR-613	746-752	7mer-1A	-0.01	24	-0.01	2.165	< 0.1
hsa-miR-548o-3p	755-761	7mer-1A	-0.01	38	-0.01	0.099	N/A
hsa-miR-1323	755-761	7mer-1A	-0.01	36	-0.01	0.099	N/A

hsa-miR-607	757-763	7mer-1A	-0.01	40	-0.01	0	N/A
hsa-miR-4682	762-768	7mer-m8	-0.06	71	-0.06	0	N/A
hsa-miR-103a-3p	770-776	7mer-m8	-0.04	49	-0.04	0.242	< 0.1
hsa-miR-107	770-776	7mer-m8	-0.04	49	-0.04	0.242	< 0.1
hsa-miR-486-3p	773-779	7mer-m8	-0.14	57	-0.14	0.085	N/A
hsa-miR-4688	774-780	7mer-m8	-0.1	64	-0.1	0	N/A
hsa-miR-6743-5p	774-780	7mer-m8	-0.08	56	-0.08	0	N/A
hsa-miR-4524b-3p	778-784	7mer-m8	-0.02	56	-0.02	0	N/A
hsa-miR-4696	781-787	7mer-m8	-0.08	73	-0.08	0	N/A
hsa-miR-451b	782-788	7mer-m8	-0.02	30	-0.02	0	N/A
hsa-miR-3120-3p	784-790	7mer-m8	-0.02	35	-0.02	0	N/A
hsa-miR-2277-3p	786-792	7mer-1A	-0.04	50	-0.04	0	N/A
hsa-miR-433-3p	790-796	7mer-1A	-0.01	29	-0.01	1.126	N/A
hsa-miR-7154-5p	790-796	7mer-1A	-0.01	19	-0.01	0	N/A
hsa-miR-29a-5p	795-801	7mer-m8	-0.02	33	-0.02	0	N/A
hsa-miR-8075	797-803	7mer-1A	-0.1	60	-0.1	0	N/A
hsa-miR-628-5p	798-804	7mer-1A	-0.05	66	-0.05	0.085	N/A
hsa-miR-4723-3p	804-811	8mer	-0.09	72	-0.09	0	N/A
hsa-miR-6769b-3p	804-811	8mer	-0.08	71	-0.08	0	N/A
hsa-miR-3183	804-811	8mer	-0.03	67	-0.03	0	N/A
hsa-miR-7111-3p	805-811	7mer-m8	-0.07	70	-0.07	0	N/A
hsa-miR-5196-3p	807-814	8mer	-0.12	82	-0.12	0	N/A
hsa-miR-4793-5p	808-814	7mer-1A	-0.01	27	-0.01	0	N/A

hsa-miR-592	812-818	7mer-m8	-0.05	50	-0.05	0.085	N/A
hsa-miR-597-5p	812-818	7mer-1A	-0.01	21	-0.01	0.085	N/A
hsa-miR-4795-3p	822-829	8mer	-0.03	63	-0.03	0	N/A
hsa-miR-126-5p	822-828	7mer-1A	-0.01	38	-0.01	0	N/A
hsa-miR-6128	835-841	7mer-m8	-0.02	47	-0.02	0.099	N/A
hsa-miR-3652	837-843	7mer-m8	-0.1	76	-0.1	0	N/A
hsa-miR-4430	837-843	7mer-m8	-0.08	71	-0.08	0	N/A
hsa-miR-4492	838-845	8mer	-0.26	95	-0.26	0.099	N/A
hsa-miR-5001-5p	838-845	8mer	-0.34	95	-0.34	0.099	N/A
hsa-miR-4498	838-845	8mer	-0.32	94	-0.32	0.099	N/A
hsa-miR-762	838-845	8mer	-0.26	94	-0.26	0.099	N/A
hsa-miR-1587	839-845	7mer-m8	-0.13	73	-0.13	0	N/A
hsa-miR-4656	839-845	7mer-1A	-0.12	73	-0.12	0	N/A
hsa-miR-4741	839-845	7mer-1A	-0.07	68	-0.07	0	N/A
hsa-miR-3620-5p	839-845	7mer-m8	-0.02	40	-0.02	0	N/A
hsa-miR-4675	839-845	7mer-1A	-0.01	34	-0.01	0	N/A
hsa-miR-6829-5p	839-845	7mer-1A	-0.01	26	-0.01	0	N/A
hsa-miR-4417	840-847	8mer	-0.14	82	-0.14	0	N/A
hsa-miR-4322	841-847	7mer-m8	-0.18	87	-0.18	0	N/A
hsa-miR-4296	841-847	7mer-m8	-0.18	86	-0.18	0	N/A
hsa-miR-4265	841-847	7mer-m8	-0.18	85	-0.18	0	N/A
hsa-miR-299-3p	842-848	7mer-m8	-0.09	71	-0.09	0.32	N/A
hsa-miR-4502	854-860	7mer-m8	-0.02	40	-0.02	0	N/A
hsa-miR-8075	855-862	8mer	-0.15	74	-0.15	0	N/A
hsa-miR-4728-3p	857-863	7mer-m8	-0.02	28	-0.02	0	N/A

hsa-miR-7853-5p	860-866	7mer-m8	-0.02	68	-0.02	0	N/A
hsa-miR-105-5p	860-866	7mer-m8	-0.02	68	-0.02	0	N/A
hsa-miR-133b	866-872	7mer-m8	-0.14	76	-0.14	0.387	＜0.1
hsa-miR-133a-3p.2	866-872	7mer-m8	-0.14	76	-0.14	0.387	＜0.1
hsa-miR-133a-3p.1	866-872	7mer-1A	-0.02	26	-0.02	0.387	＜0.1
hsa-miR-664b-5p	873-879	7mer-1A	-0.18	71	-0.18	0	N/A
hsa-miR-4417	874-881	8mer	-0.14	82	-0.14	0	N/A
hsa-miR-4265	875-881	7mer-m8	-0.12	77	-0.12	0	N/A
hsa-miR-4322	875-881	7mer-m8	-0.11	77	-0.11	0	N/A
hsa-miR-4296	875-881	7mer-m8	-0.12	77	-0.12	0	N/A
hsa-miR-134-3p	876-882	7mer-m8	-0.02	40	-0.02	0.085	N/A
hsa-miR-1207-3p	880-886	7mer-1A	-0.01	34	-0.01	0.024	N/A
hsa-miR-6787-3p	881-888	8mer	-0.03	64	-0.03	0	N/A
hsa-miR-3692-3p	890-896	7mer-m8	-0.02	32	-0.02	0.085	N/A
hsa-miR-4678	895-902	8mer	-0.09	70	-0.09	0	N/A
hsa-miR-331-5p	896-903	8mer	-0.15	81	-0.15	0.085	N/A
hsa-miR-6509-5p	897-903	7mer-m8	-0.02	14	-0.02	0	N/A
hsa-miR-362-5p	902-908	7mer-1A	-0.07	70	-0.07	0.138	N/A
hsa-miR-500b-5p	902-908	7mer-1A	-0.06	66	-0.06	0.138	N/A
hsa-miR-501-5p	902-908	7mer-1A	-0.01	22	-0.01	0.085	N/A
hsa-miR-1250-5p	908-914	7mer-m8	-0.22	72	-0.22	0.099	N/A
hsa-miR-4746-3p	909-915	7mer-m8	-0.3	92	-0.3	0	N/A

hsa-miR-4737	923-929	7mer-1A	-0.18	61	-0.18	0	N/A
hsa-miR-450a-5p	924-931	8mer	-0.21	72	-0.21	0.189	N/A
hsa-miR-129-5p	926-932	7mer-m8	-0.02	81	-0.02	0.189	＜ 0.1
hsa-miR-6888-5p	936-943	8mer	-0.1	86	-0.1	0	N/A
hsa-miR-4266	938-944	7mer-1A	-0.07	52	-0.07	0	N/A
hsa-miR-372-5p	944-950	7mer-m8	-0.02	63	-0.02	0.085	N/A
hsa-miR-2114-3p	948-955	8mer	-0.26	91	-0.26	0.099	N/A
hsa-miR-6823-3p	948-955	8mer	-0.21	87	-0.21	0.099	N/A
hsa-miR-1199-5p	950-957	8mer	-0.18	89	-0.18	0.024	N/A
hsa-miR-6751-3p	950-957	8mer	-0.17	88	-0.17	0.024	N/A
hsa-miR-4303	951-957	7mer-m8	-0.04	68	-0.04	0	N/A
hsa-miR-3200-5p	952-958	7mer-m8	-0.02	39	-0.02	0	N/A
hsa-miR-452-3p	954-961	8mer	-0.03	52	-0.03	0	N/A
hsa-miR-586	961-967	7mer-m8	-0.02	40	-0.02	0.024	N/A
hsa-miR-492	968-974	7mer-m8	-0.14	68	-0.14	0.099	N/A
hsa-miR-6816-3p	970-976	7mer-m8	-0.03	69	-0.03	0	N/A
hsa-miR-186-3p	974-980	7mer-m8	-0.02	52	-0.02	0	N/A
hsa-miR-5699-5p	976-983	8mer	-0.12	76	-0.12	0	N/A
hsa-miR-3189-5p	977-983	7mer-1A	-0.07	67	-0.07	0	N/A
hsa-miR-4758-3p	977-983	7mer-1A	-0.04	52	-0.04	0	N/A
hsa-miR-3192-3p	986-993	8mer	-0.03	65	-0.03	0	N/A
hsa-miR-3151-3p	986-992	7mer-1A	-0.04	52	-0.04	0	N/A
hsa-miR-4762-3p	987-993	7mer-m8	-0.02	51	-0.02	0	N/A
hsa-miR-3182	989-995	7mer-1A	-0.01	42	-0.01	0	N/A

hsa-miR-6740-3p	990-996	7mer-m8	-0.13	78	-0.13	0	N/A
hsa-miR-3674	996-1002	7mer-1A	-0.07	53	-0.07	0	N/A
hsa-miR-153-5p	1000-1007	8mer	-0.03	71	-0.03	0.085	N/A
hsa-miR-1250-3p	1001-1007	7mer-1A	-0.01	46	-0.01	0	N/A
hsa-miR-5696	1002-1008	7mer-1A	-0.02	63	-0.02	0	N/A
hsa-miR-664b-3p	1002-1008	7mer-1A	-0.01	42	-0.01	0.099	N/A
hsa-miR-579-3p	1002-1008	7mer-1A	-0.01	41	-0.01	0.099	N/A
hsa-miR-646	1008-1014	7mer-m8	-0.07	70	-0.07	0.024	N/A
hsa-miR-2278	1010-1016	7mer-m8	-0.02	51	-0.02	0.335	N/A
hsa-miR-6501-3p	1011-1018	8mer	-0.03	53	-0.03	0	N/A
hsa-miR-3942-3p	1014-1020	7mer-1A	-0.01	34	-0.01	0	N/A
hsa-miR-205-3p	1015-1021	7mer-m8	-0.02	69	-0.02	0	N/A
hsa-miR-6888-5p	1020-1026	7mer-m8	-0.09	86	-0.09	0	N/A
hsa-miR-4428	1021-1027	7mer-m8	-0.05	58	-0.05	0.024	N/A
hsa-miR-4509	1024-1030	7mer-m8	-0.02	43	-0.02	0	N/A
hsa-miR-6812-5p	1035-1042	8mer	-0.21	84	-0.21	0	N/A
hsa-miR-6819-5p	1035-1042	8mer	-0.2	83	-0.2	0	N/A
hsa-miR-6737-5p	1035-1042	8mer	-0.18	82	-0.18	0	N/A
hsa-miR-6747-5p	1035-1041	7mer-1A	-0.13	74	-0.13	0	N/A
hsa-miR-342-5p	1035-1041	7mer-1A	-0.09	68	-0.09	0.085	N/A
hsa-miR-4664-5p	1035-1041	7mer-1A	-0.06	65	-0.06	0.085	N/A
hsa-miR-608	1035-1041	7mer-1A	-0.14	52	-0.14	0	N/A
hsa-miR-4651	1035-1041	7mer-1A	-0.11	47	-0.11	0	N/A
hsa-miR-3170	1036-1042	7mer-1A	-0.07	62	-0.07	0	N/A
hsa-miR-6855-5p	1036-1042	7mer-1A	-0.05	51	-0.05	0	N/A

hsa-miR-5572	1036-1042	7mer-m8	-0.02	39	-0.02	0	N/A
hsa-miR-92a-1-5p	1038-1044	7mer-m8	-0.17	69	-0.17	0	N/A
hsa-miR-7109-5p	1043-1050	8mer	-0.33	94	-0.33	0	N/A
hsa-miR-328-5p	1043-1049	7mer-1A	-0.24	80	-0.24	0	N/A
hsa-miR-6885-5p	1043-1049	7mer-1A	-0.23	73	-0.23	0	N/A
hsa-miR-6795-5p	1043-1049	7mer-1A	-0.24	71	-0.24	0	N/A
hsa-miR-6887-5p	1043-1049	7mer-1A	-0.24	70	-0.24	0	N/A
hsa-miR-6751-5p	1044-1050	7mer-1A	-0.17	81	-0.17	0	N/A
hsa-miR-6803-5p	1044-1050	7mer-1A	-0.17	81	-0.17	0	N/A
hsa-miR-4665-5p	1044-1050	7mer-1A	-0.17	74	-0.17	0.024	N/A
hsa-miR-1275	1044-1050	7mer-1A	-0.08	65	-0.08	0.024	N/A
hsa-miR-5572	1045-1051	7mer-1A	-0.1	79	-0.1	0	N/A
hsa-miR-450a-2-3p	1045-1051	7mer-1A	-0.14	68	-0.14	0.494	N/A
hsa-miR-4260	1045-1051	7mer-1A	-0.04	48	-0.04	0	N/A
hsa-miR-4775	1049-1055	7mer-m8	-0.02	47	-0.02	0	N/A
hsa-miR-590-3p	1049-1055	7mer-1A	-0.01	34	-0.01	0.099	N/A
hsa-miR-4735-5p	1050-1056	7mer-m8	-0.02	33	-0.02	0	N/A
hsa-miR-3129-3p	1052-1058	7mer-m8	-0.02	50	-0.02	0	N/A
hsa-miR-5583-5p	1052-1058	7mer-m8	-0.02	49	-0.02	0	N/A
hsa-miR-587	1068-1074	7mer-m8	-0.02	39	-0.02	0.024	N/A
hsa-miR-141-5p	1071-1078	8mer	-0.14	85	-0.14	0	N/A
hsa-miR-4428	1083-1089	7mer-m8	-0.11	80	-0.11	0.024	N/A
hsa-miR-548ao-5p	1090-1097	8mer	-0.09	63	-0.09	0.085	N/A
hsa-miR-548ax	1090-1097	8mer	-0.09	63	-0.09	0.085	N/A

hsa-miR-5585-5p	1091-1097	7mer-1A	-0.01	41	-0.01	0	N/A
hsa-miR-573	1096-1103	8mer	-0.29	97	-0.29	0	N/A
hsa-miR-3616-5p	1096-1103	8mer	-0.25	96	-0.25	0	N/A
hsa-miR-4795-5p	1096-1102	7mer-1A	-0.13	78	-0.13	0	N/A
hsa-miR-3140-3p	1101-1107	7mer-m8	-0.04	60	-0.04	0	N/A
hsa-miR-208a-5p	1102-1108	7mer-m8	-0.02	57	-0.02	0.085	N/A
hsa-miR-208b-5p	1102-1108	7mer-m8	-0.02	54	-0.02	0.085	N/A
hsa-miR-6869-5p	1112-1118	7mer-1A	-0.02	47	-0.02	0	N/A
hsa-miR-8081	1113-1119	7mer-1A	-0.06	68	-0.06	0	N/A
hsa-miR-6750-5p	1128-1134	7mer-m8	-0.08	83	-0.08	0	N/A
hsa-miR-6822-5p	1128-1134	7mer-m8	-0.07	80	-0.07	0	N/A
hsa-miR-6797-5p	1130-1137	8mer	-0.13	72	-0.13	0.085	N/A
hsa-miR-1249-5p	1130-1137	8mer	-0.13	72	-0.13	0.085	N/A
hsa-miR-6515-5p	1131-1137	7mer-m8	-0.1	68	-0.1	0	N/A
hsa-miR-4254	1133-1139	7mer-m8	-0.02	42	-0.02	0	N/A
hsa-miR-619-3p	1134-1140	7mer-m8	-0.02	34	-0.02	0.024	N/A
hsa-miR-3661	1135-1142	8mer	-0.12	68	-0.12	0.099	N/A
hsa-miR-631	1135-1142	8mer	-0.13	67	-0.13	0.099	N/A
hsa-miR-486-3p	1144-1150	7mer-m8	-0.06	34	-0.06	0.085	N/A
hsa-miR-4697-5p	1146-1152	7mer-1A	-0.16	67	-0.16	0	N/A
hsa-miR-1237-5p	1146-1152	7mer-1A	-0.15	66	-0.15	0	N/A
hsa-miR-4488	1146-1152	7mer-1A	-0.15	65	-0.15	0	N/A
hsa-miR-6848-5p	1146-1152	7mer-1A	-0.13	57	-0.13	0	N/A
hsa-miR-6846-5p	1146-1152	7mer-1A	-0.12	55	-0.12	0	N/A
hsa-miR-6803-5p	1147-1153	7mer-1A	-0.15	76	-0.15	0	N/A

hsa-miR-6751-5p	1147-1153	7mer-1A	-0.11	71	-0.11	0	N/A
hsa-miR-7109-5p	1147-1153	7mer-1A	-0.13	69	-0.13	0	N/A
hsa-miR-1275	1147-1153	7mer-1A	-0.09	68	-0.09	0.024	N/A
hsa-miR-4665-5p	1147-1153	7mer-1A	-0.13	67	-0.13	0.024	N/A
hsa-miR-450a-2-3p	1148-1154	7mer-1A	-0.08	47	-0.08	0	N/A
hsa-miR-5572	1148-1154	7mer-1A	-0.01	26	-0.01	0.312	N/A
hsa-miR-4260	1148-1154	7mer-1A	-0.01	26	-0.01	0	N/A
hsa-miR-6740-5p	1149-1155	7mer-m8	-0.02	25	-0.02	0	N/A
hsa-miR-193b-5p	1152-1158	7mer-m8	-0.03	27	-0.03	0	N/A
hsa-miR-6732-5p	1154-1160	7mer-m8	-0.21	85	-0.21	0	N/A
hsa-miR-4779	1157-1163	7mer-m8	-0.11	79	-0.11	0	N/A
hsa-miR-6891-5p	1158-1165	8mer	-0.03	49	-0.03	0.024	N/A
hsa-miR-3173-3p	1158-1165	8mer	-0.03	48	-0.03	0.024	N/A
hsa-miR-4428	1159-1165	7mer-1A	-0.03	43	-0.03	0.024	N/A
hsa-miR-4504	1170-1177	8mer	-0.03	37	-0.03	0.024	N/A
hsa-miR-542-3p	1171-1177	7mer-1A	-0.06	61	-0.06	0.029	N/A
hsa-miR-6818-5p	1173-1180	8mer	-0.03	45	-0.03	0	N/A
hsa-miR-147a	1174-1180	7mer-1A	-0.01	54	-0.01	0.024	N/A
hsa-miR-5010-3p	1175-1182	8mer	-0.03	62	-0.03	0	N/A
hsa-miR-2113	1176-1182	7mer-1A	-0.01	42	-0.01	0	N/A
hsa-miR-1910-3p	1189-1195	7mer-m8	-0.02	49	-0.02	0	N/A
hsa-miR-6511a-5p	1189-1195	7mer-m8	-0.02	38	-0.02	0	N/A
hsa-miR-3714	1190-1196	7mer-m8	-0.02	49	-0.02	0	N/A
hsa-miR-3922-5p	1192-1198	7mer-1A	-0.01	34	-0.01	0	N/A

hsa-miR-5585-3p	1204-1211	8mer	-0.03	64	-0.03	0	N/A
hsa-miR-1273e	1208-1214	7mer-m8	-0.04	46	-0.04	0	N/A
hsa-miR-636	1209-1215	7mer-m8	-0.11	74	-0.11	0.085	N/A
hsa-miR-520f-3p	1210-1216	7mer-m8	-0.08	76	-0.08	0.22	< 0.1
hsa-miR-302c-3p.2	1210-1216	7mer-m8	-0.03	52	-0.03	0.22	< 0.1
hsa-miR-373-3p	1211-1218	8mer	-0.03	73	-0.03	0.245	< 0.1
hsa-miR-520b	1211-1218	8mer	-0.04	62	-0.04	0.245	< 0.1
hsa-miR-520c-3p	1211-1218	8mer	-0.04	62	-0.04	0.245	< 0.1
hsa-miR-302e	1211-1218	8mer	-0.03	61	-0.03	0.245	< 0.1
hsa-miR-520e	1211-1218	8mer	-0.03	60	-0.03	0.245	< 0.1
hsa-miR-302c-3p.1	1211-1218	8mer	-0.03	59	-0.03	0.245	< 0.1
hsa-miR-302d-3p	1211-1218	8mer	-0.03	59	-0.03	0.245	< 0.1
hsa-miR-520a-3p	1211-1218	8mer	-0.03	59	-0.03	0.245	< 0.1
hsa-miR-302a-3p	1211-1218	8mer	-0.03	59	-0.03	0.245	< 0.1
hsa-miR-520d-3p	1211-1218	8mer	-0.03	59	-0.03	0.245	<

							0.1
hsa-miR-302b-3p	1211-1218	8mer	-0.03	59	-0.03	0.245	< 0.1
hsa-miR-372-3p	1211-1218	8mer	-0.03	57	-0.03	0.245	< 0.1
hsa-miR-519a-3p	1212-1218	7mer-1A	-0.12	81	-0.12	0.099	N/A
hsa-miR-519b-3p	1212-1218	7mer-1A	-0.12	81	-0.12	0.099	N/A
hsa-miR-519c-3p	1212-1218	7mer-1A	-0.12	81	-0.12	0.099	N/A
hsa-miR-1537-5p	1216-1222	7mer-m8	-0.02	6	-0.02	0	N/A
hsa-miR-4435	1224-1230	7mer-m8	-0.12	76	-0.12	0	N/A
hsa-miR-7108-5p	1226-1233	8mer	-0.09	78	-0.09	0	N/A
hsa-miR-663b	1226-1232	7mer-1A	-0.02	41	-0.02	0.024	N/A
hsa-miR-4474-3p	1227-1233	7mer-1A	-0.03	35	-0.03	0	N/A
hsa-miR-146b-3p	1233-1240	8mer	-0.12	84	-0.12	0	N/A
hsa-miR-874-3p	1234-1240	7mer-m8	-0.08	68	-0.08	0.069	N/A
hsa-miR-7976	1234-1240	7mer-1A	-0.07	66	-0.07	0	N/A
hsa-miR-3173-5p	1234-1240	7mer-1A	-0.01	30	-0.01	0	N/A
hsa-miR-6799-3p	1234-1240	7mer-1A	-0.01	28	-0.01	0	N/A
hsa-miR-365b-3p	1236-1242	7mer-m8	-0.08	73	-0.08	0.069	< 0.1
hsa-miR-365a-3p	1236-1242	7mer-m8	-0.08	73	-0.08	0.069	< 0.1
hsa-miR-3662	1239-1245	7mer-1A	-0.01	47	-0.01	0	N/A
hsa-miR-3607-3p	1242-1248	7mer-m8	-0.02	46	-0.02	0	N/A
hsa-miR-6804-3p	1246-1252	7mer-m8	-0.11	65	-0.11	0	N/A

hsa-miR-1298-5p	1252-1258	7mer-m8	-0.02	55	-0.02	0.142	N/A
hsa-miR-616-3p	1254-1260	7mer-1A	-0.01	22	-0.01	0.024	N/A
hsa-miR-215-3p	1255-1261	7mer-m8	-0.02	47	-0.02	0	N/A
hsa-miR-5089-3p	1260-1266	7mer-m8	-0.07	55	-0.07	0	N/A
hsa-miR-155-5p	1263-1269	7mer-m8	-0.05	50	-0.05	0.812	＜ 0.1
hsa-miR-3692-3p	1277-1283	7mer-m8	-0.02	32	-0.02	0.085	N/A
hsa-miR-4452	1281-1287	7mer-m8	-0.02	66	-0.02	0	N/A
hsa-miR-4680-3p	1282-1288	7mer-m8	-0.02	47	-0.02	0	N/A
hsa-miR-3140-5p	1283-1289	7mer-m8	-0.03	57	-0.03	0	N/A
hsa-miR-4699-3p	1291-1298	8mer	-0.03	67	-0.03	0	N/A
hsa-miR-1279	1295-1301	7mer-1A	-0.01	42	-0.01	0	N/A
hsa-miR-5007-3p	1296-1302	7mer-1A	-0.01	36	-0.01	0	N/A
hsa-miR-652-5p	1305-1311	7mer-m8	-0.06	64	-0.06	0	N/A
hsa-miR-6715a-3p	1308-1314	7mer-1A	-0.11	80	-0.11	0	N/A
hsa-miR-6715b-3p	1308-1315	8mer	-0.06	80	-0.06	0	N/A
hsa-miR-607	1310-1316	7mer-1A	-0.01	40	-0.01	0	N/A
hsa-miR-4422	1323-1329	7mer-m8	-0.02	57	-0.02	0	N/A
hsa-miR-3611	1328-1334	7mer-1A	-0.05	64	-0.05	0	N/A
hsa-miR-409-3p	1332-1338	7mer-m8	-0.02	71	-0.02	0.245	N/A
hsa-miR-4772-3p	1338-1344	7mer-1A	-0.04	72	-0.04	0	N/A
hsa-miR-2276-3p	1338-1344	7mer-1A	-0.04	69	-0.04	0	N/A
hsa-miR-4799-5p	1348-1354	7mer-1A	-0.01	35	-0.01	0	N/A
hsa-miR-1248	1351-1357	7mer-1A	-0.08	77	-0.08	0.024	N/A
hsa-miR-6868-3p	1351-1358	8mer	-0.09	76	-0.09	0	N/A

hsa-miR-1237-3p	1351-1357	7mer-1A	-0.06	67	-0.06	0.024	N/A
hsa-miR-1252-5p	1364-1370	7mer-m8	-0.02	20	-0.02	0.024	N/A
hsa-miR-4533	1365-1371	7mer-m8	-0.06	62	-0.06	0	N/A
hsa-miR-6876-5p	1366-1373	8mer	-0.1	80	-0.1	0	N/A
hsa-miR-4476	1366-1373	8mer	-0.1	80	-0.1	0	N/A
hsa-miR-651-5p	1369-1375	7mer-1A	-0.02	39	-0.02	0.024	N/A
hsa-miR-944	1375-1381	7mer-m8	-0.02	56	-0.02	0.099	N/A
hsa-miR-539-5p	1379-1385	7mer-m8	-0.02	65	-0.02	0.099	N/A
hsa-miR-3925-5p	1381-1387	7mer-1A	-0.02	71	-0.02	0	N/A
hsa-miR-3123	1381-1387	7mer-1A	-0.03	70	-0.03	0	N/A
hsa-miR-6832-5p	1382-1389	8mer	-0.19	81	-0.19	0	N/A
hsa-miR-4732-5p	1383-1389	7mer-1A	-0.13	86	-0.13	0	N/A
hsa-miR-3674	1384-1390	7mer-m8	-0.03	25	-0.03	0	N/A
hsa-miR-141-5p	1393-1399	7mer-m8	-0.09	76	-0.09	0	N/A
hsa-miR-141-5p	1401-1407	7mer-m8	-0.09	76	-0.09	0	N/A
hsa-miR-5187-3p	1405-1411	7mer-m8	-0.06	78	-0.06	0	N/A
hsa-miR-4661-5p	1410-1416	7mer-m8	-0.09	59	-0.09	0	N/A
hsa-miR-708-3p	1412-1418	7mer-1A	-0.04	49	-0.04	0.085	N/A
hsa-miR-4674	1420-1426	7mer-m8	-0.11	78	-0.11	0	N/A
hsa-miR-4437	1420-1426	7mer-1A	-0.08	52	-0.08	0	N/A
hsa-miR-4417	1421-1427	7mer-m8	-0.03	40	-0.03	0	N/A
hsa-miR-654-5p	1422-1428	7mer-m8	-0.23	91	-0.23	0	N/A
hsa-miR-541-3p	1422-1428	7mer-m8	-0.21	89	-0.21	0	N/A
hsa-miR-3148	1430-1436	7mer-m8	-0.02	38	-0.02	0	N/A
hsa-miR-4496	1432-1438	7mer-1A	-0.01	64	-0.01	0	N/A

hsa-miR-8485	1442-1448	7mer-m8	-0.02	19	-0.02	0	N/A
hsa-miR-7151-3p	1451-1457	7mer-1A	-0.01	27	-0.01	0	N/A
hsa-miR-5095	1451-1457	7mer-1A	-0.01	26	-0.01	0	N/A
hsa-miR-1288-3p	1473-1479	7mer-m8	-0.19	85	-0.19	0.024	N/A
hsa-miR-3169	1474-1480	7mer-m8	-0.08	68	-0.08	0	N/A
hsa-miR-520d-5p	1479-1486	8mer	-0.03	80	-0.03	0	N/A
hsa-miR-524-5p	1479-1486	8mer	-0.03	78	-0.03	0	N/A
hsa-miR-452-5p	1485-1491	7mer-m8	-0.04	63	-0.04	0.811	N/A
hsa-miR-4676-3p	1485-1491	7mer-m8	-0.03	54	-0.03	0.811	N/A
hsa-miR-892c-3p	1485-1491	7mer-m8	-0.02	37	-0.02	0.811	N/A
hsa-miR-1208	1486-1492	7mer-m8	-0.11	87	-0.11	0.024	N/A
hsa-miR-141-3p	1488-1494	7mer-m8	-0.11	83	-0.11	1.291	＜ 0.1
hsa-miR-200a-3p	1488-1494	7mer-m8	-0.09	79	-0.09	1.291	＜ 0.1
hsa-miR-5192	1499-1505	7mer-m8	-0.19	93	-0.19	0	N/A
hsa-miR-6888-5p	1501-1507	7mer-1A	-0.01	34	-0.01	0	N/A
hsa-miR-4791	1512-1518	7mer-1A	-0.08	59	-0.08	0	N/A
hsa-miR-3201	1512-1518	7mer-1A	-0.01	26	-0.01	0	N/A
hsa-miR-376b-5p	1513-1519	7mer-m8	-0.09	60	-0.09	0	N/A
hsa-miR-376c-5p	1513-1519	7mer-m8	-0.04	50	-0.04	0	N/A
hsa-miR-3165	1514-1520	7mer-m8	-0.12	79	-0.12	0.024	N/A
hsa-miR-6745	1515-1521	7mer-m8	-0.17	81	-0.17	0	N/A
hsa-miR-363-5p	1515-1521	7mer-m8	-0.16	80	-0.16	0	N/A
hsa-miR-608	1516-1523	8mer	-0.32	89	-0.32	0	N/A

hsa-miR-4651	1516-1523	8mer	-0.3	88	-0.3	0	N/A
hsa-miR-6819-5p	1517-1523	7mer-m8	-0.19	82	-0.19	0	N/A
hsa-miR-6737-5p	1517-1523	7mer-m8	-0.18	81	-0.18	0	N/A
hsa-miR-6812-5p	1517-1523	7mer-m8	-0.18	79	-0.18	0	N/A
hsa-miR-342-5p	1517-1523	7mer-1A	-0.09	68	-0.09	0	N/A
hsa-miR-6747-5p	1517-1523	7mer-1A	-0.09	65	-0.09	0	N/A
hsa-miR-4664-5p	1517-1523	7mer-1A	-0.03	46	-0.03	0	N/A
hsa-miR-6890-5p	1518-1524	7mer-m8	-0.1	71	-0.1	0	N/A
hsa-miR-432-3p	1521-1528	8mer	-0.3	95	-0.3	0	N/A
hsa-miR-3161	1530-1536	7mer-1A	-0.01	39	-0.01	0	N/A
hsa-miR-599	1530-1536	7mer-m8	-0.02	23	-0.02	1.63	N/A
hsa-miR-502-5p	1534-1540	7mer-1A	-0.04	62	-0.04	0.099	N/A
hsa-miR-6875-3p	1537-1544	8mer	-0.06	75	-0.06	0	N/A
hsa-miR-4659b-3p	1538-1544	7mer-1A	-0.01	24	-0.01	0	N/A
hsa-miR-4659a-3p	1538-1544	7mer-1A	-0.01	24	-0.01	0	N/A
hsa-miR-4539	1547-1553	7mer-1A	-0.01	32	-0.01	0	N/A
hsa-miR-4762-3p	1549-1555	7mer-1A	-0.03	61	-0.03	0	N/A
hsa-miR-4419b	1562-1569	8mer	-0.09	93	-0.09	0	N/A
hsa-miR-3929	1562-1569	8mer	-0.08	92	-0.08	0	N/A
hsa-miR-4478	1562-1569	8mer	-0.07	89	-0.07	0	N/A
hsa-miR-6806-5p	1565-1571	7mer-1A	-0.16	55	-0.16	0	N/A
hsa-miR-1183	1567-1573	7mer-m8	-0.06	67	-0.06	0.099	N/A
hsa-miR-4803	1573-1579	7mer-m8	-0.02	41	-0.02	0.085	N/A
hsa-miR-4724-5p	1577-1583	7mer-m8	-0.09	88	-0.09	0	N/A
hsa-miR-6855-3p	1579-1585	7mer-1A	-0.11	78	-0.11	0	N/A

hsa-miR-4513	1579-1585	7mer-1A	-0.1	76	-0.1	0	N/A
hsa-miR-3136-5p	1579-1585	7mer-m8	-0.1	74	-0.1	0.024	N/A
hsa-miR-6857-3p	1579-1585	7mer-1A	-0.07	57	-0.07	0	N/A
hsa-miR-4439	1579-1585	7mer-m8	-0.02	44	-0.02	0.024	N/A
hsa-miR-134-5p	1580-1587	8mer	-0.24	92	-0.24	0.245	N/A
hsa-miR-3118	1580-1587	8mer	-0.24	91	-0.24	0.245	N/A
hsa-miR-6820-3p	1581-1587	7mer-1A	-0.05	52	-0.05	0	N/A
hsa-miR-3164	1581-1587	7mer-1A	-0.02	34	-0.02	0	N/A
hsa-miR-6818-5p	1583-1589	7mer-m8	-0.04	53	-0.04	0	N/A
hsa-miR-6867-5p	1584-1591	8mer	-0.13	57	-0.13	0	N/A
hsa-miR-6867-5p	1586-1593	8mer	-0.15	64	-0.15	0	N/A
hsa-miR-511-3p	1589-1595	7mer-1A	-0.05	56	-0.05	0.085	N/A
hsa-miR-297	1591-1598	8mer	-0.06	62	-0.06	0.085	N/A
hsa-miR-567	1591-1597	7mer-1A	-0.04	43	-0.04	0	N/A
hsa-miR-3149	1592-1599	8mer	-0.03	60	-0.03	0	N/A
hsa-miR-675-3p	1592-1598	7mer-1A	-0.02	48	-0.02	0.085	N/A
hsa-miR-450a-1-3p	1596-1607	non-canonical	N/A	N/A	N/A	0	N/A
hsa-miR-450a-1-3p	1596-1607	non-canonical	N/A	N/A	N/A	0	N/A
hsa-miR-548f-5p	1606-1612	7mer-m8	-0.05	75	-0.05	0	N/A
hsa-miR-548x-5p	1606-1612	7mer-m8	-0.04	70	-0.04	0	N/A
hsa-miR-548aj-5p	1606-1612	7mer-m8	-0.04	70	-0.04	0	N/A
hsa-miR-548g-5p	1606-1612	7mer-m8	-0.04	70	-0.04	0	N/A
hsa-miR-548p	1607-1613	7mer-m8	-0.02	40	-0.02	0.024	N/A

hsa-miR-548e-5p	1610-1617	8mer	-0.03	67	-0.03	0	N/A
hsa-miR-3613-3p	1624-1630	7mer-1A	-0.01	26	-0.01	0	N/A
hsa-miR-3192-5p	1631-1638	8mer	-0.09	78	-0.09	0.024	N/A
hsa-miR-6506-5p	1632-1638	7mer-1A	-0.09	87	-0.09	0	N/A
hsa-miR-204-3p	1632-1638	7mer-1A	-0.04	74	-0.04	0	N/A
hsa-miR-619-5p	1632-1638	7mer-1A	-0.02	73	-0.02	0	N/A
hsa-miR-4646-5p	1632-1638	7mer-1A	-0.05	62	-0.05	0	N/A
hsa-miR-4314	1632-1638	7mer-m8	-0.06	50	-0.06	0	N/A
hsa-miR-1298-3p	1633-1639	7mer-m8	-0.04	51	-0.04	0.085	N/A
hsa-miR-4513	1639-1645	7mer-1A	-0.1	75	-0.1	0	N/A
hsa-miR-6855-3p	1639-1645	7mer-1A	-0.04	51	-0.04	0	N/A
hsa-miR-4439	1639-1645	7mer-m8	-0.02	34	-0.02	0.024	N/A
hsa-miR-6857-3p	1639-1645	7mer-1A	-0.03	32	-0.03	0	N/A
hsa-miR-3136-5p	1639-1645	7mer-m8	-0.02	24	-0.02	0.024	N/A
hsa-miR-345-5p	1641-1647	7mer-1A	-0.01	44	-0.01	0.024	N/A
hsa-miR-301b-5p	1642-1648	7mer-m8	-0.02	68	-0.02	0	N/A
hsa-miR-301a-5p	1642-1648	7mer-m8	-0.02	68	-0.02	0	N/A
hsa-miR-760	1644-1650	7mer-m8	-0.1	62	-0.1	0.069	N/A
hsa-miR-4283	1646-1652	7mer-m8	-0.23	88	-0.23	0	N/A
hsa-miR-6813-5p	1647-1654	8mer	-0.2	88	-0.2	0	N/A
hsa-miR-6085	1647-1654	8mer	-0.2	86	-0.2	0	N/A
hsa-miR-6743-5p	1648-1654	7mer-1A	-0.18	82	-0.18	0	N/A
hsa-miR-4688	1648-1654	7mer-1A	-0.17	79	-0.17	0	N/A
hsa-miR-6789-5p	1648-1654	7mer-m8	-0.15	78	-0.15	0	N/A
hsa-miR-135b-3p	1650-1656	7mer-m8	-0.05	43	-0.05	0.085	N/A

hsa-miR-1537-5p	1653-1659	7mer-1A	-0.1	53	-0.1	0	N/A
hsa-miR-4718	1653-1659	7mer-1A	-0.01	17	-0.01	0	N/A
hsa-miR-4999-5p	1653-1659	7mer-1A	-0.01	10	-0.01	0	N/A
hsa-miR-3671	1672-1678	7mer-m8	-0.02	41	-0.02	0	N/A
hsa-miR-551b-5p	1675-1681	7mer-m8	-0.02	62	-0.02	0.085	N/A
hsa-miR-548c-3p	1677-1683	7mer-m8	-0.02	84	-0.02	0.024	N/A
hsa-miR-4524a-3p	1683-1689	7mer-1A	-0.05	55	-0.05	0.024	N/A
hsa-miR-3672	1684-1690	7mer-1A	-0.09	80	-0.09	0	N/A
hsa-miR-6864-3p	1684-1690	7mer-1A	-0.02	51	-0.02	0	N/A
hsa-miR-4461	1685-1691	7mer-m8	-0.02	29	-0.02	0	N/A
hsa-miR-335-3p	1690-1696	7mer-m8	-0.02	61	-0.02	0.085	N/A
hsa-miR-3646	1707-1713	7mer-m8	-0.02	48	-0.02	0	N/A
hsa-miR-5093	1709-1716	8mer	-0.06	71	-0.06	0	N/A
hsa-miR-5586-3p	1715-1721	7mer-1A	-0.08	70	-0.07	0	N/A
hsa-miR-503-3p	1733-1739	7mer-m8	-0.03	34	-0.03	0	N/A
hsa-miR-6782-5p	1736-1742	7mer-1A	-0.18	75	-0.17	0	N/A
hsa-miR-4278	1737-1743	7mer-1A	-0.18	68	-0.17	0	N/A
hsa-miR-6824-5p	1737-1743	7mer-1A	-0.13	65	-0.13	0	N/A
hsa-miR-6789-5p	1737-1743	7mer-1A	-0.05	50	-0.04	0	N/A
hsa-miR-1911-3p	1756-1763	8mer	-0.25	92	-0.23	0	N/A
hsa-miR-6753-5p	1757-1763	7mer-1A	-0.06	57	-0.06	0	N/A
hsa-miR-660-5p	1763-1769	7mer-1A	-0.03	38	-0.03	0.099	N/A
hsa-miR-5591-3p	1763-1769	7mer-m8	-0.02	28	-0.02	0	N/A
hsa-miR-485-3p	1767-1773	7mer-1A	-0.01	18	-0.01	0.099	N/A
hsa-miR-539-3p	1767-1773	7mer-1A	-0.02	12	-0.02	0.099	N/A

hsa-miR-3149	1776-1782	7mer-1A	-0.01	28	-0.01	0.024	N/A
hsa-miR-6844	1778-1784	7mer-m8	-0.06	74	-0.05	0	N/A
hsa-miR-4307	1790-1797	8mer	-0.03	66	-0.03	0	N/A
hsa-miR-211-3p	1798-1805	8mer	-0.16	94	-0.15	0	N/A
hsa-miR-6754-5p	1799-1805	7mer-1A	-0.14	83	-0.13	0	N/A
hsa-miR-4441	1799-1805	7mer-1A	-0.11	77	-0.1	0	N/A
hsa-miR-4270	1799-1805	7mer-1A	-0.03	46	-0.02	0	N/A
hsa-miR-1257	1815-1821	7mer-1A	-0.08	63	-0.08	0	N/A
hsa-miR-3611	1816-1822	7mer-1A	-0.08	77	-0.08	0	N/A
hsa-miR-4639-5p	1824-1830	7mer-1A	-0.06	67	-0.06	0	N/A
hsa-miR-3121-5p	1827-1834	8mer	-0.13	87	-0.13	0	N/A
hsa-miR-7856-5p	1855-1862	8mer	-0.03	76	-0.03	0	N/A
hsa-miR-4477b	1855-1861	7mer-1A	-0.08	65	-0.07	0	N/A
hsa-miR-1256	1873-1879	7mer-1A	-0.07	55	-0.07	0.024	N/A
hsa-miR-4720-5p	1874-1881	8mer	-0.09	77	-0.08	0	N/A
hsa-miR-5588-5p	1874-1881	8mer	-0.08	75	-0.08	0	N/A
hsa-miR-4799-3p	1874-1881	8mer	-0.08	75	-0.08	0	N/A
hsa-miR-6868-5p	1875-1881	7mer-1A	-0.13	78	-0.12	0	N/A
hsa-miR-3128	1875-1881	7mer-1A	-0.06	63	-0.06	0.437	N/A
hsa-miR-1231	1877-1883	7mer-m8	-0.02	51	-0.02	0	N/A
hsa-miR-3120-5p	1878-1884	7mer-m8	-0.02	47	-0.02	0	N/A
hsa-miR-5699-3p	1879-1886	8mer	-0.03	43	-0.03	0	N/A
hsa-miR-4421	1879-1886	8mer	-0.03	40	-0.03	0	N/A
hsa-miR-6748-3p	1880-1886	7mer-1A	-0.01	31	-0.01	0	N/A
hsa-miR-8075	1889-1895	7mer-m8	-0.02	26	-0.02	0	N/A

hsa-miR-3064-5p	1892-1898	7mer-m8	-0.05	60	-0.05	1.171	0.18
hsa-miR-6504-5p	1892-1898	7mer-m8	-0.03	47	-0.03	1.171	0.18
hsa-miR-1254	1895-1901	7mer-1A	-0.09	72	-0.08	0.024	N/A
hsa-miR-3116	1895-1901	7mer-1A	-0.05	58	-0.05	0.024	N/A
hsa-miR-661	1895-1901	7mer-1A	-0.02	26	-0.02	0.099	N/A
hsa-miR-550a-5p	1896-1902	7mer-m8	-0.02	17	-0.02	0.099	N/A
hsa-miR-550a-3-5p	1896-1902	7mer-m8	-0.02	17	-0.02	0.099	N/A
hsa-miR-1271-3p	1896-1902	7mer-m8	-0.02	16	-0.02	0.099	N/A
hsa-miR-515-3p	1897-1903	7mer-m8	-0.02	35	-0.02	0.024	N/A
hsa-miR-33b-3p	1897-1903	7mer-m8	-0.02	23	-0.02	0.024	N/A
hsa-miR-519e-3p	1897-1903	7mer-m8	-0.02	21	-0.02	0.024	N/A
hsa-miR-128-3p	1900-1907	8mer	-0.03	56	-0.03	1.013	<0.1
hsa-miR-216a-3p	1900-1907	8mer	-0.03	54	-0.03	1.013	<0.1
hsa-miR-3681-3p	1900-1907	8mer	-0.03	53	-0.03	1.013	<0.1
hsa-miR-27a-3p	1901-1907	7mer-m8	-0.02	47	-0.02	1.013	<0.1
hsa-miR-27b-3p	1901-1907	7mer-m8	-0.02	46	-0.02	1.013	<0.1
hsa-miR-342-3p	1903-1909	7mer-1A	-0.01	39	-0.01	0.379	N/A
hsa-miR-6737-3p	1909-1916	8mer	-0.03	49	-0.03	0.437	N/A

hsa-miR-7157-3p	1909-1916	8mer	-0.03	49	-0.03	0.437	N/A
hsa-miR-5008-3p	1909-1916	8mer	-0.03	49	-0.03	0.437	N/A
hsa-miR-6889-3p	1910-1916	7mer-1A	-0.01	31	-0.01	0.489	N/A
hsa-miR-6805-3p	1913-1919	7mer-m8	-0.02	38	-0.02	0	N/A
hsa-miR-5691	1913-1919	7mer-m8	-0.02	35	-0.02	0	N/A
hsa-miR-3194-3p	1913-1919	7mer-1A	-0.01	30	-0.01	0	N/A
hsa-miR-6752-3p	1917-1923	7mer-1A	-0.01	27	-0.01	0	N/A
hsa-miR-7113-3p	1918-1924	7mer-m8	-0.02	38	-0.02	0	N/A
hsa-miR-711	1927-1933	7mer-m8	-0.02	20	-0.02	0.099	N/A
hsa-miR-1286	1930-1936	7mer-m8	-0.02	48	-0.02	0.024	N/A
hsa-miR-6893-5p	1932-1938	7mer-m8	-0.02	43	-0.02	0.099	N/A
hsa-miR-6808-5p	1932-1938	7mer-m8	-0.02	43	-0.02	0.099	N/A
hsa-miR-940	1932-1938	7mer-m8	-0.02	43	-0.02	0.099	N/A
hsa-miR-4692	1933-1940	8mer	-0.03	54	-0.03	0	N/A
hsa-miR-4514	1933-1940	8mer	-0.03	54	-0.03	0	N/A
hsa-miR-4269	1934-1940	7mer-1A	-0.01	54	-0.01	0	N/A
hsa-miR-6715b-5p	1934-1940	7mer-1A	-0.01	45	-0.01	0	N/A
hsa-miR-4742-5p	1934-1940	7mer-1A	-0.01	25	-0.01	0.024	N/A
hsa-miR-4279	1939-1945	7mer-m8	-0.02	57	-0.02	0	N/A
hsa-miR-5088-3p	1950-1956	7mer-1A	-0.01	23	-0.01	0	N/A
hsa-miR-4287	1951-1957	7mer-m8	-0.02	47	-0.02	0	N/A
hsa-miR-4685-3p	1951-1957	7mer-m8	-0.02	45	-0.02	0	N/A
hsa-miR-6778-3p	1953-1959	7mer-m8	-0.02	32	-0.02	0	N/A
hsa-miR-6089	1957-1963	7mer-1A	-0.01	39	-0.01	0	N/A
hsa-miR-5008-5p	1957-1963	7mer-1A	-0.01	29	-0.01	0	N/A

hsa-miR-4316	1958-1965	8mer	-0.03	58	-0.03	0	N/A
hsa-miR-1294	1959-1965	7mer-1A	-0.01	17	-0.01	0.024	N/A
hsa-miR-4677-3p	1960-1966	7mer-m8	-0.02	40	-0.02	0.085	N/A
hsa-miR-1914-5p	1962-1968	7mer-1A	-0.02	56	-0.02	0	N/A
hsa-miR-660-3p	1964-1971	8mer	-0.03	43	-0.03	0.085	N/A
hsa-miR-5193	1965-1971	7mer-1A	-0.01	34	-0.01	0	N/A
hsa-miR-5196-3p	1967-1974	8mer	-0.03	59	-0.03	0	N/A
hsa-miR-4793-5p	1968-1974	7mer-1A	-0.01	27	-0.01	0	N/A
hsa-miR-631	1975-1981	7mer-m8	-0.05	39	-0.04	0.099	N/A
hsa-miR-3661	1975-1981	7mer-m8	-0.02	28	-0.02	0.099	N/A
hsa-miR-769-5p	1977-1984	8mer	-0.03	48	-0.03	0.085	N/A
hsa-miR-4786-5p	1978-1984	7mer-1A	-0.01	10	-0.01	0	N/A
hsa-miR-4329	1979-1985	7mer-m8	-0.02	58	-0.02	0	N/A
hsa-miR-6857-3p	1981-1988	8mer	-0.03	36	-0.03	0	N/A
hsa-miR-4439	1982-1988	7mer-m8	-0.02	34	-0.02	0.024	N/A
hsa-miR-6855-3p	1982-1988	7mer-1A	-0.01	26	-0.01	0	N/A
hsa-miR-4513	1982-1988	7mer-1A	-0.01	25	-0.01	0	N/A
hsa-miR-3136-5p	1982-1988	7mer-m8	-0.02	24	-0.02	0.024	N/A
hsa-miR-6825-3p	1985-1991	7mer-m8	-0.08	58	-0.07	0	N/A
hsa-miR-1306-5p	1993-1999	7mer-m8	-0.02	44	-0.02	0.436	< 0.1
hsa-miR-3692-3p	1997-2003	7mer-1A	-0.01	23	-0.01	0.085	N/A
hsa-miR-4325	2002-2008	7mer-m8	-0.02	44	-0.02	0	N/A
hsa-miR-4793-3p	2003-2009	7mer-m8	-0.02	36	-0.02	0	N/A
hsa-miR-6804-3p	2007-2014	8mer	-0.06	47	-0.05	0	N/A

hsa-miR-500a-3p	2008-2014	7mer-m8	-0.02	24	-0.02	0.085	N/A
hsa-miR-502-3p	2009-2015	7mer-m8	-0.02	34	-0.02	0.046	N/A
hsa-miR-501-3p	2009-2015	7mer-m8	-0.02	34	-0.02	0.046	N/A
hsa-miR-210-5p	2016-2023	8mer	-0.1	64	-0.1	0.085	N/A
hsa-miR-4749-3p	2017-2023	7mer-1A	-0.01	25	-0.01	0	N/A
hsa-miR-6823-5p	2023-2029	7mer-1A	-0.01	26	-0.01	0	N/A
hsa-miR-1238-3p	2031-2038	8mer	-0.03	61	-0.03	0	N/A
hsa-miR-670-3p	2032-2038	7mer-1A	-0.01	19	-0.01	0.07	N/A
hsa-miR-885-3p	2041-2047	7mer-1A	-0.05	57	-0.05	0.099	N/A
hsa-miR-6868-5p	2042-2049	8mer	-0.1	70	-0.09	0	N/A
hsa-miR-4720-5p	2043-2049	7mer-1A	-0.01	30	-0.01	0	N/A
hsa-miR-4799-3p	2043-2049	7mer-1A	-0.01	26	-0.01	0	N/A
hsa-miR-5588-5p	2043-2049	7mer-1A	-0.01	25	-0.01	0	N/A
hsa-miR-3128	2043-2049	7mer-1A	-0.01	19	-0.01	0	N/A
hsa-miR-6744-3p	2047-2053	7mer-m8	-0.02	50	-0.02	0	N/A
hsa-miR-4757-5p	2047-2053	7mer-m8	-0.02	34	-0.02	0	N/A
hsa-miR-650	2051-2057	7mer-1A	-0.08	67	-0.08	0.024	N/A
hsa-miR-3612	2051-2057	7mer-1A	-0.07	56	-0.07	0.024	N/A
hsa-miR-4443	2051-2057	7mer-m8	-0.03	47	-0.03	0	N/A
hsa-miR-7847-3p	2052-2058	7mer-m8	-0.03	32	-0.02	0	N/A
hsa-miR-4519	2056-2063	8mer	-0.03	45	-0.03	0	N/A
hsa-miR-4274	2057-2063	7mer-1A	-0.01	41	-0.01	0	N/A
hsa-miR-4524b-5p	2058-2064	7mer-1A	-0.05	50	-0.04	0	N/A
hsa-miR-374b-3p	2058-2065	8mer	-0.03	47	-0.03	0	N/A
hsa-miR-4524a-5p	2058-2064	7mer-1A	-0.02	34	-0.02	0	N/A

hsa-miR-5009-5p	2064-2070	7mer-m8	-0.04	48	-0.04	0	N/A
hsa-miR-8058	2064-2070	7mer-m8	-0.02	29	-0.02	0	N/A
hsa-miR-3138	2066-2072	7mer-1A	-0.12	57	-0.12	0	N/A
hsa-miR-4800-5p	2066-2072	7mer-1A	-0.08	54	-0.07	0	N/A
hsa-miR-4491	2067-2074	8mer	-0.03	29	-0.03	0	N/A
hsa-miR-4657	2067-2074	8mer	-0.03	28	-0.03	0	N/A
hsa-miR-299-3p	2068-2074	7mer-1A	-0.1	73	-0.09	0.067	N/A
hsa-miR-6790-3p	2080-2086	7mer-1A	-0.01	23	-0.01	0	N/A
hsa-miR-6821-3p	2080-2086	7mer-1A	-0.01	14	-0.01	0	N/A
hsa-miR-6855-3p	2083-2090	8mer	-0.03	46	-0.03	0	N/A
hsa-miR-4513	2083-2090	8mer	-0.03	46	-0.03	0	N/A
hsa-miR-4439	2084-2090	7mer-m8	-0.02	34	-0.02	0.024	N/A
hsa-miR-3136-5p	2084-2090	7mer-m8	-0.02	24	-0.02	0.024	N/A
hsa-miR-6857-3p	2084-2090	7mer-1A	-0.01	17	-0.01	0	N/A
hsa-miR-134-5p	2085-2091	7mer-m8	-0.02	30	-0.02	0.069	N/A
hsa-miR-3118	2085-2091	7mer-m8	-0.02	27	-0.02	0.069	N/A
hsa-miR-2909	2091-2097	7mer-1A	-0.01	34	-0.01	0	N/A
hsa-miR-657	2099-2105	7mer-m8	-0.02	51	-0.02	0.024	N/A
hsa-miR-6081	2102-2108	7mer-m8	-0.04	38	-0.04	0	N/A
hsa-miR-6133	2105-2111	7mer-m8	-0.19	83	-0.18	0.504	N/A
hsa-miR-6130	2105-2111	7mer-m8	-0.18	82	-0.17	0.504	N/A
hsa-miR-6129	2105-2111	7mer-m8	-0.16	77	-0.15	0.504	N/A
hsa-miR-4510	2105-2111	7mer-m8	-0.16	77	-0.15	0.504	N/A
hsa-miR-4419a	2105-2111	7mer-m8	-0.14	72	-0.13	0.504	N/A
hsa-miR-6127	2105-2111	7mer-m8	-0.14	71	-0.13	0.504	N/A

hsa-miR-6873-5p	2106-2113	8mer	-0.03	33	-0.03	0	N/A
hsa-miR-6866-5p	2108-2114	7mer-1A	-0.01	19	-0.01	0	N/A
hsa-miR-877-5p	2108-2114	7mer-1A	-0.01	14	-0.01	0.199	N/A
hsa-miR-331-3p	2116-2123	8mer	-0.03	54	-0.03	0.154	N/A
hsa-miR-6810-3p	2117-2123	7mer-1A	-0.01	29	-0.01	0	N/A
hsa-miR-6801-3p	2117-2123	7mer-1A	-0.01	27	-0.01	0	N/A
hsa-miR-6729-3p	2119-2126	8mer	-0.11	74	-0.1	0	N/A
hsa-miR-6870-3p	2121-2127	7mer-m8	-0.02	56	-0.02	0	N/A
hsa-miR-5695	2128-2134	7mer-m8	-0.02	38	-0.02	0	N/A
hsa-miR-6728-5p	2135-2141	7mer-m8	-0.02	50	-0.02	0	N/A
hsa-miR-5187-5p	2135-2141	7mer-1A	-0.06	45	-0.06	0	N/A
hsa-miR-4654	2136-2143	8mer	-0.07	58	-0.06	0.024	N/A
hsa-miR-4769-5p	2136-2143	8mer	-0.03	48	-0.03	0.024	N/A
hsa-miR-4648	2137-2143	7mer-1A	-0.01	23	-0.01	0	N/A
hsa-miR-1233-5p	2137-2143	7mer-1A	-0.02	21	-0.02	0	N/A
hsa-miR-6778-5p	2137-2143	7mer-1A	-0.02	20	-0.02	0	N/A
hsa-miR-6796-5p	2138-2144	7mer-1A	-0.07	56	-0.06	0	N/A
hsa-miR-4265	2138-2144	7mer-1A	-0.02	46	-0.02	0	N/A
hsa-miR-4296	2138-2144	7mer-1A	-0.03	44	-0.03	0	N/A
hsa-miR-4322	2138-2144	7mer-1A	-0.01	35	-0.01	0	N/A
hsa-miR-6759-5p	2138-2144	7mer-1A	-0.01	19	-0.01	0	N/A
hsa-miR-8059	2144-2150	7mer-m8	-0.07	68	-0.06	0	N/A
hsa-miR-4471	2144-2150	7mer-m8	-0.1	67	-0.09	0	N/A
hsa-miR-6822-5p	2145-2152	8mer	-0.1	85	-0.09	0	N/A
hsa-miR-6750-5p	2145-2152	8mer	-0.09	84	-0.08	0	N/A

hsa-miR-5584-5p	2146-2152	7mer-1A	-0.04	54	-0.03	0	N/A
hsa-miR-9500	2146-2152	7mer-1A	-0.01	32	-0.01	0	N/A
hsa-miR-3162-5p	2147-2153	7mer-1A	-0.08	51	-0.07	0	N/A
hsa-miR-2909	2148-2154	7mer-1A	-0.01	34	-0.01	0	N/A
hsa-miR-891b	2154-2161	8mer	-0.03	36	-0.03	0.099	N/A
hsa-miR-4772-3p	2156-2162	7mer-m8	-0.02	54	-0.02	0	N/A
hsa-miR-6829-5p	2159-2165	7mer-m8	-0.17	86	-0.16	0	N/A
hsa-miR-185-3p	2160-2166	7mer-m8	-0.11	49	-0.1	0	N/A
hsa-miR-6846-5p	2161-2168	8mer	-0.12	56	-0.12	0	N/A
hsa-miR-6848-5p	2161-2168	8mer	-0.12	55	-0.12	0	N/A
hsa-miR-4697-5p	2162-2168	7mer-1A	-0.16	66	-0.15	0	N/A
hsa-miR-1237-5p	2162-2168	7mer-1A	-0.12	60	-0.11	0	N/A
hsa-miR-4488	2162-2168	7mer-1A	-0.11	57	-0.1	0	N/A
hsa-miR-637	2163-2169	7mer-m8	-0.02	34	-0.02	0.024	N/A
hsa-miR-6852-5p	2164-2170	7mer-m8	-0.02	44	-0.02	0	N/A
hsa-miR-4292	2165-2171	7mer-m8	-0.02	30	-0.02	0	N/A
hsa-miR-6791-5p	2165-2171	7mer-m8	-0.02	29	-0.02	0	N/A
hsa-miR-331-3p	2166-2172	7mer-m8	-0.02	40	-0.02	0.058	N/A
hsa-miR-605-5p	2171-2177	7mer-m8	-0.02	58	-0.02	0.099	N/A
hsa-miR-4668-3p	2172-2178	7mer-m8	-0.02	74	-0.02	0	N/A
hsa-miR-8063	2173-2180	8mer	-0.03	86	-0.03	0	N/A
hsa-miR-4698	2174-2180	7mer-1A	-0.01	42	-0.01	0	N/A
hsa-miR-373-5p	2175-2181	7mer-m8	-0.02	39	-0.02	0.024	N/A
hsa-miR-371b-5p	2175-2181	7mer-m8	-0.02	38	-0.02	0.024	N/A
hsa-miR-616-5p	2175-2181	7mer-m8	-0.02	34	-0.02	0.024	N/A

hsa-miR-6854-5p	2178-2184	7mer-1A	-0.01	32	-0.01	0	N/A
hsa-miR-7705	2179-2185	7mer-m8	-0.06	67	-0.06	0	N/A
hsa-miR-3682-3p	2183-2189	7mer-m8	-0.02	17	-0.02	0	N/A
hsa-miR-6088	2185-2191	7mer-m8	-0.02	31	-0.02	0.245	＜0.1
hsa-miR-4770	2185-2191	7mer-m8	-0.02	29	-0.02	0.245	＜0.1
hsa-miR-143-3p	2185-2191	7mer-m8	-0.02	27	-0.02	0.245	＜0.1
hsa-miR-4756-3p	2187-2193	7mer-m8	-0.02	52	-0.02	0	N/A
hsa-miR-3678-3p	2189-2195	7mer-m8	-0.05	61	-0.04	0	N/A
hsa-miR-2467-3p	2189-2195	7mer-1A	-0.06	59	-0.06	0	N/A
hsa-miR-3944-5p	2191-2197	7mer-1A	-0.05	67	-0.05	0	N/A
hsa-miR-143-5p	2191-2197	7mer-1A	-0.02	42	-0.02	0	N/A
hsa-miR-1304-3p	2203-2209	7mer-1A	-0.01	26	-0.01	0.437	N/A
hsa-miR-1229-3p	2204-2210	7mer-1A	-0.02	44	-0.01	0	N/A
hsa-miR-942-5p	2206-2212	7mer-1A	-0.01	26	-0.01	0.024	N/A
hsa-miR-6740-3p	2207-2213	7mer-m8	-0.03	39	-0.03	0	N/A
hsa-miR-409-3p	2218-2224	7mer-m8	-0.02	71	-0.02	1.639	N/A
hsa-miR-203a-3p.1	2220-2226	7mer-m8	-0.02	50	-0.02	1.905	0.23
hsa-miR-5680	2221-2228	8mer	-0.03	87	-0.03	0	N/A
hsa-miR-10b-3p	2230-2237	8mer	-0.06	76	-0.06	0	N/A
hsa-miR-4330	2232-2239	8mer	-0.07	81	-0.07	0	N/A
hsa-miR-216a-5p	2235-2241	7mer-m8	-0.02	27	-0.02	0.88	＜

								0.1
hsa-miR-4668-3p	2238-2244	7mer-1A	-0.01	56	-0.01	0	N/A
hsa-miR-3163	2239-2246	8mer	-0.03	86	-0.03	0	N/A
hsa-miR-338-5p	2244-2250	7mer-m8	-0.02	51	-0.02	0	N/A
hsa-miR-888-3p	2248-2254	7mer-m8	-0.02	29	-0.02	0	N/A
hsa-miR-140-5p	2256-2262	7mer-m8	-0.13	80	-0.13	1.813	0.27
hsa-miR-4796-3p	2258-2264	7mer-m8	-0.03	74	-0.03	0	N/A
hsa-miR-1257	2272-2278	7mer-m8	-0.04	43	-0.04	0	N/A
hsa-miR-7854-3p	2275-2282	8mer	-0.14	80	-0.13	0	N/A
hsa-miR-6134	2276-2282	7mer-1A	-0.08	57	-0.08	0.024	N/A
hsa-miR-7162-3p	2277-2283	7mer-m8	-0.02	63	-0.02	0	N/A
hsa-miR-544b	2278-2285	8mer	-0.12	80	-0.12	0	N/A
hsa-miR-4324	2279-2285	7mer-1A	-0.05	63	-0.05	0	N/A
hsa-miR-1200	2279-2285	7mer-m8	-0.04	60	-0.04	0	N/A
hsa-miR-4666b	2285-2291	7mer-m8	-0.02	36	-0.02	0	N/A
hsa-miR-20a-3p	2287-2293	7mer-1A	-0.09	73	-0.08	0	N/A
hsa-miR-544a	2288-2295	8mer	-0.03	37	-0.03	0.024	N/A
hsa-miR-6738-3p	2289-2295	7mer-1A	-0.01	32	-0.01	0	N/A
hsa-miR-4513	2300-2306	7mer-1A	-0.05	60	-0.05	0	N/A
hsa-miR-6855-3p	2300-2306	7mer-1A	-0.04	53	-0.04	0	N/A
hsa-miR-6857-3p	2300-2306	7mer-1A	-0.03	39	-0.03	0	N/A
hsa-miR-4439	2300-2306	7mer-m8	-0.02	34	-0.02	0.024	N/A
hsa-miR-3136-5p	2300-2306	7mer-m8	-0.02	24	-0.02	0.024	N/A
hsa-miR-345-5p	2302-2308	7mer-1A	-0.01	44	-0.01	0.099	N/A

hsa-miR-4256	2303-2309	7mer-1A	-0.13	78	-0.12	0	N/A
hsa-miR-6874-3p	2305-2311	7mer-m8	-0.02	45	-0.02	0	N/A
hsa-miR-148b-5p	2305-2311	7mer-m8	-0.02	42	-0.02	0	N/A
hsa-miR-4277	2306-2312	7mer-m8	-0.02	45	-0.02	0	N/A
hsa-miR-7977	2310-2316	7mer-1A	-0.01	33	-0.01	0	N/A
hsa-miR-4652-3p	2316-2322	7mer-m8	-0.02	58	-0.02	0	N/A
hsa-miR-3182	2317-2323	7mer-m8	-0.02	59	-0.02	0.312	N/A
hsa-miR-1237-3p	2318-2325	8mer	-0.11	82	-0.1	0.024	N/A
hsa-miR-6868-3p	2319-2325	7mer-m8	-0.02	38	-0.02	0	N/A
hsa-miR-1248	2319-2325	7mer-1A	-0.02	37	-0.02	0.024	N/A
hsa-miR-922	2333-2339	7mer-m8	-0.02	52	-0.02	0.099	N/A
hsa-miR-2277-3p	2336-2342	7mer-1A	-0.02	33	-0.02	0	N/A
hsa-miR-542-3p	2337-2343	7mer-m8	-0.02	30	-0.02	1.28	N/A
hsa-miR-4789-3p	2345-2351	7mer-m8	-0.02	40	-0.02	0	N/A
hsa-miR-642b-3p	2346-2353	8mer	-0.03	47	-0.03	0	N/A
hsa-miR-642a-3p	2346-2353	8mer	-0.03	46	-0.03	0	N/A
hsa-miR-514a-3p	2348-2354	7mer-m8	-0.06	70	-0.05	0.099	N/A
hsa-miR-514b-3p	2348-2354	7mer-m8	-0.06	70	-0.05	0.099	N/A
hsa-miR-526b-5p	2351-2357	7mer-1A	-0.01	34	-0.01	0.099	N/A
hsa-miR-103a-2-5p	2353-2360	8mer	-0.03	45	-0.03	0.085	N/A
hsa-miR-4686	2357-2363	7mer-m8	-0.02	35	-0.02	0	N/A
hsa-miR-452-3p	2359-2365	7mer-m8	-0.02	40	-0.02	0	N/A
hsa-miR-6072	2368-2374	7mer-m8	-0.03	52	-0.03	0	N/A
hsa-miR-6891-3p	2368-2374	7mer-m8	-0.02	45	-0.02	0	N/A

hsa-miR-6747-3p	2372-2378	7mer-m8	-0.05	61	-0.05	0	N/A
hsa-miR-631	2375-2381	7mer-1A	-0.04	37	-0.04	0.024	N/A
hsa-miR-3661	2375-2381	7mer-1A	-0.03	33	-0.03	0.024	N/A
hsa-miR-3680-5p	2388-2394	7mer-m8	-0.02	35	-0.02	0	N/A
hsa-miR-134-5p	2392-2398	7mer-1A	-0.1	67	-0.1	0.206	N/A
hsa-miR-3118	2392-2398	7mer-1A	-0.1	67	-0.1	0.206	N/A
hsa-miR-6820-3p	2392-2398	7mer-1A	-0.02	28	-0.02	0	N/A
hsa-miR-3164	2392-2398	7mer-1A	-0.01	18	-0.01	0	N/A
hsa-miR-4501	2393-2399	7mer-m8	-0.09	82	-0.08	0	N/A
hsa-miR-8066	2394-2400	7mer-m8	-0.02	34	-0.02	0	N/A
hsa-miR-187-5p	2399-2406	8mer	-0.04	52	-0.04	0.085	N/A
hsa-miR-222-3p	2400-2406	7mer-1A	-0.11	70	-0.1	2.697	0.2
hsa-miR-221-3p	2400-2406	7mer-1A	-0.1	67	-0.09	2.697	0.2
hsa-miR-548v	2400-2406	7mer-1A	-0.09	58	-0.08	0	N/A
hsa-miR-155-5p	2403-2410	8mer	-0.14	80	-0.14	0.154	< 0.1
hsa-miR-28-3p	2413-2419	7mer-1A	-0.11	81	-0.11	0.492	N/A
hsa-miR-8084	2415-2421	7mer-m8	-0.02	56	-0.02	0	N/A
hsa-miR-1283	2422-2428	7mer-m8	-0.07	66	-0.07	0.024	N/A
hsa-miR-6715b-3p	2428-2434	7mer-1A	-0.01	45	-0.01	0	N/A
hsa-miR-607	2429-2435	7mer-1A	-0.01	40	-0.01	0	N/A
hsa-miR-4676-3p	2433-2440	8mer	-0.04	62	-0.04	0.069	N/A
hsa-miR-892c-3p	2433-2440	8mer	-0.03	54	-0.03	0.069	N/A
hsa-miR-452-5p	2433-2440	8mer	-0.03	52	-0.03	0.069	N/A
hsa-miR-4693-5p	2435-2441	7mer-1A	-0.1	70	-0.09	0	N/A

hsa-miR-4477a	2442-2449	8mer	-0.03	75	-0.03	0	N/A
hsa-miR-5691	2451-2457	7mer-1A	-0.05	60	-0.05	0	N/A
hsa-miR-6805-3p	2451-2457	7mer-1A	-0.01	34	-0.01	0	N/A
hsa-miR-6885-3p	2453-2459	7mer-1A	-0.01	32	-0.01	0	N/A
hsa-miR-3140-3p	2455-2461	7mer-m8	-0.02	42	-0.02	0	N/A
hsa-miR-208a-5p	2456-2463	8mer	-0.03	78	-0.03	0	N/A
hsa-miR-208b-5p	2456-2463	8mer	-0.03	59	-0.03	0	N/A
hsa-miR-6878-5p	2465-2476	non-canonical	N/A	N/A	N/A	0	N/A
hsa-miR-6878-5p	2465-2476	non-canonical	N/A	N/A	N/A	0	N/A
hsa-miR-3185	2474-2481	8mer	-0.11	86	-0.09	0	N/A
hsa-miR-4517	2479-2485	7mer-m8	-0.02	39	-0.02	0	N/A
hsa-miR-6124	2490-2496	7mer-1A	-0.01	49	-0.01	0	N/A
hsa-miR-3148	2490-2496	7mer-1A	-0.01	25	-0.01	0	N/A
hsa-miR-3165	2493-2499	7mer-1A	-0.13	80	-0.11	0.024	N/A
hsa-miR-8071	2493-2499	7mer-1A	-0.1	77	-0.08	0	N/A
hsa-miR-6880-5p	2493-2499	7mer-1A	-0.11	63	-0.1	0	N/A
hsa-miR-584-5p	2496-2502	7mer-1A	-0.01	16	-0.01	0.099	N/A
hsa-miR-4766-3p	2510-2516	7mer-1A	-0.05	59	-0.04	0	N/A
hsa-miR-374b-3p	2511-2517	7mer-1A	-0.06	59	-0.05	0	N/A
hsa-miR-32-3p	2513-2519	7mer-m8	-0.02	73	-0.02	0	N/A
hsa-miR-633	2520-2526	7mer-1A	-0.01	28	-0.01	0	N/A
hsa-miR-4282	2525-2531	7mer-1A	-0.01	49	-0.01	0	N/A
hsa-miR-302a-5p	2528-2534	7mer-1A	-0.01	50	-0.01	0	N/A

hsa-miR-6828-5p	2538-2544	7mer-m8	-0.02	19	-0.02	0	N/A
hsa-miR-4747-5p	2540-2546	7mer-1A	-0.06	53	-0.05	0	N/A
hsa-miR-5196-5p	2540-2546	7mer-1A	-0.02	31	-0.02	0	N/A
hsa-miR-450a-1-3p	2541-2548	8mer	-0.03	41	-0.03	0.085	N/A
hsa-miR-3122	2542-2548	7mer-1A	-0.01	41	-0.01	0.085	N/A
hsa-miR-887-5p	2542-2548	7mer-1A	-0.02	35	-0.02	0	N/A
hsa-miR-3913-5p	2542-2548	7mer-1A	-0.01	35	-0.01	0.085	N/A
hsa-miR-6513-5p	2542-2548	7mer-1A	-0.01	25	-0.01	0	N/A
hsa-miR-146b-5p	2551-2558	8mer	-0.05	54	-0.04	0.221	< 0.1
hsa-miR-146a-5p	2551-2558	8mer	-0.05	54	-0.04	0.221	< 0.1
hsa-miR-7153-5p	2551-2558	8mer	-0.05	54	-0.04	0.221	< 0.1
hsa-miR-589-5p	2552-2558	7mer-1A	-0.01	30	-0.01	0.099	N/A
hsa-miR-573	2557-2563	7mer-m8	-0.04	60	-0.03	0.085	N/A
hsa-miR-3616-5p	2557-2563	7mer-m8	-0.04	59	-0.04	0.085	N/A
hsa-miR-4795-5p	2557-2563	7mer-1A	-0.03	47	-0.03	0	N/A
hsa-miR-1245b-3p	2561-2567	7mer-m8	-0.02	30	-0.02	0	N/A
hsa-miR-5683	2562-2568	7mer-m8	-0.02	42	-0.02	0	N/A
hsa-miR-4520-3p	2565-2571	7mer-m8	-0.04	53	-0.04	0	N/A
hsa-miR-4638-3p	2566-2572	7mer-m8	-0.02	18	-0.02	0	N/A
hsa-miR-3690	2567-2573	7mer-m8	-0.02	52	-0.02	0	N/A
hsa-miR-4308	2568-2575	8mer	-0.03	43	-0.03	0	N/A

hsa-miR-4292	2569-2575	7mer-1A	-0.01	25	-0.01	0	N/A
hsa-miR-6791-5p	2569-2575	7mer-1A	-0.01	24	-0.01	0	N/A
hsa-miR-7113-3p	2570-2576	7mer-m8	-0.02	38	-0.02	0	N/A
hsa-miR-6778-3p	2572-2579	8mer	-0.03	43	-0.03	0	N/A
hsa-miR-6836-3p	2573-2579	7mer-m8	-0.02	38	-0.02	0	N/A
hsa-miR-6791-3p	2573-2579	7mer-1A	-0.01	26	-0.01	0	N/A
hsa-miR-6829-3p	2573-2579	7mer-1A	-0.01	25	-0.01	0	N/A
hsa-miR-550b-2-5p	2575-2581	7mer-m8	-0.02	29	-0.02	0	N/A
hsa-miR-550a-3-5p	2575-2581	7mer-1A	-0.01	12	-0.01	0.099	N/A
hsa-miR-550a-5p	2575-2581	7mer-1A	-0.01	12	-0.01	0.099	N/A
hsa-miR-1271-3p	2575-2581	7mer-1A	-0.01	11	-0.01	0.099	N/A
hsa-miR-6889-3p	2576-2582	7mer-m8	-0.02	42	-0.02	0.085	N/A
hsa-miR-1324	2585-2591	7mer-m8	-0.02	41	-0.02	0.024	N/A
hsa-miR-6815-3p	2594-2600	7mer-m8	-0.02	30	-0.02	0	N/A
hsa-miR-1251-3p	2601-2607	7mer-m8	-0.02	51	-0.02	0.489	N/A
hsa-miR-3158-3p	2604-2610	7mer-m8	-0.05	57	-0.05	0	N/A
hsa-miR-3975	2611-2617	7mer-m8	-0.06	68	-0.05	0	N/A
hsa-miR-4709-3p	2615-2621	7mer-m8	-0.02	47	-0.02	0	N/A
hsa-miR-27a-3p	2633-2639	7mer-m8	-0.02	47	-0.02	0	< 0.1
hsa-miR-27b-3p	2633-2639	7mer-m8	-0.02	46	-0.02	0	< 0.1
hsa-miR-128-3p	2633-2639	7mer-1A	-0.01	31	-0.01	0	<

							0.1
hsa-miR-216a-3p	2633-2639	7mer-1A	-0.01	30	-0.01	0	<0.1
hsa-miR-3681-3p	2633-2639	7mer-1A	-0.01	28	-0.01	0	<0.1
hsa-miR-6884-3p	2636-2642	7mer-m8	-0.02	50	-0.02	0	N/A
hsa-miR-4641	2639-2645	7mer-1A	-0.01	12	-0.01	0	N/A
hsa-miR-3661	2644-2650	7mer-1A	-0.01	15	-0.01	0.024	N/A
hsa-miR-631	2644-2650	7mer-1A	-0.01	13	-0.01	0.024	N/A
hsa-miR-378a-5p	2647-2653	7mer-m8	-0.02	54	-0.02	0	N/A
hsa-miR-3653-5p	2648-2655	8mer	-0.03	61	-0.03	0	N/A
hsa-miR-1976	2649-2655	7mer-1A	-0.01	47	-0.01	0	N/A
hsa-miR-6845-3p	2650-2656	7mer-m8	-0.02	36	-0.02	0	N/A
hsa-miR-7111-3p	2652-2658	7mer-m8	-0.02	48	-0.02	0	N/A
hsa-miR-3183	2652-2658	7mer-1A	-0.01	36	-0.01	0	N/A
hsa-miR-4723-3p	2652-2658	7mer-1A	-0.01	28	-0.01	0	N/A
hsa-miR-6769b-3p	2652-2658	7mer-1A	-0.01	28	-0.01	0	N/A
hsa-miR-4268	2654-2660	7mer-m8	-0.02	49	-0.02	0	N/A
hsa-miR-6736-3p	2656-2662	7mer-m8	-0.04	61	-0.03	0	N/A
hsa-miR-4660	2657-2663	7mer-m8	-0.02	39	-0.02	0.024	N/A
hsa-miR-6721-5p	2660-2666	7mer-1A	-0.01	21	-0.01	0	N/A
hsa-miR-365b-5p	2667-2674	8mer	-0.05	50	-0.05	0	N/A
hsa-miR-365a-5p	2667-2674	8mer	-0.04	40	-0.03	0	N/A
hsa-miR-8052	2668-2674	7mer-1A	-0.09	58	-0.08	0	N/A
hsa-miR-3199	2668-2674	7mer-1A	-0.05	38	-0.04	0	N/A

hsa-miR-5694	2668-2679	non-canonical	N/A	N/A	N/A	0	N/A
hsa-miR-5694	2668-2679	non-canonical	N/A	N/A	N/A	0	N/A
hsa-miR-2681-3p	2672-2678	7mer-m8	-0.05	65	-0.04	0	N/A
hsa-miR-6800-5p	2681-2688	8mer	-0.03	27	-0.03	0	N/A
hsa-miR-6802-5p	2682-2688	7mer-1A	-0.12	63	-0.1	0	N/A
hsa-miR-1258	2684-2690	7mer-1A	-0.16	68	-0.13	0.099	N/A
hsa-miR-4687-3p	2687-2693	7mer-m8	-0.04	53	-0.04	0	N/A
hsa-miR-4741	2688-2694	7mer-m8	-0.11	79	-0.09	0	N/A
hsa-miR-4675	2688-2694	7mer-m8	-0.06	77	-0.05	0	N/A
hsa-miR-4446-3p	2689-2695	7mer-m8	-0.13	76	-0.11	0.099	N/A
hsa-miR-3127-5p	2691-2697	7mer-m8	-0.16	81	-0.14	0.024	N/A
hsa-miR-3918	2691-2697	7mer-1A	-0.01	34	-0.01	0	N/A
hsa-miR-4772-5p	2693-2700	8mer	-0.03	32	-0.03	0	N/A
hsa-miR-383-5p.1	2694-2700	7mer-1A	-0.02	15	-0.02	0.032	＜0.1
hsa-miR-4513	2698-2704	7mer-1A	-0.06	62	-0.05	0	N/A
hsa-miR-6855-3p	2698-2704	7mer-1A	-0.03	48	-0.03	0	N/A
hsa-miR-4439	2698-2704	7mer-m8	-0.02	34	-0.02	0	N/A
hsa-miR-3136-5p	2698-2704	7mer-m8	-0.02	24	-0.02	0	N/A
hsa-miR-6857-3p	2698-2704	7mer-1A	-0.01	17	-0.01	0	N/A
hsa-miR-888-3p	2701-2707	7mer-1A	-0.01	21	-0.01	0	N/A
hsa-miR-1278	2703-2709	7mer-m8	-0.03	41	-0.03	0.024	N/A
hsa-miR-1911-5p	2705-2711	7mer-1A	-0.04	34	-0.03	0	N/A

hsa-miR-6869-5p	2706-2712	7mer-1A	-0.01	38	-0.01	0	N/A
hsa-miR-8081	2707-2713	7mer-1A	-0.01	32	-0.01	0	N/A
hsa-miR-1289	2718-2725	8mer	-0.11	69	-0.09	0.024	N/A
hsa-miR-4309	2719-2725	7mer-m8	-0.08	75	-0.07	0	N/A
hsa-miR-3198	2719-2725	7mer-m8	-0.02	66	-0.02	0	N/A
hsa-miR-4294	2719-2725	7mer-1A	-0.03	46	-0.03	0	N/A
hsa-miR-6514-5p	2720-2726	7mer-m8	-0.02	34	-0.02	0	N/A
hsa-miR-4534	2721-2727	7mer-m8	-0.02	38	-0.02	0	N/A
hsa-miR-8082	2721-2727	7mer-m8	-0.02	26	-0.02	0	N/A
hsa-miR-6757-5p	2724-2730	7mer-m8	-0.04	42	-0.03	0	N/A
hsa-miR-6754-5p	2726-2732	7mer-1A	-0.03	53	-0.03	0	N/A
hsa-miR-4441	2726-2732	7mer-1A	-0.04	53	-0.03	0	N/A
hsa-miR-211-3p	2726-2732	7mer-1A	-0.01	49	-0.01	0	N/A
hsa-miR-4270	2726-2732	7mer-1A	-0.01	31	-0.01	0	N/A
hsa-miR-3127-5p	2727-2733	7mer-1A	-0.05	52	-0.04	0.024	N/A
hsa-miR-7-5p	2735-2741	7mer-m8	-0.02	56	-0.02	0.245	< 0.1
hsa-miR-873-5p.1	2738-2744	7mer-1A	-0.01	56	-0.01	0.556	N/A
hsa-miR-4705	2742-2748	7mer-1A	-0.01	33	-0.01	0	N/A
hsa-miR-4755-3p	2751-2757	7mer-m8	-0.16	79	-0.14	0	N/A
hsa-miR-4753-5p	2754-2761	8mer	-0.03	34	-0.03	0	N/A
hsa-miR-506-3p	2756-2762	7mer-1A	-0.01	13	-0.01	0.221	< 0.1
hsa-miR-124-3p.2	2756-2762	7mer-1A	-0.01	8	-0.01	0.221	< 0.1

hsa-miR-4477a	2758-2764	7mer-m8	-0.02	64	-0.02	0	N/A
hsa-miR-503-3p	2761-2767	7mer-m8	-0.02	26	-0.02	0	N/A
hsa-miR-6755-5p	2763-2769	7mer-1A	-0.04	36	-0.04	0	N/A
hsa-miR-6841-5p	2763-2769	7mer-1A	-0.03	26	-0.02	0	N/A
hsa-miR-135b-3p	2765-2771	7mer-m8	-0.12	65	-0.1	0	N/A
hsa-miR-3137	2767-2773	7mer-1A	-0.01	39	-0.01	0	N/A
hsa-miR-20b-3p	2767-2773	7mer-1A	-0.01	20	-0.01	0	N/A
hsa-miR-3686	2768-2774	7mer-1A	-0.03	35	-0.02	0	N/A
hsa-miR-4743-3p	2769-2775	7mer-1A	-0.01	39	-0.01	0	N/A
hsa-miR-4652-3p	2769-2775	7mer-1A	-0.01	36	-0.01	0	N/A
hsa-miR-3160-5p	2771-2777	7mer-m8	-0.02	41	-0.02	0	N/A
hsa-miR-4438	2775-2781	7mer-m8	-0.02	37	-0.02	0.312	N/A
hsa-miR-4520-3p	2778-2784	7mer-m8	-0.04	50	-0.03	0	N/A
hsa-miR-3928-3p	2796-2802	7mer-1A	-0.01	50	-0.01	0	N/A
hsa-miR-3915	2796-2802	7mer-m8	-0.02	20	-0.02	0	N/A
hsa-miR-6857-3p	2799-2806	8mer	-0.03	36	-0.03	0	N/A
hsa-miR-4439	2800-2806	7mer-m8	-0.02	34	-0.02	0.024	N/A
hsa-miR-6855-3p	2800-2806	7mer-1A	-0.01	26	-0.01	0	N/A
hsa-miR-4513	2800-2806	7mer-1A	-0.01	25	-0.01	0	N/A
hsa-miR-3136-5p	2800-2806	7mer-m8	-0.02	24	-0.02	0.024	N/A
hsa-miR-873-5p.1	2807-2813	7mer-m8	-0.02	74	-0.02	1.017	N/A
hsa-miR-665	2809-2815	7mer-1A	-0.09	71	-0.08	0.899	N/A
hsa-miR-3974	2817-2823	7mer-1A	-0.08	60	-0.07	0.085	N/A
hsa-miR-555	2820-2826	7mer-m8	-0.02	15	-0.02	0.024	N/A
hsa-miR-6881-5p	2821-2828	8mer	-0.03	6	-0.03	0	N/A

hsa-miR-6890-5p	2823-2829	7mer-m8	-0.03	32	-0.03	0	N/A
hsa-miR-7704	2830-2836	7mer-1A	-0.08	56	-0.07	0	N/A
hsa-miR-615-5p	2830-2836	7mer-1A	-0.02	34	-0.02	0.085	N/A
hsa-miR-7155-5p	2831-2837	7mer-m8	-0.07	71	-0.06	0	N/A
hsa-miR-4731-5p	2832-2838	7mer-m8	-0.14	81	-0.12	0	N/A
hsa-miR-320e	2834-2840	7mer-m8	-0.02	37	-0.02	0	N/A
hsa-miR-127-5p	2836-2842	7mer-m8	-0.02	35	-0.02	0	N/A
hsa-miR-3928-5p	2836-2842	7mer-1A	-0.01	34	-0.01	0.085	N/A
hsa-miR-6806-3p	2836-2842	7mer-1A	-0.01	33	-0.01	0.085	N/A
hsa-miR-6821-3p	2861-2867	7mer-m8	-0.03	40	-0.03	0	N/A
hsa-miR-6868-3p	2868-2874	7mer-1A	-0.04	58	-0.03	0	N/A
hsa-miR-3124-3p	2870-2876	7mer-m8	-0.02	37	-0.02	0	N/A
hsa-miR-4448	2878-2884	7mer-m8	-0.02	45	-0.02	0	N/A
hsa-miR-6736-3p	2880-2887	8mer	-0.09	77	-0.08	0	N/A
hsa-miR-6787-3p	2882-2889	8mer	-0.03	64	-0.03	0	N/A
hsa-miR-3670	2888-2894	7mer-1A	-0.12	66	-0.1	0.085	N/A
hsa-miR-8066	2892-2898	7mer-m8	-0.02	34	-0.02	0	N/A
hsa-miR-6770-3p	2906-2912	7mer-m8	-0.17	72	-0.14	0	N/A
hsa-miR-4467	2906-2912	7mer-1A	-0.17	68	-0.14	0	N/A
hsa-miR-4696	2927-2933	7mer-1A	-0.01	30	-0.01	0	N/A
hsa-miR-1322	2931-2937	7mer-m8	-0.03	70	-0.02	0.024	N/A
hsa-miR-4770	2934-2940	7mer-m8	-0.03	38	-0.02	0.229	< 0.1
hsa-miR-6088	2934-2940	7mer-m8	-0.02	31	-0.02	0.229	< 0.1

hsa-miR-143-3p	2934-2940	7mer-m8	-0.02	27	-0.02	0.229	< 0.1
hsa-miR-1303	2937-2943	7mer-m8	-0.02	23	-0.02	0.024	N/A
hsa-miR-4448	2942-2948	7mer-m8	-0.02	45	-0.02	0	N/A
hsa-miR-3139	2946-2952	7mer-m8	-0.07	64	-0.06	0.482	N/A
hsa-miR-28-5p	2946-2952	7mer-m8	-0.02	27	-0.02	0.482	N/A
hsa-miR-708-5p	2946-2952	7mer-m8	-0.02	27	-0.02	0.482	N/A
hsa-miR-877-5p	2949-2956	8mer	-0.03	37	-0.03	0.069	N/A
hsa-miR-6859-5p	2949-2955	7mer-1A	-0.01	31	-0.01	0	N/A
hsa-miR-3125	2949-2955	7mer-1A	-0.01	21	-0.01	0	N/A
hsa-miR-3916	2949-2955	7mer-1A	-0.01	17	-0.01	0	N/A
hsa-miR-6866-5p	2950-2956	7mer-1A	-0.09	69	-0.08	0	N/A
hsa-miR-3622b-5p	2962-2968	7mer-m8	-0.03	41	-0.03	0	N/A
hsa-miR-6862-5p	2963-2969	7mer-m8	-0.05	38	-0.05	0	N/A
hsa-miR-4253	2963-2969	7mer-m8	-0.02	16	-0.02	0	N/A
hsa-miR-6819-5p	2972-2978	7mer-m8	-0.1	64	-0.09	0	N/A
hsa-miR-6737-5p	2972-2978	7mer-m8	-0.09	62	-0.08	0	N/A
hsa-miR-6812-5p	2972-2978	7mer-m8	-0.09	62	-0.08	0	N/A
hsa-miR-342-5p	2972-2978	7mer-1A	-0.04	45	-0.03	0.085	N/A
hsa-miR-4651	2972-2978	7mer-1A	-0.08	40	-0.06	0	N/A
hsa-miR-608	2972-2978	7mer-1A	-0.08	39	-0.07	0	N/A
hsa-miR-6747-5p	2972-2978	7mer-1A	-0.03	26	-0.02	0	N/A
hsa-miR-4664-5p	2972-2978	7mer-1A	-0.01	25	-0.01	0.085	N/A
hsa-miR-6890-5p	2973-2979	7mer-m8	-0.02	21	-0.02	0	N/A
hsa-miR-3151-3p	2983-2990	8mer	-0.03	47	-0.03	0	N/A

hsa-miR-3192-3p	2984-2990	7mer-m8	-0.02	58	-0.02	0	N/A
hsa-miR-301a-5p	2986-2992	7mer-1A	-0.01	48	-0.01	0	N/A
hsa-miR-301b-5p	2986-2992	7mer-1A	-0.01	48	-0.01	0	N/A
hsa-miR-3921	2986-2992	7mer-1A	-0.01	30	-0.01	0	N/A
hsa-miR-4653-5p	2986-2992	7mer-1A	-0.01	30	-0.01	0	N/A
hsa-miR-146a-3p	2986-2992	7mer-1A	-0.01	22	-0.01	0	N/A
hsa-miR-3191-5p	2987-2993	7mer-1A	-0.01	34	-0.01	0	N/A
hsa-miR-942-5p	2988-2994	7mer-m8	-0.02	38	-0.02	0.024	N/A
hsa-miR-5088-3p	2990-2997	8mer	-0.03	49	-0.03	0	N/A
hsa-miR-6866-3p	2992-2998	7mer-m8	-0.02	43	-0.02	0.221	N/A
hsa-miR-188-5p	2992-2998	7mer-m8	-0.02	31	-0.02	0.221	N/A
hsa-miR-6729-3p	2994-3000	7mer-1A	-0.07	66	-0.06	0	N/A
hsa-miR-6870-3p	2995-3001	7mer-m8	-0.02	56	-0.02	0	N/A
hsa-miR-4432	2999-3005	7mer-m8	-0.02	52	-0.02	0	N/A
hsa-miR-5197-3p	3002-3008	7mer-1A	-0.01	39	-0.01	0	N/A
hsa-miR-3976	3011-3017	7mer-1A	-0.12	72	-0.1	0	N/A
hsa-miR-378d	3027-3033	7mer-m8	-0.02	27	-0.02	0.705	N/A
hsa-miR-378i	3027-3033	7mer-m8	-0.02	27	-0.02	0.705	N/A
hsa-miR-378a-3p	3027-3033	7mer-m8	-0.02	27	-0.02	0.705	N/A
hsa-miR-378h	3027-3033	7mer-m8	-0.02	27	-0.02	0.705	N/A
hsa-miR-422a	3027-3033	7mer-m8	-0.02	27	-0.02	0.705	N/A
hsa-miR-378b	3027-3033	7mer-m8	-0.02	27	-0.02	0.705	N/A
hsa-miR-378e	3027-3033	7mer-m8	-0.02	27	-0.02	0.705	N/A
hsa-miR-378c	3027-3033	7mer-m8	-0.02	27	-0.02	0.705	N/A
hsa-miR-378f	3027-3033	7mer-m8	-0.02	27	-0.02	0.705	N/A

hsa-miR-3690	3028-3034	7mer-m8	-0.1	81	-0.08	0	N/A
hsa-miR-619-3p	3029-3035	7mer-m8	-0.02	34	-0.02	0.024	N/A
hsa-miR-490-3p	3030-3036	7mer-m8	-0.02	37	-0.02	1.196	< 0.1
hsa-miR-7106-3p	3043-3049	7mer-1A	-0.11	66	-0.09	0	N/A
hsa-miR-128-3p	3048-3054	7mer-m8	-0.02	40	-0.02	0.678	< 0.1
hsa-miR-216a-3p	3048-3054	7mer-m8	-0.02	38	-0.02	0.678	< 0.1
hsa-miR-3681-3p	3048-3054	7mer-m8	-0.02	36	-0.02	0.678	< 0.1
hsa-miR-140-3p.1	3050-3056	7mer-1A	-0.01	33	-0.01	0.506	< 0.1
hsa-miR-324-5p	3054-3060	7mer-1A	-0.02	29	-0.02	0.069	N/A
hsa-miR-1265	3059-3065	7mer-1A	-0.01	33	-0.01	0.024	N/A
hsa-miR-2392	3059-3065	7mer-1A	-0.01	31	-0.01	0.024	N/A
hsa-miR-6826-5p	3062-3068	7mer-m8	-0.02	35	-0.02	0	N/A
hsa-miR-4295	3066-3072	7mer-m8	-0.02	39	-0.02	0.154	< 0.1
hsa-miR-130a-3p	3066-3072	7mer-m8	-0.02	38	-0.02	0.154	< 0.1
hsa-miR-301b-3p	3066-3072	7mer-m8	-0.02	37	-0.02	0.154	< 0.1
hsa-miR-301a-3p	3066-3072	7mer-m8	-0.02	37	-0.02	0.154	< 0.1
hsa-miR-130b-3p	3066-3072	7mer-m8	-0.02	37	-0.02	0.154	<

							0.1
hsa-miR-3666	3066-3072	7mer-m8	-0.02	36	-0.02	0.154	< 0.1
hsa-miR-454-3p	3066-3072	7mer-m8	-0.02	35	-0.02	0.154	< 0.1
hsa-miR-504-3p	3068-3075	8mer	-0.03	52	-0.03	0	N/A
hsa-miR-122-5p	3069-3075	7mer-1A	-0.01	18	-0.01	0.22	< 0.1
hsa-miR-3135b	3071-3077	7mer-m8	-0.02	32	-0.02	0	N/A
hsa-miR-1207-3p	3073-3080	8mer	-0.03	56	-0.03	0.024	N/A
hsa-miR-9-5p	3085-3091	7mer-m8	-0.02	55	-0.02	0.069	< 0.1
hsa-miR-100-3p	3089-3095	7mer-1A	-0.01	37	-0.01	0	N/A
hsa-miR-545-3p	3100-3107	8mer	-0.03	48	-0.03	0.099	N/A
hsa-miR-4291	3102-3108	7mer-1A	-0.02	40	-0.02	0	N/A
hsa-miR-4511	3109-3115	7mer-m8	-0.02	56	-0.02	0	N/A
hsa-miR-5003-5p	3118-3125	8mer	-0.03	35	-0.03	0	N/A
hsa-miR-224-5p	3122-3129	8mer	-0.03	69	-0.03	0.126	N/A
hsa-miR-4483	3129-3135	7mer-m8	-0.02	48	-0.02	0.024	N/A
hsa-miR-1293	3129-3135	7mer-m8	-0.02	26	-0.02	0.024	N/A
hsa-miR-4651	3130-3136	7mer-m8	-0.11	48	-0.09	0.437	N/A
hsa-miR-608	3130-3136	7mer-m8	-0.09	42	-0.08	0.437	N/A
hsa-miR-6782-5p	3131-3137	7mer-m8	-0.09	59	-0.08	0	N/A
hsa-miR-6722-3p	3133-3139	7mer-m8	-0.13	72	-0.11	0	N/A
hsa-miR-1909-3p	3133-3139	7mer-m8	-0.11	68	-0.1	0	N/A

hsa-miR-4763-3p	3134-3140	7mer-m8	-0.11	71	-0.09	0	N/A
hsa-miR-1207-5p	3134-3140	7mer-m8	-0.1	71	-0.09	0	N/A
hsa-miR-6808-5p	3135-3141	7mer-m8	-0.02	43	-0.02	0.024	N/A
hsa-miR-940	3135-3141	7mer-m8	-0.02	43	-0.02	0.024	N/A
hsa-miR-6893-5p	3135-3141	7mer-m8	-0.02	43	-0.02	0.024	N/A
hsa-miR-1827	3136-3143	8mer	-0.12	70	-0.1	0.024	N/A
hsa-miR-4316	3138-3145	8mer	-0.03	58	-0.03	0	N/A
hsa-miR-1294	3139-3145	7mer-1A	-0.01	17	-0.01	0.024	N/A
hsa-miR-17-3p	3146-3153	8mer	-0.03	62	-0.03	0.402	N/A
hsa-miR-3158-5p	3147-3153	7mer-1A	-0.11	85	-0.09	0.085	N/A
hsa-miR-1205	3147-3153	7mer-1A	-0.03	59	-0.02	0.024	N/A
hsa-miR-1184	3147-3153	7mer-1A	-0.01	44	-0.01	0.099	N/A
hsa-miR-632	3149-3155	7mer-m8	-0.09	86	-0.08	0.099	N/A
hsa-miR-4288	3149-3155	7mer-m8	-0.13	85	-0.11	0.099	N/A
hsa-miR-346	3149-3155	7mer-1A	-0.04	38	-0.03	1.967	N/A
hsa-miR-654-3p	3150-3157	8mer	-0.03	50	-0.03	0.099	N/A
hsa-miR-6756-3p	3157-3163	7mer-1A	-0.06	60	-0.05	0	N/A
hsa-miR-3127-3p	3157-3163	7mer-1A	-0.03	45	-0.02	0	N/A
hsa-miR-5588-3p	3160-3166	7mer-1A	-0.09	67	-0.08	0	N/A
hsa-miR-2114-5p	3160-3166	7mer-1A	-0.01	17	-0.01	0	N/A
hsa-miR-4639-5p	3174-3180	7mer-m8	-0.02	40	-0.02	0	N/A
hsa-miR-6893-3p	3177-3183	7mer-1A	-0.12	75	-0.1	0.154	N/A
hsa-miR-370-3p	3177-3183	7mer-1A	-0.06	71	-0.05	0.154	N/A
hsa-miR-3074-5p	3178-3185	8mer	-0.03	77	-0.03	0	N/A
hsa-miR-7152-5p	3179-3185	7mer-1A	-0.02	53	-0.02	0	N/A

hsa-miR-3124-3p	3180-3186	7mer-m8	-0.02	37	-0.02	0	N/A
hsa-miR-515-3p	3185-3191	7mer-m8	-0.02	35	-0.02	0.024	N/A
hsa-miR-33b-3p	3185-3191	7mer-m8	-0.02	30	-0.02	0.024	N/A
hsa-miR-519e-3p	3185-3191	7mer-m8	-0.02	21	-0.02	0.024	N/A
hsa-miR-1825	3187-3193	7mer-m8	-0.09	78	-0.07	0.024	N/A
hsa-miR-589-5p	3192-3199	8mer	-0.03	56	-0.03	0.099	N/A
hsa-miR-146b-5p	3193-3199	7mer-1A	-0.01	18	-0.01	1.269	0.1 2
hsa-miR-7153-5p	3193-3199	7mer-1A	-0.01	18	-0.01	1.269	0.1 2
hsa-miR-146a-5p	3193-3199	7mer-1A	-0.01	18	-0.01	1.269	0.1 2
hsa-miR-580-3p	3194-3200	7mer-1A	-0.01	36	-0.01	0.024	N/A
hsa-miR-4780	3197-3203	7mer-m8	-0.02	19	-0.02	0	N/A
hsa-miR-1249-3p	3199-3205	7mer-m8	-0.07	25	-0.06	0.058	N/A
hsa-miR-4632-3p	3201-3207	7mer-m8	-0.19	64	-0.16	0	N/A
hsa-miR-6746-3p	3202-3208	7mer-m8	-0.1	55	-0.09	0	N/A
hsa-miR-4632-5p	3207-3213	7mer-m8	-0.02	40	-0.02	0.335	N/A
hsa-miR-7843-5p	3207-3213	7mer-m8	-0.02	39	-0.02	0.335	N/A
hsa-miR-6879-5p	3207-3213	7mer-m8	-0.02	31	-0.02	0.335	N/A
hsa-miR-4436b-3p	3207-3213	7mer-m8	-0.02	30	-0.02	0.335	N/A
hsa-miR-6735-5p	3207-3213	7mer-m8	-0.02	30	-0.02	0.335	N/A
hsa-miR-6814-5p	3211-3218	8mer	-0.09	79	-0.08	0	N/A
hsa-miR-4699-5p	3218-3224	7mer-m8	-0.02	35	-0.02	0	N/A
hsa-miR-3649	3226-3232	7mer-1A	-0.06	45	-0.05	0	N/A

hsa-miR-3122	3227-3233	7mer-m8	-0.02	77	-0.02	0.085	N/A
hsa-miR-4298	3227-3233	7mer-1A	-0.11	71	-0.09	0.024	N/A
hsa-miR-3913-5p	3227-3233	7mer-m8	-0.02	68	-0.02	0.085	N/A
hsa-miR-1302	3227-3233	7mer-1A	-0.09	66	-0.08	0.024	N/A
hsa-miR-4425	3228-3234	7mer-m8	-0.02	6	-0.02	0	N/A
hsa-miR-92a-1-5p	3229-3236	8mer	-0.13	60	-0.11	0	N/A
hsa-miR-29b-1-5p	3232-3238	7mer-1A	-0.01	30	-0.01	0	N/A
hsa-miR-6830-3p	3237-3243	7mer-m8	-0.02	54	-0.02	0	N/A
hsa-miR-449b-3p	3244-3250	7mer-m8	-0.02	30	-0.02	0	N/A
hsa-miR-551b-5p	3250-3256	7mer-m8	-0.02	62	-0.02	0	N/A
hsa-miR-539-5p	3255-3261	7mer-1A	-0.01	46	-0.01	0.099	N/A
hsa-miR-4251	3256-3262	7mer-m8	-0.02	36	-0.02	0	N/A
hsa-miR-4324	3257-3263	7mer-m8	-0.02	35	-0.02	0	N/A
hsa-miR-3622b-3p	3259-3265	7mer-m8	-0.02	38	-0.02	0	N/A
hsa-miR-3622a-3p	3259-3265	7mer-m8	-0.02	34	-0.02	0	N/A
hsa-miR-6804-3p	3260-3267	8mer	-0.03	33	-0.03	0	N/A
hsa-miR-500a-3p	3261-3267	7mer-m8	-0.02	24	-0.02	0.085	N/A
hsa-miR-3130-3p	3262-3268	7mer-m8	-0.02	61	-0.02	0	N/A
hsa-miR-6829-5p	3265-3272	8mer	-0.03	48	-0.03	0	N/A
hsa-miR-5001-5p	3266-3272	7mer-1A	-0.06	44	-0.05	0.099	N/A
hsa-miR-3620-5p	3266-3272	7mer-m8	-0.02	40	-0.02	0	N/A
hsa-miR-4675	3266-3272	7mer-1A	-0.01	34	-0.01	0	N/A
hsa-miR-4498	3266-3272	7mer-1A	-0.03	32	-0.03	0.099	N/A
hsa-miR-1587	3266-3272	7mer-m8	-0.02	30	-0.02	0	N/A
hsa-miR-4492	3266-3272	7mer-1A	-0.01	29	-0.01	0.099	N/A

hsa-miR-762	3266-3272	7mer-1A	-0.01	27	-0.01	0.099	N/A
hsa-miR-4741	3266-3272	7mer-1A	-0.01	22	-0.01	0	N/A
hsa-miR-4656	3266-3272	7mer-1A	-0.01	9	-0.01	0	N/A
hsa-miR-378g	3267-3273	7mer-m8	-0.02	39	-0.02	0	N/A
hsa-miR-5787	3269-3275	7mer-m8	-0.02	41	-0.02	0	N/A
hsa-miR-4505	3269-3275	7mer-m8	-0.02	25	-0.02	0	N/A
hsa-miR-6768-3p	3280-3286	7mer-m8	-0.02	52	-0.02	0	N/A
hsa-miR-4766-5p	3285-3291	7mer-1A	-0.01	43	-0.01	0.489	N/A
hsa-miR-146a-3p	3286-3292	7mer-m8	-0.02	30	-0.02	0	N/A
hsa-miR-6805-3p	3288-3294	7mer-m8	-0.02	38	-0.02	0	N/A
hsa-miR-5691	3288-3294	7mer-m8	-0.02	35	-0.02	0	N/A
hsa-miR-3194-3p	3288-3294	7mer-1A	-0.01	30	-0.01	0	N/A
hsa-miR-3907	3290-3296	7mer-m8	-0.12	72	-0.1	0	N/A
hsa-miR-342-5p	3292-3298	7mer-m8	-0.13	77	-0.11	0.085	N/A
hsa-miR-4664-5p	3292-3298	7mer-m8	-0.11	77	-0.09	0.085	N/A
hsa-miR-6842-5p	3293-3299	7mer-m8	-0.14	48	-0.12	0	N/A
hsa-miR-7110-5p	3293-3299	7mer-m8	-0.12	42	-0.1	0	N/A
hsa-miR-6752-5p	3293-3299	7mer-m8	-0.06	33	-0.05	0	N/A
hsa-miR-6732-5p	3294-3300	7mer-m8	-0.06	41	-0.05	0	N/A
hsa-miR-6798-5p	3295-3301	7mer-m8	-0.07	63	-0.06	0.085	N/A
hsa-miR-6753-5p	3297-3303	7mer-m8	-0.06	59	-0.05	0	N/A
hsa-miR-106a-3p	3305-3311	7mer-1A	-0.01	32	-0.01	0	N/A
hsa-miR-4684-3p	3306-3313	8mer	-0.03	33	-0.03	0	N/A
hsa-miR-409-3p	3309-3315	7mer-m8	-0.02	71	-0.02	0.154	N/A
hsa-miR-6508-5p	3324-3331	8mer	-0.03	46	-0.03	0	N/A

hsa-miR-8067	3324-3331	8mer	-0.03	46	-0.03	0	N/A
hsa-miR-519c-5p	3326-3332	7mer-1A	-0.01	32	-0.01	0.024	N/A
hsa-miR-522-5p	3326-3332	7mer-1A	-0.01	32	-0.01	0.024	N/A
hsa-miR-523-5p	3326-3332	7mer-1A	-0.01	32	-0.01	0.024	N/A
hsa-miR-519a-5p	3326-3332	7mer-1A	-0.01	32	-0.01	0.024	N/A
hsa-miR-518e-5p	3326-3332	7mer-1A	-0.01	32	-0.01	0.024	N/A
hsa-miR-519b-5p	3326-3332	7mer-1A	-0.01	32	-0.01	0.024	N/A
hsa-miR-518d-5p	3326-3332	7mer-1A	-0.01	30	-0.01	0.024	N/A
hsa-miR-518f-5p	3326-3332	7mer-1A	-0.01	30	-0.01	0.024	N/A
hsa-miR-526a	3326-3332	7mer-1A	-0.01	30	-0.01	0.024	N/A
hsa-miR-520c-5p	3326-3332	7mer-1A	-0.01	30	-0.01	0.024	N/A
hsa-miR-4777-5p	3326-3332	7mer-1A	-0.01	27	-0.01	0	N/A
hsa-miR-4802-5p	3326-3337	non-canonical	N/A	N/A	N/A	0	N/A
hsa-miR-4802-5p	3326-3337	non-canonical	N/A	N/A	N/A	0	N/A
hsa-miR-182-3p	3328-3334	7mer-m8	-0.02	19	-0.02	0	N/A
hsa-miR-4257	3332-3338	7mer-m8	-0.02	48	-0.02	0	N/A
hsa-miR-3922-5p	3332-3343	non-canonical	N/A	N/A	N/A	0	N/A
hsa-miR-3922-5p	3332-3343	non-canonical	N/A	N/A	N/A	0	N/A
hsa-miR-4717-5p	3337-3343	7mer-1A	-0.01	39	-0.01	0	N/A
hsa-miR-15a-3p	3337-3343	7mer-1A	-0.01	28	-0.01	0	N/A
hsa-miR-5704	3337-3344	8mer	-0.03	7	-0.03	0	N/A

hsa-miR-1295a	3338-3344	7mer-1A	-0.12	68	-0.1	0.024	N/A
hsa-miR-3667-3p	3345-3351	7mer-m8	-0.02	34	-0.02	0	N/A
hsa-miR-6886-3p	3347-3353	7mer-m8	-0.02	35	-0.02	0	N/A
hsa-miR-6851-3p	3348-3354	7mer-m8	-0.02	28	-0.02	0	N/A
hsa-miR-1911-3p	3352-3358	7mer-m8	-0.02	33	-0.02	0	N/A
hsa-miR-4733-3p	3353-3359	7mer-m8	-0.02	39	-0.02	0	N/A
hsa-miR-1260b	3356-3362	7mer-m8	-0.02	33	-0.02	0	N/A
hsa-miR-1260a	3356-3362	7mer-m8	-0.02	33	-0.02	0	N/A
hsa-miR-1224-3p	3356-3362	7mer-1A	-0.01	30	-0.01	0	N/A
hsa-miR-5697	3364-3370	7mer-m8	-0.02	38	-0.02	0	N/A
hsa-miR-4480	3366-3373	8mer	-0.03	50	-0.03	0	N/A
hsa-miR-1909-5p	3370-3376	7mer-m8	-0.06	64	-0.05	0	N/A
hsa-miR-361-3p	3378-3384	7mer-m8	-0.02	27	-0.02	0.099	N/A
hsa-miR-874-5p	3382-3388	7mer-1A	-0.01	5	-0.01	0.085	N/A
hsa-miR-663b	3383-3389	7mer-m8	-0.02	42	-0.02	0.024	N/A
hsa-miR-4651	3386-3392	7mer-m8	-0.02	18	-0.02	0	N/A
hsa-miR-608	3386-3392	7mer-m8	-0.02	15	-0.02	0	N/A
hsa-miR-6752-5p	3387-3393	7mer-m8	-0.02	16	-0.02	0	N/A
hsa-miR-7110-5p	3387-3393	7mer-m8	-0.02	10	-0.02	0	N/A
hsa-miR-6842-5p	3387-3393	7mer-m8	-0.02	9	-0.02	0	N/A
hsa-miR-7109-5p	3389-3395	7mer-m8	-0.02	30	-0.02	0	N/A
hsa-miR-6885-5p	3389-3395	7mer-1A	-0.05	24	-0.04	0	N/A
hsa-miR-328-5p	3389-3395	7mer-1A	-0.01	14	-0.01	0	N/A
hsa-miR-6887-5p	3389-3395	7mer-1A	-0.01	1	-0.01	0	N/A
hsa-miR-6795-5p	3389-3395	7mer-1A	-0.01	1	-0.01	0	N/A

hsa-miR-637	3390-3396	7mer-m8	-0.02	34	-0.02	0.024	N/A
hsa-miR-183-5p.2	3395-3401	7mer-m8	-0.02	27	-0.02	0.154	＜0.1
hsa-miR-891a-3p	3396-3402	7mer-m8	-0.05	57	-0.04	0	N/A
hsa-miR-4693-3p	3400-3407	8mer	-0.03	29	-0.03	0	N/A
hsa-miR-3174	3404-3410	7mer-1A	-0.01	35	-0.01	0	N/A
hsa-miR-921	3404-3410	7mer-m8	-0.02	23	-0.02	0	N/A
hsa-miR-6847-5p	3413-3419	7mer-1A	-0.07	68	-0.06	0	N/A
hsa-miR-4257	3413-3419	7mer-1A	-0.01	37	-0.01	0	N/A
hsa-miR-4295	3420-3426	7mer-m8	-0.02	39	-0.02	0.623	＜0.1
hsa-miR-130a-3p	3420-3426	7mer-m8	-0.02	38	-0.02	0.623	＜0.1
hsa-miR-301b-3p	3420-3426	7mer-m8	-0.02	37	-0.02	0.623	＜0.1
hsa-miR-130b-3p	3420-3426	7mer-m8	-0.02	37	-0.02	0.623	＜0.1
hsa-miR-301a-3p	3420-3426	7mer-m8	-0.02	37	-0.02	0.623	＜0.1
hsa-miR-3666	3420-3426	7mer-m8	-0.02	36	-0.02	0.623	＜0.1
hsa-miR-454-3p	3420-3426	7mer-m8	-0.02	35	-0.02	0.623	＜0.1
hsa-miR-519c-3p	3421-3427	7mer-m8	-0.02	25	-0.02	0.571	N/A
hsa-miR-519a-3p	3421-3427	7mer-m8	-0.02	25	-0.02	0.571	N/A
hsa-miR-519b-3p	3421-3427	7mer-m8	-0.02	25	-0.02	0.571	N/A

hsa-miR-125b-2-3p	3424-3430	7mer-m8	-0.02	49	-0.02	0.442	N/A
hsa-miR-4731-3p	3425-3432	8mer	-0.03	70	-0.03	0	N/A
hsa-miR-4801	3425-3432	8mer	-0.03	66	-0.03	0	N/A
hsa-miR-155-3p	3429-3435	7mer-m8	-0.02	13	-0.02	0	N/A
hsa-miR-4793-5p	3432-3438	7mer-1A	-0.03	53	-0.03	0	N/A
hsa-miR-5196-3p	3432-3438	7mer-1A	-0.01	38	-0.01	0	N/A
hsa-miR-1226-3p	3440-3446	7mer-m8	-0.02	51	-0.02	0.024	N/A
hsa-miR-500b-3p	3445-3451	7mer-1A	-0.01	37	-0.01	0	N/A
hsa-miR-6884-3p	3448-3454	7mer-m8	-0.02	50	-0.02	0	N/A
hsa-miR-4682	3456-3462	7mer-m8	-0.02	44	-0.02	0	N/A
hsa-miR-8077	3457-3464	8mer	-0.03	73	-0.03	0	N/A
hsa-miR-4663	3458-3464	7mer-1A	-0.01	41	-0.01	0	N/A
hsa-miR-7160-5p	3458-3464	7mer-1A	-0.01	23	-0.01	0	N/A
hsa-miR-6851-5p	3463-3469	7mer-m8	-0.07	50	-0.06	0	N/A
hsa-miR-3689d	3463-3469	7mer-m8	-0.02	35	-0.02	0	N/A
hsa-miR-6799-5p	3464-3470	7mer-m8	-0.02	53	-0.02	0	N/A
hsa-miR-6825-5p	3465-3471	7mer-m8	-0.08	44	-0.07	0	N/A
hsa-miR-6738-5p	3468-3474	7mer-1A	-0.08	65	-0.07	0	N/A
hsa-miR-6734-5p	3468-3474	7mer-m8	-0.06	55	-0.05	0	N/A
hsa-miR-5194	3468-3474	7mer-1A	-0.03	35	-0.03	0	N/A
hsa-miR-1914-3p	3468-3474	7mer-1A	-0.01	34	-0.01	0	N/A
hsa-miR-423-5p	3468-3474	7mer-1A	-0.01	22	-0.01	0.814	N/A
hsa-miR-3184-5p	3468-3474	7mer-1A	-0.01	22	-0.01	0.814	N/A
hsa-miR-544b	3470-3476	7mer-m8	-0.02	33	-0.02	0	N/A

hsa-miR-5001-3p	3474-3481	8mer	-0.03	43	-0.03	0	N/A
hsa-miR-4727-5p	3475-3481	7mer-1A	-0.08	61	-0.06	0	N/A
hsa-miR-6738-3p	3475-3482	8mer	-0.03	52	-0.03	0	N/A
hsa-miR-544a	3476-3482	7mer-1A	-0.05	51	-0.04	0	N/A
hsa-miR-627-3p	3478-3485	8mer	-0.03	57	-0.03	0	N/A
hsa-miR-1245b-3p	3487-3493	7mer-m8	-0.02	30	-0.02	0	N/A
hsa-miR-5683	3488-3494	7mer-m8	-0.02	42	-0.02	0	N/A
hsa-miR-140-3p.1	3491-3497	7mer-1A	-0.01	33	-0.01	0.154	< 0.1
hsa-miR-3928-5p	3496-3503	8mer	-0.03	59	-0.03	0.085	N/A
hsa-miR-6806-3p	3496-3503	8mer	-0.03	58	-0.03	0.085	N/A
hsa-miR-127-5p	3497-3504	8mer	-0.03	49	-0.03	0	N/A
hsa-miR-7843-3p	3498-3504	7mer-1A	-0.01	30	-0.01	0	N/A
hsa-miR-1248	3505-3511	7mer-m8	-0.02	39	-0.02	0.024	N/A
hsa-miR-5088-3p	3506-3512	7mer-m8	-0.02	35	-0.02	0	N/A
hsa-miR-3127-3p	3507-3513	7mer-m8	-0.06	60	-0.05	0	N/A
hsa-miR-6756-3p	3507-3513	7mer-m8	-0.06	59	-0.05	0	N/A
hsa-miR-4645-5p	3513-3519	7mer-1A	-0.01	25	-0.01	0	N/A
hsa-miR-4673	3513-3519	7mer-1A	-0.01	19	-0.01	0	N/A
hsa-miR-508-5p	3515-3521	7mer-m8	-0.02	33	-0.02	0	N/A
hsa-miR-3678-3p	3522-3528	7mer-m8	-0.02	38	-0.02	0	N/A
hsa-miR-2467-3p	3522-3528	7mer-1A	-0.01	29	-0.01	0	N/A
hsa-miR-3158-5p	3523-3530	8mer	-0.03	57	-0.03	0.085	N/A
hsa-miR-1184	3524-3530	7mer-1A	-0.01	44	-0.01	0.099	N/A
hsa-miR-1205	3524-3530	7mer-1A	-0.01	39	-0.01	0.024	N/A

hsa-miR-17-3p	3524-3530	7mer-1A	-0.01	35	-0.01	0.099	N/A
hsa-miR-4527	3526-3533	8mer	-0.11	77	-0.09	0	N/A
hsa-miR-6503-5p	3526-3533	8mer	-0.1	72	-0.08	0	N/A
hsa-miR-7107-3p	3527-3533	7mer-1A	-0.06	60	-0.05	0	N/A
hsa-miR-6753-3p	3527-3533	7mer-1A	-0.04	43	-0.03	0	N/A
hsa-miR-6848-3p	3528-3534	7mer-1A	-0.01	40	-0.01	0	N/A
hsa-miR-6843-3p	3528-3534	7mer-1A	-0.01	29	-0.01	0	N/A
hsa-miR-219b-3p	3532-3538	7mer-1A	-0.01	29	-0.01	0	N/A
hsa-miR-219a-2-3p	3532-3538	7mer-1A	-0.01	21	-0.01	0.58	N/A
hsa-miR-4452	3533-3539	7mer-1A	-0.01	46	-0.01	0	N/A
hsa-miR-183-3p	3533-3539	7mer-1A	-0.01	43	-0.01	0.085	N/A
hsa-miR-4507	3537-3544	8mer	-0.13	78	-0.11	0	N/A
hsa-miR-3940-5p	3537-3544	8mer	-0.04	65	-0.03	0	N/A
hsa-miR-6839-3p	3538-3544	7mer-1A	-0.01	21	-0.01	0	N/A
hsa-miR-4740-3p	3546-3552	7mer-m8	-0.02	17	-0.02	0	N/A
hsa-miR-3943	3550-3556	7mer-m8	-0.1	74	-0.08	0.024	N/A
hsa-miR-1225-3p	3551-3557	7mer-m8	-0.07	64	-0.06	0.024	N/A
hsa-miR-1276	3554-3560	7mer-m8	-0.02	69	-0.02	0.024	N/A
hsa-miR-8060	3559-3565	7mer-1A	-0.01	39	-0.01	0	N/A
hsa-miR-7154-5p	3559-3565	7mer-m8	-0.02	28	-0.02	0	N/A
hsa-miR-15b-3p	3562-3568	7mer-m8	-0.02	43	-0.02	0.085	N/A
hsa-miR-4641	3571-3577	7mer-1A	-0.01	12	-0.01	0	N/A
hsa-miR-656-5p	3574-3581	8mer	-0.12	72	-0.1	0	N/A
hsa-miR-504-5p.1	3579-3585	7mer-m8	-0.03	61	-0.03	0.229	N/A

hsa-miR-6732-3p	3580-3586	7mer-m8	-0.02	32	-0.02	0	N/A
hsa-miR-1305	3584-3590	7mer-m8	-0.02	78	-0.02	0	N/A
hsa-miR-5680	3596-3602	7mer-1A	-0.01	53	-0.01	0	N/A
hsa-miR-8067	3597-3603	7mer-m8	-0.02	27	-0.02	0	N/A
hsa-miR-6508-5p	3597-3603	7mer-m8	-0.02	27	-0.02	0	N/A
hsa-miR-3667-5p	3603-3609	7mer-m8	-0.04	62	-0.04	0	N/A
hsa-let-7c-3p	3613-3620	8mer	-0.03	48	-0.03	0	N/A
hsa-let-7g-3p	3614-3620	7mer-1A	-0.01	37	-0.01	0	N/A
hsa-let-7a-2-3p	3614-3620	7mer-1A	-0.01	36	-0.01	0	N/A
hsa-miR-493-5p	3614-3620	7mer-1A	-0.01	31	-0.01	0.327	N/A
hsa-miR-643	3616-3622	7mer-1A	-0.01	18	-0.01	0.024	N/A
hsa-miR-7114-3p	3625-3631	7mer-m8	-0.06	65	-0.05	0.085	N/A
hsa-miR-1180-5p	3625-3631	7mer-m8	-0.02	54	-0.02	0.085	N/A
hsa-miR-150-3p	3632-3638	7mer-m8	-0.02	11	-0.02	0.085	N/A
hsa-miR-4774-5p	3634-3640	7mer-1A	-0.01	13	-0.01	0	N/A
hsa-miR-4490	3634-3640	7mer-1A	-0.01	7	-0.01	0	N/A
hsa-miR-6824-3p	3635-3641	7mer-m8	-0.02	50	-0.02	0	N/A
hsa-miR-6764-3p	3635-3641	7mer-m8	-0.02	50	-0.02	0	N/A
hsa-miR-760	3637-3643	7mer-m8	-0.02	24	-0.02	0.029	N/A
hsa-miR-4283	3639-3645	7mer-m8	-0.03	45	-0.03	0	N/A
hsa-miR-6846-5p	3640-3646	7mer-m8	-0.02	9	-0.02	0	N/A
hsa-miR-6848-5p	3640-3646	7mer-m8	-0.02	8	-0.02	0	N/A
hsa-miR-301a-5p	3647-3653	7mer-m8	-0.02	68	-0.02	0.085	N/A
hsa-miR-301b-5p	3647-3653	7mer-m8	-0.02	68	-0.02	0.085	N/A
hsa-miR-4714-5p	3648-3655	8mer	-0.03	38	-0.03	0	N/A

hsa-miR-514a-5p	3649-3655	7mer-1A	-0.01	34	-0.01	0.085	N/A
hsa-miR-3664-5p	3649-3655	7mer-1A	-0.01	25	-0.01	0	N/A
hsa-miR-6849-3p	3663-3669	7mer-m8	-0.03	54	-0.03	0	N/A
hsa-miR-4482-5p	3666-3672	7mer-1A	-0.06	70	-0.05	0	N/A
hsa-miR-1295b-5p	3666-3672	7mer-1A	-0.05	60	-0.04	0	N/A
hsa-miR-3130-5p	3666-3672	7mer-1A	-0.01	25	-0.01	0	N/A
hsa-miR-1912	3666-3672	7mer-1A	-0.01	12	-0.01	0	N/A
hsa-miR-3162-3p	3669-3675	7mer-m8	-0.06	52	-0.05	0	N/A
hsa-miR-4684-5p	3675-3681	7mer-m8	-0.02	48	-0.02	0	N/A
hsa-miR-3675-3p	3676-3682	7mer-m8	-0.02	31	-0.02	0	N/A
hsa-miR-216b-5p	3677-3683	7mer-m8	-0.02	37	-0.02	1.041	＜ 0.1
hsa-miR-6716-5p	3681-3687	7mer-1A	-0.01	20	-0.01	0	N/A
hsa-miR-4717-3p	3685-3691	7mer-m8	-0.02	43	-0.02	0	N/A
hsa-miR-5589-3p	3686-3693	8mer	-0.03	40	-0.03	0	N/A
hsa-miR-1273g-3p	3691-3698	8mer	-0.03	52	-0.03	0	N/A
hsa-miR-6509-3p	3692-3698	7mer-m8	-0.02	29	-0.02	0	N/A
hsa-miR-181a-2-3p	3692-3698	7mer-1A	-0.01	29	-0.01	0	N/A
hsa-miR-6845-3p	3696-3702	7mer-1A	-0.01	28	-0.01	0	N/A
hsa-miR-6833-3p	3697-3703	7mer-m8	-0.02	61	-0.02	0	N/A
hsa-miR-4768-5p	3697-3703	7mer-m8	-0.02	59	-0.02	0	N/A
hsa-miR-6873-3p	3697-3703	7mer-1A	-0.01	20	-0.01	0	N/A
hsa-miR-2117	3698-3705	8mer	-0.03	33	-0.03	0	N/A
hsa-miR-4273	3699-3706	8mer	-0.03	77	-0.03	0	N/A

hsa-miR-7156-5p	3699-3706	8mer	-0.03	66	-0.03	0	N/A
hsa-miR-4677-5p	3700-3706	7mer-1A	-0.01	31	-0.01	0.085	N/A
hsa-miR-6739-3p	3700-3706	7mer-m8	-0.02	29	-0.02	0	N/A
hsa-miR-4445-5p	3702-3708	7mer-m8	-0.02	24	-0.02	0	N/A
hsa-miR-3158-5p	3707-3713	7mer-m8	-0.02	43	-0.02	0	N/A
hsa-miR-509-5p	3708-3714	7mer-m8	-0.03	66	-0.03	0	N/A
hsa-miR-509-3-5p	3708-3714	7mer-m8	-0.02	59	-0.02	0	N/A
hsa-miR-4418	3708-3714	7mer-m8	-0.02	52	-0.02	0	N/A
hsa-miR-5002-3p	3710-3716	7mer-1A	-0.03	36	-0.03	0	N/A
hsa-miR-3118	3711-3717	7mer-m8	-0.08	59	-0.07	0.941	N/A
hsa-miR-134-5p	3711-3717	7mer-m8	-0.07	55	-0.06	0.941	N/A
hsa-miR-5190	3713-3720	8mer	-0.03	71	-0.03	0	N/A
hsa-miR-4276	3714-3720	7mer-m8	-0.02	25	-0.02	0	N/A
hsa-miR-5094	3715-3722	8mer	-0.03	43	-0.03	0	N/A
hsa-miR-374a-3p	3717-3723	7mer-m8	-0.02	46	-0.02	0.085	N/A
hsa-miR-361-5p	3717-3723	7mer-1A	-0.01	12	-0.01	0.245	N/A
hsa-miR-3155b	3723-3729	7mer-1A	-0.04	74	-0.04	0.099	N/A
hsa-miR-3155a	3723-3729	7mer-1A	-0.04	74	-0.04	0.099	N/A
hsa-miR-484	3723-3729	7mer-1A	-0.01	50	-0.01	0.099	N/A
hsa-miR-1266-5p	3725-3731	7mer-1A	-0.06	71	-0.05	0.024	N/A
hsa-miR-4518	3725-3731	7mer-1A	-0.02	69	-0.02	0.024	N/A
hsa-miR-3664-3p	3725-3731	7mer-1A	-0.02	44	-0.02	0	N/A
hsa-miR-3184-3p	3728-3734	7mer-m8	-0.02	52	-0.02	0	N/A
hsa-miR-3691-3p	3730-3736	7mer-m8	-0.02	52	-0.02	0	N/A
hsa-miR-4480	3731-3737	7mer-m8	-0.02	39	-0.02	0	N/A

hsa-miR-6807-5p	3734-3740	7mer-m8	-0.02	32	-0.02	0	N/A
hsa-miR-3646	3735-3746	non-canonical	N/A	N/A	N/A	0	N/A
hsa-miR-3646	3735-3746	non-canonical	N/A	N/A	N/A	0	N/A
hsa-miR-1252-3p	3737-3743	7mer-m8	-0.02	64	-0.02	0	N/A
hsa-miR-203a-3p.1	3739-3745	7mer-1A	-0.01	33	-0.01	0.058	＜0.1
hsa-miR-510-3p	3741-3747	7mer-1A	-0.01	43	-0.01	0	N/A
hsa-miR-3140-3p	3744-3750	7mer-m8	-0.02	42	-0.02	0	N/A
hsa-miR-887-3p	3750-3756	7mer-1A	-0.09	61	-0.08	0	N/A
hsa-miR-219a-2-3p	3754-3760	7mer-1A	-0.03	49	-0.03	0.635	N/A
hsa-miR-219b-3p	3754-3760	7mer-1A	-0.01	29	-0.01	0	N/A
hsa-miR-3605-5p	3759-3766	8mer	-0.07	52	-0.06	0	N/A
hsa-miR-1185-5p	3760-3766	7mer-1A	-0.08	58	-0.07	0.024	N/A
hsa-miR-3679-5p	3760-3766	7mer-1A	-0.02	15	-0.01	0.024	N/A
hsa-miR-4710	3762-3769	8mer	-0.08	66	-0.07	0	N/A
hsa-miR-4792	3763-3769	7mer-1A	-0.03	46	-0.03	0	N/A
hsa-miR-7855-5p	3763-3769	7mer-m8	-0.02	35	-0.02	0	N/A
hsa-miR-4299	3764-3770	7mer-m8	-0.02	36	-0.02	0	N/A
hsa-miR-133a-5p	3766-3772	7mer-1A	-0.2	87	-0.17	0.085	N/A
hsa-miR-6131	3766-3772	7mer-1A	-0.01	30	-0.01	0.024	N/A
hsa-miR-3692-5p	3767-3773	7mer-m8	-0.12	81	-0.1	0	N/A
hsa-miR-203b-3p	3771-3777	7mer-m8	-0.02	47	-0.02	0	N/A

hsa-miR-4539	3772-3778	7mer-m8	-0.02	46	-0.02	0	N/A
hsa-miR-4267	3777-3783	7mer-1A	-0.01	42	-0.01	0	N/A
hsa-miR-6512-3p	3778-3784	7mer-1A	-0.01	24	-0.01	0	N/A
hsa-miR-6720-5p	3778-3784	7mer-1A	-0.01	24	-0.01	0	N/A
hsa-miR-3180-5p	3779-3785	7mer-1A	-0.01	29	-0.01	0	N/A
hsa-miR-210-5p	3785-3791	7mer-1A	-0.04	38	-0.03	0.085	N/A
hsa-miR-4749-3p	3785-3791	7mer-1A	-0.01	25	-0.01	0	N/A
hsa-miR-503-3p	3792-3798	7mer-m8	-0.02	26	-0.02	0	N/A
hsa-miR-6835-5p	3794-3801	8mer	-0.08	38	-0.07	0	N/A
hsa-miR-6751-5p	3795-3801	7mer-m8	-0.08	63	-0.07	0	N/A
hsa-miR-6803-5p	3795-3801	7mer-m8	-0.05	46	-0.04	0	N/A
hsa-miR-6752-5p	3795-3801	7mer-1A	-0.09	44	-0.08	0	N/A
hsa-miR-6842-5p	3795-3801	7mer-1A	-0.09	33	-0.07	0	N/A
hsa-miR-7110-5p	3795-3801	7mer-1A	-0.07	30	-0.06	0	N/A
hsa-miR-4447	3796-3802	7mer-m8	-0.09	70	-0.08	0	N/A
hsa-miR-4472	3796-3802	7mer-m8	-0.09	70	-0.08	0	N/A
hsa-miR-92a-2-5p	3797-3803	7mer-m8	-0.07	65	-0.06	0	N/A
hsa-miR-125a-3p	3800-3806	7mer-1A	-0.06	66	-0.05	0.024	N/A
hsa-miR-764	3800-3806	7mer-1A	-0.01	51	-0.01	0	N/A
hsa-miR-3934-5p	3800-3806	7mer-1A	-0.01	34	-0.01	0	N/A
hsa-miR-3934-3p	3802-3808	7mer-m8	-0.06	58	-0.05	0	N/A
hsa-miR-6854-5p	3803-3809	7mer-m8	-0.02	40	-0.02	0	N/A
hsa-miR-330-3p	3807-3813	7mer-1A	-0.01	30	-0.01	0.265	N/A
hsa-miR-607	3809-3815	7mer-1A	-0.01	40	-0.01	0	N/A
hsa-miR-1305	3810-3816	7mer-1A	-0.01	49	-0.01	0	N/A

hsa-miR-6837-5p	3818-3824	7mer-m8	-0.05	57	-0.05	0	N/A
hsa-miR-4685-5p	3818-3824	7mer-m8	-0.04	53	-0.03	0	N/A
hsa-miR-1915-3p	3819-3826	8mer	-0.03	64	-0.03	0	N/A
hsa-miR-6764-5p	3819-3826	8mer	-0.03	60	-0.03	0	N/A
hsa-miR-4726-3p	3820-3826	7mer-1A	-0.01	39	-0.01	0	N/A
hsa-miR-6840-3p	3820-3826	7mer-1A	-0.01	37	-0.01	0	N/A
hsa-miR-2467-3p	3828-3835	8mer	-0.03	44	-0.03	0	N/A
hsa-miR-3678-3p	3829-3835	7mer-m8	-0.02	38	-0.02	0	N/A
hsa-miR-3123	3835-3842	8mer	-0.03	71	-0.03	0	N/A
hsa-miR-3925-5p	3836-3842	7mer-1A	-0.01	54	-0.01	0	N/A
hsa-miR-1303	3837-3843	7mer-m8	-0.02	23	-0.02	0.024	N/A
hsa-miR-374c-3p	3840-3846	7mer-1A	-0.01	34	-0.01	0	N/A
hsa-miR-7159-3p	3851-3857	7mer-1A	-0.01	36	-0.01	0	N/A
hsa-miR-4482-3p	3851-3857	7mer-1A	-0.01	29	-0.01	0	N/A
hsa-miR-29b-2-5p	3854-3860	7mer-m8	-0.02	56	-0.02	0	N/A
hsa-miR-29b-1-5p	3855-3861	7mer-m8	-0.02	43	-0.02	0	N/A
hsa-miR-4463	3858-3864	7mer-m8	-0.02	55	-0.02	0	N/A
hsa-miR-873-3p	3859-3865	7mer-m8	-0.02	45	-0.02	0	N/A
hsa-miR-6077	3863-3869	7mer-m8	-0.02	50	-0.02	0	N/A
hsa-miR-9500	3865-3871	7mer-m8	-0.02	39	-0.02	0	N/A
hsa-miR-6797-5p	3867-3873	7mer-m8	-0.08	59	-0.07	0	N/A
hsa-miR-1249-5p	3867-3873	7mer-m8	-0.08	59	-0.07	0	N/A
hsa-miR-4779	3868-3874	7mer-m8	-0.12	80	-0.1	0	N/A
hsa-miR-6891-5p	3869-3875	7mer-m8	-0.03	52	-0.03	0.099	N/A
hsa-miR-3173-3p	3869-3875	7mer-m8	-0.02	36	-0.02	0.099	N/A

hsa-miR-4719	3874-3880	7mer-1A	-0.01	38	-0.01	0	N/A
hsa-miR-4660	3883-3889	7mer-m8	-0.1	74	-0.08	0.024	N/A
hsa-miR-646	3884-3891	8mer	-0.03	48	-0.03	0.024	N/A
hsa-miR-6838-5p	3885-3891	7mer-1A	-0.08	65	-0.07	0.154	< 0.1
hsa-miR-4524a-5p	3885-3891	7mer-m8	-0.08	64	-0.07	0	N/A
hsa-miR-4524b-5p	3885-3891	7mer-m8	-0.07	60	-0.06	0	N/A
hsa-miR-503-5p	3885-3891	7mer-1A	-0.05	60	-0.04	0.154	< 0.1
hsa-miR-424-5p	3885-3891	7mer-1A	-0.05	57	-0.04	0.154	< 0.1
hsa-miR-497-5p	3885-3891	7mer-1A	-0.05	57	-0.04	0.154	< 0.1
hsa-miR-15b-5p	3885-3891	7mer-1A	-0.03	39	-0.02	0.154	< 0.1
hsa-miR-16-5p	3885-3891	7mer-1A	-0.02	25	-0.01	0.154	< 0.1
hsa-miR-195-5p	3885-3891	7mer-1A	-0.02	25	-0.01	0.154	< 0.1
hsa-miR-15a-5p	3885-3891	7mer-1A	-0.01	18	-0.01	0.154	< 0.1
hsa-miR-6505-5p	3890-3896	7mer-m8	-0.02	34	-0.02	0	N/A
hsa-miR-24-2-5p	3898-3904	7mer-1A	-0.03	15	-0.03	0	N/A
hsa-miR-24-1-5p	3898-3904	7mer-1A	-0.03	15	-0.03	0	N/A
hsa-miR-4774-3p	3899-3905	7mer-m8	-0.02	20	-0.02	0	N/A
hsa-miR-4423-5p	3900-3907	8mer	-0.03	22	-0.03	0	N/A

hsa-miR-744-3p	3901-3907	7mer-m8	-0.02	33	-0.02	0	N/A
hsa-miR-6501-5p	3901-3907	7mer-1A	-0.06	23	-0.05	0	N/A
hsa-miR-3934-5p	3905-3911	7mer-m8	-0.02	47	-0.02	0	N/A
hsa-miR-552-3p	3906-3912	7mer-m8	-0.02	33	-0.02	0.024	N/A
hsa-miR-421	3909-3916	8mer	-0.03	49	-0.03	1.156	N/A
hsa-miR-4709-5p	3909-3915	7mer-1A	-0.01	33	-0.01	0	N/A
hsa-miR-505-3p.2	3910-3916	7mer-1A	-0.01	46	-0.01	1.206	N/A
hsa-miR-7159-5p	3910-3916	7mer-1A	-0.01	20	-0.01	0	N/A
hsa-miR-1305	3911-3917	7mer-m8	-0.02	78	-0.02	0	N/A
hsa-miR-3616-3p	3919-3926	8mer	-0.32	94	-0.27	0	N/A
hsa-miR-1226-5p	3920-3927	8mer	-0.25	94	-0.21	0	N/A
hsa-miR-4721	3920-3926	7mer-1A	-0.18	81	-0.15	0	N/A
hsa-miR-6834-5p	3921-3927	7mer-1A	-0.15	86	-0.13	0	N/A
hsa-miR-6734-5p	3921-3927	7mer-1A	-0.06	53	-0.05	0	N/A
hsa-miR-411-5p.1	3929-3935	7mer-m8	-0.06	82	-0.05	0.96	N/A
hsa-miR-2467-3p	3934-3940	7mer-1A	-0.12	76	-0.1	0	N/A
hsa-miR-3678-3p	3934-3940	7mer-m8	-0.08	71	-0.07	0	N/A
hsa-miR-5680	3939-3945	7mer-m8	-0.02	68	-0.02	0	N/A
hsa-miR-513b-5p	3948-3954	7mer-m8	-0.02	48	-0.02	0.099	N/A
hsa-miR-125b-2-3p	3948-3954	7mer-1A	-0.01	32	-0.01	0	N/A
hsa-miR-4457	3948-3954	7mer-1A	-0.01	31	-0.01	0	N/A
hsa-miR-218-5p	3956-3962	7mer-m8	-0.04	57	-0.04	2.624	0.28
hsa-miR-636	3956-3962	7mer-1A	-0.01	21	-0.01	0.085	N/A

hsa-miR-6867-5p	3959-3965	7mer-m8	-0.07	42	-0.06	0	N/A
hsa-miR-595	3961-3967	7mer-m8	-0.02	55	-0.02	0.024	N/A
hsa-miR-548az-5p	3963-3970	8mer	-0.03	78	-0.03	0	N/A
hsa-miR-548t-5p	3963-3970	8mer	-0.03	78	-0.03	0	N/A
hsa-miR-548n	3964-3970	7mer-1A	-0.01	41	-0.01	0.024	N/A
hsa-miR-95-5p	3967-3973	7mer-m8	-0.02	22	-0.02	0.085	N/A
hsa-miR-106a-3p	3970-3976	7mer-1A	-0.01	32	-0.01	0	N/A
hsa-miR-4684-3p	3971-3978	8mer	-0.08	64	-0.07	0	N/A
hsa-miR-4781-3p	3974-3980	7mer-1A	-0.01	32	-0.01	0	N/A
hsa-miR-4528	3978-3984	7mer-1A	-0.01	22	-0.01	0	N/A
hsa-miR-5694	3981-3987	7mer-m8	-0.02	47	-0.02	0	N/A
hsa-miR-1288-5p	3982-3988	7mer-m8	-0.02	30	-0.02	0	N/A
hsa-miR-4468	3985-3992	8mer	-0.1	71	-0.09	0	N/A
hsa-miR-8066	3990-3996	7mer-m8	-0.02	34	-0.02	0	N/A
hsa-miR-203a-3p.1	3992-3998	7mer-m8	-0.02	50	-0.02	0.829	0.23
hsa-miR-5093	3993-3999	7mer-m8	-0.02	40	-0.02	0	N/A
hsa-miR-651-3p	3995-4001	7mer-m8	-0.02	37	-0.02	0	N/A
hsa-miR-6830-5p	3996-4002	7mer-m8	-0.02	24	-0.02	0	N/A
hsa-miR-500b-3p	3997-4009	non-canonical	N/A	N/A	N/A	0	N/A
hsa-miR-500b-3p	3997-4009	non-canonical	N/A	N/A	N/A	0	N/A
hsa-miR-6809-5p	3998-4004	7mer-m8	-0.11	68	-0.09	0	N/A
hsa-miR-4742-5p	4000-4006	7mer-m8	-0.02	36	-0.02	0.024	N/A

hsa-miR-4645-5p	4001-4007	7mer-m8	-0.02	32	-0.02	0	N/A
hsa-miR-4673	4001-4007	7mer-m8	-0.02	28	-0.02	0	N/A
hsa-miR-361-3p	4004-4010	7mer-m8	-0.02	27	-0.02	0.099	N/A
hsa-miR-3943	4006-4012	7mer-m8	-0.1	75	-0.08	0.024	N/A
hsa-miR-6856-3p	4008-4015	8mer	-0.03	48	-0.03	0.085	N/A
hsa-miR-329-5p	4015-4021	7mer-m8	-0.02	51	-0.02	0.085	N/A
hsa-miR-3607-3p	4021-4027	7mer-1A	-0.01	39	-0.01	0	N/A
hsa-miR-3686	4021-4028	8mer	-0.03	37	-0.03	0	N/A
hsa-miR-26b-3p	4025-4032	8mer	-0.03	47	-0.03	0.085	N/A
hsa-miR-4652-3p	4027-4034	8mer	-0.03	75	-0.03	0	N/A
hsa-miR-4743-3p	4028-4034	7mer-1A	-0.01	39	-0.01	0	N/A
hsa-miR-876-5p	4031-4037	7mer-m8	-0.05	70	-0.04	1.634	N/A
hsa-miR-3167	4031-4037	7mer-m8	-0.04	61	-0.03	1.634	N/A
hsa-miR-541-5p	4033-4039	7mer-m8	-0.02	38	-0.02	0.085	N/A
hsa-miR-6809-5p	4036-4042	7mer-m8	-0.09	64	-0.08	0	N/A
hsa-miR-583	4041-4047	7mer-m8	-0.02	58	-0.02	0.024	N/A
hsa-miR-4299	4047-4053	7mer-m8	-0.03	50	-0.02	0	N/A
hsa-miR-138-5p	4048-4054	7mer-m8	-0.08	58	-0.07	0.433	＜ 0.1
hsa-miR-3652	4050-4056	7mer-1A	-0.15	86	-0.13	0.437	N/A
hsa-miR-4430	4050-4056	7mer-1A	-0.12	81	-0.1	0.437	N/A
hsa-miR-4505	4050-4056	7mer-1A	-0.12	71	-0.1	0.437	N/A
hsa-miR-5787	4050-4056	7mer-1A	-0.06	68	-0.05	0.437	N/A
hsa-miR-6842-3p	4050-4056	7mer-m8	-0.09	65	-0.08	0.437	N/A
hsa-miR-4474-3p	4052-4058	7mer-m8	-0.04	40	-0.03	0	N/A

hsa-miR-644a	4053-4059	7mer-m8	-0.15	81	-0.12	0	N/A
hsa-miR-3934-5p	4056-4062	7mer-m8	-0.02	47	-0.02	0	N/A
hsa-miR-3665	4057-4063	7mer-m8	-0.11	66	-0.1	0	N/A
hsa-miR-4736	4058-4065	8mer	-0.06	68	-0.05	0	N/A
hsa-miR-4763-3p	4059-4065	7mer-1A	-0.15	80	-0.13	0	N/A
hsa-miR-1207-5p	4059-4065	7mer-1A	-0.12	74	-0.1	0	N/A
hsa-miR-7150	4059-4065	7mer-m8	-0.08	66	-0.07	0	N/A
hsa-miR-183-5p.1	4061-4067	7mer-1A	-0.01	31	-0.01	0.773	< 0.1
hsa-miR-4774-5p	4065-4071	7mer-m8	-0.02	22	-0.02	0	N/A
hsa-miR-331-3p	4068-4074	7mer-m8	-0.02	40	-0.02	0.433	N/A
hsa-miR-5195-5p	4070-4076	7mer-1A	-0.02	8	-0.02	0	N/A
hsa-miR-4718	4074-4080	7mer-m8	-0.02	27	-0.02	0	N/A
hsa-miR-320b	4077-4083	7mer-m8	-0.02	48	-0.02	0.229	N/A
hsa-miR-320d	4077-4083	7mer-m8	-0.02	48	-0.02	0.229	N/A
hsa-miR-4429	4077-4083	7mer-m8	-0.02	48	-0.02	0.229	N/A
hsa-miR-320c	4077-4083	7mer-m8	-0.02	48	-0.02	0.229	N/A
hsa-miR-320a	4077-4083	7mer-m8	-0.02	48	-0.02	0.229	N/A
hsa-miR-520d-5p	4080-4086	7mer-m8	-0.02	68	-0.02	0.085	N/A
hsa-miR-524-5p	4080-4086	7mer-m8	-0.02	67	-0.02	0.085	N/A
hsa-miR-6826-5p	4087-4093	7mer-1A	-0.01	24	-0.01	0	N/A
hsa-miR-676-5p	4090-4096	7mer-1A	-0.01	42	-0.01	0	N/A
hsa-miR-6740-3p	4092-4098	7mer-1A	-0.09	68	-0.08	0	N/A
hsa-miR-4711-3p	4093-4100	8mer	-0.03	41	-0.03	0	N/A
hsa-miR-3120-5p	4099-4105	7mer-m8	-0.04	62	-0.03	0	N/A

hsa-miR-5699-3p	4100-4107	8mer	-0.09	70	-0.07	0	N/A
hsa-miR-4421	4100-4107	8mer	-0.09	67	-0.07	0	N/A
hsa-miR-6748-3p	4101-4107	7mer-1A	-0.04	54	-0.03	0	N/A
hsa-miR-4668-3p	4105-4111	7mer-m8	-0.02	74	-0.02	0	N/A
hsa-miR-548c-3p	4106-4113	8mer	-0.03	93	-0.03	0.024	N/A
hsa-miR-3121-3p	4122-4128	7mer-m8	-0.02	48	-0.02	0.024	N/A
hsa-miR-520d-5p	4128-4134	7mer-1A	-0.01	52	-0.01	0.085	N/A
hsa-miR-524-5p	4128-4134	7mer-1A	-0.01	51	-0.01	0.085	N/A
hsa-miR-548at-5p	4132-4138	7mer-m8	-0.02	46	-0.02	0	N/A
hsa-miR-3065-5p	4136-4142	7mer-m8	-0.02	24	-0.02	0	N/A
hsa-miR-146b-5p	4151-4157	7mer-m8	-0.08	71	-0.07	0.029	＜0.1
hsa-miR-146a-5p	4151-4157	7mer-m8	-0.08	71	-0.07	0.029	＜0.1
hsa-miR-7153-5p	4151-4157	7mer-m8	-0.05	56	-0.04	0.029	＜0.1
hsa-miR-3925-5p	4152-4158	7mer-m8	-0.06	85	-0.05	0	N/A
hsa-miR-4311	4154-4161	8mer	-0.03	78	-0.03	0	N/A
hsa-miR-583	4155-4161	7mer-1A	-0.02	56	-0.01	0.099	N/A
hsa-miR-1276	4155-4161	7mer-1A	-0.01	54	-0.01	0.024	N/A
hsa-miR-187-5p	4162-4168	7mer-1A	-0.05	55	-0.04	0.085	N/A
hsa-miR-548v	4162-4168	7mer-1A	-0.02	26	-0.02	0	N/A
hsa-miR-4276	4167-4173	7mer-1A	-0.04	45	-0.03	0	N/A
hsa-miR-5000-5p	4169-4175	7mer-m8	-0.02	48	-0.02	0	N/A
hsa-miR-520d-5p	4174-4180	7mer-m8	-0.02	68	-0.02	0.085	N/A

hsa-miR-524-5p	4174-4180	7mer-m8	-0.02	67	-0.02	0.085	N/A
hsa-let-7c-3p	4176-4183	8mer	-0.03	48	-0.03	0.085	N/A
hsa-let-7g-3p	4177-4183	7mer-1A	-0.07	72	-0.06	0.085	N/A
hsa-let-7a-2-3p	4177-4183	7mer-1A	-0.05	63	-0.04	0.085	N/A
hsa-miR-493-5p	4177-4183	7mer-1A	-0.01	31	-0.01	0.154	N/A
hsa-miR-1277-5p	4183-4189	7mer-m8	-0.02	53	-0.02	0	N/A
hsa-miR-548c-3p	4187-4193	7mer-1A	-0.01	57	-0.01	0.349	N/A
hsa-miR-4799-5p	4190-4196	7mer-1A	-0.01	35	-0.01	0	N/A
hsa-miR-4263	4191-4197	7mer-1A	-0.02	57	-0.01	0	N/A
hsa-miR-576-5p	4191-4197	7mer-1A	-0.01	37	-0.01	0.085	N/A
hsa-miR-1252-3p	4198-4204	7mer-m8	-0.02	64	-0.02	0	N/A
hsa-miR-3662	4199-4205	7mer-m8	-0.02	64	-0.02	0	N/A
hsa-miR-411-5p.1	4206-4212	7mer-1A	-0.01	32	-0.01	2.702	N/A
hsa-miR-6499-3p	4212-4223	non-canonical	N/A	N/A	N/A	0	N/A
hsa-miR-6499-3p	4212-4223	non-canonical	N/A	N/A	N/A	0	N/A
hsa-miR-8068	4214-4221	8mer	-0.03	42	-0.03	0	N/A
hsa-miR-892a	4219-4225	7mer-m8	-0.08	77	-0.07	0.099	N/A
hsa-miR-548g-3p	4221-4228	8mer	-0.03	69	-0.03	0	N/A
hsa-miR-548av-3p	4222-4228	7mer-1A	-0.01	51	-0.01	0	N/A
hsa-miR-548aq-5p	4226-4233	8mer	-0.03	79	-0.03	0.099	N/A
hsa-miR-559	4226-4233	8mer	-0.03	67	-0.03	0.099	N/A
hsa-miR-548as-5p	4226-4233	8mer	-0.03	67	-0.03	0.099	N/A
hsa-miR-548ap-5p	4226-4233	8mer	-0.03	67	-0.03	0.099	N/A

hsa-miR-548h-5p	4226-4233	8mer	-0.03	66	-0.03	0.099	N/A
hsa-miR-548ak	4226-4233	8mer	-0.03	66	-0.03	0.099	N/A
hsa-miR-548j-5p	4226-4233	8mer	-0.03	66	-0.03	0.099	N/A
hsa-miR-548au-5p	4226-4233	8mer	-0.03	66	-0.03	0.099	N/A
hsa-miR-548b-5p	4226-4233	8mer	-0.03	65	-0.03	0.099	N/A
hsa-miR-548o-5p	4226-4233	8mer	-0.03	65	-0.03	0.099	N/A
hsa-miR-548d-5p	4226-4233	8mer	-0.03	65	-0.03	0.099	N/A
hsa-miR-548ay-5p	4226-4233	8mer	-0.03	65	-0.03	0.099	N/A
hsa-miR-548w	4226-4233	8mer	-0.03	65	-0.03	0.099	N/A
hsa-miR-548ad-5p	4226-4233	8mer	-0.03	65	-0.03	0.099	N/A
hsa-miR-548am-5p	4226-4233	8mer	-0.03	65	-0.03	0.099	N/A
hsa-miR-548ae-5p	4226-4233	8mer	-0.03	65	-0.03	0.099	N/A
hsa-miR-548c-5p	4226-4233	8mer	-0.03	65	-0.03	0.099	N/A
hsa-miR-548i	4226-4233	8mer	-0.03	64	-0.03	0.099	N/A
hsa-miR-548a-5p	4226-4233	8mer	-0.03	64	-0.03	0.099	N/A
hsa-miR-548y	4226-4233	8mer	-0.03	64	-0.03	0.099	N/A
hsa-miR-548bb-5p	4226-4233	8mer	-0.03	64	-0.03	0.099	N/A
hsa-miR-548ar-5p	4226-4233	8mer	-0.03	63	-0.03	0.099	N/A
hsa-miR-548ab	4226-4233	8mer	-0.03	63	-0.03	0.099	N/A
hsa-miR-8054	4227-4233	7mer-1A	-0.01	59	-0.01	0.024	N/A
hsa-miR-548av-5p	4227-4233	7mer-1A	-0.01	52	-0.01	0.024	N/A
hsa-miR-548k	4227-4233	7mer-1A	-0.01	51	-0.01	0.024	N/A
hsa-miR-548l	4227-4233	7mer-1A	-0.01	43	-0.01	0.024	N/A
hsa-miR-4744	4229-4235	7mer-1A	-0.04	57	-0.03	0	N/A
hsa-miR-4509	4229-4235	7mer-1A	-0.01	32	-0.01	0	N/A

hsa-miR-520f-5p	4230-4237	8mer	-0.03	47	-0.03	0	N/A
hsa-miR-6873-3p	4233-4239	7mer-m8	-0.02	32	-0.02	0	N/A
hsa-miR-7110-3p	4233-4239	7mer-1A	-0.01	30	-0.01	0	N/A
hsa-miR-6817-3p	4233-4239	7mer-1A	-0.01	26	-0.01	0	N/A
hsa-miR-6895-3p	4234-4240	7mer-m8	-0.1	81	-0.09	0	N/A
hsa-miR-6516-5p	4239-4245	7mer-m8	-0.02	34	-0.02	0.65	N/A
hsa-miR-92b-3p	4241-4247	7mer-1A	-0.01	40	-0.01	1.67	< 0.1
hsa-miR-25-3p	4241-4247	7mer-1A	-0.01	40	-0.01	1.67	< 0.1
hsa-miR-92a-3p	4241-4247	7mer-1A	-0.01	39	-0.01	1.67	< 0.1
hsa-miR-32-5p	4241-4247	7mer-1A	-0.01	35	-0.01	1.67	< 0.1
hsa-miR-367-3p	4241-4247	7mer-1A	-0.01	35	-0.01	1.67	< 0.1
hsa-miR-363-3p	4241-4247	7mer-1A	-0.01	35	-0.01	1.67	< 0.1
hsa-miR-4482-3p	4244-4250	7mer-m8	-0.02	51	-0.02	0	N/A
hsa-miR-607	4256-4263	8mer	-0.03	85	-0.03	0	N/A
hsa-miR-3671	4256-4262	7mer-1A	-0.01	27	-0.01	0	N/A
hsa-miR-4328	4261-4267	7mer-1A	-0.04	73	-0.03	0	N/A
hsa-miR-30e-3p	4263-4269	7mer-m8	-0.02	54	-0.02	0.024	N/A
hsa-miR-30d-3p	4263-4269	7mer-m8	-0.02	54	-0.02	0.024	N/A
hsa-miR-30a-3p	4263-4269	7mer-m8	-0.02	54	-0.02	0.024	N/A

hsa-miR-205-3p	4264-4270	7mer-m8	-0.02	69	-0.02	0	N/A
hsa-miR-6832-3p	4279-4285	7mer-m8	-0.04	60	-0.04	0	N/A
hsa-miR-652-5p	4282-4288	7mer-1A	-0.12	82	-0.1	0	N/A
hsa-miR-6732-3p	4282-4288	7mer-1A	-0.01	23	-0.01	0	N/A
hsa-miR-3152-5p	4293-4299	7mer-m8	-0.16	84	-0.14	0	N/A
hsa-miR-6836-3p	4293-4299	7mer-1A	-0.08	68	-0.07	0	N/A
hsa-miR-6881-3p	4298-4305	8mer	-0.03	49	-0.03	0	N/A
hsa-miR-877-3p	4298-4304	7mer-1A	-0.01	28	-0.01	0	N/A
hsa-miR-6780a-3p	4299-4305	7mer-1A	-0.16	85	-0.14	0	N/A
hsa-miR-7111-3p	4299-4305	7mer-1A	-0.01	33	-0.01	0	N/A
hsa-miR-670-3p	4300-4306	7mer-1A	-0.06	61	-0.05	0.718	N/A
hsa-miR-1238-3p	4300-4306	7mer-1A	-0.01	43	-0.01	0	N/A
hsa-miR-3124-3p	4301-4307	7mer-m8	-0.02	37	-0.02	0	N/A
hsa-miR-3158-3p	4305-4311	7mer-m8	-0.05	54	-0.04	0	N/A
hsa-miR-5681a	4308-4314	7mer-m8	-0.02	40	-0.02	0	N/A
hsa-miR-550b-2-5p	4329-4336	8mer	-0.19	88	-0.16	0	N/A
hsa-miR-550a-5p	4329-4335	7mer-1A	-0.16	75	-0.14	0.099	N/A
hsa-miR-550a-3-5p	4329-4335	7mer-1A	-0.16	75	-0.14	0.099	N/A
hsa-miR-1271-3p	4329-4335	7mer-1A	-0.1	57	-0.09	0.099	N/A
hsa-miR-544b	4331-4342	non-canonical	N/A	N/A	N/A	0	N/A
hsa-miR-544b	4331-4342	non-canonical	N/A	N/A	N/A	0	N/A

hsa-miR-6086	4334-4341	8mer	-0.03	60	-0.03	0	N/A
hsa-miR-377-5p	4334-4341	8mer	-0.03	40	-0.03	0	N/A
hsa-miR-655-5p	4335-4341	7mer-1A	-0.1	71	-0.09	0	N/A
hsa-miR-6793-3p	4342-4348	7mer-1A	-0.01	39	-0.01	0	N/A
hsa-miR-590-5p	4351-4357	7mer-1A	-0.01	32	-0.01	2.045	0.12
hsa-miR-5579-3p	4351-4357	7mer-m8	-0.02	20	-0.02	0	N/A
hsa-miR-21-5p	4351-4357	7mer-1A	-0.01	20	-0.01	2.045	0.12
hsa-miR-192-5p	4356-4362	7mer-m8	-0.02	28	-0.02	1.924	0.17
hsa-miR-215-5p	4356-4362	7mer-m8	-0.02	22	-0.02	1.924	0.17
hsa-miR-197-3p	4369-4375	7mer-1A	-0.01	30	-0.01	0.099	N/A
hsa-miR-412-3p	4370-4377	8mer	-0.07	68	-0.06	0.099	N/A
hsa-miR-6754-3p	4370-4377	8mer	-0.05	65	-0.04	0.099	N/A
hsa-miR-6837-3p	4371-4377	7mer-1A	-0.01	25	-0.01	0	N/A
hsa-miR-4778-3p	4373-4380	8mer	-0.03	59	-0.03	0	N/A
hsa-miR-1236-3p	4376-4382	7mer-1A	-0.1	77	-0.08	0.024	N/A
hsa-miR-6515-3p	4376-4382	7mer-1A	-0.07	68	-0.06	0	N/A
hsa-miR-6809-3p	4377-4383	7mer-m8	-0.02	49	-0.02	0	N/A
hsa-miR-4753-3p	4377-4383	7mer-1A	-0.01	28	-0.01	0	N/A
hsa-miR-942-5p	4378-4384	7mer-m8	-0.02	38	-0.02	0.024	N/A
hsa-miR-103a-2-5p	4380-4386	7mer-1A	-0.04	52	-0.03	0.085	N/A

hsa-miR-548ad-3p	4387-4393	7mer-m8	-0.02	29	-0.02	0	N/A
hsa-miR-548ac	4389-4395	7mer-1A	-0.01	57	-0.01	0	N/A
hsa-miR-548z	4389-4395	7mer-1A	-0.01	57	-0.01	0	N/A
hsa-miR-548bb-3p	4389-4395	7mer-1A	-0.01	57	-0.01	0	N/A
hsa-miR-548d-3p	4389-4395	7mer-1A	-0.01	57	-0.01	0	N/A
hsa-miR-548h-3p	4389-4395	7mer-1A	-0.01	57	-0.01	0	N/A
hsa-miR-548ah-3p	4389-4395	7mer-1A	-0.01	50	-0.01	0	N/A
hsa-miR-548am-3p	4389-4395	7mer-1A	-0.01	50	-0.01	0	N/A
hsa-miR-548ae-3p	4389-4395	7mer-1A	-0.01	50	-0.01	0	N/A
hsa-miR-548aq-3p	4389-4395	7mer-1A	-0.01	50	-0.01	0	N/A
hsa-miR-548j-3p	4389-4395	7mer-1A	-0.01	50	-0.01	0	N/A
hsa-miR-548aj-3p	4389-4395	7mer-1A	-0.01	49	-0.01	0	N/A
hsa-miR-548x-3p	4389-4395	7mer-1A	-0.01	49	-0.01	0	N/A
hsa-miR-3163	4391-4397	7mer-1A	-0.01	45	-0.01	0	N/A
hsa-miR-5009-3p	4397-4404	8mer	-0.03	61	-0.03	0	N/A
hsa-miR-3685	4400-4406	7mer-1A	-0.01	39	-0.01	0	N/A
hsa-miR-384	4400-4406	7mer-1A	-0.01	32	-0.01	0.069	N/A
hsa-miR-513c-3p	4403-4409	7mer-m8	-0.02	78	-0.02	0	N/A
hsa-miR-3606-3p	4403-4409	7mer-m8	-0.02	78	-0.02	0	N/A
hsa-miR-513a-3p	4403-4409	7mer-m8	-0.02	78	-0.02	0	N/A
hsa-miR-4282	4404-4411	8mer	-0.03	87	-0.03	0	N/A
hsa-miR-6884-3p	4417-4423	7mer-1A	-0.04	66	-0.04	0	N/A
hsa-miR-587	4419-4425	7mer-1A	-0.01	25	-0.01	0.024	N/A
hsa-miR-3160-5p	4422-4428	7mer-1A	-0.01	25	-0.01	0	N/A
hsa-miR-218-5p	4424-4430	7mer-m8	-0.05	61	-0.04	1.029	<

								0.1
hsa-miR-636	4424-4430	7mer-1A	-0.04	49	-0.04	0	N/A
hsa-miR-6747-3p	4445-4451	7mer-m8	-0.02	31	-0.02	0	N/A
hsa-miR-6793-3p	4456-4462	7mer-m8	-0.04	69	-0.03	0	N/A
hsa-miR-5699-5p	4457-4464	8mer	-0.03	28	-0.03	0	N/A
hsa-miR-3189-5p	4458-4464	7mer-1A	-0.04	46	-0.03	0	N/A
hsa-miR-4758-3p	4458-4464	7mer-1A	-0.01	24	-0.01	0	N/A
hsa-miR-1913	4459-4465	7mer-m8	-0.14	82	-0.12	0.024	N/A
hsa-miR-324-3p	4459-4465	7mer-m8	-0.13	80	-0.11	0.024	N/A
hsa-miR-6890-3p	4461-4467	7mer-m8	-0.02	64	-0.02	0	N/A
hsa-miR-200a-3p	4463-4470	8mer	-0.03	58	-0.03	0.369	＜0.1
hsa-miR-141-3p	4463-4470	8mer	-0.03	57	-0.03	0.369	＜0.1
hsa-miR-885-5p	4469-4475	7mer-1A	-0.01	35	-0.01	0	N/A
hsa-miR-587	4471-4477	7mer-1A	-0.01	25	-0.01	0.024	N/A
hsa-miR-129-2-3p	4476-4482	7mer-m8	-0.04	56	-0.04	0.208	＜0.1
hsa-miR-129-1-3p	4476-4482	7mer-m8	-0.04	56	-0.04	0.208	＜0.1
hsa-miR-1233-3p	4477-4484	8mer	-0.12	78	-0.1	0.099	N/A
hsa-miR-1225-3p	4478-4484	7mer-1A	-0.05	52	-0.04	0.024	N/A
hsa-miR-4709-5p	4484-4490	7mer-m8	-0.02	45	-0.02	0	N/A
hsa-miR-1244	4493-4499	7mer-1A	-0.02	19	-0.01	0.024	N/A
hsa-miR-325	4494-4501	8mer	-0.18	84	-0.16	0.437	N/A

hsa-miR-628-3p	4495-4501	7mer-1A	-0.07	47	-0.06	0.024	N/A
hsa-miR-1290	4502-4509	8mer	-0.03	49	-0.03	0.024	N/A
hsa-miR-876-5p	4503-4509	7mer-1A	-0.01	30	-0.01	0.461	N/A
hsa-miR-3167	4503-4509	7mer-1A	-0.01	29	-0.01	0.461	N/A
hsa-miR-6839-5p	4504-4510	7mer-m8	-0.04	59	-0.04	0.099	N/A
hsa-miR-378j	4504-4510	7mer-m8	-0.02	40	-0.02	0.099	N/A
hsa-miR-1243	4505-4511	7mer-m8	-0.1	78	-0.09	0	N/A
hsa-miR-891b	4509-4515	7mer-1A	-0.01	14	-0.01	0.085	N/A
hsa-miR-3622b-5p	4514-4520	7mer-1A	-0.01	24	-0.01	0	N/A
hsa-miR-183-5p.1	4516-4522	7mer-1A	-0.01	31	-0.01	0.38	＜ 0.1
hsa-miR-4693-3p	4522-4528	7mer-1A	-0.08	55	-0.07	0	N/A
hsa-miR-4712-3p	4523-4529	7mer-m8	-0.04	55	-0.03	0	N/A
hsa-miR-1272	4526-4532	7mer-m8	-0.02	39	-0.02	0.024	N/A
hsa-miR-1245b-3p	4529-4535	7mer-m8	-0.02	30	-0.02	0	N/A
hsa-miR-6868-5p	4533-4539	7mer-m8	-0.15	81	-0.12	0	N/A
hsa-miR-6757-3p	4536-4543	8mer	-0.03	54	-0.03	0	N/A
hsa-miR-6823-5p	4543-4549	7mer-m8	-0.09	74	-0.07	0	N/A
hsa-let-7g-3p	4547-4553	7mer-m8	-0.02	46	-0.02	0	N/A
hsa-let-7a-2-3p	4547-4553	7mer-m8	-0.02	45	-0.02	0	N/A
hsa-miR-4666a-5p	4551-4558	8mer	-0.03	49	-0.03	0	N/A
hsa-miR-600	4554-4560	7mer-1A	-0.05	56	-0.04	0.024	N/A
hsa-miR-627-3p	4558-4564	7mer-m8	-0.02	38	-0.02	0	N/A
hsa-miR-3140-3p	4560-4566	7mer-1A	-0.01	29	-0.01	0	N/A
hsa-miR-31-3p	4571-4577	7mer-1A	-0.05	60	-0.05	0	N/A

hsa-miR-520g-3p	4576-4582	7mer-m8	-0.02	44	-0.02	0.024	N/A
hsa-miR-520h	4576-4582	7mer-m8	-0.02	44	-0.02	0.024	N/A
hsa-miR-3065-5p	4579-4585	7mer-m8	-0.02	24	-0.02	0.442	N/A
hsa-miR-497-3p	4584-4590	7mer-1A	-0.01	55	-0.01	0.489	N/A
hsa-miR-410-3p	4588-4594	7mer-1A	-0.01	55	-0.01	2.098	N/A
hsa-miR-5011-5p	4589-4596	8mer	-0.03	56	-0.03	0	N/A
hsa-miR-190a-3p	4590-4596	7mer-m8	-0.02	40	-0.02	0.085	N/A
hsa-miR-5011-5p	4591-4598	8mer	-0.03	56	-0.03	0	N/A
hsa-miR-190a-3p	4592-4599	8mer	-0.03	69	-0.03	0	N/A
hsa-miR-4528	4595-4601	7mer-m8	-0.02	35	-0.02	0	N/A
hsa-miR-130a-5p	4598-4604	7mer-m8	-0.02	41	-0.02	0.338	< 0.1
hsa-miR-23b-3p	4598-4604	7mer-m8	-0.02	39	-0.02	0.338	< 0.1
hsa-miR-23a-3p	4598-4604	7mer-m8	-0.02	38	-0.02	0.338	< 0.1
hsa-miR-23c	4598-4604	7mer-m8	-0.02	37	-0.02	0.338	< 0.1
hsa-miR-224-5p	4601-4607	7mer-m8	-0.06	79	-0.05	0.51	N/A
hsa-miR-3616-5p	4604-4610	7mer-1A	-0.07	70	-0.06	0	N/A
hsa-miR-573	4604-4610	7mer-1A	-0.05	67	-0.05	0	N/A
hsa-miR-33a-5p	4608-4615	8mer	-0.03	70	-0.03	0.178	< 0.1
hsa-miR-33b-5p	4608-4615	8mer	-0.03	70	-0.03	0.178	< 0.1

hsa-miR-3680-3p	4610-4616	7mer-m8	-0.02	51	-0.02	0	N/A
hsa-miR-4282	4614-4621	8mer	-0.03	87	-0.03	0	N/A
hsa-miR-3163	4616-4622	7mer-m8	-0.02	65	-0.02	0	N/A
hsa-miR-548c-3p	4621-4627	7mer-1A	-0.01	57	-0.01	0.024	N/A
hsa-miR-3163	4623-4629	7mer-1A	-0.01	45	-0.01	0	N/A
hsa-miR-448	4640-4646	7mer-m8	-0.02	36	-0.02	1.03	N/A
hsa-miR-5700	4642-4648	7mer-m8	-0.04	70	-0.04	0	N/A
hsa-miR-4490	4648-4654	7mer-m8	-0.09	61	-0.08	0	N/A
hsa-miR-8078	4661-4667	7mer-1A	-0.08	62	-0.07	0	N/A
hsa-miR-3925-3p	4671-4677	7mer-1A	-0.01	41	-0.01	0	N/A
hsa-miR-766-3p	4671-4677	7mer-1A	-0.01	31	-0.01	0.024	N/A
hsa-miR-126-5p	4681-4687	7mer-m8	-0.02	51	-0.02	0	N/A
hsa-miR-648	4693-4699	7mer-m8	-0.14	84	-0.12	0.085	N/A
hsa-miR-595	4694-4700	7mer-m8	-0.02	55	-0.02	0.099	N/A
hsa-miR-520h	4696-4702	7mer-m8	-0.02	44	-0.02	0.099	N/A
hsa-miR-520g-3p	4696-4702	7mer-m8	-0.02	44	-0.02	0.099	N/A
hsa-miR-7158-3p	4705-4711	7mer-m8	-0.02	39	-0.02	0	N/A
hsa-miR-7855-5p	4709-4715	7mer-1A	-0.02	39	-0.02	0	N/A
hsa-miR-8087	4717-4723	7mer-1A	-0.01	45	-0.01	0	N/A
hsa-miR-4432	4717-4723	7mer-1A	-0.01	40	-0.01	0	N/A
hsa-miR-499a-5p	4717-4723	7mer-m8	-0.02	30	-0.02	0.914	＜0.1
hsa-miR-6773-5p	4732-4738	7mer-m8	-0.1	70	-0.09	0	N/A
hsa-miR-6724-5p	4732-4738	7mer-m8	-0.06	58	-0.05	0	N/A
hsa-miR-296-5p	4732-4738	7mer-1A	-0.04	34	-0.03	0.245	N/A

hsa-miR-5787	4735-4742	8mer	-0.03	53	-0.03	0	N/A
hsa-miR-4505	4735-4742	8mer	-0.03	35	-0.03	0	N/A
hsa-miR-6842-3p	4736-4742	7mer-m8	-0.02	29	-0.02	0	N/A
hsa-miR-3652	4736-4742	7mer-1A	-0.01	17	-0.01	0	N/A
hsa-miR-4430	4736-4742	7mer-1A	-0.01	15	-0.01	0	N/A
hsa-miR-1251-5p	4745-4751	7mer-1A	-0.01	30	-0.01	0.959	N/A
hsa-miR-517-5p	4745-4751	7mer-1A	-0.02	29	-0.02	0.024	N/A
hsa-miR-4684-5p	4746-4752	7mer-1A	-0.01	33	-0.01	0	N/A
hsa-miR-942-5p	4747-4753	7mer-m8	-0.02	38	-0.02	0.024	N/A
hsa-miR-4427	4755-4761	7mer-m8	-0.02	34	-0.02	0	N/A
hsa-miR-4656	4758-4764	7mer-m8	-0.04	35	-0.04	0	N/A
hsa-miR-4446-3p	4759-4765	7mer-m8	-0.03	43	-0.03	0.099	N/A
hsa-miR-4685-5p	4761-4767	7mer-m8	-0.02	40	-0.02	0	N/A
hsa-miR-6837-5p	4761-4767	7mer-m8	-0.02	36	-0.02	0	N/A
hsa-miR-7156-3p	4766-4772	7mer-m8	-0.06	63	-0.05	0	N/A
hsa-miR-1184	4767-4773	7mer-m8	-0.02	56	-0.02	0.099	N/A
hsa-miR-4418	4768-4774	7mer-m8	-0.02	52	-0.02	0	N/A
hsa-miR-509-5p	4768-4774	7mer-m8	-0.02	51	-0.02	0	N/A
hsa-miR-509-3-5p	4768-4774	7mer-m8	-0.02	51	-0.02	0	N/A
hsa-miR-6890-3p	4770-4776	7mer-1A	-0.01	43	-0.01	0	N/A
hsa-miR-668-3p	4772-4778	7mer-1A	-0.01	28	-0.01	0.752	N/A
hsa-miR-219b-5p	4775-4781	7mer-1A	-0.02	24	-0.01	0	N/A
hsa-miR-125a-3p	4779-4786	8mer	-0.03	44	-0.03	0.099	N/A
hsa-miR-3934-5p	4780-4786	7mer-1A	-0.04	70	-0.04	0	N/A
hsa-miR-764	4780-4786	7mer-1A	-0.01	51	-0.01	0	N/A

hsa-miR-4268	4785-4791	7mer-m8	-0.08	77	-0.07	0	N/A
hsa-miR-548e-5p	4790-4796	7mer-1A	-0.01	31	-0.01	0	N/A
hsa-miR-208a-5p	4796-4802	7mer-m8	-0.02	57	-0.02	0.085	N/A
hsa-miR-208b-5p	4796-4802	7mer-m8	-0.02	42	-0.02	0.085	N/A
hsa-miR-498	4800-4806	7mer-m8	-0.02	69	-0.02	0.024	N/A
hsa-miR-27a-5p	4805-4811	7mer-1A	-0.15	73	-0.12	0.085	N/A
hsa-miR-378g	4806-4812	7mer-m8	-0.02	39	-0.02	0	N/A
hsa-miR-7854-3p	4816-4822	7mer-1A	-0.05	47	-0.04	0	N/A
hsa-miR-6134	4816-4822	7mer-1A	-0.01	18	-0.01	0.024	N/A
hsa-miR-7162-3p	4817-4824	8mer	-0.03	67	-0.03	0	N/A
hsa-miR-4649-3p	4818-4824	7mer-1A	-0.01	41	-0.01	0	N/A
hsa-miR-423-3p	4818-4829	non-canonical	N/A	N/A	N/A	0	N/A
hsa-miR-423-3p	4818-4829	non-canonical	N/A	N/A	N/A	0	N/A
hsa-miR-3610	4825-4831	7mer-1A	-0.16	68	-0.14	0	N/A
hsa-miR-6854-3p	4831-4837	7mer-1A	-0.11	52	-0.09	0	N/A
hsa-miR-4756-3p	4839-4845	7mer-m8	-0.02	52	-0.02	0	N/A
hsa-miR-4323	4844-4850	7mer-m8	-0.02	17	-0.02	0	N/A
hsa-miR-5685	4846-4852	7mer-1A	-0.04	51	-0.03	0	N/A
hsa-miR-4690-3p	4846-4852	7mer-1A	-0.01	29	-0.01	0	N/A
hsa-miR-3157-5p	4847-4853	7mer-1A	-0.03	35	-0.02	0	N/A
hsa-miR-6839-3p	4852-4859	8mer	-0.03	43	-0.03	0	N/A
hsa-miR-629-5p	4852-4858	7mer-1A	-0.01	19	-0.01	0	N/A
hsa-miR-3940-5p	4853-4859	7mer-1A	-0.01	42	-0.01	0	N/A

hsa-miR-4507	4853-4859	7mer-1A	-0.01	31	-0.01	0	N/A
hsa-miR-548aq-3p	4868-4875	8mer	-0.03	84	-0.03	0	N/A
hsa-miR-548ah-3p	4868-4875	8mer	-0.03	84	-0.03	0	N/A
hsa-miR-548ae-3p	4868-4875	8mer	-0.03	84	-0.03	0	N/A
hsa-miR-548x-3p	4868-4875	8mer	-0.03	84	-0.03	0	N/A
hsa-miR-548aj-3p	4868-4875	8mer	-0.03	84	-0.03	0	N/A
hsa-miR-548am-3p	4868-4875	8mer	-0.03	84	-0.03	0	N/A
hsa-miR-548j-3p	4868-4875	8mer	-0.03	84	-0.03	0	N/A
hsa-miR-548d-3p	4869-4875	7mer-1A	-0.01	57	-0.01	0	N/A
hsa-miR-548h-3p	4869-4875	7mer-1A	-0.01	57	-0.01	0	N/A
hsa-miR-548ac	4869-4875	7mer-1A	-0.01	57	-0.01	0	N/A
hsa-miR-548bb-3p	4869-4875	7mer-1A	-0.01	57	-0.01	0	N/A
hsa-miR-548z	4869-4875	7mer-1A	-0.01	57	-0.01	0	N/A
hsa-miR-28-5p	4877-4883	7mer-m8	-0.08	72	-0.07	1.354	N/A
hsa-miR-708-5p	4877-4883	7mer-m8	-0.08	72	-0.07	1.354	N/A
hsa-miR-3139	4877-4883	7mer-m8	-0.06	62	-0.05	1.354	N/A
hsa-miR-4768-3p	4879-4885	7mer-1A	-0.05	65	-0.04	0.085	N/A
hsa-miR-4459	4879-4885	7mer-1A	-0.04	64	-0.03	0	N/A
hsa-miR-4433a-3p	4879-4885	7mer-1A	-0.01	39	-0.01	0.024	N/A
hsa-miR-3664-3p	4880-4886	7mer-m8	-0.02	33	-0.02	0	N/A
hsa-miR-6821-3p	4884-4890	7mer-1A	-0.05	53	-0.04	0	N/A
hsa-miR-6790-3p	4884-4890	7mer-1A	-0.04	45	-0.03	0	N/A
hsa-miR-613	4889-4895	7mer-1A	-0.07	67	-0.06	0.984	< 0.1
hsa-miR-206	4889-4895	7mer-1A	-0.03	52	-0.03	0.984	<

							0.1
hsa-miR-1-3p	4889-4895	7mer-1A	-0.03	52	-0.03	0.984	< 0.1
hsa-miR-6505-5p	4890-4896	7mer-1A	-0.01	23	-0.01	0	N/A
hsa-miR-513c-5p	4910-4916	7mer-1A	-0.01	45	-0.01	0.085	N/A
hsa-miR-514b-5p	4910-4916	7mer-1A	-0.01	43	-0.01	0.085	N/A
hsa-miR-3168	4913-4919	7mer-1A	-0.01	37	-0.01	0	N/A
hsa-miR-6874-3p	4913-4919	7mer-1A	-0.01	36	-0.01	0	N/A
hsa-miR-148b-5p	4913-4919	7mer-1A	-0.01	33	-0.01	0	N/A
hsa-miR-5584-3p	4913-4919	7mer-1A	-0.01	32	-0.01	0	N/A
hsa-miR-1244	4915-4921	7mer-m8	-0.02	22	-0.02	0	N/A
hsa-miR-3129-5p	4916-4922	7mer-m8	-0.02	47	-0.02	0	< 0.1
hsa-miR-199b-3p	4916-4922	7mer-m8	-0.02	30	-0.02	0	< 0.1
hsa-miR-199a-3p	4916-4922	7mer-m8	-0.02	30	-0.02	0	< 0.1
hsa-miR-4717-5p	4921-4927	7mer-1A	-0.01	39	-0.01	0	N/A
hsa-miR-15a-3p	4921-4927	7mer-1A	-0.01	28	-0.01	0	N/A
hsa-miR-5704	4921-4927	7mer-m8	-0.02	2	-0.02	0	N/A
hsa-miR-4474-3p	4934-4941	8mer	-0.08	65	-0.07	0	N/A
hsa-miR-7108-5p	4935-4941	7mer-1A	-0.01	41	-0.01	0	N/A
hsa-miR-4716-5p	4944-4950	7mer-1A	-0.01	24	-0.01	0	N/A
hsa-miR-3691-3p	4949-4956	8mer	-0.03	60	-0.03	0	N/A
hsa-miR-5695	4951-4957	7mer-m8	-0.02	38	-0.02	0	N/A

hsa-miR-6836-3p	4954-4961	8mer	-0.18	87	-0.15	0	N/A
hsa-miR-6791-3p	4954-4960	7mer-1A	-0.04	60	-0.04	0	N/A
hsa-miR-6829-3p	4954-4960	7mer-1A	-0.03	52	-0.03	0	N/A
hsa-miR-6778-3p	4954-4960	7mer-1A	-0.01	24	-0.01	0	N/A
hsa-miR-3152-5p	4955-4962	8mer	-0.1	72	-0.09	0	N/A
hsa-miR-4774-3p	4956-4962	7mer-1A	-0.01	13	-0.01	0	N/A
hsa-miR-298	4963-4969	7mer-1A	-0.01	24	-0.01	0.099	N/A
hsa-miR-3158-5p	4964-4970	7mer-m8	-0.02	43	-0.02	0.364	N/A
hsa-miR-580-3p	4980-4986	7mer-m8	-0.02	51	-0.02	0.024	N/A
hsa-miR-4251	4981-4987	7mer-m8	-0.02	36	-0.02	0	N/A
hsa-miR-4324	4982-4988	7mer-m8	-0.02	35	-0.02	0	N/A
hsa-miR-1245b-5p	4986-4992	7mer-1A	-0.06	69	-0.05	0	N/A
hsa-miR-3142	4986-4992	7mer-1A	-0.04	52	-0.03	0	N/A
hsa-miR-645	4988-4994	7mer-1A	-0.04	40	-0.04	0.024	N/A
hsa-miR-1251-5p	4990-4996	7mer-1A	-0.01	30	-0.01	0.61	N/A
hsa-miR-517-5p	4990-4996	7mer-1A	-0.01	15	-0.01	0.099	N/A
hsa-miR-200c-5p	4999-5005	7mer-1A	-0.01	24	-0.01	0	N/A
hsa-miR-550a-3p	4999-5005	7mer-1A	-0.01	22	-0.01	0	N/A
hsa-miR-4711-3p	5000-5007	8mer	-0.03	41	-0.03	0	N/A
hsa-miR-592	5002-5008	7mer-1A	-0.08	65	-0.07	0.099	N/A
hsa-miR-5010-3p	5003-5009	7mer-m8	-0.02	49	-0.02	0	N/A
hsa-miR-6844	5005-5011	7mer-m8	-0.02	49	-0.02	0	N/A
hsa-miR-3689d	5011-5017	7mer-1A	-0.17	88	-0.14	0	N/A
hsa-miR-6851-5p	5011-5017	7mer-1A	-0.21	86	-0.18	0	N/A
hsa-miR-7847-3p	5012-5019	8mer	-0.12	66	-0.1	0	N/A

hsa-miR-4491	5014-5020	7mer-m8	-0.02	17	-0.02	0	N/A
hsa-miR-4657	5014-5020	7mer-m8	-0.02	16	-0.02	0	N/A
hsa-miR-576-3p	5016-5022	7mer-m8	-0.02	35	-0.02	0.024	N/A
hsa-miR-5683	5018-5024	7mer-m8	-0.02	42	-0.02	0	N/A
hsa-miR-6787-3p	5026-5032	7mer-1A	-0.01	41	-0.01	0	N/A
hsa-miR-4522	5030-5036	7mer-1A	-0.02	24	-0.02	0	N/A
hsa-miR-526b-5p	5034-5040	7mer-1A	-0.01	34	-0.01	0.099	N/A
hsa-miR-26b-3p	5044-5050	7mer-1A	-0.02	43	-0.02	0.085	N/A
hsa-miR-3664-5p	5046-5052	7mer-m8	-0.02	35	-0.02	0	N/A
hsa-miR-4261	5051-5057	7mer-1A	-0.06	57	-0.05	0.437	N/A
hsa-miR-3688-3p	5052-5058	7mer-m8	-0.02	29	-0.02	0.437	N/A
hsa-miR-490-5p	5054-5060	7mer-1A	-0.06	68	-0.05	0.085	N/A
hsa-miR-205-5p	5057-5063	7mer-m8	-0.02	44	-0.02	0.216	＜0.1
hsa-miR-589-5p	5062-5068	7mer-m8	-0.04	62	-0.03	0.099	N/A
hsa-miR-4516	5064-5070	7mer-1A	-0.01	45	-0.01	0	N/A
hsa-miR-4531	5064-5071	8mer	-0.03	36	-0.03	0	N/A
hsa-miR-4434	5064-5070	7mer-1A	-0.01	33	-0.01	0	N/A
hsa-miR-5703	5064-5070	7mer-1A	-0.01	31	-0.01	0	N/A
hsa-miR-620	5065-5071	7mer-1A	-0.01	31	-0.01	0	N/A
hsa-miR-1270	5065-5071	7mer-1A	-0.01	30	-0.01	0	N/A
hsa-miR-432-5p	5066-5072	7mer-m8	-0.02	34	-0.02	0.099	N/A
hsa-miR-636	5069-5075	7mer-m8	-0.02	31	-0.02	0.085	N/A
hsa-miR-302c-3p.2	5070-5077	8mer	-0.03	55	-0.03	1.422	0.12

hsa-miR-520f-3p	5070-5077	8mer	-0.03	53	-0.03	1.422	0.1<br>2
hsa-miR-512-3p	5071-5077	7mer-1A	-0.01	24	-0.01	0.099	N/A
hsa-miR-4473	5071-5077	7mer-m8	-0.02	13	-0.02	0	N/A
hsa-miR-6080	5072-5079	8mer	-0.1	74	-0.08	0	N/A
hsa-miR-5088-3p	5077-5084	8mer	-0.04	55	-0.03	0	N/A
hsa-miR-4287	5079-5086	8mer	-0.03	59	-0.03	0	N/A
hsa-miR-4685-3p	5079-5086	8mer	-0.03	57	-0.03	0	N/A
hsa-miR-4469	5080-5086	7mer-1A	-0.01	42	-0.01	0	N/A
hsa-miR-6867-3p	5080-5086	7mer-m8	-0.02	32	-0.02	0	N/A
hsa-miR-7113-3p	5080-5086	7mer-1A	-0.01	30	-0.01	0	N/A
hsa-miR-4639-3p	5083-5089	7mer-m8	-0.08	79	-0.07	0.085	N/A
hsa-miR-7702	5089-5095	7mer-1A	-0.02	32	-0.02	0	N/A
hsa-miR-1305	5101-5108	8mer	-0.03	85	-0.03	0	N/A
hsa-miR-3140-3p	5105-5111	7mer-1A	-0.01	29	-0.01	0	N/A
hsa-miR-6885-3p	5107-5113	7mer-m8	-0.02	46	-0.02	0	N/A
hsa-miR-3121-5p	5109-5116	8mer	-0.16	89	-0.13	0	N/A
hsa-miR-6083	5118-5124	7mer-1A	-0.01	45	-0.01	0	N/A
hsa-miR-583	5132-5138	7mer-m8	-0.02	58	-0.02	0.099	N/A
hsa-miR-3978	5135-5141	7mer-m8	-0.02	64	-0.02	0	N/A
hsa-miR-3688-3p	5136-5142	7mer-m8	-0.02	29	-0.02	0	N/A
hsa-miR-4694-3p	5138-5144	7mer-m8	-0.02	42	-0.02	0	N/A
hsa-miR-5093	5140-5146	7mer-m8	-0.02	40	-0.02	0	N/A
hsa-miR-4496	5142-5148	7mer-1A	-0.01	64	-0.01	0	N/A
hsa-miR-4311	5156-5163	8mer	-0.03	78	-0.03	0	N/A

hsa-miR-1276	5157-5163	7mer-1A	-0.01	54	-0.01	0	N/A
hsa-miR-583	5157-5163	7mer-1A	-0.01	49	-0.01	0.085	N/A
hsa-miR-554	5172-5178	7mer-m8	-0.12	78	-0.1	0.099	N/A
hsa-miR-3923	5174-5180	7mer-1A	-0.1	62	-0.08	0	N/A
hsa-miR-556-3p	5178-5184	7mer-1A	-0.01	38	-0.01	0.024	N/A
hsa-miR-656-3p	5188-5194	7mer-m8	-0.02	56	-0.02	0.099	N/A
hsa-miR-6507-5p	5192-5198	7mer-m8	-0.02	59	-0.02	0	N/A
hsa-miR-451b	5195-5201	7mer-m8	-0.03	46	-0.02	0	N/A
hsa-miR-195-5p	5198-5204	7mer-m8	-0.15	80	-0.12	1.266	0.13
hsa-miR-16-5p	5198-5204	7mer-m8	-0.15	80	-0.12	1.266	0.13
hsa-miR-15a-5p	5198-5204	7mer-m8	-0.09	69	-0.08	1.266	0.13
hsa-miR-15b-5p	5198-5204	7mer-m8	-0.08	66	-0.07	1.266	0.13
hsa-miR-424-5p	5198-5204	7mer-m8	-0.07	63	-0.06	1.266	0.13
hsa-miR-6838-5p	5198-5204	7mer-m8	-0.06	57	-0.05	1.266	0.13
hsa-miR-497-5p	5198-5204	7mer-m8	-0.04	53	-0.04	1.266	0.13
hsa-miR-6792-5p	5200-5206	7mer-m8	-0.02	48	-0.02	0	N/A
hsa-miR-1207-3p	5209-5215	7mer-1A	-0.01	34	-0.01	0.024	N/A
hsa-miR-513b-3p	5213-5219	7mer-m8	-0.02	25	-0.02	0	N/A
hsa-miR-196a-3p	5219-5225	7mer-m8	-0.05	40	-0.05	0	N/A

hsa-miR-3616-3p	5222-5228	7mer-m8	-0.14	67	-0.12	0	N/A
hsa-miR-6785-5p	5224-5230	7mer-m8	-0.13	77	-0.11	0	N/A
hsa-miR-6883-5p	5224-5230	7mer-m8	-0.11	70	-0.09	0	N/A
hsa-miR-4728-5p	5224-5230	7mer-m8	-0.12	70	-0.1	0	N/A
hsa-miR-149-3p	5224-5230	7mer-m8	-0.08	60	-0.07	0	N/A
hsa-miR-1321	5225-5232	8mer	-0.05	56	-0.05	0	N/A
hsa-miR-4756-5p	5225-5232	8mer	-0.03	45	-0.03	0	N/A
hsa-miR-4739	5225-5232	8mer	-0.03	40	-0.03	0	N/A
hsa-miR-3162-5p	5226-5233	8mer	-0.03	24	-0.03	0	N/A
hsa-miR-6760-5p	5226-5232	7mer-1A	-0.02	22	-0.01	0	N/A
hsa-miR-2909	5228-5234	7mer-1A	-0.01	34	-0.01	0	N/A
hsa-miR-656-5p	5235-5241	7mer-1A	-0.12	72	-0.11	0	N/A
hsa-miR-890	5239-5245	7mer-1A	-0.05	53	-0.04	0.099	N/A
hsa-miR-548an	5246-5252	7mer-1A	-0.12	81	-0.1	0	N/A
hsa-miR-4484	5246-5252	7mer-1A	-0.07	76	-0.06	0	N/A
hsa-miR-6768-3p	5246-5252	7mer-1A	-0.01	43	-0.01	0	N/A
hsa-miR-4668-5p	5253-5259	7mer-1A	-0.14	83	-0.12	0	N/A
hsa-miR-6739-5p	5253-5259	7mer-1A	-0.08	74	-0.07	0	N/A
hsa-miR-3153	5253-5259	7mer-1A	-0.04	73	-0.03	0	N/A
hsa-miR-6733-5p	5253-5259	7mer-1A	-0.07	71	-0.06	0	N/A
hsa-miR-4654	5255-5261	7mer-1A	-0.18	88	-0.15	0.024	N/A
hsa-miR-4769-5p	5255-5261	7mer-1A	-0.12	85	-0.1	0.024	N/A
hsa-miR-4648	5255-5261	7mer-1A	-0.08	71	-0.06	0	N/A
hsa-miR-1233-5p	5255-5261	7mer-1A	-0.11	58	-0.1	0	N/A
hsa-miR-6778-5p	5255-5261	7mer-1A	-0.11	57	-0.1	0	N/A

hsa-miR-134-3p	5256-5262	7mer-m8	-0.13	81	-0.11	0.085	N/A
hsa-miR-4318	5257-5263	7mer-m8	-0.07	67	-0.06	0	N/A
hsa-miR-6760-3p	5259-5265	7mer-1A	-0.01	39	-0.01	0	N/A
hsa-miR-1208	5259-5265	7mer-1A	-0.01	32	-0.01	0.024	N/A
hsa-miR-653-3p	5260-5266	7mer-1A	-0.04	67	-0.04	0.085	N/A
hsa-miR-181b-2-3p	5260-5266	7mer-1A	-0.05	65	-0.04	0	N/A
hsa-miR-181b-3p	5260-5266	7mer-1A	-0.05	65	-0.04	0	N/A
hsa-miR-4420	5260-5266	7mer-1A	-0.01	42	-0.01	0	N/A
hsa-miR-3606-3p	5261-5272	non-canonical	N/A	N/A	N/A	0	N/A
hsa-miR-3606-3p	5261-5272	non-canonical	N/A	N/A	N/A	0	N/A
hsa-miR-15a-3p	5276-5282	7mer-1A	-0.02	43	-0.02	0	N/A
hsa-miR-4717-5p	5276-5282	7mer-1A	-0.01	39	-0.01	0	N/A
hsa-miR-5704	5276-5282	7mer-m8	-0.04	12	-0.04	0	N/A
hsa-miR-1295b-3p	5277-5283	7mer-m8	-0.14	76	-0.12	0	N/A
hsa-miR-379-3p	5286-5292	7mer-m8	-0.04	81	-0.04	1.438	N/A
hsa-miR-411-3p	5286-5292	7mer-m8	-0.04	81	-0.04	1.438	N/A
hsa-miR-2054	5291-5297	7mer-1A	-0.01	46	-0.01	0	N/A
hsa-miR-5001-3p	5299-5305	7mer-m8	-0.13	82	-0.11	0	N/A
hsa-miR-4635	5307-5313	7mer-1A	-0.06	80	-0.05	0	N/A
hsa-miR-6881-3p	5310-5316	7mer-m8	-0.04	54	-0.03	0	N/A
hsa-miR-877-3p	5310-5316	7mer-1A	-0.01	28	-0.01	0	N/A
hsa-miR-888-5p	5317-5323	7mer-1A	-0.07	69	-0.06	0	N/A

hsa-miR-550b-3p	5319-5325	7mer-m8	-0.1	83	-0.09	0	N/A
hsa-miR-606	5324-5330	7mer-m8	-0.02	28	-0.02	0.024	N/A
hsa-miR-578	5348-5354	7mer-m8	-0.02	50	-0.02	0.085	N/A
hsa-miR-4452	5352-5359	8mer	-0.12	92	-0.1	0	N/A
hsa-miR-183-3p	5353-5359	7mer-1A	-0.01	43	-0.01	0.085	N/A
hsa-miR-4635	5355-5361	7mer-1A	-0.07	84	-0.06	0	N/A
hsa-miR-526b-5p	5356-5362	7mer-1A	-0.03	53	-0.03	0.099	N/A
hsa-miR-4778-3p	5358-5364	7mer-1A	-0.01	32	-0.01	0.437	N/A
hsa-miR-877-3p	5360-5367	8mer	-0.09	73	-0.08	0	N/A
hsa-miR-6881-3p	5361-5367	7mer-m8	-0.02	37	-0.02	0	N/A
hsa-miR-4422	5371-5378	8mer	-0.03	72	-0.03	0	N/A
hsa-miR-6835-3p	5372-5378	7mer-1A	-0.01	55	-0.01	2.373	N/A
hsa-miR-9-3p	5373-5379	7mer-1A	-0.07	73	-0.06	0.085	N/A
hsa-miR-302a-5p	5375-5381	7mer-1A	-0.01	50	-0.01	0.312	N/A
hsa-miR-33a-3p	5381-5387	7mer-m8	-0.02	49	-0.02	0	N/A
hsa-miR-7-1-3p	5385-5392	8mer	-0.03	87	-0.03	0	N/A
hsa-miR-7-2-3p	5385-5392	8mer	-0.03	87	-0.03	0	N/A
hsa-miR-495-3p	5386-5392	7mer-1A	-0.01	63	-0.01	0.345	N/A
hsa-miR-5688	5386-5392	7mer-1A	-0.01	62	-0.01	0.345	N/A
hsa-miR-568	5390-5396	7mer-m8	-0.02	25	-0.02	0.099	N/A
hsa-miR-206	5394-5400	7mer-m8	-0.12	79	-0.1	0.058	< 0.1
hsa-miR-1-3p	5394-5400	7mer-m8	-0.12	79	-0.1	0.058	< 0.1
hsa-miR-613	5394-5400	7mer-m8	-0.1	74	-0.08	0.058	<

								0.1
hsa-miR-3928-3p	5397-5403	7mer-1A	-0.01	50	-0.01	0	N/A
hsa-miR-3915	5397-5403	7mer-m8	-0.03	36	-0.03	0	N/A
hsa-miR-1294	5399-5405	7mer-1A	-0.13	80	-0.11	0.024	N/A
hsa-miR-4316	5399-5405	7mer-1A	-0.01	35	-0.01	0	N/A
hsa-miR-758-3p	5401-5407	7mer-1A	-0.01	34	-0.01	0.514	N/A
hsa-miR-4726-3p	5412-5419	8mer	-0.22	96	-0.19	0	N/A
hsa-miR-6840-3p	5413-5419	7mer-1A	-0.12	93	-0.1	0	N/A
hsa-miR-1915-3p	5413-5419	7mer-1A	-0.09	82	-0.08	0.67	N/A
hsa-miR-6764-5p	5413-5419	7mer-1A	-0.08	79	-0.07	0.67	N/A
hsa-miR-4733-5p	5415-5421	7mer-1A	-0.09	79	-0.08	0	N/A
hsa-miR-376c-3p	5426-5432	7mer-1A	-0.06	54	-0.05	0	N/A
hsa-miR-221-3p	5428-5435	8mer	-0.03	33	-0.03	0	＜ 0.1
hsa-miR-222-3p	5428-5435	8mer	-0.03	33	-0.03	0	＜ 0.1
hsa-miR-548v	5429-5435	7mer-1A	-0.08	55	-0.07	0	N/A
hsa-miR-187-5p	5429-5435	7mer-1A	-0.03	39	-0.02	0.085	N/A
hsa-miR-6894-3p	5433-5439	7mer-m8	-0.13	86	-0.11	0.085	N/A
hsa-miR-4763-5p	5433-5439	7mer-1A	-0.07	47	-0.06	0	N/A
hsa-miR-6890-3p	5436-5442	7mer-m8	-0.08	84	-0.07	0	N/A
hsa-miR-603	5439-5445	7mer-m8	-0.02	54	-0.02	0.085	N/A
hsa-miR-570-3p	5443-5449	7mer-m8	-0.02	25	-0.02	0	N/A
hsa-miR-651-3p	5446-5452	7mer-m8	-0.02	37	-0.02	0	N/A
hsa-miR-1252-5p	5447-5453	7mer-m8	-0.02	20	-0.02	0.024	N/A

hsa-miR-4533	5448-5455	8mer	-0.03	42	-0.03	0	N/A
hsa-miR-3202	5449-5455	7mer-1A	-0.01	29	-0.01	0	N/A
hsa-miR-4802-3p	5452-5458	7mer-m8	-0.05	52	-0.04	0	N/A
hsa-miR-2467-3p	5461-5467	7mer-1A	-0.07	63	-0.06	0	N/A
hsa-miR-3678-3p	5461-5467	7mer-m8	-0.02	38	-0.02	0	N/A
hsa-miR-3666	5464-5470	7mer-1A	-0.03	51	-0.03	0.221	<0.1
hsa-miR-4295	5464-5470	7mer-1A	-0.01	32	-0.01	0.221	<0.1
hsa-miR-130a-3p	5464-5470	7mer-1A	-0.01	32	-0.01	0.221	<0.1
hsa-miR-301b-3p	5464-5470	7mer-1A	-0.01	31	-0.01	0.221	<0.1
hsa-miR-301a-3p	5464-5470	7mer-1A	-0.01	30	-0.01	0.221	<0.1
hsa-miR-130b-3p	5464-5470	7mer-1A	-0.01	30	-0.01	0.221	<0.1
hsa-miR-454-3p	5464-5470	7mer-1A	-0.01	27	-0.01	0.221	<0.1
hsa-miR-4671-3p	5464-5470	7mer-m8	-0.02	5	-0.02	0	N/A
hsa-miR-196a-5p	5467-5473	7mer-m8	-0.02	26	-0.02	0.058	<0.1
hsa-miR-196b-5p	5467-5473	7mer-m8	-0.02	26	-0.02	0.058	<0.1
hsa-miR-3665	5470-5476	7mer-1A	-0.1	63	-0.09	0	N/A
hsa-miR-657	5470-5476	7mer-1A	-0.01	36	-0.01	0.024	N/A

hsa-miR-1205	5471-5477	7mer-m8	-0.03	63	-0.03	0.024	N/A
hsa-miR-4418	5472-5478	7mer-m8	-0.02	52	-0.02	0	N/A
hsa-miR-509-5p	5472-5478	7mer-m8	-0.02	51	-0.02	0	N/A
hsa-miR-509-3-5p	5472-5478	7mer-m8	-0.02	51	-0.02	0	N/A
hsa-miR-603	5476-5482	7mer-m8	-0.02	54	-0.02	0.085	N/A
hsa-miR-1197	5479-5485	7mer-m8	-0.02	52	-0.02	0.49	N/A
hsa-miR-5004-5p	5480-5486	7mer-m8	-0.02	13	-0.02	0	N/A
hsa-miR-6847-5p	5482-5489	8mer	-0.03	45	-0.03	0	N/A
hsa-miR-4257	5483-5489	7mer-1A	-0.03	62	-0.03	0	N/A
hsa-miR-7156-3p	5490-5497	8mer	-0.2	89	-0.17	0	N/A
hsa-miR-1301-3p	5491-5497	7mer-1A	-0.14	83	-0.12	0	N/A
hsa-miR-5047	5491-5497	7mer-1A	-0.08	71	-0.07	0	N/A
hsa-miR-499b-5p	5496-5502	7mer-m8	-0.02	37	-0.02	0	N/A
hsa-miR-1197	5501-5507	7mer-1A	-0.01	41	-0.01	0.043	N/A
hsa-miR-3942-3p	5507-5513	7mer-m8	-0.02	55	-0.02	0	N/A
hsa-miR-5100	5507-5513	7mer-1A	-0.01	38	-0.01	0	N/A
hsa-miR-892c-5p	5508-5514	7mer-m8	-0.02	32	-0.02	0.099	N/A
hsa-miR-6716-5p	5513-5519	7mer-1A	-0.1	65	-0.09	0	N/A
hsa-miR-204-3p	5514-5520	7mer-m8	-0.02	50	-0.02	0	N/A
hsa-miR-4646-5p	5514-5520	7mer-m8	-0.02	33	-0.02	0	N/A
hsa-miR-4731-5p	5516-5522	7mer-1A	-0.05	58	-0.05	0	N/A
hsa-miR-5589-5p	5516-5522	7mer-1A	-0.01	37	-0.01	0	N/A
hsa-miR-3692-5p	5517-5524	8mer	-0.17	89	-0.14	0	N/A
hsa-miR-93-3p	5518-5524	7mer-1A	-0.1	77	-0.08	0.085	N/A
hsa-miR-6071	5518-5525	8mer	-0.03	45	-0.03	0	N/A

hsa-miR-6828-3p	5519-5525	7mer-1A	-0.01	33	-0.01	0	N/A
hsa-miR-103a-2-5p	5522-5528	7mer-1A	-0.01	21	-0.01	0.723	N/A
hsa-miR-302c-3p.2	5524-5531	8mer	-0.03	55	-0.03	0.383	＜0.1
hsa-miR-520f-3p	5524-5531	8mer	-0.03	53	-0.03	0.383	＜0.1
hsa-miR-512-3p	5525-5531	7mer-1A	-0.07	71	-0.06	0.099	N/A
hsa-miR-4473	5525-5532	8mer	-0.16	70	-0.14	0	N/A
hsa-miR-4671-3p	5526-5532	7mer-1A	-0.11	54	-0.09	0	N/A
hsa-miR-103a-2-5p	5531-5537	7mer-m8	-0.08	70	-0.07	0.085	N/A
hsa-miR-4633-3p	5535-5541	7mer-1A	-0.14	78	-0.12	0	N/A
hsa-miR-6500-5p	5535-5541	7mer-1A	-0.13	76	-0.11	0	N/A
hsa-miR-6874-5p	5535-5541	7mer-m8	-0.08	62	-0.07	0	N/A
hsa-miR-6880-5p	5537-5543	7mer-m8	-0.1	59	-0.08	0	N/A
hsa-miR-6745	5538-5544	7mer-m8	-0.11	67	-0.09	0.085	N/A
hsa-miR-363-5p	5538-5544	7mer-m8	-0.09	63	-0.08	0.085	N/A
hsa-miR-6800-5p	5547-5553	7mer-m8	-0.03	29	-0.03	0	N/A
hsa-miR-4686	5553-5560	8mer	-0.14	82	-0.12	0	N/A
hsa-miR-2355-5p	5567-5573	7mer-1A	-0.14	80	-0.12	0	N/A
hsa-miR-3679-3p	5568-5574	7mer-1A	-0.1	72	-0.09	0	N/A
hsa-miR-4446-5p	5569-5575	7mer-1A	-0.01	28	-0.01	0	N/A
hsa-miR-502-3p	5585-5591	7mer-1A	-0.09	74	-0.08	0.123	N/A
hsa-miR-501-3p	5585-5591	7mer-1A	-0.09	74	-0.08	0.123	N/A

hsa-miR-675-3p	5587-5593	7mer-m8	-0.13	85	-0.11	0.085	N/A
hsa-miR-203a-3p.1	5591-5597	7mer-m8	-0.02	50	-0.02	0.497	＜0.1
hsa-miR-5680	5592-5598	7mer-m8	-0.02	68	-0.02	0	N/A
hsa-miR-324-5p	5599-5605	7mer-1A	-0.11	68	-0.1	0.154	N/A
hsa-miR-5700	5601-5607	7mer-m8	-0.02	48	-0.02	0	N/A
hsa-miR-145-3p	5627-5633	7mer-m8	-0.03	53	-0.03	0.085	N/A
hsa-miR-3159	5630-5636	7mer-1A	-0.01	15	-0.01	0	N/A
hsa-miR-3926	5645-5652	8mer	-0.08	65	-0.07	0	N/A
hsa-miR-548s	5646-5652	7mer-m8	-0.07	67	-0.06	0	N/A
hsa-miR-7108-5p	5648-5655	8mer	-0.24	95	-0.2	0	N/A
hsa-miR-663b	5648-5654	7mer-1A	-0.03	51	-0.03	0.024	N/A
hsa-miR-4474-3p	5649-5655	7mer-1A	-0.08	65	-0.07	0	N/A
hsa-miR-2113	5651-5657	7mer-1A	-0.01	42	-0.01	0	N/A
hsa-miR-5010-3p	5651-5657	7mer-1A	-0.01	31	-0.01	0	N/A
hsa-miR-451b	5662-5668	7mer-m8	-0.1	80	-0.08	0	N/A
hsa-miR-3120-3p	5664-5671	8mer	-0.06	69	-0.05	0.335	N/A
hsa-let-7a-2-3p	5667-5674	8mer	-0.07	70	-0.06	0.085	N/A
hsa-let-7g-3p	5667-5674	8mer	-0.06	69	-0.05	0.085	N/A
hsa-let-7c-3p	5668-5674	7mer-1A	-0.02	42	-0.02	0.085	N/A
hsa-miR-493-5p	5668-5674	7mer-1A	-0.01	31	-0.01	0.213	N/A
hsa-miR-508-3p	5675-5681	7mer-m8	-0.09	60	-0.07	0.024	N/A
hsa-miR-5186	5677-5683	7mer-m8	-0.07	69	-0.06	0	N/A
hsa-miR-4775	5688-5694	7mer-1A	-0.01	30	-0.01	0	N/A
hsa-miR-4661-3p	5697-5703	7mer-1A	-0.01	26	-0.01	0	N/A

hsa-miR-4762-5p	5706-5712	7mer-1A	-0.01	30	-0.01	0	N/A
hsa-miR-1306-5p	5725-5731	7mer-1A	-0.04	56	-0.03	1.567	<0.1
hsa-miR-6879-3p	5727-5733	7mer-1A	-0.02	33	-0.02	0	N/A
hsa-miR-3610	5740-5746	7mer-1A	-0.17	70	-0.15	0	N/A
hsa-miR-2053	5743-5750	8mer	-0.03	83	-0.03	0	N/A
hsa-miR-569	5743-5749	7mer-1A	-0.01	25	-0.01	0.024	N/A
hsa-miR-1267	5748-5754	7mer-1A	-0.14	82	-0.12	0.024	N/A
hsa-miR-367-5p	5748-5754	7mer-1A	-0.13	80	-0.11	0	N/A
hsa-miR-3120-5p	5751-5757	7mer-m8	-0.1	79	-0.08	0	N/A
hsa-miR-626	5752-5759	8mer	-0.25	89	-0.21	0.024	N/A
hsa-miR-6876-3p	5752-5759	8mer	-0.25	89	-0.21	0.024	N/A
hsa-miR-4670-3p	5761-5767	7mer-m8	-0.04	46	-0.03	0	N/A
hsa-miR-548ad-3p	5766-5773	8mer	-0.03	46	-0.03	0	N/A
hsa-miR-424-3p	5767-5773	7mer-1A	-0.11	79	-0.1	0	N/A
hsa-miR-4799-5p	5770-5776	7mer-1A	-0.01	35	-0.01	0	N/A
hsa-miR-4263	5771-5777	7mer-1A	-0.01	40	-0.01	0	N/A
hsa-miR-576-5p	5771-5777	7mer-1A	-0.01	37	-0.01	0	N/A
hsa-miR-33a-3p	5775-5781	7mer-m8	-0.02	49	-0.02	0	N/A
hsa-miR-1273e	5781-5788	8mer	-0.11	73	-0.09	0	N/A
hsa-miR-320d	5790-5796	7mer-1A	-0.04	70	-0.03	1.192	N/A
hsa-miR-320c	5790-5796	7mer-1A	-0.04	69	-0.03	1.192	N/A
hsa-miR-320b	5790-5796	7mer-1A	-0.04	69	-0.03	1.192	N/A
hsa-miR-320a	5790-5796	7mer-1A	-0.04	69	-0.03	1.192	N/A
hsa-miR-4429	5790-5796	7mer-1A	-0.03	63	-0.02	1.192	N/A

hsa-miR-9-3p	5790-5796	7mer-m8	-0.04	60	-0.04	0.65	N/A
hsa-miR-340-5p	5792-5798	7mer-m8	-0.02	76	-0.02	1.278	N/A
hsa-miR-142-5p	5792-5798	7mer-1A	-0.01	44	-0.01	1.278	< 0.1
hsa-miR-5590-3p	5792-5798	7mer-1A	-0.01	41	-0.01	1.278	< 0.1
hsa-miR-127-5p	5798-5805	8mer	-0.13	84	-0.11	0	N/A
hsa-miR-6806-3p	5798-5804	7mer-1A	-0.05	68	-0.04	0	N/A
hsa-miR-3928-5p	5798-5804	7mer-1A	-0.03	56	-0.02	0	N/A
hsa-miR-7843-3p	5799-5805	7mer-1A	-0.03	48	-0.02	0	N/A
hsa-miR-4781-3p	5803-5809	7mer-1A	-0.09	83	-0.08	0	N/A
hsa-miR-424-3p	5814-5821	8mer	-0.11	79	-0.09	0	N/A
hsa-miR-548ad-3p	5815-5821	7mer-1A	-0.03	48	-0.03	0	N/A
hsa-miR-4761-5p	5822-5828	7mer-1A	-0.1	74	-0.08	0	N/A
hsa-miR-561-5p	5823-5829	7mer-1A	-0.19	88	-0.16	0	N/A
hsa-miR-498	5824-5830	7mer-1A	-0.01	48	-0.01	0.099	N/A
hsa-miR-5582-3p	5829-5835	7mer-m8	-0.02	65	-0.02	0.335	N/A
hsa-miR-1323	5830-5836	7mer-m8	-0.02	52	-0.02	0.099	N/A
hsa-miR-548o-3p	5830-5836	7mer-m8	-0.02	51	-0.02	0.099	N/A
hsa-miR-548aw	5832-5839	8mer	-0.03	73	-0.03	0	N/A
hsa-miR-548f-5p	5832-5838	7mer-1A	-0.01	35	-0.01	0	N/A
hsa-miR-548g-5p	5832-5838	7mer-1A	-0.01	35	-0.01	0	N/A
hsa-miR-548aj-5p	5832-5838	7mer-1A	-0.01	35	-0.01	0	N/A
hsa-miR-548x-5p	5832-5838	7mer-1A	-0.01	35	-0.01	0	N/A
hsa-miR-1468-3p	5832-5838	7mer-1A	-0.01	26	-0.01	0	N/A

hsa-miR-527	5833-5839	7mer-1A	-0.01	44	-0.01	0.024	N/A
hsa-miR-518a-5p	5833-5839	7mer-1A	-0.01	44	-0.01	0.024	N/A
hsa-miR-4317	5836-5842	7mer-1A	-0.05	61	-0.05	0	N/A
hsa-miR-3160-5p	5844-5850	7mer-1A	-0.01	25	-0.01	0	N/A
hsa-miR-429	5849-5855	7mer-m8	-0.02	52	-0.02	1.508	0.11
hsa-miR-200b-3p	5849-5855	7mer-m8	-0.02	52	-0.02	1.508	0.11
hsa-miR-200c-3p	5849-5855	7mer-m8	-0.02	52	-0.02	1.508	0.11
hsa-let-7c-3p	5855-5862	8mer	-0.08	75	-0.07	1.288	N/A
hsa-let-7g-3p	5856-5862	7mer-1A	-0.12	84	-0.1	1.288	N/A
hsa-let-7a-2-3p	5856-5862	7mer-1A	-0.09	78	-0.08	1.288	N/A
hsa-miR-664b-3p	5860-5867	8mer	-0.03	78	-0.03	0.099	N/A
hsa-miR-579-3p	5860-5867	8mer	-0.03	76	-0.03	0.099	N/A
hsa-miR-5696	5861-5867	7mer-1A	-0.01	34	-0.01	0	N/A
hsa-miR-335-3p	5863-5869	7mer-m8	-0.02	61	-0.02	0.085	N/A
hsa-miR-3140-3p	5866-5872	7mer-1A	-0.01	36	-0.01	0	N/A
hsa-miR-6071	5869-5875	7mer-1A	-0.12	80	-0.11	0	N/A
hsa-miR-6828-3p	5869-5875	7mer-1A	-0.07	70	-0.06	0	N/A
hsa-miR-3123	5875-5881	7mer-m8	-0.02	58	-0.02	0	N/A
hsa-miR-4311	5877-5883	7mer-m8	-0.02	52	-0.02	0	N/A
hsa-miR-3163	5881-5887	7mer-1A	-0.01	45	-0.01	0	N/A
hsa-miR-497-3p	5888-5894	7mer-1A	-0.01	55	-0.01	0.085	N/A
hsa-miR-4709-5p	5896-5903	8mer	-0.13	89	-0.11	0	N/A

hsa-miR-3192-3p	5902-5909	8mer	-0.18	93	-0.15	0	N/A
hsa-miR-3151-3p	5902-5908	7mer-1A	-0.07	64	-0.06	0	N/A
hsa-miR-4762-3p	5903-5910	8mer	-0.03	63	-0.03	0	N/A
hsa-miR-888-5p	5924-5930	7mer-1A	-0.12	85	-0.1	0.085	N/A
hsa-miR-200c-3p	5927-5933	7mer-1A	-0.03	63	-0.02	1.794	0.1 4
hsa-miR-200b-3p	5927-5933	7mer-1A	-0.03	62	-0.02	1.794	0.1 4
hsa-miR-8084	5927-5933	7mer-1A	-0.02	54	-0.01	0	N/A
hsa-miR-429	5927-5933	7mer-1A	-0.02	49	-0.01	1.794	0.1 4
hsa-miR-374c-5p	5928-5934	7mer-1A	-0.05	52	-0.04	2.013	N/A
hsa-miR-655-3p	5928-5934	7mer-1A	-0.03	42	-0.03	2.013	N/A
hsa-miR-4775	5935-5941	7mer-m8	-0.02	47	-0.02	0	N/A
hsa-miR-590-3p	5935-5941	7mer-1A	-0.01	34	-0.01	0.099	N/A
hsa-miR-4735-5p	5936-5943	8mer	-0.06	85	-0.05	0	N/A
hsa-miR-1277-5p	5946-5952	7mer-1A	-0.01	40	0	0	N/A
hsa-miR-5692c	5947-5953	7mer-m8	-0.02	58	0	0	N/A
hsa-miR-5692b	5947-5953	7mer-m8	-0.02	58	0	0	N/A
hsa-miR-3133	5959-5966	8mer	-0.03	87	0	0	N/A
hsa-miR-186-5p	5960-5966	7mer-1A	-0.01	59	0	2.948	N/A
hsa-miR-4433a-3p	5966-5973	8mer	-0.17	94	0	0	N/A
hsa-miR-4768-3p	5967-5973	7mer-1A	-0.18	94	0	0	N/A
hsa-miR-4459	5967-5973	7mer-1A	-0.15	90	0	0	N/A
hsa-miR-3664-3p	5968-5974	7mer-m8	-0.18	93	0	0	N/A

hsa-miR-510-5p	5969-5976	8mer	-0.29	97	0	0	N/A
hsa-miR-512-5p	5970-5976	7mer-1A	-0.06	72	0	0	N/A
hsa-miR-548ai	5975-5981	7mer-1A	-0.1	73	0	0	N/A
hsa-miR-570-5p	5975-5981	7mer-1A	-0.1	73	0	0	N/A
hsa-miR-548ag	5975-5981	7mer-1A	-0.07	66	0	0	N/A
hsa-miR-548ba	5975-5981	7mer-1A	-0.07	64	0	0	N/A
hsa-miR-4795-3p	5981-5987	7mer-1A	-0.03	62	0	0	N/A
hsa-miR-126-5p	5982-5989	8mer	-0.03	68	0	0	N/A
hsa-miR-4795-3p	5983-5990	8mer	-0.03	63	0	0	N/A
hsa-miR-126-5p	5985-5992	8mer	-0.03	68	0	0	N/A
hsa-miR-4795-3p	5986-5993	8mer	-0.03	63	0	0	N/A
hsa-miR-126-5p	5988-5994	7mer-m8	-0.02	51	0	0.67	N/A
hsa-miR-323a-3p	5991-5997	7mer-1A	-0.01	52	0	0.67	N/A
hsa-miR-6507-5p	5996-6002	7mer-m8	-0.02	59	0	0	N/A
hsa-miR-186-5p	5997-6003	7mer-m8	-0.02	73	0	1.972	N/A
hsa-miR-495-3p	6001-6007	7mer-1A	-0.01	63	0	2.043	N/A
hsa-miR-5688	6001-6007	7mer-1A	-0.01	62	0	2.043	N/A
hsa-miR-7-2-3p	6001-6007	7mer-1A	-0.01	59	0	0.437	N/A
hsa-miR-7-1-3p	6001-6007	7mer-1A	-0.01	59	0	0.437	N/A
hsa-miR-2053	6005-6011	7mer-1A	-0.01	46	0	0	N/A

b. starBaseV3_hg19_CLIP-seq_miRNA-

#please cite:																					
#1.Zhou KR, Liu S, Cai L, et al. starBase: decoding the atlas of miRNA-target, RNA-RNA and protein-RNA interactions.																					
#2.Li JH, et al.starBase v2.0: decoding miRNA-ceRNA, miRNA-ncRNA and protein-RNA interaction networks from large-scale CLIP-Seq data , Nucleic Acids Res. 2014 Jan;42:D92-7.																					
miRNAid	miRNAname	geneID	geneName	geneType	chromosome	narrowStart	narrowEnd	broadStart	broadEnd	strand	clipExpNum	degraExpNum	RBP	PITA	RNA22	miRmap	microT	miRanda	PicTar	TargetScan	pancancerNum
MIMAT0000068	hsa-miR-15a-5p	ENSG00000146648	EGFR	protein_coding	chr7	55274319	55274324	55274319	55274324	+	4	0	AGO1-4,AGO2	1	0	1	0	0	0	0	11
MIMAT0000069	hsa-miR-16-5p	ENSG00000146648	EGFR	protein_coding	chr7	55274319	55274324	55274319	55274324	+	4	0	AGO1-4,AGO2	1	0	1	0	0	0	0	13
MIMAT0000070	hsa-miR-17-5p	ENSG00000146648	EGFR	protein_coding	chr7	55277885	55277891	55277868	55277892	+	2	0	AGO1-4,AGO2	0	1	0	0	0	0	1	13
MIMAT0000075	hsa-miR-20a-5p	ENSG00000146648	EGFR	protein_coding	chr7	55277885	55277891	55277868	55277892	+	2	0	AGO1-4,AGO2	0	1	0	0	0	0	1	10
MIMAT00000	hsa-miR-27a-	ENSG00000146	EGFR	protein_co	chr7	55210039	552100	55210039	552100	+	2	0	AGO2	0	0	0	1	0	0	0	3

84	3p	648		ding			67		67													
MIMAT0000084	hsa-miR-27a-3p	ENSG00000146648	EGFR	protein_coding	chr7	55273510	55273516	55273489	55273517	+	4	0	AGO1-4,AGO2	1	0	1	1	1	1	0	3
MIMAT0000084	hsa-miR-27a-3p	ENSG00000146648	EGFR	protein_coding	chr7	55275943	55275949	55275922	55275950	+	2	0	AGO1-4	0	0	1	1	0	0	0	3
MIMAT0000087	hsa-miR-30a-5p	ENSG00000146648	EGFR	protein_coding	chr7	55273438	55273443	55273438	55273443	+	5	0	AGO1-4,AGO2	1	0	0	0	0	0	0	6
MIMAT0000090	hsa-miR-32-5p	ENSG00000146648	EGFR	protein_coding	chr7	55279123	55279151	55279123	55279151	+	1	0	AGO1-4	0	0	0	1	0	0	0	15
MIMAT0000091	hsa-miR-33a-5p	ENSG00000146648	EGFR	protein_coding	chr7	55238699	55238704	55238685	55238705	+	1	0	AGO2	1	0	0	0	1	0	0	15
MIMAT0000091	hsa-miR-33a-5p	ENSG00000146648	EGFR	protein_coding	chr7	55274271	55274276	55274271	55274276	+	2	2	AGO1-4,AGO2	1	0	0	0	0	0	0	15
MIMAT0000092	hsa-miR-92a-3p	ENSG00000146648	EGFR	protein_coding	chr7	55279123	55279151	55279123	55279151	+	1	0	AGO1-4	0	0	0	1	0	0	0	12
MIMAT0000093	hsa-miR-93-5p	ENSG00000146648	EGFR	protein_coding	chr7	55277885	55277891	55277868	55277892	+	2	0	AGO1-4,AGO2	0	1	0	0	0	0	1	10
MIMAT	hsa-mi	ENSG00	EGF	prote	chr7	5527	552	5527	552	+	1	0	AGO2	1	0	0	0	0	0	0	5

0000099	R-101-3p	000146648	R	in_coding		3869	73875	3869	73875												
MIMAT0000101	hsa-miR-103a-3p	ENSG00000146648	EGFR	protein_coding	chr7	55274080	55274086	55274060	55274087	+	5	0	AGO1-4,AGO2	1	0	0	0	1	0	0	14
MIMAT0000102	hsa-miR-105-5p	ENSG00000146648	EGFR	protein_coding	chr7	55274169	55274176	55274149	55274177	+	5	0	AGO1-4,AGO2	1	0	0	1	0	0	0	7
MIMAT0000103	hsa-miR-106a-5p	ENSG00000146648	EGFR	protein_coding	chr7	55277885	55277891	55277885	55277891	+	2	0	AGO1-4,AGO2	0	0	0	0	0	0	1	13
MIMAT0000104	hsa-miR-107	ENSG00000146648	EGFR	protein_coding	chr7	55274080	55274086	55274060	55274087	+	5	0	AGO1-4,AGO2	1	0	0	0	1	0	0	12
MIMAT0000222	hsa-miR-192-5p	ENSG00000146648	EGFR	protein_coding	chr7	55274447	55274452	55274447	55274452	+	2	0	AGO2	1	0	0	0	0	0	0	11
MIMAT0000242	hsa-miR-129-5p	ENSG00000146648	EGFR	protein_coding	chr7	55224282	55224302	55224282	55224302	+	2	0	AGO1-4	0	0	0	0	1	0	0	5
MIMAT0000242	hsa-miR-129-5p	ENSG00000146648	EGFR	protein_coding	chr7	55274235	55274242	55274215	55274243	+	5	0	AGO1-4,AGO2	1	0	0	1	1	0	0	5
MIMAT0000242	hsa-miR-129-5p	ENSG00000146648	EGFR	protein_coding	chr7	55274309	55274314	55274287	55274315	+	4	1	AGO1-4,AGO2	1	0	0	1	0	0	0	5

MIMAT0000242	hsa-miR-129-5p	ENSG00000146648	EGFR	protein_coding	chr7	55274488	55274493	55274466	55274494	+	2	0	AGO2	1	0	0	1	0	0	0	5
MIMAT0000243	hsa-miR-148a-3p	ENSG00000146648	EGFR	protein_coding	chr7	55276357	55276362	55276357	55276362	+	1	0	AGO1-4	0	0	1	0	0	0	0	9
MIMAT0000244	hsa-miR-30c-5p	ENSG00000146648	EGFR	protein_coding	chr7	55273438	55273443	55273438	55273443	+	5	0	AGO1-4,AGO2	1	0	0	0	0	0	0	9
MIMAT0000245	hsa-miR-30d-5p	ENSG00000146648	EGFR	protein_coding	chr7	55273438	55273443	55273438	55273443	+	5	0	AGO1-4,AGO2	1	0	0	0	0	0	0	8
MIMAT0000251	hsa-miR-147a	ENSG00000146648	EGFR	protein_coding	chr7	55274484	55274489	55274484	55274489	+	1	0	AGO2	1	0	1	0	0	0	0	0
MIMAT0000251	hsa-miR-147a	ENSG00000146648	EGFR	protein_coding	chr7	55274898	55274899	55274894	55274903	+	1	0	AGO1-4	3	0	3	0	0	0	0	0
MIMAT0000251	hsa-miR-147a	ENSG00000146648	EGFR	protein_coding	chr7	55277269	55277274	55277269	55277274	+	2	0	AGO1-4,AGO2	0	0	1	0	0	0	0	0
MIMAT0000252	hsa-miR-7-5p	ENSG00000146648	EGFR	protein_coding	chr7	55238402	55238407	55238402	55238407	+	2	0	AGO2	1	0	0	0	0	0	0	11
MIMAT00002	hsa-miR-7-5p	ENSG00000146	EGFR	protein_co	chr7	55273767	552737	55273746	552737	+	1	0	AGO2	1	0	1	1	1	0	1	11

52		648		ding			73		74												
MIMAT0000252	hsa-miR-7-5p	ENSG00000146648	EGFR	protein_coding	chr7	55273952	55273957	55273930	55273958	+	4	0	AGO1-4,AGO2	1	0	1	1	0	0	0	11
MIMAT0000252	hsa-miR-7-5p	ENSG00000146648	EGFR	protein_coding	chr7	55274050	55274056	55274029	55274057	+	5	0	AGO1-4,AGO2	1	0	1	1	1	0	0	11
MIMAT0000252	hsa-miR-7-5p	ENSG00000146648	EGFR	protein_coding	chr7	55276045	55276051	55276024	55276052	+	2	0	AGO1-4	0	0	1	1	0	0	0	11
MIMAT0000252	hsa-miR-7-5p	ENSG00000146648	EGFR	protein_coding	chr7	55276104	55276109	55276082	55276110	+	1	0	AGO1-4	0	0	1	1	0	0	0	11
MIMAT0000256	hsa-miR-181a-5p	ENSG00000146648	EGFR	protein_coding	chr7	55274197	55274202	55274197	55274202	+	4	0	AGO1-4,AGO2	1	0	0	0	0	0	0	4
MIMAT0000257	hsa-miR-181b-5p	ENSG00000146648	EGFR	protein_coding	chr7	55274197	55274202	55274197	55274202	+	4	0	AGO1-4,AGO2	1	0	0	0	0	0	0	3
MIMAT0000258	hsa-miR-181c-5p	ENSG00000146648	EGFR	protein_coding	chr7	55274197	55274202	55274197	55274202	+	4	0	AGO1-4,AGO2	1	0	0	0	0	0	0	5
MIMAT0000261	hsa-miR-183-5p	ENSG00000146648	EGFR	protein_coding	chr7	55233093	55233114	55233093	55233114	+	1	0	AGO2	0	0	0	0	1	0	0	9
MIMAT	hsa-mi	ENSG00	EGF	prote	chr7	5527	552	5527	552	+	2	0	AGO2	1	0	0	0	0	0	0	7

0000272	R-215-5p	000146648	R	in_coding		4447	74452	4447	74452												
MIMAT0000275	hsa-miR-218-5p	ENSG00000146648	EGFR	protein_coding	chr7	55238419	55238439	55238419	55238439	+	1	0	AGO2	0	0	0	0	1	0	0	6
MIMAT0000275	hsa-miR-218-5p	ENSG00000146648	EGFR	protein_coding	chr7	55273753	55273758	55273753	55273758	+	1	0	AGO2	1	0	1	0	0	0	0	6
MIMAT0000275	hsa-miR-218-5p	ENSG00000146648	EGFR	protein_coding	chr7	55277266	55277272	55277266	55277272	+	2	0	AGO1-4,AGO2	0	0	1	0	0	0	0	6
MIMAT0000278	hsa-miR-221-3p	ENSG00000146648	EGFR	protein_coding	chr7	55275710	55275715	55275710	55275715	+	2	0	AGO2	0	0	1	0	0	0	0	6
MIMAT0000279	hsa-miR-222-3p	ENSG00000146648	EGFR	protein_coding	chr7	55275710	55275715	55275710	55275715	+	2	0	AGO2	0	0	1	0	0	0	0	6
MIMAT0000280	hsa-miR-223-3p	ENSG00000146648	EGFR	protein_coding	chr7	55238395	55238400	55238395	55238400	+	2	0	AGO2	1	0	0	0	0	0	0	5
MIMAT0000280	hsa-miR-223-3p	ENSG00000146648	EGFR	protein_coding	chr7	55274036	55274043	55274036	55274043	+	5	0	AGO1-4,AGO2	1	0	0	0	0	0	0	5
MIMAT0000281	hsa-miR-224-5p	ENSG00000146648	EGFR	protein_coding	chr7	55274040	55274045	55274040	55274045	+	5	0	AGO1-4,AGO2	1	0	1	0	0	0	0	3

MIMAT0000281	hsa-miR-224-5p	ENSG00000146648	EGFR	protein_coding	chr7	55277911	55277917	55277911	55277917	+	3	0	AGO1-4,AGO2	0	0	1	0	0	0	0	3
MIMAT0000318	hsa-miR-200b-3p	ENSG00000146648	EGFR	protein_coding	chr7	55274980	55274985	55274958	55274986	+	1	0	AGO1-4	1	0	1	1	0	0	0	5
MIMAT0000318	hsa-miR-200b-3p	ENSG00000146648	EGFR	protein_coding	chr7	55275726	55275731	55275704	55275732	+	2	0	AGO2	0	0	1	1	0	0	0	5
MIMAT0000318	hsa-miR-200b-3p	ENSG00000146648	EGFR	protein_coding	chr7	55279159	55279165	55279138	55279166	+	1	0	AGO1-4	0	0	1	1	0	0	0	5
MIMAT0000318	hsa-miR-200b-3p	ENSG00000146648	EGFR	protein_coding	chr7	55279237	55279242	55279215	55279243	+	1	0	AGO1-4	0	0	1	1	0	0	0	5
MIMAT0000416	hsa-miR-1-3p	ENSG00000146648	EGFR	protein_coding	chr7	55273493	55273498	55273491	55273498	+	4	0	AGO1-4,AGO2	1	0	1	0	0	0	0	3
MIMAT0000416	hsa-miR-1-3p	ENSG00000146648	EGFR	protein_coding	chr7	55274056	55274061	55274034	55274062	+	5	0	AGO1-4,AGO2	1	0	1	1	0	0	0	3
MIMAT0000417	hsa-miR-15b-5p	ENSG00000146648	EGFR	protein_coding	chr7	55274319	55274324	55274319	55274324	+	4	0	AGO1-4,AGO2	1	0	1	0	0	0	0	4
MIMAT00004	hsa-miR-27b-	ENSG00000146	EGFR	protein_co	chr7	55210039	552100	55210039	552100	+	2	0	AGO2	0	0	0	1	0	0	0	4

19	3p	648		ding			67		67												
MIMAT0000419	hsa-miR-27b-3p	ENSG00000146648	EGFR	protein_coding	chr7	55273510	55273516	55273489	55273517	+	4	0	AGO1-4,AGO2	1	0	1	1	1	1	0	4
MIMAT0000419	hsa-miR-27b-3p	ENSG00000146648	EGFR	protein_coding	chr7	55275943	55275949	55275922	55275950	+	2	0	AGO1-4	0	0	1	1	0	0	0	4
MIMAT0000420	hsa-miR-30b-5p	ENSG00000146648	EGFR	protein_coding	chr7	55273438	55273443	55273438	55273443	+	5	0	AGO1-4,AGO2	1	0	0	0	0	0	0	10
MIMAT0000422	hsa-miR-124-3p	ENSG00000146648	EGFR	protein_coding	chr7	55274501	55274506	55274501	55274506	+	1	0	AGO2	1	0	0	0	0	0	0	4
MIMAT0000424	hsa-miR-128-3p	ENSG00000146648	EGFR	protein_coding	chr7	55210038	55210066	55210038	55210066	+	2	0	AGO2	0	0	0	1	0	0	0	11
MIMAT0000424	hsa-miR-128-3p	ENSG00000146648	EGFR	protein_coding	chr7	55273510	55273515	55273488	55273516	+	4	0	AGO1-4,AGO2	1	0	0	1	0	1	0	11
MIMAT0000424	hsa-miR-128-3p	ENSG00000146648	EGFR	protein_coding	chr7	55275921	55275949	55275921	55275949	+	2	0	AGO1-4	0	0	0	1	0	0	0	11
MIMAT0000424	hsa-miR-128-3p	ENSG00000146648	EGFR	protein_coding	chr7	55276337	55276365	55276337	55276365	+	1	0	AGO1-4	0	0	0	1	0	0	0	11
MIMAT	hsa-mi	ENSG00	EGF	prote	chr7	5527	552	5527	552	+	5	0	AGO1	1	0	1	1	0	0	1	1

0000427	R-133a-3p	000146648	R	in_coding		3360	73366	3339	73367				-4, AGO2								
MIMAT0000427	hsa-miR-133a-3p	ENSG00000146648	EGFR	protein_coding	chr7	55274176	55274182	55274155	55274183	+	5	0	AGO1-4, AGO2	1	0	1	1	1	0	0	1
MIMAT0000428	hsa-miR-135a-5p	ENSG00000146648	EGFR	protein_coding	chr7	55273392	55273397	55273392	55273397	+	4	0	AGO1-4, AGO2	1	0	0	0	0	0	0	10
MIMAT0000428	hsa-miR-135a-5p	ENSG00000146648	EGFR	protein_coding	chr7	55274339	55274344	55274339	55274344	+	5	0	AGO1-4, AGO2	1	0	0	0	0	0	0	10
MIMAT0000429	hsa-miR-137	ENSG00000146648	EGFR	protein_coding	chr7	55277873	55277879	55277852	55277880	+	2	0	AGO1-4, AGO2	0	0	1	1	0	0	1	2
MIMAT0000429	hsa-miR-137	ENSG00000146648	EGFR	protein_coding	chr7	55279066	55279072	55279045	55279073	+	1	0	AGO1-4	0	0	1	1	0	0	1	2
MIMAT0000429	hsa-miR-137	ENSG00000146648	EGFR	protein_coding	chr7	55279094	55279100	55279073	55279101	+	1	0	AGO1-4	0	0	1	1	0	0	1	2
MIMAT0000430	hsa-miR-138-5p	ENSG00000146648	EGFR	protein_coding	chr7	55277358	55277364	55277358	55277364	+	1	0	AGO1-4	0	0	1	0	0	0	0	5
MIMAT0000432	hsa-miR-141-3p	ENSG00000146648	EGFR	protein_coding	chr7	55224564	55224570	55224547	55224571	+	1	0	AGO2	1	0	1	0	1	0	0	13

MIMAT0000432	hsa-miR-141-3p	ENSG00000146648	EGFR	protein_coding	chr7	55274798	55274804	55274777	55274805	+	3	0	AGO1-4,AGO2	1	0	1	1	1	0	0	13
MIMAT0000432	hsa-miR-141-3p	ENSG00000146648	EGFR	protein_coding	chr7	55277773	55277779	55277752	55277780	+	1	0	AGO1-4	0	0	1	1	0	0	0	13
MIMAT0000433	hsa-miR-142-5p	ENSG00000146648	EGFR	protein_coding	chr7	55279102	55279107	55279102	55279107	+	1	0	AGO1-4	0	0	1	0	0	0	0	10
MIMAT0000435	hsa-miR-143-3p	ENSG00000146648	EGFR	protein_coding	chr7	55275495	55275501	55275495	55275501	+	1	0	AGO1-4	0	0	1	0	0	0	0	2
MIMAT0000436	hsa-miR-144-3p	ENSG00000146648	EGFR	protein_coding	chr7	55273870	55273875	55273848	55273876	+	2	0	AGO2	1	0	0	1	0	0	0	2
MIMAT0000436	hsa-miR-144-3p	ENSG00000146648	EGFR	protein_coding	chr7	55279204	55279210	55279183	55279211	+	1	0	AGO1-4	0	0	0	1	0	0	1	2
MIMAT0000437	hsa-miR-145-5p	ENSG00000146648	EGFR	protein_coding	chr7	55224477	55224497	55224477	55224497	+	1	1	AGO1-4	0	0	0	0	1	0	0	3
MIMAT0000438	hsa-miR-152-3p	ENSG00000146648	EGFR	protein_coding	chr7	55276357	55276362	55276357	55276362	+	1	0	AGO1-4	0	0	1	0	0	0	0	4
MIMAT00004	hsa-miR-9-3p	ENSG00000146	EGFR	protein_co	chr7	55279079	552791	55279079	552791	+	1	0	AGO1-4	0	0	0	1	0	0	0	6

42		648		ding			07		07												
MIMAT00004 44	hsa-miR-126-5p	ENSG00000146 648	EGFR	protein_coding	chr7	55274110	55274138	55274110	55274138	+	3	0	AGO1-4,AGO2	0	0	0	1	0	0	0	8
MIMAT00004 45	hsa-miR-126-3p	ENSG00000146 648	EGFR	protein_coding	chr7	55279121	55279126	55279121	55279126	+	1	0	AGO1-4	0	0	1	0	0	0	0	10
MIMAT00004 47	hsa-miR-134-5p	ENSG00000146 648	EGFR	protein_coding	chr7	55274890	55274896	55274874	55274897	+	1	0	AGO1-4	1	0	1	0	1	0	0	4
MIMAT00004 47	hsa-miR-134-5p	ENSG00000146 648	EGFR	protein_coding	chr7	55275395	55275401	55275395	55275401	+	1	0	AGO2	0	0	1	0	0	0	0	4
MIMAT00004 47	hsa-miR-134-5p	ENSG00000146 648	EGFR	protein_coding	chr7	55275702	55275707	55275702	55275707	+	2	0	AGO2	0	0	1	0	0	0	0	4
MIMAT00004 49	hsa-miR-146a-5p	ENSG00000146 648	EGFR	protein_coding	chr7	55274230	55274235	55274208	55274236	+	4	0	AGO1-4,AGO2	1	0	1	1	0	0	0	10
MIMAT00004 49	hsa-miR-146a-5p	ENSG00000146 648	EGFR	protein_coding	chr7	55275861	55275867	55275840	55275868	+	1	0	AGO2	0	0	1	1	0	0	0	10
MIMAT00004 50	hsa-miR-149-5p	ENSG00000146 648	EGFR	protein_coding	chr7	55238552	55238558	55238537	55238559	+	2	0	AGO2	1	0	1	0	1	0	0	7
MIMAT	hsa-mi	ENSG00	EGF	prote	chr7	5527	552	5527	552	+	5	0	AGO1	1	0	0	0	0	0	0	7

0000450	R-149-5p	000146648	R	in_coding		3451	73456	3451	73456				-4,AGO2								
MIMAT0000452	hsa-miR-154-5p	ENSG00000146648	EGFR	protein_coding	chr7	55274765	55274770	55274765	55274770	+	1	0	AGO2	1	0	0	0	0	0	0	3
MIMAT0000455	hsa-miR-185-5p	ENSG00000146648	EGFR	protein_coding	chr7	55224249	55224270	55224249	55224270	+	2	0	AGO1-4	0	0	0	0	1	0	0	12
MIMAT0000455	hsa-miR-185-5p	ENSG00000146648	EGFR	protein_coding	chr7	55274810	55274815	55274810	55274815	+	2	0	AGO1-4,AGO2	1	0	0	0	0	0	0	12
MIMAT0000456	hsa-miR-186-5p	ENSG00000146648	EGFR	protein_coding	chr7	55279248	55279276	55279248	55279276	+	1	0	AGO1-4	0	0	0	1	0	0	0	17
MIMAT0000459	hsa-miR-193a-3p	ENSG00000146648	EGFR	protein_coding	chr7	55238350	55238355	55238350	55238355	+	2	0	AGO2	1	0	0	0	0	0	0	3
MIMAT0000460	hsa-miR-194-5p	ENSG00000146648	EGFR	protein_coding	chr7	55274371	55274376	55274371	55274376	+	4	0	AGO1-4,AGO2	1	0	1	0	0	0	0	9
MIMAT0000460	hsa-miR-194-5p	ENSG00000146648	EGFR	protein_coding	chr7	55277799	55277804	55277799	55277804	+	1	0	AGO1-4	0	0	1	0	0	0	0	9
MIMAT0000461	hsa-miR-195-5p	ENSG00000146648	EGFR	protein_coding	chr7	55274319	55274324	55274319	55274324	+	4	0	AGO1-4,AGO2	1	0	1	0	0	0	0	1

MIMAT0000462	hsa-miR-206	ENSG00000146648	EGFR	protein_coding	chr7	55273493	55273498	55273491	55273498	+	4	0	AGO1-4,AGO2	1	0	1	0	0	0	0	2
MIMAT0000462	hsa-miR-206	ENSG00000146648	EGFR	protein_coding	chr7	55274056	55274061	55274034	55274062	+	5	0	AGO1-4,AGO2	1	0	1	1	0	0	0	2
MIMAT0000510	hsa-miR-320a	ENSG00000146648	EGFR	protein_coding	chr7	55221790	55221818	55221790	55221818	+	2	0	AGO1-4,AGO2	0	0	0	1	0	0	0	7
MIMAT0000510	hsa-miR-320a	ENSG00000146648	EGFR	protein_coding	chr7	55274415	55274420	55274393	55274421	+	6	0	AGO1-4,AGO2	1	0	0	1	0	0	0	7
MIMAT0000510	hsa-miR-320a	ENSG00000146648	EGFR	protein_coding	chr7	55277366	55277394	55277366	55277394	+	1	0	AGO1-4	0	0	0	1	0	0	0	7
MIMAT0000510	hsa-miR-320a	ENSG00000146648	EGFR	protein_coding	chr7	55279078	55279106	55279078	55279106	+	1	0	AGO1-4	0	0	0	1	0	0	0	7
MIMAT0000617	hsa-miR-200c-3p	ENSG00000146648	EGFR	protein_coding	chr7	55274980	55274985	55274958	55274986	+	1	0	AGO1-4	1	0	1	1	0	0	0	8
MIMAT0000617	hsa-miR-200c-3p	ENSG00000146648	EGFR	protein_coding	chr7	55275726	55275731	55275704	55275732	+	2	0	AGO2	0	0	1	1	0	0	0	8
MIMAT00006	hsa-miR-200c	ENSG00000146	EGFR	protein_co	chr7	55279159	552791	55279138	552791	+	1	0	AGO1-4	0	0	1	1	0	0	0	8

17	-3p	648		ding			65		66											
MIMAT0000617	hsa-miR-200c-3p	ENSG00000146648	EGFR	protein_coding	chr7	55279237	55279242	55279215	55279243	+	1	0	AGO1-4	0	0	1	1	0	0	0	8
MIMAT0000646	hsa-miR-155-5p	ENSG00000146648	EGFR	protein_coding	chr7	55273402	55273407	55273402	55273407	+	6	0	AGO1-4,AGO2	1	0	1	0	0	0	0	6
MIMAT0000646	hsa-miR-155-5p	ENSG00000146648	EGFR	protein_coding	chr7	55274573	55274579	55274559	55274580	+	1	0	AGO2	1	0	1	0	1	0	0	6
MIMAT0000646	hsa-miR-155-5p	ENSG00000146648	EGFR	protein_coding	chr7	55275713	55275719	55275713	55275719	+	2	0	AGO2	0	0	1	0	0	0	0	6
MIMAT0000680	hsa-miR-106b-5p	ENSG00000146648	EGFR	protein_coding	chr7	55277885	55277891	55277870	55277892	+	2	0	AGO1-4,AGO2	0	1	0	0	0	0	1	11
MIMAT0000682	hsa-miR-200a-3p	ENSG00000146648	EGFR	protein_coding	chr7	55224564	55224570	55224550	55224571	+	1	0	AGO2	1	0	1	0	1	0	0	11
MIMAT0000682	hsa-miR-200a-3p	ENSG00000146648	EGFR	protein_coding	chr7	55274798	55274804	55274777	55274805	+	3	0	AGO1-4,AGO2	1	0	1	1	1	0	0	11
MIMAT0000682	hsa-miR-200a-3p	ENSG00000146648	EGFR	protein_coding	chr7	55277773	55277779	55277752	55277780	+	1	0	AGO1-4	0	0	1	1	0	0	0	11
MIMAT	hsa-mi	ENSG00	EGF	prote	chr7	5527	552	5527	552	+	1	2	AGO2	1	0	1	1	1	0	0	6

0000684	R-302a-3p	000146648	R	in_coding		4521	74527	4500	74528												
MIMAT0000684	hsa-miR-302a-3p	ENSG00000146648	EGFR	protein_coding	chr7	55277884	55277890	55277863	55277891	+	2	0	AGO1-4,AGO2	0	0	1	1	0	0	1	6
MIMAT0000687	hsa-miR-299-3p	ENSG00000146648	EGFR	protein_coding	chr7	55274152	55274158	55274139	55274159	+	2	0	AGO1-4	1	0	0	0	1	0	0	2
MIMAT0000692	hsa-miR-30e-5p	ENSG00000146648	EGFR	protein_coding	chr7	55273438	55273443	55273438	55273443	+	5	0	AGO1-4,AGO2	1	0	0	0	0	0	0	12
MIMAT0000705	hsa-miR-362-5p	ENSG00000146648	EGFR	protein_coding	chr7	55274210	55274217	55274210	55274217	+	4	0	AGO1-4,AGO2	1	0	0	0	0	0	0	16
MIMAT0000715	hsa-miR-302b-3p	ENSG00000146648	EGFR	protein_coding	chr7	55274521	55274527	55274500	55274528	+	1	2	AGO2	1	0	1	1	1	0	0	4
MIMAT0000715	hsa-miR-302b-3p	ENSG00000146648	EGFR	protein_coding	chr7	55277884	55277890	55277863	55277891	+	2	0	AGO1-4,AGO2	0	0	1	1	0	0	1	4
MIMAT0000717	hsa-miR-302c-3p	ENSG00000146648	EGFR	protein_coding	chr7	55274521	55274527	55274500	55274528	+	1	2	AGO2	1	0	1	1	1	0	0	2
MIMAT0000717	hsa-miR-302c-3p	ENSG00000146648	EGFR	protein_coding	chr7	55277884	55277890	55277863	55277891	+	2	0	AGO1-4,AGO2	0	0	1	1	0	0	1	2

MIMAT0000718	hsa-miR-302d-3p	ENSG00000146648	EGFR	protein_coding	chr7	55274521	55274527	55274500	55274528	+	1	2	AGO2	1	0	1	1	1	0	0	4
MIMAT0000718	hsa-miR-302d-3p	ENSG00000146648	EGFR	protein_coding	chr7	55277884	55277890	55277863	55277891	+	2	0	AGO1-4,AGO2	0	0	1	1	0	0	1	4
MIMAT0000722	hsa-miR-370-3p	ENSG00000146648	EGFR	protein_coding	chr7	55273371	55273377	55273356	55273378	+	5	0	AGO1-4,AGO2	1	0	1	0	1	0	0	2
MIMAT0000722	hsa-miR-370-3p	ENSG00000146648	EGFR	protein_coding	chr7	55273854	55273860	55273853	55273860	+	2	0	AGO2	1	0	1	0	0	0	0	2
MIMAT0000724	hsa-miR-372-3p	ENSG00000146648	EGFR	protein_coding	chr7	55274521	55274527	55274506	55274528	+	1	2	AGO2	1	0	1	0	1	0	0	4
MIMAT0000724	hsa-miR-372-3p	ENSG00000146648	EGFR	protein_coding	chr7	55277884	55277890	55277884	55277890	+	2	0	AGO1-4,AGO2	0	0	1	0	0	0	1	4
MIMAT0000726	hsa-miR-373-3p	ENSG00000146648	EGFR	protein_coding	chr7	55274521	55274527	55274500	55274528	+	1	2	AGO2	1	0	1	1	1	0	0	3
MIMAT0000726	hsa-miR-373-3p	ENSG00000146648	EGFR	protein_coding	chr7	55277884	55277890	55277863	55277891	+	2	0	AGO1-4,AGO2	0	0	1	1	0	0	1	3
MIMAT00007	hsa-miR-330-	ENSG00000146	EGFR	protein_co	chr7	55274477	552744	55274477	552744	+	1	0	AGO2	1	0	0	0	0	0	0	5

51	3p	648		ding			82		82												
MIMAT0000753	hsa-miR-342-3p	ENSG00000146648	EGFR	protein_coding	chr7	55276333	55276338	55276333	55276338	+	1	0	AGO1-4	0	0	1	0	0	0	0	10
MIMAT0000755	hsa-miR-323a-3p	ENSG00000146648	EGFR	protein_coding	chr7	55277907	55277912	55277907	55277912	+	3	0	AGO1-4,AGO2	0	0	1	0	0	0	0	4
MIMAT0000756	hsa-miR-326	ENSG00000146648	EGFR	protein_coding	chr7	55273772	55273777	55273772	55273777	+	1	0	AGO2	1	0	0	0	0	0	0	3
MIMAT0000758	hsa-miR-135b-5p	ENSG00000146648	EGFR	protein_coding	chr7	55273392	55273397	55273392	55273397	+	4	0	AGO1-4,AGO2	1	0	0	0	0	0	0	8
MIMAT0000758	hsa-miR-135b-5p	ENSG00000146648	EGFR	protein_coding	chr7	55274339	55274344	55274339	55274344	+	5	0	AGO1-4,AGO2	1	0	0	0	0	0	0	8
MIMAT0000759	hsa-miR-148b-3p	ENSG00000146648	EGFR	protein_coding	chr7	55276357	55276362	55276357	55276362	+	1	0	AGO1-4	0	0	1	0	0	0	0	16
MIMAT0000760	hsa-miR-331-3p	ENSG00000146648	EGFR	protein_coding	chr7	55275326	55275331	55275326	55275331	+	1	0	AGO2	0	0	1	0	0	0	0	7
MIMAT0000760	hsa-miR-331-3p	ENSG00000146648	EGFR	protein_coding	chr7	55277378	55277384	55277378	55277384	+	1	0	AGO1-4	0	0	1	0	0	0	0	7
MIMAT	hsa-mi	ENSG00	EGF	prote	chr7	5527	552	5527	552	+	1	0	AGO2	1	0	1	0	0	0	0	15

0000761	R-324-5p	000146648	R	in_coding		3777	73782	3777	73782												
MIMAT0000761	hsa-miR-324-5p	ENSG00000146648	EGFR	protein_coding	chr7	55276364	55276369	55276364	55276369	+	1	0	AGO1-4	0	0	1	0	0	0	0	15
MIMAT0000762	hsa-miR-324-3p	ENSG00000146648	EGFR	protein_coding	chr7	55275951	55275956	55275951	55275956	+	2	0	AGO1-4	0	0	1	0	0	0	0	12
MIMAT0000762	hsa-miR-324-3p	ENSG00000146648	EGFR	protein_coding	chr7	55277769	55277775	55277769	55277775	+	1	0	AGO1-4	0	0	1	0	0	0	0	12
MIMAT0000763	hsa-miR-338-3p	ENSG00000146648	EGFR	protein_coding	chr7	55241688	55241708	55241688	55241708	+	2	0	AGO1-4,AGO2	0	1	0	0	0	0	0	12
MIMAT0000770	hsa-miR-133b	ENSG00000146648	EGFR	protein_coding	chr7	55273360	55273366	55273339	55273367	+	5	0	AGO1-4,AGO2	1	0	1	1	0	0	1	3
MIMAT0000770	hsa-miR-133b	ENSG00000146648	EGFR	protein_coding	chr7	55274176	55274182	55274155	55274183	+	5	0	AGO1-4,AGO2	1	0	1	1	1	0	0	3
MIMAT0000772	hsa-miR-345-5p	ENSG00000146648	EGFR	protein_coding	chr7	55274951	55274956	55274951	55274956	+	1	0	AGO1-4	1	0	1	0	0	0	0	11
MIMAT0000772	hsa-miR-345-5p	ENSG00000146648	EGFR	protein_coding	chr7	55275612	55275617	55275612	55275617	+	2	0	AGO1-4	0	0	1	0	0	0	0	11

MIMAT0000772	hsa-miR-345-5p	ENSG00000146648	EGFR	protein_coding	chr7	55276010	55276015	55276010	55276015	+	2	0	AGO1-4	0	0	1	0	0	0	0	11
MIMAT0000772	hsa-miR-345-5p	ENSG00000146648	EGFR	protein_coding	chr7	55276112	55276117	55276112	55276117	+	1	0	AGO1-4	0	0	1	0	0	0	0	11
MIMAT0001341	hsa-miR-424-5p	ENSG00000146648	EGFR	protein_coding	chr7	55240702	55240730	55240702	55240730	+	2	0	AGO1-4,AGO2	0	0	0	1	0	0	0	6
MIMAT0001341	hsa-miR-424-5p	ENSG00000146648	EGFR	protein_coding	chr7	55274319	55274324	55274297	55274325	+	4	2	AGO1-4,AGO2	1	0	1	1	0	0	0	6
MIMAT0001413	hsa-miR-20b-5p	ENSG00000146648	EGFR	protein_coding	chr7	55277885	55277891	55277868	55277892	+	2	0	AGO1-4,AGO2	0	1	0	0	0	0	1	9
MIMAT0001536	hsa-miR-429	ENSG00000146648	EGFR	protein_coding	chr7	55274980	55274985	55274958	55274986	+	1	0	AGO1-4	1	0	1	1	0	0	0	7
MIMAT0001536	hsa-miR-429	ENSG00000146648	EGFR	protein_coding	chr7	55275726	55275731	55275704	55275732	+	2	0	AGO2	0	0	1	1	0	0	0	7
MIMAT0001536	hsa-miR-429	ENSG00000146648	EGFR	protein_coding	chr7	55279159	55279165	55279138	55279166	+	1	0	AGO1-4	0	0	1	1	0	0	0	7
MIMAT00015	hsa-miR-429	ENSG00000146	EGFR	protein_co	chr7	55279237	552792	55279215	552792	+	1	0	AGO1-4	0	0	1	1	0	0	0	7

36		648		ding			42		43												
MIMAT0001545	hsa-miR-450a-5p	ENSG00000146648	EGFR	protein_coding	chr7	55274234	55274240	55274220	55274241	+	5	0	AGO1-4,AGO2	1	0	0	0	1	0	0	7
MIMAT0001627	hsa-miR-433-3p	ENSG00000146648	EGFR	protein_coding	chr7	55274025	55274030	55274025	55274030	+	5	0	AGO1-4,AGO2	1	0	1	0	0	0	0	3
MIMAT0001627	hsa-miR-433-3p	ENSG00000146648	EGFR	protein_coding	chr7	55274100	55274105	55274100	55274105	+	4	0	AGO1-4,AGO2	1	0	1	0	0	0	0	3
MIMAT0001629	hsa-miR-329-3p	ENSG00000146648	EGFR	protein_coding	chr7	55274753	55274758	55274753	55274758	+	1	0	AGO2	1	0	0	0	0	0	0	1
MIMAT0001635	hsa-miR-452-5p	ENSG00000146648	EGFR	protein_coding	chr7	55274795	55274801	55274795	55274801	+	3	0	AGO1-4,AGO2	1	0	1	0	0	0	0	2
MIMAT0001638	hsa-miR-409-5p	ENSG00000146648	EGFR	protein_coding	chr7	55274764	55274769	55274764	55274769	+	1	0	AGO2	1	0	1	0	0	0	0	6
MIMAT0001638	hsa-miR-409-5p	ENSG00000146648	EGFR	protein_coding	chr7	55277851	55277856	55277851	55277856	+	2	0	AGO1-4,AGO2	0	0	1	0	0	0	0	6
MIMAT0001639	hsa-miR-409-3p	ENSG00000146648	EGFR	protein_coding	chr7	55224617	55224622	55224617	55224622	+	1	0	AGO2	1	0	0	0	0	0	0	4
MIMAT	hsa-mi	ENSG00	EGF	prote	chr7	5527	552	5527	552	+	1	0	AGO1	0	0	1	1	0	0	0	4

0001639	R-409-3p	000146648	R	in_coding		5528	75534	5507	75535				-4								
MIMAT0001639	hsa-miR-409-3p	ENSG00000146648	EGFR	protein_coding	chr7	55279086	55279091	55279064	55279092	+	1	0	AGO1-4	0	0	1	1	0	0	0	4
MIMAT0002171	hsa-miR-410-3p	ENSG00000146648	EGFR	protein_coding	chr7	55275552	55275557	55275530	55275558	+	1	0	AGO1-4	0	0	1	1	0	0	0	2
MIMAT0002171	hsa-miR-410-3p	ENSG00000146648	EGFR	protein_coding	chr7	55277898	55277903	55277876	55277904	+	3	1	AGO1-4,AGO2	0	0	1	1	0	0	0	2
MIMAT0002171	hsa-miR-410-3p	ENSG00000146648	EGFR	protein_coding	chr7	55277935	55277940	55277913	55277941	+	3	0	AGO1-4,AGO2	0	0	1	1	0	0	0	2
MIMAT0002171	hsa-miR-410-3p	ENSG00000146648	EGFR	protein_coding	chr7	55279193	55279198	55279171	55279199	+	1	0	AGO1-4	0	0	1	1	0	0	0	2
MIMAT0002807	hsa-miR-491-5p	ENSG00000146648	EGFR	protein_coding	chr7	55273983	55273987	55273966	55274004	+	5	0	AGO1-4,AGO2	1	0	1	0	2	0	0	5
MIMAT0002807	hsa-miR-491-5p	ENSG00000146648	EGFR	protein_coding	chr7	55273997	55274003	55273997	55274003	+	5	0	AGO1-4,AGO2	1	0	1	0	0	0	0	5
MIMAT0002809	hsa-miR-146b-5p	ENSG00000146648	EGFR	protein_coding	chr7	55274230	55274235	55274208	55274236	+	4	0	AGO1-4,AGO2	1	0	1	1	0	0	0	7

MIMAT0002809	hsa-miR-146b-5p	ENSG00000146648	EGFR	protein_coding	chr7	55275861	55275867	55275840	55275868	+	1	0	AGO2	0	0	1	1	0	0	0	7
MIMAT0002813	hsa-miR-493-5p	ENSG00000146648	EGFR	protein_coding	chr7	55277858	55277863	55277836	55277864	+	2	0	AGO1-4,AGO2	0	0	1	1	0	0	0	1
MIMAT0002813	hsa-miR-493-5p	ENSG00000146648	EGFR	protein_coding	chr7	55279166	55279171	55279144	55279172	+	1	0	AGO1-4	0	0	1	1	0	0	1	1
MIMAT0002814	hsa-miR-432-5p	ENSG00000146648	EGFR	protein_coding	chr7	55273832	55273837	55273832	55273837	+	2	0	AGO2	1	0	0	0	0	0	0	1
MIMAT0002817	hsa-miR-495-3p	ENSG00000146648	EGFR	protein_coding	chr7	55274227	55274232	55274205	55274233	+	4	0	AGO1-4,AGO2	1	0	1	1	0	0	0	3
MIMAT0002817	hsa-miR-495-3p	ENSG00000146648	EGFR	protein_coding	chr7	55277889	55277894	55277867	55277895	+	2	0	AGO1-4,AGO2	0	0	1	1	0	0	0	3
MIMAT0002819	hsa-miR-193b-3p	ENSG00000146648	EGFR	protein_coding	chr7	55238350	55238355	55238350	55238355	+	2	0	AGO2	1	0	0	0	0	0	0	7
MIMAT0002820	hsa-miR-497-5p	ENSG00000146648	EGFR	protein_coding	chr7	55274319	55274324	55274319	55274324	+	4	0	AGO1-4,AGO2	1	0	1	0	0	0	0	2
MIMAT00028	hsa-miR-181d	ENSG00000146	EGFR	protein_co	chr7	55274197	552742	55274197	552742	+	4	0	AGO1-4,A	1	0	0	0	0	0	0	3

21	-5p	648		ding			02		02				G02								
MIMAT0002823	hsa-miR-512-3p	ENSG00000146648	EGFR	protein_coding	chr7	55273043	55273071	55273043	55273071	+	3	0	AGO1-4,AGO2	0	0	0	1	0	0	0	4
MIMAT0002823	hsa-miR-512-3p	ENSG00000146648	EGFR	protein_coding	chr7	55274521	55274526	55274499	55274527	+	1	1	AGO2	1	0	1	1	0	0	0	4
MIMAT0002823	hsa-miR-512-3p	ENSG00000146648	EGFR	protein_coding	chr7	55276356	55276361	55276334	55276362	+	1	0	AGO1-4	0	0	1	1	0	0	0	4
MIMAT0002823	hsa-miR-512-3p	ENSG00000146648	EGFR	protein_coding	chr7	55277884	55277889	55277862	55277890	+	2	0	AGO1-4,AGO2	0	1	1	1	0	0	0	4
MIMAT0002824	hsa-miR-498	ENSG00000146648	EGFR	protein_coding	chr7	55279112	55279140	55279112	55279140	+	1	0	AGO1-4	0	0	0	1	0	0	0	3
MIMAT0002825	hsa-miR-520e	ENSG00000146648	EGFR	protein_coding	chr7	55274521	55274527	55274500	55274528	+	1	2	AGO2	1	0	1	1	1	0	0	4
MIMAT0002825	hsa-miR-520e	ENSG00000146648	EGFR	protein_coding	chr7	55277884	55277890	55277863	55277891	+	2	0	AGO1-4,AGO2	0	0	1	1	0	0	1	4
MIMAT0002828	hsa-miR-519e-5p	ENSG00000146648	EGFR	protein_coding	chr7	55266472	55266494	55266472	55266494	+	1	0	AGO1-4	0	1	0	0	0	0	0	1
MIMAT	hsa-mi	ENSG00	EGF	prote	chr7	5527	552	5527	552	+	1	2	AGO2	1	0	1	1	0	0	0	4

0002830	R-520f-3p	000146648	R	in_coding		4520	74526	4499	74527											
MIMAT0002830	hsa-miR-520f-3p	ENSG00000146648	EGFR	protein_coding	chr7	55276356	55276361	55276334	55276362	+	1	0	AGO1-4	0	0	1	1	0	0	0	4
MIMAT0002831	hsa-miR-519c-5p	ENSG00000146648	EGFR	protein_coding	chr7	55238548	55238553	55238548	55238553	+	2	0	AGO2	1	0	0	0	0	0	0	0
MIMAT0002832	hsa-miR-519c-3p	ENSG00000146648	EGFR	protein_coding	chr7	55274522	55274527	55274522	55274527	+	1	0	AGO2	1	0	0	0	0	0	0	3
MIMAT0002834	hsa-miR-520a-3p	ENSG00000146648	EGFR	protein_coding	chr7	55274521	55274527	55274500	55274528	+	1	2	AGO2	1	0	1	1	1	0	0	2
MIMAT0002834	hsa-miR-520a-3p	ENSG00000146648	EGFR	protein_coding	chr7	55277884	55277890	55277863	55277891	+	2	0	AGO1-4,AGO2	0	0	1	1	0	0	1	2
MIMAT0002836	hsa-miR-526b-3p	ENSG00000146648	EGFR	protein_coding	chr7	55277885	55277891	55277885	55277891	+	2	0	AGO1-4,AGO2	0	0	0	0	0	0	1	2
MIMAT0002837	hsa-miR-519b-3p	ENSG00000146648	EGFR	protein_coding	chr7	55274522	55274527	55274522	55274527	+	1	0	AGO2	1	0	0	0	0	0	0	1
MIMAT0002842	hsa-miR-518f-3p	ENSG00000146648	EGFR	protein_coding	chr7	55238267	55238272	55238267	55238272	+	1	0	AGO2	1	0	0	0	0	0	0	2

MIMAT0002842	hsa-miR-518f-3p	ENSG00000146648	EGFR	protein_coding	chr7	55274223	55274228	55274223	55274228	+	4	0	AGO1-4,AGO2	1	0	0	0	0	0	0	2
MIMAT0002842	hsa-miR-518f-3p	ENSG00000146648	EGFR	protein_coding	chr7	55274476	55274481	55274476	55274481	+	1	0	AGO2	1	0	0	0	0	0	0	2
MIMAT0002843	hsa-miR-520b	ENSG00000146648	EGFR	protein_coding	chr7	55274521	55274527	55274500	55274528	+	1	2	AGO2	1	0	1	1	1	0	0	2
MIMAT0002843	hsa-miR-520b	ENSG00000146648	EGFR	protein_coding	chr7	55277884	55277890	55277863	55277891	+	2	0	AGO1-4,AGO2	0	0	1	1	0	0	1	2
MIMAT0002844	hsa-miR-518b	ENSG00000146648	EGFR	protein_coding	chr7	55238267	55238272	55238267	55238272	+	1	0	AGO2	1	0	0	0	0	0	0	3
MIMAT0002844	hsa-miR-518b	ENSG00000146648	EGFR	protein_coding	chr7	55274223	55274228	55274223	55274228	+	4	0	AGO1-4,AGO2	1	0	0	0	0	0	0	3
MIMAT0002844	hsa-miR-518b	ENSG00000146648	EGFR	protein_coding	chr7	55274476	55274481	55274476	55274481	+	1	0	AGO2	1	0	0	0	0	0	0	3
MIMAT0002845	hsa-miR-526a	ENSG00000146648	EGFR	protein_coding	chr7	55238548	55238553	55238548	55238553	+	2	0	AGO2	1	0	0	0	0	0	0	0
MIMAT00028	hsa-miR-520c	ENSG00000146	EGFR	protein_co	chr7	55274521	552745	55274500	552745	+	1	2	AGO2	1	0	1	1	1	0	0	2

46	-3p	648		ding			27		28												
MIMAT0002846	hsa-miR-520c-3p	ENSG00000146648	EGFR	protein_coding	chr7	55277884	55277890	55277863	55277891	+	2	0	AGO1-4,AGO2	0	0	1	1	0	0	1	2
MIMAT0002848	hsa-miR-518c-3p	ENSG00000146648	EGFR	protein_coding	chr7	55238267	55238272	55238267	55238272	+	1	0	AGO2	1	0	0	0	0	0	0	1
MIMAT0002848	hsa-miR-518c-3p	ENSG00000146648	EGFR	protein_coding	chr7	55274223	55274228	55274223	55274228	+	4	0	AGO1-4,AGO2	1	0	0	0	0	0	0	1
MIMAT0002848	hsa-miR-518c-3p	ENSG00000146648	EGFR	protein_coding	chr7	55274476	55274481	55274476	55274481	+	1	0	AGO2	1	0	0	0	0	0	0	1
MIMAT0002849	hsa-miR-524-5p	ENSG00000146648	EGFR	protein_coding	chr7	55273969	55273974	55273967	55273974	+	4	0	AGO1-4,AGO2	1	0	1	0	0	0	0	3
MIMAT0002849	hsa-miR-524-5p	ENSG00000146648	EGFR	protein_coding	chr7	55274789	55274795	55274768	55274796	+	2	0	AGO2	1	0	1	1	0	0	0	3
MIMAT0002849	hsa-miR-524-5p	ENSG00000146648	EGFR	protein_coding	chr7	55275710	55275738	55275710	55275738	+	2	0	AGO2	0	0	0	1	0	0	0	3
MIMAT0002849	hsa-miR-524-5p	ENSG00000146648	EGFR	protein_coding	chr7	55277390	55277396	55277369	55277397	+	1	0	AGO1-4	0	0	1	1	0	0	0	3
MIMAT	hsa-mi	ENSG00	EGF	prote	chr7	5527	552	5527	552	+	1	0	AGO1	0	0	0	1	0	0	0	3

0002849	R-524-5p	000146648	R	in_coding		7416	77444	7416	77444				-4								
MIMAT0002849	hsa-miR-524-5p	ENSG00000146648	EGFR	protein_coding	chr7	55279164	55279169	55279142	55279170	+	1	0	AGO1-4	0	0	1	1	0	0	0	3
MIMAT0002853	hsa-miR-519d-3p	ENSG00000146648	EGFR	protein_coding	chr7	55277885	55277891	55277885	55277891	+	2	0	AGO1-4,AGO2	0	0	0	0	0	0	1	2
MIMAT0002855	hsa-miR-520d-5p	ENSG00000146648	EGFR	protein_coding	chr7	55273969	55273974	55273967	55273974	+	4	0	AGO1-4,AGO2	1	0	1	0	0	0	0	1
MIMAT0002855	hsa-miR-520d-5p	ENSG00000146648	EGFR	protein_coding	chr7	55274789	55274795	55274768	55274796	+	2	0	AGO2	1	0	1	1	0	0	0	1
MIMAT0002855	hsa-miR-520d-5p	ENSG00000146648	EGFR	protein_coding	chr7	55275710	55275738	55275710	55275738	+	2	0	AGO2	0	0	0	1	0	0	0	1
MIMAT0002855	hsa-miR-520d-5p	ENSG00000146648	EGFR	protein_coding	chr7	55277390	55277396	55277369	55277397	+	1	0	AGO1-4	0	0	1	1	0	0	0	1
MIMAT0002855	hsa-miR-520d-5p	ENSG00000146648	EGFR	protein_coding	chr7	55277416	55277444	55277416	55277444	+	1	0	AGO1-4	0	0	0	1	0	0	0	1
MIMAT0002855	hsa-miR-520d-5p	ENSG00000146648	EGFR	protein_coding	chr7	55279164	55279169	55279142	55279170	+	1	0	AGO1-4	0	0	1	1	0	0	0	1

MIMAT0002856	hsa-miR-520d-3p	ENSG00000146648	EGFR	protein_coding	chr7	55274521	55274527	55274500	55274528	+	1	2	AGO2	1	0	1	1	1	0	0	2
MIMAT0002856	hsa-miR-520d-3p	ENSG00000146648	EGFR	protein_coding	chr7	55277884	55277890	55277863	55277891	+	2	0	AGO1-4,AGO2	0	0	1	1	0	0	1	2
MIMAT0002858	hsa-miR-520g-3p	ENSG00000146648	EGFR	protein_coding	chr7	55277886	55277892	55277886	55277892	+	2	0	AGO1-4,AGO2	0	0	1	0	0	0	0	3
MIMAT0002859	hsa-miR-516b-5p	ENSG00000146648	EGFR	protein_coding	chr7	55273339	55273344	55273339	55273344	+	3	0	AGO1-4,AGO2	1	0	0	0	0	0	0	3
MIMAT0002859	hsa-miR-516b-5p	ENSG00000146648	EGFR	protein_coding	chr7	55273769	55273776	55273769	55273776	+	1	0	AGO2	1	0	0	0	0	0	0	3
MIMAT0002863	hsa-miR-518a-3p	ENSG00000146648	EGFR	protein_coding	chr7	55238267	55238272	55238267	55238272	+	1	0	AGO2	1	0	0	0	0	0	0	1
MIMAT0002863	hsa-miR-518a-3p	ENSG00000146648	EGFR	protein_coding	chr7	55274223	55274228	55274223	55274228	+	4	0	AGO1-4,AGO2	1	0	0	0	0	0	0	1
MIMAT0002863	hsa-miR-518a-3p	ENSG00000146648	EGFR	protein_coding	chr7	55274476	55274481	55274476	55274481	+	1	0	AGO2	1	0	0	0	0	0	0	1
MIMAT00028	hsa-miR-518d	ENSG00000146	EGFR	protein_co	chr7	55238267	552382	55238267	552382	+	1	0	AGO2	1	0	0	0	0	0	0	3

64	-3p	648		ding			72		72												
MIMAT0002864	hsa-miR-518d-3p	ENSG00000146648	EGFR	protein_coding	chr7	55274223	55274228	55274223	55274228	+	4	0	AGO1-4,AGO2	1	0	0	0	0	0	0	3
MIMAT0002864	hsa-miR-518d-3p	ENSG00000146648	EGFR	protein_coding	chr7	55274476	55274481	55274476	55274481	+	1	0	AGO2	1	0	0	0	0	0	0	3
MIMAT0002867	hsa-miR-520h	ENSG00000146648	EGFR	protein_coding	chr7	55277886	55277892	55277886	55277892	+	2	0	AGO1-4,AGO2	0	0	1	0	0	0	0	4
MIMAT0002869	hsa-miR-519a-3p	ENSG00000146648	EGFR	protein_coding	chr7	55274522	55274527	55274522	55274527	+	1	0	AGO2	1	0	0	0	0	0	0	2
MIMAT0002874	hsa-miR-503-5p	ENSG00000146648	EGFR	protein_coding	chr7	55274319	55274324	55274319	55274324	+	4	0	AGO1-4,AGO2	1	0	1	0	0	0	0	7
MIMAT0002877	hsa-miR-513a-5p	ENSG00000146648	EGFR	protein_coding	chr7	55273511	55273516	55273511	55273516	+	4	0	AGO1-4,AGO2	1	0	1	0	0	0	0	3
MIMAT0002877	hsa-miR-513a-5p	ENSG00000146648	EGFR	protein_coding	chr7	55275944	55275949	55275944	55275949	+	2	0	AGO1-4	0	0	1	0	0	0	0	3
MIMAT0002878	hsa-miR-506-3p	ENSG00000146648	EGFR	protein_coding	chr7	55274501	55274506	55274501	55274506	+	1	0	AGO2	1	0	0	0	0	0	0	5
MIMAT	hsa-mi	ENSG00	EGF	prote	chr7	5523	552	5523	552	+	1	0	AGO2	1	0	1	0	0	0	0	8

0002881	R-509-3p	000146648	R	in_coding		8721	38727	8721	38727												
MIMAT0002882	hsa-miR-510-5p	ENSG00000146648	EGFR	protein_coding	chr7	55279258	55279286	55279258	55279286	+	1	0	AGO1-4	0	0	0	1	0	0	0	2
MIMAT0003150	hsa-miR-455-5p	ENSG00000146648	EGFR	protein_coding	chr7	55273474	55273480	55273456	55273481	+	5	0	AGO1-4,AGO2	1	0	1	0	1	0	0	5
MIMAT0003150	hsa-miR-455-5p	ENSG00000146648	EGFR	protein_coding	chr7	55275503	55275508	55275503	55275508	+	1	0	AGO1-4	0	0	1	0	0	0	0	5
MIMAT0003161	hsa-miR-493-3p	ENSG00000146648	EGFR	protein_coding	chr7	55229298	55229318	55229298	55229318	+	1	0	AGO1-4	0	1	0	0	0	0	0	0
MIMAT0003165	hsa-miR-545-3p	ENSG00000146648	EGFR	protein_coding	chr7	55274094	55274099	55274094	55274099	+	4	0	AGO1-4,AGO2	1	0	0	0	0	0	0	2
MIMAT0003218	hsa-miR-92b-3p	ENSG00000146648	EGFR	protein_coding	chr7	55279123	55279151	55279123	55279151	+	1	0	AGO1-4	0	0	0	1	0	0	0	6
MIMAT0003239	hsa-miR-574-3p	ENSG00000146648	EGFR	protein_coding	chr7	55274028	55274034	55274027	55274034	+	5	0	AGO1-4,AGO2	1	0	1	0	0	0	0	1
MIMAT0003242	hsa-miR-577	ENSG00000146648	EGFR	protein_coding	chr7	55273567	55273573	55273567	55273573	+	1	0	AGO2	1	0	0	0	0	0	0	11

MIMAT0003244	hsa-miR-579-3p	ENSG00000146648	EGFR	protein_coding	chr7	55274312	55274317	55274290	55274318	+	4	2	AGO1-4,AGO2	1	0	0	1	0	0	0	6
MIMAT0003244	hsa-miR-579-3p	ENSG00000146648	EGFR	protein_coding	chr7	55279149	55279177	55279149	55279177	+	1	0	AGO1-4	0	0	0	1	0	0	0	6
MIMAT0003247	hsa-miR-582-5p	ENSG00000146648	EGFR	protein_coding	chr7	55273871	55273876	55273871	55273876	+	1	0	AGO2	1	0	0	0	0	0	0	9
MIMAT0003267	hsa-miR-599	ENSG00000146648	EGFR	protein_coding	chr7	55274485	55274490	55274485	55274490	+	1	0	AGO2	1	0	0	0	0	0	0	6
MIMAT0003281	hsa-miR-613	ENSG00000146648	EGFR	protein_coding	chr7	55273493	55273498	55273471	55273499	+	5	0	AGO1-4,AGO2	1	0	1	1	0	0	0	0
MIMAT0003281	hsa-miR-613	ENSG00000146648	EGFR	protein_coding	chr7	55274056	55274061	55274034	55274062	+	5	0	AGO1-4,AGO2	1	0	1	1	0	0	0	0
MIMAT0003294	hsa-miR-625-5p	ENSG00000146648	EGFR	protein_coding	chr7	55238260	55238266	55238260	55238266	+	1	0	AGO2	1	0	1	0	0	0	0	16
MIMAT0003301	hsa-miR-33b-5p	ENSG00000146648	EGFR	protein_coding	chr7	55238699	55238704	55238686	55238705	+	1	0	AGO2	1	0	0	0	1	0	0	12
MIMAT00033	hsa-miR-33b-	ENSG00000146	EGFR	protein_co	chr7	55274271	552742	55274271	552742	+	2	2	AGO1-4,A	1	0	0	0	0	0	0	12

01	5p	648		ding			76		76				GO2								
MIMAT0003311	hsa-miR-641	ENSG00000146648	EGFR	protein_coding	chr7	55273501	55273506	55273501	55273506	+	4	0	AGO1-4,AGO2	1	0	0	0	0	0	0	4
MIMAT0003311	hsa-miR-641	ENSG00000146648	EGFR	protein_coding	chr7	55274049	55274054	55274049	55274054	+	5	0	AGO1-4,AGO2	1	0	0	0	0	0	0	4
MIMAT0003311	hsa-miR-641	ENSG00000146648	EGFR	protein_coding	chr7	55274090	55274095	55274090	55274095	+	4	0	AGO1-4,AGO2	1	0	0	0	0	0	0	4
MIMAT0003321	hsa-miR-651-5p	ENSG00000146648	EGFR	protein_coding	chr7	55274249	55274254	55274249	55274254	+	2	0	AGO1-4,AGO2	1	0	1	0	0	0	0	8
MIMAT0003321	hsa-miR-651-5p	ENSG00000146648	EGFR	protein_coding	chr7	55274744	55274749	55274744	55274749	+	1	0	AGO2	1	0	1	0	0	0	0	8
MIMAT0003339	hsa-miR-421	ENSG00000146648	EGFR	protein_coding	chr7	55277219	55277225	55277198	55277226	+	2	0	AGO1-4,AGO2	0	0	1	1	0	0	0	7
MIMAT0003339	hsa-miR-421	ENSG00000146648	EGFR	protein_coding	chr7	55279207	55279213	55279186	55279227	+	1	0	AGO1-4	0	0	1	2	0	0	1	7
MIMAT0003339	hsa-miR-421	ENSG00000146648	EGFR	protein_coding	chr7	55279221	55279226	55279221	55279226	+	1	0	AGO1-4	0	0	1	0	0	0	0	7
MIMAT	hsa-mi	ENSG00	EGF	prote	chr7	5527	552	5527	552	+	1	0	AGO2	1	0	0	0	0	0	0	5

0003389	R-542-3p	000146648	R	in_coding		4479	74486	4479	74486												
MIMAT0003393	hsa-miR-425-5p	ENSG00000146648	EGFR	protein_coding	chr7	55274098	55274103	55274098	55274103	+	4	0	AGO1-4,AGO2	1	0	0	0	0	0	0	19
MIMAT0003880	hsa-miR-671-5p	ENSG00000146648	EGFR	protein_coding	chr7	55275849	55275854	55275849	55275854	+	1	0	AGO2	0	0	1	0	0	0	0	12
MIMAT0004185	hsa-miR-802	ENSG00000146648	EGFR	protein_coding	chr7	55274371	55274377	55274371	55274377	+	4	0	AGO1-4,AGO2	1	0	0	0	0	0	0	1
MIMAT0004614	hsa-miR-193a-5p	ENSG00000146648	EGFR	protein_coding	chr7	55273414	55273419	55273414	55273419	+	6	0	AGO1-4,AGO2	1	0	0	0	0	0	0	1
MIMAT0004682	hsa-miR-361-3p	ENSG00000146648	EGFR	protein_coding	chr7	55276688	55276694	55276688	55276694	+	1	0	AGO1	0	0	1	0	0	0	0	11
MIMAT0004682	hsa-miR-361-3p	ENSG00000146648	EGFR	protein_coding	chr7	55277314	55277320	55277314	55277320	+	2	0	AGO1-4,AGO2	0	0	1	0	0	0	0	11
MIMAT0004683	hsa-miR-362-3p	ENSG00000146648	EGFR	protein_coding	chr7	55274753	55274758	55274753	55274758	+	1	0	AGO2	1	0	0	0	0	0	0	10
MIMAT0004687	hsa-miR-371a-5p	ENSG00000146648	EGFR	protein_coding	chr7	55273555	55273560	55273555	55273560	+	2	0	AGO1-4,AGO2	1	0	0	0	0	0	0	4

MIMAT0004687	hsa-miR-371a-5p	ENSG00000146648	EGFR	protein_coding	chr7	55274255	55274260	55274255	55274260	+	2	0	AGO1-4,AGO2	1	0	0	0	0	0	0	4
MIMAT0004692	hsa-miR-340-5p	ENSG00000146648	EGFR	protein_coding	chr7	55275551	55275556	55275529	55275557	+	1	0	AGO1-4	0	0	1	1	0	0	0	7
MIMAT0004692	hsa-miR-340-5p	ENSG00000146648	EGFR	protein_coding	chr7	55277897	55277902	55277875	55277903	+	3	1	AGO1-4,AGO2	0	0	1	1	0	0	0	7
MIMAT0004692	hsa-miR-340-5p	ENSG00000146648	EGFR	protein_coding	chr7	55277934	55277939	55277912	55277940	+	3	0	AGO1-4,AGO2	0	0	1	1	0	0	0	7
MIMAT0004692	hsa-miR-340-5p	ENSG00000146648	EGFR	protein_coding	chr7	55279102	55279108	55279081	55279109	+	1	0	AGO1-4	0	0	1	1	0	0	0	7
MIMAT0004692	hsa-miR-340-5p	ENSG00000146648	EGFR	protein_coding	chr7	55279192	55279197	55279170	55279207	+	1	0	AGO1-4	0	0	1	2	0	0	0	7
MIMAT0004692	hsa-miR-340-5p	ENSG00000146648	EGFR	protein_coding	chr7	55279201	55279206	55279201	55279206	+	1	0	AGO1-4	0	0	1	0	0	0	0	7
MIMAT0004693	hsa-miR-330-5p	ENSG00000146648	EGFR	protein_coding	chr7	55273772	55273777	55273772	55273777	+	1	0	AGO2	1	0	0	0	0	0	0	7
MIMAT00046	hsa-miR-151a	ENSG00000146	EGFR	protein_co	chr7	55238407	552384	55238407	552384	+	2	0	AGO2	1	0	1	0	0	0	0	11

97	-5p	648		ding			13		13												
MIMAT0004748	hsa-miR-423-5p	ENSG00000146648	EGFR	protein_coding	chr7	55238531	55238537	55238530	55238537	+	2	0	AGO2	1	0	1	0	0	0	0	10
MIMAT0004748	hsa-miR-423-5p	ENSG00000146648	EGFR	protein_coding	chr7	55274466	55274471	55274466	55274471	+	2	0	AGO2	1	0	0	0	0	0	0	10
MIMAT0004763	hsa-miR-488-3p	ENSG00000146648	EGFR	protein_coding	chr7	55273572	55273577	55273550	55273578	+	2	0	AGO1-4,AGO2	1	1	1	1	0	0	0	6
MIMAT0004763	hsa-miR-488-3p	ENSG00000146648	EGFR	protein_coding	chr7	55275571	55275576	55275549	55275577	+	2	0	AGO1-4	0	0	1	1	0	0	0	6
MIMAT0004763	hsa-miR-488-3p	ENSG00000146648	EGFR	protein_coding	chr7	55277354	55277359	55277332	55277360	+	1	0	AGO1-4	0	0	1	1	0	0	0	6
MIMAT0004774	hsa-miR-501-3p	ENSG00000146648	EGFR	protein_coding	chr7	55275319	55275325	55275319	55275325	+	1	0	AGO2	0	0	1	0	0	0	0	12
MIMAT0004774	hsa-miR-501-3p	ENSG00000146648	EGFR	protein_coding	chr7	55279229	55279234	55279229	55279234	+	1	0	AGO1-4	0	0	1	0	0	0	0	12
MIMAT0004775	hsa-miR-502-3p	ENSG00000146648	EGFR	protein_coding	chr7	55275319	55275325	55275298	55275326	+	1	0	AGO2	0	0	1	1	0	0	0	10
MIMAT	hsa-mi	ENSG00	EGF	prote	chr7	5527	552	5527	552	+	1	0	AGO1	0	0	1	1	0	0	0	10

0004775	R-502-3p	000146648	R	in_coding		9229	79234	9207	79235				-4								
MIMAT0004784	hsa-miR-455-3p	ENSG00000146648	EGFR	protein_coding	chr7	55238393	55238398	55238393	55238398	+	2	0	AGO2	1	0	0	0	0	0	0	5
MIMAT0004799	hsa-miR-589-5p	ENSG00000146648	EGFR	protein_coding	chr7	55274230	55274235	55274230	55274235	+	3	0	AGO2	1	0	1	0	0	0	0	7
MIMAT0004799	hsa-miR-589-5p	ENSG00000146648	EGFR	protein_coding	chr7	55275862	55275867	55275840	55275868	+	1	0	AGO2	0	0	1	1	0	0	0	7
MIMAT0004805	hsa-miR-616-3p	ENSG00000146648	EGFR	protein_coding	chr7	55274564	55274569	55274564	55274569	+	1	0	AGO2	1	0	0	0	0	0	0	4
MIMAT0004809	hsa-miR-628-5p	ENSG00000146648	EGFR	protein_coding	chr7	55274108	55274113	55274108	55274113	+	3	0	AGO1-4,AGO2	1	0	0	0	0	0	0	10
MIMAT0004809	hsa-miR-628-5p	ENSG00000146648	EGFR	protein_coding	chr7	55274167	55274172	55274167	55274172	+	4	0	AGO1-4,AGO2	1	0	0	0	0	0	0	10
MIMAT0004810	hsa-miR-629-5p	ENSG00000146648	EGFR	protein_coding	chr7	55274462	55274467	55274462	55274467	+	2	0	AGO2	1	0	0	0	0	0	0	8
MIMAT0004911	hsa-miR-874-3p	ENSG00000146648	EGFR	protein_coding	chr7	55274525	55274551	55274525	55274551	+	1	0	AGO2	0	0	0	0	1	0	0	9

MIMAT0004916	hsa-miR-888-5p	ENSG00000146648	EGFR	protein_coding	chr7	55274505	55274510	55274483	55274511	+	2	0	AGO2	1	0	0	1	0	0	0	2
MIMAT0004916	hsa-miR-888-5p	ENSG00000146648	EGFR	protein_coding	chr7	55279212	55279240	55279212	55279240	+	1	0	AGO1-4	0	0	0	1	0	0	0	2
MIMAT0004922	hsa-miR-875-5p	ENSG00000146648	EGFR	protein_coding	chr7	55273580	55273586	55273559	55273587	+	2	0	AGO1-4,AGO2	1	0	1	1	1	0	1	6
MIMAT0004924	hsa-miR-876-5p	ENSG00000146648	EGFR	protein_coding	chr7	55273336	55273341	55273314	55273342	+	3	0	AGO1-4,AGO2	1	0	0	1	0	0	0	4
MIMAT0004924	hsa-miR-876-5p	ENSG00000146648	EGFR	protein_coding	chr7	55277320	55277348	55277320	55277348	+	2	0	AGO1-4,AGO2	0	0	0	1	0	0	0	4
MIMAT0004924	hsa-miR-876-5p	ENSG00000146648	EGFR	protein_coding	chr7	55277791	55277819	55277791	55277819	+	2	0	AGO1-4,AGO2	0	0	0	1	0	0	0	4
MIMAT0004945	hsa-miR-744-5p	ENSG00000146648	EGFR	protein_coding	chr7	55238264	55238269	55238264	55238269	+	1	0	AGO2	1	0	0	0	0	0	0	8
MIMAT0004952	hsa-miR-665	ENSG00000146648	EGFR	protein_coding	chr7	55266469	55266491	55266469	55266491	+	1	0	AGO1-4	0	1	0	0	0	0	0	0
MIMAT00049	hsa-miR-665	ENSG00000146	EGFR	protein_co	chr7	55273917	552739	55273917	552739	+	3	0	AGO1-4,A	1	0	0	0	0	0	0	0

52		648		ding			24		24				G02								
MIMAT0004953	hsa-miR-873-5p	ENSG00000146648	EGFR	protein_coding	chr7	55224596	55224601	55224596	55224601	+	1	0	AGO2	1	0	0	0	0	0	0	5
MIMAT0004953	hsa-miR-873-5p	ENSG00000146648	EGFR	protein_coding	chr7	55273918	55273923	55273918	55273923	+	3	0	AGO1-4,AGO2	1	0	0	0	0	0	0	5
MIMAT0004954	hsa-miR-543	ENSG00000146648	EGFR	protein_coding	chr7	55274909	55274914	55274909	55274914	+	1	0	AGO1-4	1	0	0	0	0	0	0	1
MIMAT0004957	hsa-miR-760	ENSG00000146648	EGFR	protein_coding	chr7	55238549	55238556	55238549	55238556	+	2	0	AGO2	1	0	0	0	0	0	0	4
MIMAT0004957	hsa-miR-760	ENSG00000146648	EGFR	protein_coding	chr7	55274954	55274960	55274954	55274960	+	1	0	AGO1-4	1	0	1	0	0	0	0	4
MIMAT0004985	hsa-miR-942-5p	ENSG00000146648	EGFR	protein_coding	chr7	55275516	55275521	55275516	55275521	+	1	0	AGO1-4	0	0	1	0	0	0	0	11
MIMAT0004985	hsa-miR-942-5p	ENSG00000146648	EGFR	protein_coding	chr7	55276298	55276304	55276298	55276304	+	1	0	AGO2	0	0	1	0	0	0	0	11
MIMAT0004987	hsa-miR-944	ENSG00000146648	EGFR	protein_coding	chr7	55238710	55238716	55238710	55238716	+	1	0	AGO2	1	0	0	0	0	0	0	3
MIMAT	hsa-mi	ENSG00	EGF	prote	chr7	5523	552	5523	552	+	2	0	AGO2	1	0	0	0	0	0	0	0

0005454	R-519b-5p	000146648	R	in_coding		8548	38553	8548	38553												
MIMAT0005455	hsa-miR-520c-5p	ENSG00000146648	EGFR	protein_coding	chr7	55238548	55238553	55238548	55238553	+	2	0	AGO2	1	0	0	0	0	0	0	0
MIMAT0005456	hsa-miR-518d-5p	ENSG00000146648	EGFR	protein_coding	chr7	55238548	55238553	55238548	55238553	+	2	0	AGO2	1	0	0	0	0	0	0	1
MIMAT0005792	hsa-miR-320b	ENSG00000146648	EGFR	protein_coding	chr7	55221790	55221818	55221790	55221818	+	2	0	AGO1-4,AGO2	0	0	0	1	0	0	0	6
MIMAT0005792	hsa-miR-320b	ENSG00000146648	EGFR	protein_coding	chr7	55274415	55274420	55274393	55274421	+	6	0	AGO1-4,AGO2	1	0	0	1	0	0	0	6
MIMAT0005792	hsa-miR-320b	ENSG00000146648	EGFR	protein_coding	chr7	55277366	55277394	55277366	55277394	+	1	0	AGO1-4	0	0	0	1	0	0	0	6
MIMAT0005792	hsa-miR-320b	ENSG00000146648	EGFR	protein_coding	chr7	55279078	55279106	55279078	55279106	+	1	0	AGO1-4	0	0	0	1	0	0	0	6
MIMAT0005793	hsa-miR-320c	ENSG00000146648	EGFR	protein_coding	chr7	55221790	55221818	55221790	55221818	+	2	0	AGO1-4,AGO2	0	0	0	1	0	0	0	1
MIMAT0005793	hsa-miR-320c	ENSG00000146648	EGFR	protein_coding	chr7	55274415	55274420	55274393	55274421	+	6	0	AGO1-4,AGO2	1	0	0	1	0	0	0	1

MIMAT0005793	hsa-miR-320c	ENSG00000146648	EGFR	protein_coding	chr7	55277366	55277394	55277366	55277394	+	1	0	AGO1-4	0	0	0	1	0	0	0	1
MIMAT0005793	hsa-miR-320c	ENSG00000146648	EGFR	protein_coding	chr7	55279078	55279106	55279078	55279106	+	1	0	AGO1-4	0	0	0	1	0	0	0	1
MIMAT0005794	hsa-miR-1296-5p	ENSG00000146648	EGFR	protein_coding	chr7	55273329	55273334	55273329	55273334	+	2	0	AGO1-4,AGO2	1	0	0	0	0	0	0	8
MIMAT0005795	hsa-miR-1323	ENSG00000146648	EGFR	protein_coding	chr7	55273428	55273433	55273406	55273434	+	6	0	AGO1-4,AGO2	1	0	0	1	0	0	0	1
MIMAT0005795	hsa-miR-1323	ENSG00000146648	EGFR	protein_coding	chr7	55274065	55274070	55274043	55274071	+	5	0	AGO1-4,AGO2	1	0	0	1	0	0	0	1
MIMAT0005795	hsa-miR-1323	ENSG00000146648	EGFR	protein_coding	chr7	55279119	55279147	55279119	55279147	+	1	0	AGO1-4	0	0	0	1	0	0	0	1
MIMAT0005800	hsa-miR-1298-5p	ENSG00000146648	EGFR	protein_coding	chr7	55274562	55274568	55274562	55274568	+	1	0	AGO2	1	0	0	0	0	0	0	4
MIMAT0005878	hsa-miR-1287-5p	ENSG00000146648	EGFR	protein_coding	chr7	55273534	55273540	55273513	55273541	+	4	0	AGO1-4,AGO2	1	0	1	1	0	0	0	8
MIMAT00058	hsa-miR-1287	ENSG00000146	EGFR	protein_co	chr7	55274147	552741	55274125	552741	+	2	0	AGO1-4	1	0	0	1	0	0	0	8

78	-5p	648		ding			52		53												
MIMAT0005878	hsa-miR-1287-5p	ENSG00000146648	EGFR	protein_coding	chr7	55276360	55276388	55276360	55276388	+	1	0	AGO1-4	0	0	0	1	0	0	0	8
MIMAT0005885	hsa-miR-1295a	ENSG00000146648	EGFR	protein_coding	chr7	55276648	55276653	55276648	55276653	+	1	0	AGO1	0	0	1	0	0	0	0	0
MIMAT0005885	hsa-miR-1295a	ENSG00000146648	EGFR	protein_coding	chr7	55278297	55278302	55278297	55278302	+	1	0	AGO2	0	0	1	0	0	0	0	0
MIMAT0005903	hsa-miR-1251-5p	ENSG00000146648	EGFR	protein_coding	chr7	55238549	55238554	55238549	55238554	+	2	0	AGO2	1	0	0	0	0	0	0	9
MIMAT0005919	hsa-miR-548o-3p	ENSG00000146648	EGFR	protein_coding	chr7	55273428	55273433	55273406	55273434	+	6	0	AGO1-4,AGO2	1	0	0	1	0	0	0	5
MIMAT0005919	hsa-miR-548o-3p	ENSG00000146648	EGFR	protein_coding	chr7	55274065	55274070	55274043	55274071	+	5	0	AGO1-4,AGO2	1	0	0	1	0	0	0	5
MIMAT0005919	hsa-miR-548o-3p	ENSG00000146648	EGFR	protein_coding	chr7	55279119	55279147	55279119	55279147	+	1	0	AGO1-4	0	0	0	1	0	0	0	5
MIMAT0005923	hsa-miR-1269a	ENSG00000146648	EGFR	protein_coding	chr7	55238501	55238507	55238501	55238507	+	1	0	AGO2	1	0	0	0	0	0	0	6
MIMAT	hsa-mi	ENSG00	EGF	prote	chr7	5527	552	5527	552	+	3	0	AGO1	1	0	1	1	0	0	0	1

00059 30	R-1276	000146 648	R	in_co ding		3345	733 50	3323	733 51				-4, A GO2							
MIMAT 00059 30	hsa-mi R-1276	ENSG00 000146 648	EGF R	prote in_co ding	chr7	5527 7352	552 773 57	5527 7352	552 773 57	+	1	0	AGO1 -4	0	0	1	0	0	0	0	1
MIMAT 00059 30	hsa-mi R-1276	ENSG00 000146 648	EGF R	prote in_co ding	chr7	5527 9188	552 791 93	5527 9166	552 791 94	+	1	0	AGO1 -4	0	0	1	1	0	0	0	1
MIMAT 00059 31	hsa-mi R-302e	ENSG00 000146 648	EGF R	prote in_co ding	chr7	5527 4521	552 745 27	5527 4500	552 745 28	+	1	2	AGO2	1	0	1	1	1	0	0	0
MIMAT 00059 31	hsa-mi R-302e	ENSG00 000146 648	EGF R	prote in_co ding	chr7	5527 7884	552 778 90	5527 7863	552 778 91	+	2	0	AGO1 -4, A GO2	0	0	1	1	0	0	1	0
MIMAT 00059 36	hsa-mi R-1278	ENSG00 000146 648	EGF R	prote in_co ding	chr7	5527 3459	552 734 64	5527 3459	552 734 64	+	5	0	AGO1 -4, A GO2	1	0	0	0	0	0	0	3
MIMAT 00059 44	hsa-mi R-1252 -5p	ENSG00 000146 648	EGF R	prote in_co ding	chr7	5521 0097	552 101 25	5521 0097	552 101 25	+	1	0	AGO2	0	0	0	1	0	0	0	5
MIMAT 00059 44	hsa-mi R-1252 -5p	ENSG00 000146 648	EGF R	prote in_co ding	chr7	5523 8523	552 385 29	5523 8523	552 385 29	+	2	0	AGO2	1	0	1	0	0	0	0	5
MIMAT 00059 51	hsa-mi R-1307 -3p	ENSG00 000146 648	EGF R	prote in_co ding	chr7	5522 9268	552 292 88	5522 9268	552 292 88	+	2	0	AGO1 -4, A GO2	0	1	0	0	0	0	0	11

MIMAT0006764	hsa-miR-320d	ENSG00000146648	EGFR	protein_coding	chr7	55221790	55221818	55221790	55221818	+	2	0	AGO1-4,AGO2	0	0	0	1	0	0	0	1
MIMAT0006764	hsa-miR-320d	ENSG00000146648	EGFR	protein_coding	chr7	55274415	55274420	55274393	55274421	+	6	0	AGO1-4,AGO2	1	0	0	1	0	0	0	1
MIMAT0006764	hsa-miR-320d	ENSG00000146648	EGFR	protein_coding	chr7	55277366	55277394	55277366	55277394	+	1	0	AGO1-4	0	0	0	1	0	0	0	1
MIMAT0006764	hsa-miR-320d	ENSG00000146648	EGFR	protein_coding	chr7	55279078	55279106	55279078	55279106	+	1	0	AGO1-4	0	0	0	1	0	0	0	1
MIMAT0007888	hsa-miR-1913	ENSG00000146648	EGFR	protein_coding	chr7	55275951	55275956	55275951	55275956	+	2	0	AGO1-4	0	0	1	0	0	0	0	1
MIMAT0007888	hsa-miR-1913	ENSG00000146648	EGFR	protein_coding	chr7	55277769	55277775	55277769	55277775	+	1	0	AGO1-4	0	0	1	0	0	0	0	1
MIMAT0010214	hsa-miR-151b	ENSG00000146648	EGFR	protein_coding	chr7	55238407	55238413	55238407	55238413	+	2	0	AGO2	0	0	1	0	0	0	0	6
MIMAT0011157	hsa-miR-2114-3p	ENSG00000146648	EGFR	protein_coding	chr7	55274258	55274264	55274258	55274264	+	2	0	AGO1-4,AGO2	0	0	1	0	0	0	0	4
MIMAT00149	hsa-miR-3118	ENSG00000146	EGFR	protein_co	chr7	55274890	552748	55274869	552748	+	1	1	AGO1-4	0	0	1	1	0	0	0	0

80		648		ding			96		97										
MIMAT0014980	hsa-miR-3118	ENSG00000146648	EGFR	protein_coding	chr7	55275395	55275401	55275374	55275402	+	1	0	AGO2	0	0	1	1	0	0	0	0
MIMAT0014980	hsa-miR-3118	ENSG00000146648	EGFR	protein_coding	chr7	55275702	55275707	55275680	55275708	+	3	0	AGO1-4,AGO2	0	0	1	1	0	0	0	0
MIMAT0014989	hsa-miR-3126-5p	ENSG00000146648	EGFR	protein_coding	chr7	55274440	55274445	55274440	55274445	+	3	0	AGO1-4,AGO2	0	0	1	0	0	0	0	0
MIMAT0014990	hsa-miR-3127-5p	ENSG00000146648	EGFR	protein_coding	chr7	55275334	55275339	55275334	55275339	+	1	0	AGO2	0	0	1	0	0	0	0	7
MIMAT0014990	hsa-miR-3127-5p	ENSG00000146648	EGFR	protein_coding	chr7	55276001	55276007	55276001	55276007	+	2	0	AGO1-4	0	0	1	0	0	0	0	7
MIMAT0014990	hsa-miR-3127-5p	ENSG00000146648	EGFR	protein_coding	chr7	55276037	55276042	55276037	55276042	+	1	0	AGO1-4	0	0	1	0	0	0	0	7
MIMAT0015008	hsa-miR-3140-3p	ENSG00000146648	EGFR	protein_coding	chr7	55274411	55274417	55274390	55274418	+	6	0	AGO1-4,AGO2	0	0	1	1	0	0	0	3
MIMAT0015008	hsa-miR-3140-3p	ENSG00000146648	EGFR	protein_coding	chr7	55277870	55277875	55277848	55277876	+	2	0	AGO1-4,AGO2	0	0	1	1	0	0	0	3
MIMAT	hsa-mi	ENSG00	EGF	prote	chr7	5527	552	5527	552	+	1	0	AGO1	0	0	1	1	0	0	0	3

0015008	R-3140-3p	000146648	R	in_coding		9176	79181	9154	79182				-4								
MIMAT0015011	hsa-miR-3142	ENSG00000146648	EGFR	protein_coding	chr7	55273838	55273844	55273838	55273844	+	2	0	AGO2	0	0	1	0	0	0	0	1
MIMAT0015011	hsa-miR-3142	ENSG00000146648	EGFR	protein_coding	chr7	55275359	55275364	55275359	55275364	+	1	0	AGO2	0	0	1	0	0	0	0	1
MIMAT0015011	hsa-miR-3142	ENSG00000146648	EGFR	protein_coding	chr7	55278296	55278301	55278296	55278301	+	1	0	AGO2	0	0	1	0	0	0	0	1
MIMAT0015015	hsa-miR-3144-3p	ENSG00000146648	EGFR	protein_coding	chr7	55273581	55273587	55273560	55273588	+	2	0	AGO1-4,AGO2	0	0	1	1	0	0	0	1
MIMAT0015037	hsa-miR-3163	ENSG00000146648	EGFR	protein_coding	chr7	55275528	55275556	55275528	55275556	+	1	0	AGO1-4	0	0	0	1	0	0	0	2
MIMAT0015037	hsa-miR-3163	ENSG00000146648	EGFR	protein_coding	chr7	55277254	55277282	55277254	55277282	+	2	0	AGO1-4,AGO2	0	0	0	1	0	0	0	2
MIMAT0015037	hsa-miR-3163	ENSG00000146648	EGFR	protein_coding	chr7	55277911	55277933	55277905	55277939	+	3	0	AGO1-4,AGO2	0	0	0	2	0	0	0	2
MIMAT0015037	hsa-miR-3163	ENSG00000146648	EGFR	protein_coding	chr7	55279178	55279197	55279169	55279206	+	1	0	AGO1-4	0	0	0	2	0	0	0	2

MIMAT0015038	hsa-miR-3164	ENSG00000146648	EGFR	protein_coding	chr7	55274891	55274896	55274891	55274896	+	1	0	AGO1-4	0	0	1	0	0	0	0	4
MIMAT0015038	hsa-miR-3164	ENSG00000146648	EGFR	protein_coding	chr7	55275396	55275401	55275396	55275401	+	1	0	AGO2	0	0	1	0	0	0	0	4
MIMAT0015038	hsa-miR-3164	ENSG00000146648	EGFR	protein_coding	chr7	55275702	55275707	55275702	55275707	+	2	0	AGO2	0	0	1	0	0	0	0	4
MIMAT0015049	hsa-miR-1193	ENSG00000146648	EGFR	protein_coding	chr7	55273382	55273387	55273382	55273387	+	3	0	AGO1-4,AGO2	0	0	1	0	0	0	0	1
MIMAT0015049	hsa-miR-1193	ENSG00000146648	EGFR	protein_coding	chr7	55274831	55274836	55274831	55274836	+	1	0	AGO1-4	0	0	1	0	0	0	0	1
MIMAT0015049	hsa-miR-1193	ENSG00000146648	EGFR	protein_coding	chr7	55276033	55276038	55276033	55276038	+	1	0	AGO1-4	0	0	1	0	0	0	0	1
MIMAT0015064	hsa-miR-3184-5p	ENSG00000146648	EGFR	protein_coding	chr7	55238531	55238537	55238531	55238537	+	2	0	AGO2	0	0	1	0	0	0	0	3
MIMAT0017992	hsa-miR-3614-5p	ENSG00000146648	EGFR	protein_coding	chr7	55229291	55229310	55229291	55229310	+	1	0	AGO1-4	0	1	0	0	0	0	0	3
MIMAT00180	hsa-miR-3622	ENSG00000146	EGFR	protein_co	chr7	55273394	552734	55273394	552734	+	4	0	AGO1-4,A	0	0	1	0	0	0	0	0

05	b-5p	648		ding			00		00				GO2								
MIMAT0018005	hsa-miR-3622b-5p	ENSG00000146648	EGFR	protein_coding	chr7	55276272	55276278	55276272	55276278	+	1	0	AGO2	0	0	1	0	0	0	0	0
MIMAT0018005	hsa-miR-3622b-5p	ENSG00000146648	EGFR	protein_coding	chr7	55277824	55277829	55277824	55277829	+	2	0	AGO1-4,AGO2	0	0	1	0	0	0	0	0
MIMAT0018109	hsa-miR-3681-3p	ENSG00000146648	EGFR	protein_coding	chr7	55273510	55273516	55273510	55273516	+	4	0	AGO1-4,AGO2	0	0	0	0	0	1	0	0
MIMAT0018119	hsa-miR-3690	ENSG00000146648	EGFR	protein_coding	chr7	55274444	55274449	55274444	55274449	+	2	0	AGO2	0	0	1	0	0	0	0	1
MIMAT0018119	hsa-miR-3690	ENSG00000146648	EGFR	protein_coding	chr7	55276338	55276344	55276338	55276344	+	1	0	AGO1-4	0	0	1	0	0	0	0	1
MIMAT0018192	hsa-miR-3918	ENSG00000146648	EGFR	protein_coding	chr7	55274759	55274764	55274759	55274764	+	1	0	AGO2	0	0	1	0	0	0	0	2
MIMAT0018192	hsa-miR-3918	ENSG00000146648	EGFR	protein_coding	chr7	55276001	55276006	55276001	55276006	+	2	0	AGO1-4	0	0	1	0	0	0	0	2
MIMAT0018194	hsa-miR-3150b-3p	ENSG00000146648	EGFR	protein_coding	chr7	55266463	55266483	55266463	55266483	+	1	0	AGO1-4	0	1	0	0	0	0	0	2
MIMAT	hsa-mi	ENSG00	EGF	prote	chr7	5527	552	5527	552	+	4	0	AGO1	0	0	1	0	0	0	0	2

0018943	R-4428	000146648	R	in_coding		4331	74337	4331	74337				-4,AGO2								
MIMAT0018943	hsa-miR-4428	ENSG00000146648	EGFR	protein_coding	chr7	55274393	55274399	55274393	55274399	+	6	0	AGO1-4,AGO2	0	0	1	0	0	0	0	2
MIMAT0018943	hsa-miR-4428	ENSG00000146648	EGFR	protein_coding	chr7	55274469	55274474	55274469	55274474	+	2	0	AGO2	0	0	1	0	0	0	0	2
MIMAT0018944	hsa-miR-4429	ENSG00000146648	EGFR	protein_coding	chr7	55221790	55221818	55221790	55221818	+	2	0	AGO1-4,AGO2	0	0	0	1	0	0	0	0
MIMAT0018944	hsa-miR-4429	ENSG00000146648	EGFR	protein_coding	chr7	55274393	55274421	55274393	55274421	+	6	0	AGO1-4,AGO2	0	0	0	1	0	0	0	0
MIMAT0018944	hsa-miR-4429	ENSG00000146648	EGFR	protein_coding	chr7	55277366	55277394	55277366	55277394	+	1	0	AGO1-4	0	0	0	1	0	0	0	0
MIMAT0018944	hsa-miR-4429	ENSG00000146648	EGFR	protein_coding	chr7	55279078	55279106	55279078	55279106	+	1	0	AGO1-4	0	0	0	1	0	0	0	0
MIMAT0018952	hsa-miR-4436a	ENSG00000146648	EGFR	protein_coding	chr7	55236308	55236313	55236308	55236313	+	1	0	AGO2	0	0	1	0	0	0	0	1
MIMAT0019059	hsa-miR-1269b	ENSG00000146648	EGFR	protein_coding	chr7	55238501	55238507	55238501	55238507	+	1	0	AGO2	0	0	1	0	0	0	0	2

MIMAT0019218	hsa-miR-3194-3p	ENSG00000146648	EGFR	protein_coding	chr7	55274954	55274959	55274954	55274959	+	1	0	AGO1-4	0	0	1	0	0	0	0	5
MIMAT0019759	hsa-miR-4676-3p	ENSG00000146648	EGFR	protein_coding	chr7	55274795	55274801	55274795	55274801	+	3	0	AGO1-4,AGO2	0	0	1	0	0	0	0	3
MIMAT0019776	hsa-miR-1343-3p	ENSG00000146648	EGFR	protein_coding	chr7	55238389	55238395	55238389	55238395	+	2	0	AGO2	0	0	1	0	0	0	0	4
MIMAT0019776	hsa-miR-1343-3p	ENSG00000146648	EGFR	protein_coding	chr7	55273356	55273362	55273356	55273362	+	5	0	AGO1-4,AGO2	0	0	1	0	0	0	0	4
MIMAT0019776	hsa-miR-1343-3p	ENSG00000146648	EGFR	protein_coding	chr7	55273453	55273458	55273453	55273458	+	5	0	AGO1-4,AGO2	0	0	1	0	0	0	0	4
MIMAT0019853	hsa-miR-4731-5p	ENSG00000146648	EGFR	protein_coding	chr7	55273010	55273038	55273010	55273038	+	3	0	AGO1-4,AGO2	0	0	0	1	0	0	0	1
MIMAT0019853	hsa-miR-4731-5p	ENSG00000146648	EGFR	protein_coding	chr7	55273851	55273857	55273830	55273858	+	2	0	AGO2	0	0	1	1	0	0	0	1
MIMAT0019909	hsa-miR-4761-3p	ENSG00000146648	EGFR	protein_coding	chr7	55274758	55274763	55274758	55274763	+	1	0	AGO2	0	0	1	0	0	0	0	1
MIMAT00199	hsa-miR-4761	ENSG00000146	EGFR	protein_co	chr7	55277229	552772	55277229	552772	+	2	0	AGO1-4,A	0	0	1	0	0	0	0	1

09	-3p	648		ding			34		34				GO2								
MIMAT0019953	hsa-miR-2467-3p	ENSG00000146648	EGFR	protein_coding	chr7	55274498	55274503	55274498	55274503	+	1	0	AGO2	0	0	1	0	0	0	0	0
MIMAT0019953	hsa-miR-2467-3p	ENSG00000146648	EGFR	protein_coding	chr7	55275499	55275504	55275499	55275504	+	1	0	AGO1-4	0	0	1	0	0	0	0	0
MIMAT0019953	hsa-miR-2467-3p	ENSG00000146648	EGFR	protein_coding	chr7	55277244	55277249	55277244	55277249	+	2	0	AGO1-4,AGO2	0	0	1	0	0	0	0	0
MIMAT0022272	hsa-miR-664b-3p	ENSG00000146648	EGFR	protein_coding	chr7	55274290	55274318	55274290	55274318	+	4	2	AGO1-4,AGO2	0	0	0	1	0	0	0	11
MIMAT0022272	hsa-miR-664b-3p	ENSG00000146648	EGFR	protein_coding	chr7	55279149	55279177	55279149	55279177	+	1	0	AGO1-4	0	0	0	1	0	0	0	11
MIMAT0022300	hsa-miR-5590-3p	ENSG00000146648	EGFR	protein_coding	chr7	55279102	55279107	55279102	55279107	+	1	0	AGO1-4	0	0	1	0	0	0	0	3
MIMAT0022479	hsa-miR-5688	ENSG00000146648	EGFR	protein_coding	chr7	55274227	55274232	55274205	55274233	+	4	0	AGO1-4,AGO2	0	0	1	1	0	0	0	0
MIMAT0022479	hsa-miR-5688	ENSG00000146648	EGFR	protein_coding	chr7	55277889	55277894	55277867	55277895	+	2	0	AGO1-4,AGO2	0	0	1	1	0	0	0	0
MIMAT	hsa-mi	ENSG00	EGF	prote	chr7	5526	552	5526	552	+	1	0	AGO1	0	1	0	0	0	0	0	4

0022714	R-766-5p	000146648	R	in_coding		6463	66484	6463	66484				-4								
MIMAT0022724	hsa-miR-1277-5p	ENSG00000146648	EGFR	protein_coding	chr7	55273562	55273590	55273562	55273590	+	2	0	AGO1-4,AGO2	0	0	0	1	0	0	0	1
MIMAT0022724	hsa-miR-1277-5p	ENSG00000146648	EGFR	protein_coding	chr7	55274991	55275019	55274991	55275019	+	1	0	AGO1-4	0	0	0	1	0	0	0	1
MIMAT0022724	hsa-miR-1277-5p	ENSG00000146648	EGFR	protein_coding	chr7	55275531	55275559	55275531	55275559	+	1	0	AGO1-4	0	0	0	1	0	0	0	1
MIMAT0022724	hsa-miR-1277-5p	ENSG00000146648	EGFR	protein_coding	chr7	55276302	55276330	55276302	55276330	+	1	0	AGO2	0	0	0	1	0	0	0	1
MIMAT0022724	hsa-miR-1277-5p	ENSG00000146648	EGFR	protein_coding	chr7	55277914	55277942	55277914	55277942	+	3	0	AGO1-4,AGO2	0	0	0	1	0	0	0	1
MIMAT0022844	hsa-miR-216a-3p	ENSG00000146648	EGFR	protein_coding	chr7	55273510	55273515	55273510	55273516	+	4	0	AGO1-4,AGO2	0	0	1	0	0	1	0	3
MIMAT0022844	hsa-miR-216a-3p	ENSG00000146648	EGFR	protein_coding	chr7	55275943	55275948	55275943	55275948	+	2	0	AGO1-4	0	0	1	0	0	0	0	3
MIMAT0022844	hsa-miR-216a-3p	ENSG00000146648	EGFR	protein_coding	chr7	55276358	55276364	55276358	55276364	+	1	0	AGO1-4	0	0	1	0	0	0	0	3

MIMAT0025464	hsa-miR-6504-5p	ENSG00000146648	EGFR	protein_coding	chr7	55238553	55238558	55238553	55238558	+	2	0	AGO2	0	0	1	0	0	0	0	0

c. RNA22

hsa-let-7a-2-3p	hsa-miR-3620-3p	hsa-miR-4768-5p	hsa-miR-6772-5p
hsa-let-7a-5p	hsa-miR-3620-5p	hsa-miR-4768-5p	hsa-miR-6772-5p
hsa-let-7a-5p	hsa-miR-3620-5p	hsa-miR-4769-3p	hsa-miR-6772-5p
hsa-let-7a-5p	hsa-miR-3620-5p	hsa-miR-4769-3p	hsa-miR-6772-5p
hsa-let-7a-5p	hsa-miR-3620-5p	hsa-miR-4769-5p	hsa-miR-6772-5p
hsa-let-7b-5p	hsa-miR-3620-5p	hsa-miR-4769-5p	hsa-miR-6772-5p
hsa-let-7b-5p	hsa-miR-3621	hsa-miR-4769-5p	hsa-miR-6772-5p
hsa-let-7b-5p	hsa-miR-3621	hsa-miR-4769-5p	hsa-miR-6772-5p
hsa-let-7b-5p	hsa-miR-3621	hsa-miR-4772-5p	hsa-miR-6772-5p
hsa-let-7b-5p	hsa-miR-3621	hsa-miR-4774-3p	hsa-miR-6772-5p
hsa-let-7b-5p	hsa-miR-3622a-3p	hsa-miR-4774-5p	hsa-miR-6772-5p
hsa-let-7b-5p	hsa-miR-3622a-3p	hsa-miR-4774-5p	hsa-miR-6773-3p
hsa-let-7c-5p	hsa-miR-3622a-5p	hsa-miR-4776-3p	hsa-miR-6773-3p
hsa-let-7c-5p	hsa-miR-3622b-3p	hsa-miR-4776-3p	hsa-miR-6773-5p
hsa-let-7c-5p	hsa-miR-3622b-3p	hsa-miR-4776-5p	hsa-miR-6773-5p
hsa-let-7c-5p	hsa-miR-3622b-5p	hsa-miR-4776-5p	hsa-miR-6774-3p
hsa-let-7d-5p	hsa-miR-3622b-5p	hsa-miR-4776-5p	hsa-miR-6774-5p
hsa-let-7d-5p	hsa-miR-363-5p	hsa-miR-4778-3p	hsa-miR-6774-5p
hsa-let-7d-5p	hsa-miR-363-5p	hsa-miR-4778-3p	hsa-miR-6774-5p
hsa-let-7d-5p	hsa-miR-363-5p	hsa-miR-4778-3p	hsa-miR-6774-5p
hsa-let-7e-5p	hsa-miR-3648	hsa-miR-4778-3p	hsa-miR-6775-3p
hsa-let-7e-5p	hsa-miR-3648	hsa-miR-4778-3p	hsa-miR-6775-3p
hsa-let-7e-5p	hsa-miR-3648	hsa-miR-4783-3p	hsa-miR-6775-3p
hsa-let-7e-5p	hsa-miR-3649	hsa-miR-4783-3p	hsa-miR-6775-3p
hsa-let-7f-5p	hsa-miR-3650	hsa-miR-4783-5p	hsa-miR-6775-5p
hsa-let-7g-3p	hsa-miR-3650	hsa-miR-4783-5p	hsa-miR-6775-5p
hsa-let-7g-5p	hsa-miR-3650	hsa-miR-4783-5p	hsa-miR-6775-5p
hsa-let-7g-5p	hsa-miR-3650	hsa-miR-4783-5p	hsa-miR-6776-5p
hsa-let-7g-5p	hsa-miR-3651	hsa-miR-4784	hsa-miR-6776-5p
hsa-let-7i-3p	hsa-miR-3651	hsa-miR-4784	hsa-miR-6776-5p
hsa-let-7i-3p	hsa-miR-3652	hsa-miR-4784	hsa-miR-6776-5p
hsa-let-7i-3p	hsa-miR-3652	hsa-miR-4784	hsa-miR-6776-5p
hsa-let-7i-5p	hsa-miR-3652	hsa-miR-4785	hsa-miR-6776-5p
hsa-let-7i-5p	hsa-miR-3652	hsa-miR-4785	hsa-miR-6776-5p
hsa-let-7i-5p	hsa-miR-3652	hsa-miR-4787-3p	hsa-miR-6776-5p
hsa-let-7i-5p	hsa-miR-3653-5p	hsa-miR-4787-3p	hsa-miR-6777-3p
hsa-let-7i-5p	hsa-miR-3653-5p	hsa-miR-4787-5p	hsa-miR-6777-5p
hsa-let-7i-5p	hsa-miR-3655	hsa-miR-4787-5p	hsa-miR-6777-5p
hsa-let-7i-5p	hsa-miR-3655	hsa-miR-4787-5p	hsa-miR-6777-5p
hsa-let-7i-5p	hsa-miR-3655	hsa-miR-4787-5p	hsa-miR-6777-5p
hsa-miR-103a-2-5	hsa-miR-3655	hsa-miR-4787-5p	hsa-miR-6777-5p

hsa-miR-103a-3p	hsa-miR-3655	hsa-miR-4787-5p	hsa-miR-6778-3p
hsa-miR-103a-3p	hsa-miR-3655	hsa-miR-4792	hsa-miR-6778-3p
hsa-miR-105-3p	hsa-miR-3656	hsa-miR-4792	hsa-miR-6778-5p
hsa-miR-106a-5p	hsa-miR-3656	hsa-miR-4792	hsa-miR-6778-5p
hsa-miR-106b-3p	hsa-miR-3656	hsa-miR-4792	hsa-miR-6779-5p
hsa-miR-106b-3p	hsa-miR-3656	hsa-miR-4792	hsa-miR-6779-5p
hsa-miR-106b-5p	hsa-miR-3656	hsa-miR-4793-3p	hsa-miR-6779-5p
hsa-miR-107 E	hsa-miR-3656	hsa-miR-4793-5p	hsa-miR-6779-5p
hsa-miR-107 E	hsa-miR-3657	hsa-miR-4794	hsa-miR-6779-5p
hsa-miR-10a-5p	hsa-miR-365a-5p	hsa-miR-4794	hsa-miR-6779-5p
hsa-miR-1178-3p	hsa-miR-365a-5p	hsa-miR-4798-5p	hsa-miR-6780a-3p
hsa-miR-1178-5p	hsa-miR-365b-5p	hsa-miR-4798-5p	hsa-miR-6780a-5p
hsa-miR-1178-5p	hsa-miR-365b-5p	hsa-miR-4800-5p	hsa-miR-6780a-5p
hsa-miR-1178-5p	hsa-miR-365b-5p	hsa-miR-4800-5p	hsa-miR-6780a-5p
hsa-miR-1180-3p	hsa-miR-3661	hsa-miR-4804-5p	hsa-miR-6780a-5p
hsa-miR-1180-5p	hsa-miR-3661	hsa-miR-483-5p	hsa-miR-6780a-5p
hsa-miR-1182	hsa-miR-3663-5p	hsa-miR-484 E	hsa-miR-6780a-5p
hsa-miR-1182	hsa-miR-3663-5p	hsa-miR-485-3p	hsa-miR-6780a-5p
hsa-miR-1183	hsa-miR-3663-5p	hsa-miR-485-3p	hsa-miR-6780b-3p
hsa-miR-1183	hsa-miR-3663-5p	hsa-miR-485-5p	hsa-miR-6781-5p
hsa-miR-1183	hsa-miR-3663-5p	hsa-miR-485-5p	hsa-miR-6781-5p
hsa-miR-1184	hsa-miR-3663-5p	hsa-miR-485-5p	hsa-miR-6782-5p
hsa-miR-1184	hsa-miR-3663-5p	hsa-miR-486-3p	hsa-miR-6782-5p
hsa-miR-1184	hsa-miR-3663-5p	hsa-miR-486-3p	hsa-miR-6783-3p
hsa-miR-1193	hsa-miR-3665	hsa-miR-486-3p	hsa-miR-6783-3p
hsa-miR-1193	hsa-miR-3665	hsa-miR-486-3p	hsa-miR-6783-5p
hsa-miR-1193	hsa-miR-3675-3p	hsa-miR-486-3p	hsa-miR-6784-5p
hsa-miR-1193	hsa-miR-3677-3p	hsa-miR-486-5p	hsa-miR-6784-5p
hsa-miR-1193	hsa-miR-3677-3p	hsa-miR-486-5p	hsa-miR-6784-5p
hsa-miR-1193	hsa-miR-3677-3p	hsa-miR-487a-5p	hsa-miR-6785-5p
hsa-miR-1193	hsa-miR-3677-3p	hsa-miR-489-5p	hsa-miR-6785-5p
hsa-miR-1199-3p	hsa-miR-3677-3p	hsa-miR-489-5p	hsa-miR-6785-5p
hsa-miR-1199-5p	hsa-miR-3677-5p	hsa-miR-489-5p	hsa-miR-6785-5p
hsa-miR-1199-5p	hsa-miR-3677-5p	hsa-miR-490-5p	hsa-miR-6785-5p
hsa-miR-1200	hsa-miR-3677-5p	hsa-miR-490-5p	hsa-miR-6785-5p
hsa-miR-1200	hsa-miR-3678-3p	hsa-miR-490-5p	hsa-miR-6785-5p
hsa-miR-1202	hsa-miR-3679-5p	hsa-miR-491-5p	hsa-miR-6785-5p
hsa-miR-1202	hsa-miR-3679-5p	hsa-miR-492 E	hsa-miR-6785-5p
hsa-miR-1202	hsa-miR-3679-5p	hsa-miR-492 E	hsa-miR-6786-3p
hsa-miR-1203	hsa-miR-3679-5p	hsa-miR-492 E	hsa-miR-6786-5p
hsa-miR-1204	hsa-miR-3680-3p	hsa-miR-493-3p	hsa-miR-6786-5p
hsa-miR-1204	hsa-miR-3680-3p	hsa-miR-493-3p	hsa-miR-6786-5p
hsa-miR-1204	hsa-miR-3682-3p	hsa-miR-493-5p	hsa-miR-6786-5p

hsa-miR-1205	hsa-miR-3682-3p	hsa-miR-493-5p	hsa-miR-6787-5p
hsa-miR-1205	hsa-miR-3682-5p	hsa-miR-494-5p	hsa-miR-6787-5p
hsa-miR-1205	hsa-miR-3683	hsa-miR-494-5p	hsa-miR-6787-5p
hsa-miR-1207-3p	hsa-miR-3685	hsa-miR-494-5p	hsa-miR-6787-5p
hsa-miR-1207-5p	hsa-miR-3687	hsa-miR-494-5p	hsa-miR-6787-5p
hsa-miR-1207-5p	hsa-miR-3687	hsa-miR-494-5p	hsa-miR-6787-5p
hsa-miR-1207-5p	hsa-miR-3687	hsa-miR-494-5p	hsa-miR-6787-5p
hsa-miR-1207-5p	hsa-miR-3689a-3p	hsa-miR-494-5p	hsa-miR-6787-5p
hsa-miR-1207-5p	hsa-miR-3689a-3p	hsa-miR-494-5p	hsa-miR-6787-5p
hsa-miR-1207-5p	hsa-miR-3689a-3p	hsa-miR-494-5p	hsa-miR-6787-5p
hsa-miR-1208	hsa-miR-3689a-3p	hsa-miR-496 E	hsa-miR-6787-5p
hsa-miR-1208	hsa-miR-3689a-3p	hsa-miR-497-5p	hsa-miR-6788-3p
hsa-miR-1208	hsa-miR-3689a-3p	hsa-miR-4999-5p	hsa-miR-6788-3p
hsa-miR-1224-5p	hsa-miR-3689b-3p	hsa-miR-4999-5p	hsa-miR-6788-3p
hsa-miR-1224-5p	hsa-miR-3689b-3p	hsa-miR-5000-3p	hsa-miR-6788-5p
hsa-miR-1225-3p	hsa-miR-3689b-3p	hsa-miR-5000-3p	hsa-miR-6788-5p
hsa-miR-1225-5p	hsa-miR-3689b-3p	hsa-miR-5000-5p	hsa-miR-6789-3p
hsa-miR-1225-5p	hsa-miR-3689b-3p	hsa-miR-5001-3p	hsa-miR-6789-3p
hsa-miR-1226-3p	hsa-miR-3689c	hsa-miR-5001-3p	hsa-miR-6789-5p
hsa-miR-1226-5p	hsa-miR-3689c	hsa-miR-5001-5p	hsa-miR-6789-5p
hsa-miR-1226-5p	hsa-miR-3689c	hsa-miR-5001-5p	hsa-miR-6789-5p
hsa-miR-1226-5p	hsa-miR-3689c	hsa-miR-5001-5p	hsa-miR-6789-5p
hsa-miR-1226-5p	hsa-miR-3689c	hsa-miR-5001-5p	hsa-miR-6789-5p
hsa-miR-1226-5p	hsa-miR-3689d	hsa-miR-5001-5p	hsa-miR-6790-5p
hsa-miR-1226-5p	hsa-miR-3689d	hsa-miR-5001-5p	hsa-miR-6790-5p
hsa-miR-1227-5p	hsa-miR-3689d	hsa-miR-5001-5p	hsa-miR-6790-5p
hsa-miR-1227-5p	hsa-miR-3689f	hsa-miR-5001-5p	hsa-miR-6791-3p
hsa-miR-1227-5p	hsa-miR-3691-3p	hsa-miR-5001-5p	hsa-miR-6791-3p
hsa-miR-1227-5p	hsa-miR-3691-5p	hsa-miR-5001-5p	hsa-miR-6791-5p
hsa-miR-1227-5p	hsa-miR-3691-5p	hsa-miR-5002-3p	hsa-miR-6791-5p
hsa-miR-1227-5p	hsa-miR-3692-5p	hsa-miR-5003-3p	hsa-miR-6791-5p
hsa-miR-1227-5p	hsa-miR-3692-5p	hsa-miR-5004-3p	hsa-miR-6792-3p
hsa-miR-1227-5p	hsa-miR-3692-5p	hsa-miR-5004-3p	hsa-miR-6792-3p
hsa-miR-1227-5p	hsa-miR-3692-5p	hsa-miR-5004-3p	hsa-miR-6793-5p
hsa-miR-1228-5p	hsa-miR-370-3p	hsa-miR-5004-5p	hsa-miR-6793-5p
hsa-miR-1228-5p	hsa-miR-370-3p	hsa-miR-5004-5p	hsa-miR-6793-5p
hsa-miR-1228-5p	hsa-miR-370-3p	hsa-miR-5006-3p	hsa-miR-6793-5p
hsa-miR-1228-5p	hsa-miR-370-3p	hsa-miR-5006-5p	hsa-miR-6793-5p
hsa-miR-1229-3p	hsa-miR-370-3p	hsa-miR-5007-5p	hsa-miR-6794-3p
hsa-miR-1229-5p	hsa-miR-3713	hsa-miR-5007-5p	hsa-miR-6794-5p
hsa-miR-1229-5p	hsa-miR-372-3p	hsa-miR-5008-3p	hsa-miR-6794-5p
hsa-miR-1229-5p	hsa-miR-373-3p	hsa-miR-5008-3p	hsa-miR-6795-5p
hsa-miR-1229-5p	hsa-miR-374b-3p	hsa-miR-5008-3p	hsa-miR-6795-5p

hsa-miR-1229-5p	hsa-miR-375 E	hsa-miR-5008-3p	hsa-miR-6795-5p
hsa-miR-122-5p	hsa-miR-375 E	hsa-miR-5008-5p	hsa-miR-6795-5p
hsa-miR-122-5p	hsa-miR-376a-2-5	hsa-miR-5008-5p	hsa-miR-6795-5p
hsa-miR-122-5p	hsa-miR-376a-5p	hsa-miR-5009-5p	hsa-miR-6795-5p
hsa-miR-122-5p	hsa-miR-376c-3p	hsa-miR-5009-5p	hsa-miR-6795-5p
hsa-miR-122-5p	hsa-miR-377-3p	hsa-miR-500a-3p	hsa-miR-6795-5p
hsa-miR-1231	hsa-miR-377-5p	hsa-miR-500a-3p	hsa-miR-6796-5p
hsa-miR-1231	hsa-miR-377-5p	hsa-miR-5010-3p	hsa-miR-6796-5p
hsa-miR-1231	hsa-miR-377-5p	hsa-miR-5010-3p	hsa-miR-6796-5p
hsa-miR-1231	hsa-miR-378a-5p	hsa-miR-5010-5p	hsa-miR-6796-5p
hsa-miR-1231	hsa-miR-378b	hsa-miR-5010-5p	hsa-miR-6796-5p
hsa-miR-1231	hsa-miR-378e	hsa-miR-5011-3p	hsa-miR-6796-5p
hsa-miR-1231	hsa-miR-378g	hsa-miR-501-5p	hsa-miR-6796-5p
hsa-miR-1231	hsa-miR-378g	hsa-miR-502-5p	hsa-miR-6796-5p
hsa-miR-1233-3p	hsa-miR-381-5p	hsa-miR-502-5p	hsa-miR-6796-5p
hsa-miR-1233-3p	hsa-miR-381-5p	hsa-miR-502-5p	hsa-miR-6796-5p
hsa-miR-1233-5p	hsa-miR-382-5p	hsa-miR-502-5p	hsa-miR-6796-5p
hsa-miR-1233-5p	hsa-miR-384 E	hsa-miR-503-3p	hsa-miR-6797-5p
hsa-miR-1233-5p	hsa-miR-3907	hsa-miR-503-3p	hsa-miR-6797-5p
hsa-miR-1233-5p	hsa-miR-3907	hsa-miR-503-3p	hsa-miR-6797-5p
hsa-miR-1236-3p	hsa-miR-3907	hsa-miR-503-3p	hsa-miR-6797-5p
hsa-miR-1236-3p	hsa-miR-3907	hsa-miR-503-3p	hsa-miR-6797-5p
hsa-miR-1236-5p	hsa-miR-3907	hsa-miR-503-3p	hsa-miR-6797-5p
hsa-miR-1237-5p	hsa-miR-3909	hsa-miR-503-3p	hsa-miR-6798-3p
hsa-miR-1237-5p	hsa-miR-3909	hsa-miR-5047	hsa-miR-6798-5p
hsa-miR-1237-5p	hsa-miR-3909	hsa-miR-5047	hsa-miR-6798-5p
hsa-miR-1237-5p	hsa-miR-3909	hsa-miR-504-3p	hsa-miR-6798-5p
hsa-miR-1237-5p	hsa-miR-3909	hsa-miR-504-3p	hsa-miR-6798-5p
hsa-miR-1237-5p	hsa-miR-3911	hsa-miR-504-3p	hsa-miR-6798-5p
hsa-miR-1237-5p	hsa-miR-3911	hsa-miR-504-3p	hsa-miR-6799-3p
hsa-miR-1238-3p	hsa-miR-3911	hsa-miR-504-3p	hsa-miR-6799-3p
hsa-miR-1238-3p	hsa-miR-3911	hsa-miR-504-5p	hsa-miR-6799-5p
hsa-miR-1238-3p	hsa-miR-3911	hsa-miR-504-5p	hsa-miR-6799-5p
hsa-miR-1238-3p	hsa-miR-3911	hsa-miR-504-5p	hsa-miR-6799-5p
hsa-miR-1238-5p	hsa-miR-3912-5p	hsa-miR-505-3p	hsa-miR-6799-5p
hsa-miR-1238-5p	hsa-miR-3913-5p	hsa-miR-505-5p	hsa-miR-6799-5p
hsa-miR-1245b-3p	hsa-miR-3917	hsa-miR-507 E	hsa-miR-6799-5p
hsa-miR-1245b-3p	hsa-miR-3917	hsa-miR-5087	hsa-miR-6799-5p
hsa-miR-1248	hsa-miR-3921	hsa-miR-5087	hsa-miR-6800-5p
hsa-miR-1248	hsa-miR-3921	hsa-miR-5087	hsa-miR-6800-5p
hsa-miR-1249-5p	hsa-miR-3922-3p	hsa-miR-5087	hsa-miR-6800-5p
hsa-miR-1249-5p	hsa-miR-3922-3p	hsa-miR-5087	hsa-miR-6801-3p
hsa-miR-1249-5p	hsa-miR-3922-3p	hsa-miR-5087	hsa-miR-6801-5p

hsa-miR-1249-5p	hsa-miR-3922-5p	hsa-miR-5087	hsa-miR-6801-5p
hsa-miR-1249-5p	hsa-miR-3922-5p	hsa-miR-5087	hsa-miR-6801-5p
hsa-miR-1249-5p	hsa-miR-3927-3p	hsa-miR-5087	hsa-miR-6802-5p
hsa-miR-1249-5p	hsa-miR-3928-5p	hsa-miR-5088-3p	hsa-miR-6802-5p
hsa-miR-124-3p	hsa-miR-3929	hsa-miR-5088-5p	hsa-miR-6802-5p
hsa-miR-124-5p	hsa-miR-3934-3p	hsa-miR-5088-5p	hsa-miR-6803-3p
hsa-miR-1250-5p	hsa-miR-3934-3p	hsa-miR-5088-5p	hsa-miR-6803-3p
hsa-miR-1250-5p	hsa-miR-3934-3p	hsa-miR-5088-5p	hsa-miR-6803-3p
hsa-miR-1250-5p	hsa-miR-3934-3p	hsa-miR-5089-3p	hsa-miR-6803-5p
hsa-miR-1251-3p	hsa-miR-3934-3p	hsa-miR-5089-5p	hsa-miR-6803-5p
hsa-miR-1251-3p	hsa-miR-3937	hsa-miR-508-5p	hsa-miR-6803-5p
hsa-miR-1251-3p	hsa-miR-3940-5p	hsa-miR-5090	hsa-miR-6803-5p
hsa-miR-1254	hsa-miR-3940-5p	hsa-miR-5090	hsa-miR-6803-5p
hsa-miR-1255a	hsa-miR-3940-5p	hsa-miR-5090	hsa-miR-6803-5p
hsa-miR-1255b-5p	hsa-miR-3940-5p	hsa-miR-5090	hsa-miR-6803-5p
hsa-miR-1257	hsa-miR-3940-5p	hsa-miR-5095	hsa-miR-6803-5p
hsa-miR-1258	hsa-miR-3940-5p	hsa-miR-5096	hsa-miR-6803-5p
hsa-miR-1258	hsa-miR-3940-5p	hsa-miR-5096	hsa-miR-6803-5p
hsa-miR-125a-3p	hsa-miR-3940-5p	hsa-miR-5096	hsa-miR-6804-3p
hsa-miR-125a-3p	hsa-miR-3940-5p	hsa-miR-509-3p	hsa-miR-6804-3p
hsa-miR-125a-5p	hsa-miR-3944-3p	hsa-miR-509-5p	hsa-miR-6804-5p
hsa-miR-125a-5p	hsa-miR-3944-3p	hsa-miR-5100	hsa-miR-6804-5p
hsa-miR-125b-1-3	hsa-miR-3944-3p	hsa-miR-511-5p	hsa-miR-6804-5p
hsa-miR-125b-1-3	hsa-miR-3944-3p	hsa-miR-511-5p	hsa-miR-6804-5p
hsa-miR-1261	hsa-miR-3944-3p	hsa-miR-512-3p	hsa-miR-6805-3p
hsa-miR-1262	hsa-miR-3944-5p	hsa-miR-514b-5p	hsa-miR-6805-3p
hsa-miR-1262	hsa-miR-3945	hsa-miR-515-3p	hsa-miR-6805-5p
hsa-miR-1262	hsa-miR-3945	hsa-miR-515-5p	hsa-miR-6805-5p
hsa-miR-1263	hsa-miR-3945	hsa-miR-516a-3p	hsa-miR-6805-5p
hsa-miR-1263	hsa-miR-3945	hsa-miR-516a-5p	hsa-miR-6805-5p
hsa-miR-1263	hsa-miR-3945	hsa-miR-516b-3p	hsa-miR-6806-3p
hsa-miR-1265	hsa-miR-3960	hsa-miR-516b-5p	hsa-miR-6806-5p
hsa-miR-1265	hsa-miR-3960	hsa-miR-516b-5p	hsa-miR-6806-5p
hsa-miR-1266-3p	hsa-miR-3960	hsa-miR-517-5p	hsa-miR-6806-5p
hsa-miR-1266-3p	hsa-miR-3960	hsa-miR-517-5p	hsa-miR-6806-5p
hsa-miR-1266-3p	hsa-miR-3960	hsa-miR-517a-3p	hsa-miR-6806-5p
hsa-miR-1266-3p	hsa-miR-3960	hsa-miR-517a-3p	hsa-miR-6806-5p
hsa-miR-1266-5p	hsa-miR-3972	hsa-miR-517b-3p	hsa-miR-6807-3p
hsa-miR-1268a	hsa-miR-3972	hsa-miR-517b-3p	hsa-miR-6807-3p
hsa-miR-1268a	hsa-miR-3974	hsa-miR-517c-3p	hsa-miR-6807-5p
hsa-miR-1268a	hsa-miR-3975	hsa-miR-5187-5p	hsa-miR-6807-5p
hsa-miR-1268a	hsa-miR-3975	hsa-miR-5187-5p	hsa-miR-6808-3p
hsa-miR-1268a	hsa-miR-3978	hsa-miR-5189-5p	hsa-miR-6808-5p

hsa-miR-1268a	hsa-miR-409-3p	hsa-miR-5189-5p	hsa-miR-6809-3p
hsa-miR-1268a	hsa-miR-409-5p	hsa-miR-5189-5p	hsa-miR-6809-5p
hsa-miR-1268a	hsa-miR-410-5p	hsa-miR-5189-5p	hsa-miR-6810-3p
hsa-miR-1268b	hsa-miR-410-5p	hsa-miR-5189-5p	hsa-miR-6810-5p
hsa-miR-1268b	hsa-miR-410-5p	hsa-miR-518c-5p	hsa-miR-6810-5p
hsa-miR-1268b	hsa-miR-410-5p	hsa-miR-518c-5p	hsa-miR-6810-5p
hsa-miR-1268b	hsa-miR-410-5p	hsa-miR-518c-5p	hsa-miR-6810-5p
hsa-miR-1268b	hsa-miR-410-5p	hsa-miR-518d-5p	hsa-miR-6810-5p
hsa-miR-1268b	hsa-miR-410-5p	hsa-miR-518e-5p	hsa-miR-6811-3p
hsa-miR-1269a	hsa-miR-412-3p	hsa-miR-518f-5p	hsa-miR-6811-5p
hsa-miR-1269a	hsa-miR-422a	hsa-miR-5190	hsa-miR-6812-5p
hsa-miR-1269a	hsa-miR-422a	hsa-miR-5191	hsa-miR-6812-5p
hsa-miR-1269a	hsa-miR-422a	hsa-miR-5192	hsa-miR-6812-5p
hsa-miR-1269a	hsa-miR-423-3p	hsa-miR-5192	hsa-miR-6812-5p
hsa-miR-1269b	hsa-miR-423-5p	hsa-miR-5192	hsa-miR-6812-5p
hsa-miR-1269b	hsa-miR-423-5p	hsa-miR-5192	hsa-miR-6813-5p
hsa-miR-126-5p	hsa-miR-423-5p	hsa-miR-5194	hsa-miR-6813-5p
hsa-miR-126-5p	hsa-miR-423-5p	hsa-miR-5194	hsa-miR-6813-5p
hsa-miR-1270	hsa-miR-423-5p	hsa-miR-5194	hsa-miR-6813-5p
hsa-miR-1271-3p	hsa-miR-4252	hsa-miR-5194	hsa-miR-6814-3p
hsa-miR-1271-3p	hsa-miR-4252	hsa-miR-5194	hsa-miR-6814-5p
hsa-miR-1271-3p	hsa-miR-4252	hsa-miR-5195-5p	hsa-miR-6815-3p
hsa-miR-1271-5p	hsa-miR-4253	hsa-miR-5196-3p	hsa-miR-6815-5p
hsa-miR-1271-5p	hsa-miR-4253	hsa-miR-5196-5p	hsa-miR-6815-5p
hsa-miR-1271-5p	hsa-miR-4253	hsa-miR-5196-5p	hsa-miR-6816-5p
hsa-miR-1272	hsa-miR-4253	hsa-miR-5196-5p	hsa-miR-6816-5p
hsa-miR-1273a	hsa-miR-4254	hsa-miR-519a-5p	hsa-miR-6816-5p
hsa-miR-1273d	hsa-miR-4254	hsa-miR-519b-5p	hsa-miR-6816-5p
hsa-miR-1273f	hsa-miR-4254	hsa-miR-519c-5p	hsa-miR-6816-5p
hsa-miR-1273f	hsa-miR-4255	hsa-miR-519d-5p	hsa-miR-6817-3p
hsa-miR-1273g-3p	hsa-miR-4256	hsa-miR-519e-5p	hsa-miR-6817-3p
hsa-miR-1273g-5p	hsa-miR-4257	hsa-miR-520a-5p	hsa-miR-6817-5p
hsa-miR-1273g-5p	hsa-miR-4257	hsa-miR-520c-5p	hsa-miR-6818-3p
hsa-miR-1273g-5p	hsa-miR-4258	hsa-miR-520g-5p	hsa-miR-6818-3p
hsa-miR-1273g-5p	hsa-miR-4259	hsa-miR-520g-5p	hsa-miR-6818-3p
hsa-miR-1273g-5p	hsa-miR-4259	hsa-miR-520g-5p	hsa-miR-6818-3p
hsa-miR-1273g-5p	hsa-miR-425-3p	hsa-miR-522-5p	hsa-miR-6818-3p
hsa-miR-1273g-5p	hsa-miR-4260	hsa-miR-523-5p	hsa-miR-6818-5p
hsa-miR-1273h-3p	hsa-miR-4260	hsa-miR-525-5p	hsa-miR-6818-5p
hsa-miR-1273h-5p	hsa-miR-4260	hsa-miR-526a	hsa-miR-6818-5p
hsa-miR-1273h-5p	hsa-miR-4260	hsa-miR-526b-5p	hsa-miR-6818-5p
hsa-miR-1273h-5p	hsa-miR-4260	hsa-miR-526b-5p	hsa-miR-6819-3p
hsa-miR-1273h-5p	hsa-miR-4260	hsa-miR-526b-5p	hsa-miR-6819-5p

hsa-miR-1273h-5p	hsa-miR-4265	hsa-miR-532-5p	hsa-miR-6819-5p
hsa-miR-1273h-5p	hsa-miR-4265	hsa-miR-541-3p	hsa-miR-6819-5p
hsa-miR-1275	hsa-miR-4265	hsa-miR-541-3p	hsa-miR-6819-5p
hsa-miR-1275	hsa-miR-4265	hsa-miR-541-3p	hsa-miR-6819-5p
hsa-miR-1275	hsa-miR-4265	hsa-miR-541-3p	hsa-miR-6819-5p
hsa-miR-127-3p	hsa-miR-4265	hsa-miR-541-3p	hsa-miR-6819-5p
hsa-miR-127-3p	hsa-miR-4265	hsa-miR-542-5p	hsa-miR-6820-5p
hsa-miR-127-5p	hsa-miR-4265	hsa-miR-544a	hsa-miR-6820-5p
hsa-miR-127-5p	hsa-miR-4267	hsa-miR-544b	hsa-miR-6820-5p
hsa-miR-127-5p	hsa-miR-4268	hsa-miR-544b	hsa-miR-6820-5p
hsa-miR-1281	hsa-miR-4271	hsa-miR-544b	hsa-miR-6820-5p
hsa-miR-1282	hsa-miR-4271	hsa-miR-544b	hsa-miR-6820-5p
hsa-miR-1285-3p	hsa-miR-4271	hsa-miR-548ai	hsa-miR-6820-5p
hsa-miR-1285-3p	hsa-miR-4273	hsa-miR-548an	hsa-miR-6820-5p
hsa-miR-1285-5p	hsa-miR-4277	hsa-miR-548ao-3p	hsa-miR-6820-5p
hsa-miR-1286	hsa-miR-4277	hsa-miR-548ao-3p	hsa-miR-6821-3p
hsa-miR-1287-5p	hsa-miR-4277	hsa-miR-548aq-5p	hsa-miR-6821-5p
hsa-miR-1287-5p	hsa-miR-4278	hsa-miR-548ar-5p	hsa-miR-6822-3p
hsa-miR-1287-5p	hsa-miR-4278	hsa-miR-548au-3p	hsa-miR-6822-3p
hsa-miR-1288-3p	hsa-miR-4279	hsa-miR-548ax	hsa-miR-6822-3p
hsa-miR-1289	hsa-miR-4279	hsa-miR-548bb-3p	hsa-miR-6822-3p
hsa-miR-128-1-5p	hsa-miR-4280	hsa-miR-548d-3p	hsa-miR-6822-5p
hsa-miR-128-1-5p	hsa-miR-4281	hsa-miR-548h-3p	hsa-miR-6823-5p
hsa-miR-128-1-5p	hsa-miR-4281	hsa-miR-548q	hsa-miR-6824-3p
hsa-miR-128-1-5p	hsa-miR-4281	hsa-miR-548q	hsa-miR-6824-3p
hsa-miR-128-2-5p	hsa-miR-4283	hsa-miR-548q	hsa-miR-6824-5p
hsa-miR-128-2-5p	hsa-miR-4283	hsa-miR-548q	hsa-miR-6824-5p
hsa-miR-128-2-5p	hsa-miR-4285	hsa-miR-548s	hsa-miR-6824-5p
hsa-miR-128-3p	hsa-miR-4287	hsa-miR-548u	hsa-miR-6824-5p
hsa-miR-128-3p	hsa-miR-4287	hsa-miR-548z	hsa-miR-6824-5p
hsa-miR-1290	hsa-miR-4289	hsa-miR-550a-3-5	hsa-miR-6825-3p
hsa-miR-1291	hsa-miR-4289	hsa-miR-550a-3p	hsa-miR-6825-5p
hsa-miR-1291	hsa-miR-4289	hsa-miR-550a-3p	hsa-miR-6825-5p
hsa-miR-1292-3p	hsa-miR-4289	hsa-miR-550a-5p	hsa-miR-6825-5p
hsa-miR-1292-3p	hsa-miR-4289	hsa-miR-550a-5p	hsa-miR-6825-5p
hsa-miR-1292-5p	hsa-miR-4290	hsa-miR-550a-5p	hsa-miR-6825-5p
hsa-miR-1292-5p	hsa-miR-4290	hsa-miR-550b-2-5	hsa-miR-6827-3p
hsa-miR-1292-5p	hsa-miR-4291	hsa-miR-552-3p	hsa-miR-6827-5p
hsa-miR-1293	hsa-miR-4292	hsa-miR-552-3p	hsa-miR-6827-5p
hsa-miR-1293	hsa-miR-4292	hsa-miR-552-3p	hsa-miR-6827-5p
hsa-miR-1293	hsa-miR-4292	hsa-miR-554 E	hsa-miR-6827-5p
hsa-miR-1293	hsa-miR-4293	hsa-miR-554 E	hsa-miR-6828-3p
hsa-miR-1293	hsa-miR-4297	hsa-miR-555 E	hsa-miR-6829-3p

hsa-miR-1293	hsa-miR-4298	hsa-miR-5571-3p	hsa-miR-6829-5p
hsa-miR-1293	hsa-miR-4298	hsa-miR-5571-3p	hsa-miR-6830-3p
hsa-miR-1294	hsa-miR-4298	hsa-miR-5571-3p	hsa-miR-6830-3p
hsa-miR-1294	hsa-miR-4298	hsa-miR-5571-3p	hsa-miR-6831-5p
hsa-miR-1294	hsa-miR-4298	hsa-miR-5571-5p	hsa-miR-6831-5p
hsa-miR-1294	hsa-miR-4299	hsa-miR-5572	hsa-miR-6831-5p
hsa-miR-1294	hsa-miR-4301	hsa-miR-5572	hsa-miR-6832-5p
hsa-miR-1296-3p	hsa-miR-4302	hsa-miR-5572	hsa-miR-6832-5p
hsa-miR-1296-5p	hsa-miR-4304	hsa-miR-5572	hsa-miR-6832-5p
hsa-miR-1296-5p	hsa-miR-4304	hsa-miR-5572	hsa-miR-6832-5p
hsa-miR-1296-5p	hsa-miR-4306	hsa-miR-5572	hsa-miR-6833-3p
hsa-miR-1296-5p	hsa-miR-4308	hsa-miR-5572	hsa-miR-6833-3p
hsa-miR-1299	hsa-miR-4312	hsa-miR-5572	hsa-miR-6833-5p
hsa-miR-1299	hsa-miR-4312	hsa-miR-5572	hsa-miR-6833-5p
hsa-miR-1299	hsa-miR-4312	hsa-miR-5572	hsa-miR-6833-5p
hsa-miR-129-5p	hsa-miR-4312	hsa-miR-557 E	hsa-miR-6834-5p
hsa-miR-129-5p	hsa-miR-4312	hsa-miR-557 E	hsa-miR-6834-5p
hsa-miR-129-5p	hsa-miR-4314	hsa-miR-557 E	hsa-miR-6834-5p
hsa-miR-129-5p	hsa-miR-4315	hsa-miR-557 E	hsa-miR-6834-5p
hsa-miR-1301-3p	hsa-miR-4316	hsa-miR-557 E	hsa-miR-6834-5p
hsa-miR-1302	hsa-miR-4316	hsa-miR-5580-5p	hsa-miR-6834-5p
hsa-miR-1302	hsa-miR-4316	hsa-miR-5581-3p	hsa-miR-6834-5p
hsa-miR-1303	hsa-miR-4316	hsa-miR-5584-3p	hsa-miR-6834-5p
hsa-miR-1304-3p	hsa-miR-4316	hsa-miR-5584-3p	hsa-miR-6835-5p
hsa-miR-1304-5p	hsa-miR-4318	hsa-miR-5584-5p	hsa-miR-6835-5p
hsa-miR-1304-5p	hsa-miR-4318	hsa-miR-5585-3p	hsa-miR-6835-5p
hsa-miR-1304-5p	hsa-miR-4318	hsa-miR-5586-3p	hsa-miR-6835-5p
hsa-miR-1304-5p	hsa-miR-431-3p	hsa-miR-5586-3p	hsa-miR-6835-5p
hsa-miR-1306-3p	hsa-miR-431-3p	hsa-miR-5587-3p	hsa-miR-6835-5p
hsa-miR-1306-3p	hsa-miR-431-3p	hsa-miR-5587-3p	hsa-miR-6835-5p
hsa-miR-1307-3p	hsa-miR-431-3p	hsa-miR-5587-5p	hsa-miR-6835-5p
hsa-miR-1307-3p	hsa-miR-431-5p	hsa-miR-5587-5p	hsa-miR-6835-5p
hsa-miR-1307-3p	hsa-miR-431-5p	hsa-miR-5589-3p	hsa-miR-6835-5p
hsa-miR-1307-3p	hsa-miR-431-5p	hsa-miR-5589-5p	hsa-miR-6836-3p
hsa-miR-1307-3p	hsa-miR-431-5p	hsa-miR-5589-5p	hsa-miR-6836-5p
hsa-miR-1307-3p	hsa-miR-431-5p	hsa-miR-5589-5p	hsa-miR-6836-5p
hsa-miR-1307-5p	hsa-miR-4320	hsa-miR-5589-5p	hsa-miR-6836-5p
hsa-miR-130a-5p	hsa-miR-4322	hsa-miR-5589-5p	hsa-miR-6837-3p
hsa-miR-130b-3p	hsa-miR-4322	hsa-miR-5589-5p	hsa-miR-6837-3p
hsa-miR-1321	hsa-miR-4322	hsa-miR-5589-5p	hsa-miR-6837-3p
hsa-miR-1322	hsa-miR-4322	hsa-miR-5589-5p	hsa-miR-6837-5p
hsa-miR-133a-3p	hsa-miR-4322	hsa-miR-558 E	hsa-miR-6838-3p
hsa-miR-133a-3p	hsa-miR-4322	hsa-miR-5591-5p	hsa-miR-6838-3p

hsa-miR-133b	hsa-miR-4322	hsa-miR-5591-5p	hsa-miR-6840-3p
hsa-miR-1343-3p	hsa-miR-4322	hsa-miR-5591-5p	hsa-miR-6840-3p
hsa-miR-1343-3p	hsa-miR-4322	hsa-miR-5591-5p	hsa-miR-6840-3p
hsa-miR-1343-3p	hsa-miR-4322	hsa-miR-5591-5p	hsa-miR-6840-5p
hsa-miR-1343-5p	hsa-miR-4324	hsa-miR-563 E	hsa-miR-6841-3p
hsa-miR-134-3p	hsa-miR-4325	hsa-miR-564 E	hsa-miR-6841-3p
hsa-miR-134-3p	hsa-miR-4326	hsa-miR-564 E	hsa-miR-6842-3p
hsa-miR-134-3p	hsa-miR-4326	hsa-miR-564 E	hsa-miR-6842-3p
hsa-miR-134-5p	hsa-miR-4326	hsa-miR-566 E	hsa-miR-6842-5p
hsa-miR-135a-3p	hsa-miR-4327	hsa-miR-566 E	hsa-miR-6842-5p
hsa-miR-135b-3p	hsa-miR-4327	hsa-miR-567 E	hsa-miR-6842-5p
hsa-miR-136-3p	hsa-miR-4327	hsa-miR-5691	hsa-miR-6842-5p
hsa-miR-136-5p	hsa-miR-432-3p	hsa-miR-5691	hsa-miR-6842-5p
hsa-miR-138-1-3p	hsa-miR-432-3p	hsa-miR-5691	hsa-miR-6843-3p
hsa-miR-138-1-3p	hsa-miR-432-5p	hsa-miR-5698	hsa-miR-6843-3p
hsa-miR-139-3p	hsa-miR-432-5p	hsa-miR-5698	hsa-miR-6845-5p
hsa-miR-139-3p	hsa-miR-432-5p	hsa-miR-5698	hsa-miR-6845-5p
hsa-miR-139-3p	hsa-miR-432-5p	hsa-miR-5698	hsa-miR-6846-3p
hsa-miR-140-3p	hsa-miR-432-5p	hsa-miR-5698	hsa-miR-6846-5p
hsa-miR-140-5p	hsa-miR-433-3p	hsa-miR-5698	hsa-miR-6846-5p
hsa-miR-140-5p	hsa-miR-4417	hsa-miR-5698	hsa-miR-6846-5p
hsa-miR-141-5p	hsa-miR-4417	hsa-miR-5698	hsa-miR-6846-5p
hsa-miR-142-3p	hsa-miR-4417	hsa-miR-5698	hsa-miR-6846-5p
hsa-miR-142-3p	hsa-miR-4418	hsa-miR-5698	hsa-miR-6846-5p
hsa-miR-142-3p	hsa-miR-4419a	hsa-miR-5698	hsa-miR-6846-5p
hsa-miR-142-3p	hsa-miR-4419b	hsa-miR-5699-3p	hsa-miR-6846-5p
hsa-miR-143-5p	hsa-miR-4419b	hsa-miR-5699-3p	hsa-miR-6846-5p
hsa-miR-143-5p	hsa-miR-4419b	hsa-miR-5699-3p	hsa-miR-6846-5p
hsa-miR-143-5p	hsa-miR-4420	hsa-miR-5699-3p	hsa-miR-6847-3p
hsa-miR-143-5p	hsa-miR-4420	hsa-miR-5699-3p	hsa-miR-6847-3p
hsa-miR-145-3p	hsa-miR-4420	hsa-miR-5699-5p	hsa-miR-6847-5p
hsa-miR-145-5p	hsa-miR-4421	hsa-miR-5702	hsa-miR-6847-5p
hsa-miR-145-5p	hsa-miR-4421	hsa-miR-5703	hsa-miR-6848-3p
hsa-miR-1468-5p	hsa-miR-4421	hsa-miR-5703	hsa-miR-6848-3p
hsa-miR-1469	hsa-miR-4421	hsa-miR-5705	hsa-miR-6848-3p
hsa-miR-1469	hsa-miR-4421	hsa-miR-5705	hsa-miR-6848-3p
hsa-miR-146a-3p	hsa-miR-4423-5p	hsa-miR-5705	hsa-miR-6848-5p
hsa-miR-146b-3p	hsa-miR-4423-5p	hsa-miR-5705	hsa-miR-6848-5p
hsa-miR-146b-3p	hsa-miR-4423-5p	hsa-miR-5705	hsa-miR-6848-5p
hsa-miR-146b-3p	hsa-miR-4424	hsa-miR-5706	hsa-miR-6848-5p
hsa-miR-146b-3p	hsa-miR-4425	hsa-miR-5707	hsa-miR-6848-5p
hsa-miR-146b-3p	hsa-miR-4425	hsa-miR-570-3p	hsa-miR-6848-5p
hsa-miR-146b-3p	hsa-miR-4425	hsa-miR-570-5p	hsa-miR-6848-5p

hsa-miR-146b-3p	hsa-miR-4425	hsa-miR-572 E	hsa-miR-6849-5p
hsa-miR-1470	hsa-miR-4425	hsa-miR-572 E	hsa-miR-6849-5p
hsa-miR-1471	hsa-miR-4425	hsa-miR-572 E	hsa-miR-6850-3p
hsa-miR-1471	hsa-miR-4430	hsa-miR-572 E	hsa-miR-6850-5p
hsa-miR-147a	hsa-miR-4432	hsa-miR-572 E	hsa-miR-6850-5p
hsa-miR-147a	hsa-miR-4433a-3p	hsa-miR-572 E	hsa-miR-6850-5p
hsa-miR-147b	hsa-miR-4433a-3p	hsa-miR-5739	hsa-miR-6850-5p
hsa-miR-147b	hsa-miR-4433b-3p	hsa-miR-5739	hsa-miR-6850-5p
hsa-miR-147b	hsa-miR-4433b-3p	hsa-miR-574-3p	hsa-miR-6851-5p
hsa-miR-148b-5p	hsa-miR-4433b-3p	hsa-miR-574-5p	hsa-miR-6851-5p
hsa-miR-149-3p	hsa-miR-4433b-5p	hsa-miR-574-5p	hsa-miR-6851-5p
hsa-miR-149-3p	hsa-miR-4435	hsa-miR-574-5p	hsa-miR-6851-5p
hsa-miR-149-3p	hsa-miR-4435	hsa-miR-574-5p	hsa-miR-6851-5p
hsa-miR-149-3p	hsa-miR-4436a	hsa-miR-575 E	hsa-miR-6852-3p
hsa-miR-149-3p	hsa-miR-4436a	hsa-miR-5787	hsa-miR-6852-3p
hsa-miR-149-3p	hsa-miR-4436b-3p	hsa-miR-5787	hsa-miR-6852-5p
hsa-miR-149-3p	hsa-miR-4436b-3p	hsa-miR-5787	hsa-miR-6852-5p
hsa-miR-149-5p	hsa-miR-4436b-3p	hsa-miR-578 E	hsa-miR-6852-5p
hsa-miR-149-5p	hsa-miR-4436b-3p	hsa-miR-578 E	hsa-miR-6852-5p
hsa-miR-149-5p	hsa-miR-4436b-3p	hsa-miR-578 E	hsa-miR-6852-5p
hsa-miR-149-5p	hsa-miR-4436b-3p	hsa-miR-578 E	hsa-miR-6852-5p
hsa-miR-150-3p	hsa-miR-4436b-5p	hsa-miR-578 E	hsa-miR-6852-5p
hsa-miR-150-3p	hsa-miR-4436b-5p	hsa-miR-578 E	hsa-miR-6853-3p
hsa-miR-150-3p	hsa-miR-4437	hsa-miR-579-3p	hsa-miR-6853-3p
hsa-miR-150-3p	hsa-miR-4437	hsa-miR-579-5p	hsa-miR-6853-5p
hsa-miR-150-3p	hsa-miR-4439	hsa-miR-579-5p	hsa-miR-6854-3p
hsa-miR-151a-3p	hsa-miR-4440	hsa-miR-579-5p	hsa-miR-6854-3p
hsa-miR-152-3p	hsa-miR-4440	hsa-miR-581 E	hsa-miR-6855-5p
hsa-miR-152-5p	hsa-miR-4440	hsa-miR-581 E	hsa-miR-6855-5p
hsa-miR-152-5p	hsa-miR-4440	hsa-miR-581 E	hsa-miR-6855-5p
hsa-miR-152-5p	hsa-miR-4440	hsa-miR-582-5p	hsa-miR-6855-5p
hsa-miR-1537-5p	hsa-miR-4440	hsa-miR-584-3p	hsa-miR-6855-5p
hsa-miR-1538	hsa-miR-4440	hsa-miR-584-3p	hsa-miR-6856-5p
hsa-miR-1538	hsa-miR-4440	hsa-miR-584-5p	hsa-miR-6856-5p
hsa-miR-1539	hsa-miR-4440	hsa-miR-584-5p	hsa-miR-6857-5p
hsa-miR-1539	hsa-miR-4440	hsa-miR-585-3p	hsa-miR-6857-5p
hsa-miR-1539	hsa-miR-4441	hsa-miR-585-3p	hsa-miR-6857-5p
hsa-miR-1539	hsa-miR-4441	hsa-miR-585-3p	hsa-miR-6858-5p
hsa-miR-1539	hsa-miR-4442	hsa-miR-587 E	hsa-miR-6858-5p
hsa-miR-153-3p	hsa-miR-4443	hsa-miR-588 E	hsa-miR-6859-3p
hsa-miR-155-3p	hsa-miR-4443	hsa-miR-588 E	hsa-miR-6859-5p
hsa-miR-155-5p	hsa-miR-4443	hsa-miR-588 E	hsa-miR-6860
hsa-miR-1587	hsa-miR-4443	hsa-miR-592 E	hsa-miR-6860

hsa-miR-1587	hsa-miR-4443	hsa-miR-592 E	hsa-miR-6860
hsa-miR-1587	hsa-miR-4445-5p	hsa-miR-593-3p	hsa-miR-6860
hsa-miR-1587	hsa-miR-4446-3p	hsa-miR-593-3p	hsa-miR-6860
hsa-miR-1587	hsa-miR-4446-3p	hsa-miR-593-3p	hsa-miR-6860
hsa-miR-15a-3p	hsa-miR-4446-3p	hsa-miR-593-3p	hsa-miR-6861-3p
hsa-miR-15a-5p	hsa-miR-4446-3p	hsa-miR-593-5p	hsa-miR-6861-5p
hsa-miR-16-1-3p	hsa-miR-4446-3p	hsa-miR-593-5p	hsa-miR-6861-5p
hsa-miR-16-5p	hsa-miR-4447	hsa-miR-593-5p	hsa-miR-6861-5p
hsa-miR-17-5p	hsa-miR-4447	hsa-miR-593-5p	hsa-miR-6861-5p
hsa-miR-181a-2-3	hsa-miR-4447	hsa-miR-593-5p	hsa-miR-6861-5p
hsa-miR-181d-3p	hsa-miR-4447	hsa-miR-593-5p	hsa-miR-6861-5p
hsa-miR-181d-3p	hsa-miR-4447	hsa-miR-596 E	hsa-miR-6861-5p
hsa-miR-1827	hsa-miR-4447	hsa-miR-596 E	hsa-miR-6861-5p
hsa-miR-182-3p	hsa-miR-4447	hsa-miR-596 E	hsa-miR-6862-3p
hsa-miR-182-5p	hsa-miR-4448	hsa-miR-597-3p	hsa-miR-6862-5p
hsa-miR-183-3p	hsa-miR-4448	hsa-miR-597-3p	hsa-miR-6862-5p
hsa-miR-183-5p	hsa-miR-4448	hsa-miR-597-3p	hsa-miR-6862-5p
hsa-miR-184 E	hsa-miR-4449	hsa-miR-597-3p	hsa-miR-6863
hsa-miR-184 E	hsa-miR-4449	hsa-miR-597-3p	hsa-miR-6864-5p
hsa-miR-185-3p	hsa-miR-4449	hsa-miR-597-5p	hsa-miR-6865-5p
hsa-miR-185-3p	hsa-miR-4449	hsa-miR-598-5p	hsa-miR-6865-5p
hsa-miR-185-3p	hsa-miR-4450	hsa-miR-598-5p	hsa-miR-6866-5p
hsa-miR-185-3p	hsa-miR-4450	hsa-miR-598-5p	hsa-miR-6867-3p
hsa-miR-185-3p	hsa-miR-4450	hsa-miR-600 E	hsa-miR-6867-5p
hsa-miR-185-5p	hsa-miR-4451	hsa-miR-601 E	hsa-miR-6867-5p
hsa-miR-185-5p	hsa-miR-4452	hsa-miR-601 E	hsa-miR-6868-3p
hsa-miR-187-3p	hsa-miR-4453	hsa-miR-601 E	hsa-miR-6868-3p
hsa-miR-187-3p	hsa-miR-4455	hsa-miR-601 E	hsa-miR-6868-3p
hsa-miR-187-3p	hsa-miR-4455	hsa-miR-602 E	hsa-miR-6868-3p
hsa-miR-187-3p	hsa-miR-4456	hsa-miR-602 E	hsa-miR-6869-5p
hsa-miR-187-3p	hsa-miR-4458	hsa-miR-604 E	hsa-miR-6869-5p
hsa-miR-187-3p	hsa-miR-4459	hsa-miR-604 E	hsa-miR-6869-5p
hsa-miR-187-3p	hsa-miR-4459	hsa-miR-6068	hsa-miR-6870-5p
hsa-miR-188-3p	hsa-miR-4461	hsa-miR-6068	hsa-miR-6870-5p
hsa-miR-188-5p	hsa-miR-4462	hsa-miR-6069	hsa-miR-6870-5p
hsa-miR-188-5p	hsa-miR-4462	hsa-miR-6069	hsa-miR-6870-5p
hsa-miR-188-5p	hsa-miR-4464	hsa-miR-6069	hsa-miR-6870-5p
hsa-miR-18a-3p	hsa-miR-4465	hsa-miR-6069	hsa-miR-6870-5p
hsa-miR-18a-5p	hsa-miR-4465	hsa-miR-6069	hsa-miR-6870-5p
hsa-miR-18b-5p	hsa-miR-4466	hsa-miR-6070	hsa-miR-6870-5p
hsa-miR-1908-3p	hsa-miR-4466	hsa-miR-6070	hsa-miR-6870-5p
hsa-miR-1908-5p	hsa-miR-4466	hsa-miR-6071	hsa-miR-6870-5p
hsa-miR-1908-5p	hsa-miR-4466	hsa-miR-6071	hsa-miR-6870-5p

hsa-miR-1908-5p	hsa-miR-4466	hsa-miR-6071	hsa-miR-6871-5p
hsa-miR-1908-5p	hsa-miR-4466	hsa-miR-6073	hsa-miR-6871-5p
hsa-miR-1908-5p	hsa-miR-4466	hsa-miR-6074	hsa-miR-6872-3p
hsa-miR-1908-5p	hsa-miR-4467	hsa-miR-6074	hsa-miR-6873-3p
hsa-miR-1908-5p	hsa-miR-4467	hsa-miR-6075	hsa-miR-6874-3p
hsa-miR-1909-3p	hsa-miR-4467	hsa-miR-6076	hsa-miR-6874-3p
hsa-miR-1909-3p	hsa-miR-4467	hsa-miR-6078	hsa-miR-6874-3p
hsa-miR-1909-3p	hsa-miR-4467	hsa-miR-6081	hsa-miR-6874-3p
hsa-miR-1909-3p	hsa-miR-4467	hsa-miR-6081	hsa-miR-6874-3p
hsa-miR-1909-3p	hsa-miR-4467	hsa-miR-6081	hsa-miR-6874-5p
hsa-miR-1909-5p	hsa-miR-4467	hsa-miR-6081	hsa-miR-6874-5p
hsa-miR-1910-3p	hsa-miR-4467	hsa-miR-6081	hsa-miR-6874-5p
hsa-miR-1910-3p	hsa-miR-4467	hsa-miR-6081	hsa-miR-6874-5p
hsa-miR-1910-5p	hsa-miR-4467	hsa-miR-6081	hsa-miR-6875-3p
hsa-miR-1911-3p	hsa-miR-4468	hsa-miR-6084	hsa-miR-6875-5p
hsa-miR-1911-3p	hsa-miR-4468	hsa-miR-6084	hsa-miR-6875-5p
hsa-miR-1913	hsa-miR-4469	hsa-miR-6084	hsa-miR-6875-5p
hsa-miR-1914-3p	hsa-miR-4470	hsa-miR-6085	hsa-miR-6876-3p
hsa-miR-1914-3p	hsa-miR-4471	hsa-miR-6085	hsa-miR-6876-3p
hsa-miR-1914-5p	hsa-miR-4472	hsa-miR-6086	hsa-miR-6876-3p
hsa-miR-1914-5p	hsa-miR-4472	hsa-miR-6086	hsa-miR-6876-3p
hsa-miR-1915-3p	hsa-miR-4472	hsa-miR-6086	hsa-miR-6876-3p
hsa-miR-1915-3p	hsa-miR-4472	hsa-miR-6086	hsa-miR-6876-3p
hsa-miR-1915-3p	hsa-miR-4472	hsa-miR-6086	hsa-miR-6876-5p
hsa-miR-1915-3p	hsa-miR-4472	hsa-miR-6086	hsa-miR-6877-3p
hsa-miR-1915-5p	hsa-miR-4472	hsa-miR-6086	hsa-miR-6877-5p
hsa-miR-1915-5p	hsa-miR-4474-3p	hsa-miR-6087	hsa-miR-6877-5p
hsa-miR-1915-5p	hsa-miR-4474-3p	hsa-miR-6087	hsa-miR-6877-5p
hsa-miR-191-3p	hsa-miR-4476	hsa-miR-6087	hsa-miR-6877-5p
hsa-miR-193a-5p	hsa-miR-4476	hsa-miR-6087	hsa-miR-6878-3p
hsa-miR-193a-5p	hsa-miR-4478	hsa-miR-6087	hsa-miR-6878-3p
hsa-miR-193a-5p	hsa-miR-4478	hsa-miR-6087	hsa-miR-6878-5p
hsa-miR-193a-5p	hsa-miR-4479	hsa-miR-6088	hsa-miR-6879-3p
hsa-miR-193a-5p	hsa-miR-4479	hsa-miR-6089	hsa-miR-6879-3p
hsa-miR-193a-5p	hsa-miR-4479	hsa-miR-6089	hsa-miR-6879-5p
hsa-miR-193b-5p	hsa-miR-4481	hsa-miR-6089	hsa-miR-6879-5p
hsa-miR-193b-5p	hsa-miR-4481	hsa-miR-6089	hsa-miR-6879-5p
hsa-miR-193b-5p	hsa-miR-4481	hsa-miR-608 E	hsa-miR-6879-5p
hsa-miR-193b-5p	hsa-miR-4482-3p	hsa-miR-608 E	hsa-miR-6880-5p
hsa-miR-193b-5p	hsa-miR-4482-3p	hsa-miR-608 E	hsa-miR-6880-5p
hsa-miR-194-3p	hsa-miR-4482-5p	hsa-miR-608 E	hsa-miR-6880-5p
hsa-miR-194-3p	hsa-miR-4483	hsa-miR-608 E	hsa-miR-6880-5p
hsa-miR-194-3p	hsa-miR-4483	hsa-miR-608 E	hsa-miR-6880-5p

hsa-miR-195-5p	hsa-miR-4483	hsa-miR-608 E	hsa-miR-6881-3p
hsa-miR-196a-5p	hsa-miR-4483	hsa-miR-608 E	hsa-miR-6881-5p
hsa-miR-196a-5p	hsa-miR-4483	hsa-miR-608 E	hsa-miR-6881-5p
hsa-miR-196a-5p	hsa-miR-4483	hsa-miR-6090	hsa-miR-6881-5p
hsa-miR-196b-3p	hsa-miR-4483	hsa-miR-6090	hsa-miR-6881-5p
hsa-miR-196b-5p	hsa-miR-4483	hsa-miR-6090	hsa-miR-6881-5p
hsa-miR-1972	hsa-miR-4485-5p	hsa-miR-609 E	hsa-miR-6881-5p
hsa-miR-1973	hsa-miR-4486	hsa-miR-611 E	hsa-miR-6881-5p
hsa-miR-1976	hsa-miR-4486	hsa-miR-611 E	hsa-miR-6882-3p
hsa-miR-197-5p	hsa-miR-4486	hsa-miR-6126	hsa-miR-6883-3p
hsa-miR-197-5p	hsa-miR-4486	hsa-miR-6127	hsa-miR-6883-3p
hsa-miR-197-5p	hsa-miR-4486	hsa-miR-6127	hsa-miR-6883-5p
hsa-miR-197-5p	hsa-miR-4486	hsa-miR-6129	hsa-miR-6883-5p
hsa-miR-198 E	hsa-miR-4487	hsa-miR-6129	hsa-miR-6883-5p
hsa-miR-199a-3p	hsa-miR-4487	hsa-miR-6129	hsa-miR-6883-5p
hsa-miR-199a-3p	hsa-miR-4488	hsa-miR-612 E	hsa-miR-6883-5p
hsa-miR-199b-3p	hsa-miR-4488	hsa-miR-612 E	hsa-miR-6883-5p
hsa-miR-199b-3p	hsa-miR-4488	hsa-miR-612 E	hsa-miR-6884-5p
hsa-miR-19a-5p	hsa-miR-4488	hsa-miR-612 E	hsa-miR-6884-5p
hsa-miR-19b-1-5p	hsa-miR-4488	hsa-miR-6130	hsa-miR-6885-3p
hsa-miR-19b-1-5p	hsa-miR-4488	hsa-miR-6130	hsa-miR-6885-3p
hsa-miR-19b-1-5p	hsa-miR-4489	hsa-miR-6130	hsa-miR-6885-3p
hsa-miR-19b-1-5p	hsa-miR-4489	hsa-miR-6131	hsa-miR-6885-5p
hsa-miR-19b-2-5p	hsa-miR-4489	hsa-miR-6131	hsa-miR-6885-5p
hsa-miR-19b-2-5p	hsa-miR-4489	hsa-miR-6131	hsa-miR-6885-5p
hsa-miR-19b-2-5p	hsa-miR-448 E	hsa-miR-6131	hsa-miR-6886-5p
hsa-miR-19b-2-5p	hsa-miR-4490	hsa-miR-6131	hsa-miR-6886-5p
hsa-miR-19b-3p	hsa-miR-4490	hsa-miR-6132	hsa-miR-6887-5p
hsa-miR-1-5p	hsa-miR-4490	hsa-miR-6132	hsa-miR-6887-5p
hsa-miR-200b-5p	hsa-miR-4492	hsa-miR-6132	hsa-miR-6887-5p
hsa-miR-200c-5p	hsa-miR-4492	hsa-miR-6132	hsa-miR-6887-5p
hsa-miR-200c-5p	hsa-miR-4494	hsa-miR-6133	hsa-miR-6887-5p
hsa-miR-202-3p	hsa-miR-4494	hsa-miR-6133	hsa-miR-6888-3p
hsa-miR-202-5p	hsa-miR-4494	hsa-miR-6134	hsa-miR-6888-3p
hsa-miR-202-5p	hsa-miR-4496	hsa-miR-6134	hsa-miR-6888-3p
hsa-miR-203a-3p	hsa-miR-4497	hsa-miR-6134	hsa-miR-6888-5p
hsa-miR-204-3p	hsa-miR-4497	hsa-miR-6134	hsa-miR-6889-5p
hsa-miR-204-3p	hsa-miR-4497	hsa-miR-615-3p	hsa-miR-6889-5p
hsa-miR-204-5p	hsa-miR-4498	hsa-miR-615-5p	hsa-miR-6889-5p
hsa-miR-205-3p	hsa-miR-4498	hsa-miR-615-5p	hsa-miR-6890-3p
hsa-miR-205-5p	hsa-miR-4498	hsa-miR-615-5p	hsa-miR-6890-5p
hsa-miR-20a-5p	hsa-miR-4498	hsa-miR-6165	hsa-miR-6890-5p
hsa-miR-20b-3p	hsa-miR-4498	hsa-miR-6165	hsa-miR-6891-5p

hsa-miR-210-3p	hsa-miR-4498	hsa-miR-6165	hsa-miR-6892-3p
hsa-miR-210-3p	hsa-miR-4498	hsa-miR-616-3p	hsa-miR-6892-3p
hsa-miR-210-5p	hsa-miR-4498	hsa-miR-616-3p	hsa-miR-6893-5p
hsa-miR-2110	hsa-miR-449a	hsa-miR-619-3p	hsa-miR-6893-5p
hsa-miR-2110	hsa-miR-449a	hsa-miR-619-5p	hsa-miR-6893-5p
hsa-miR-2110	hsa-miR-449a	hsa-miR-619-5p	hsa-miR-6894-3p
hsa-miR-2113	hsa-miR-449a	hsa-miR-619-5p	hsa-miR-6894-5p
hsa-miR-2113	hsa-miR-449a	hsa-miR-619-5p	hsa-miR-6894-5p
hsa-miR-2114-3p	hsa-miR-449a	hsa-miR-622 E	hsa-miR-6895-3p
hsa-miR-2116-5p	hsa-miR-449a	hsa-miR-622 E	hsa-miR-6895-3p
hsa-miR-2116-5p	hsa-miR-449b-5p	hsa-miR-623 E	hsa-miR-708-5p
hsa-miR-2116-5p	hsa-miR-449b-5p	hsa-miR-623 E	hsa-miR-7106-5p
hsa-miR-2116-5p	hsa-miR-449b-5p	hsa-miR-623 E	hsa-miR-7106-5p
hsa-miR-2116-5p	hsa-miR-449b-5p	hsa-miR-623 E	hsa-miR-7106-5p
hsa-miR-2117	hsa-miR-449b-5p	hsa-miR-624-3p	hsa-miR-7106-5p
hsa-miR-2117	hsa-miR-449b-5p	hsa-miR-625-5p	hsa-miR-7106-5p
hsa-miR-212-5p	hsa-miR-449b-5p	hsa-miR-626 E	hsa-miR-7106-5p
hsa-miR-212-5p	hsa-miR-449b-5p	hsa-miR-627-3p	hsa-miR-7107-3p
hsa-miR-212-5p	hsa-miR-449b-5p	hsa-miR-627-3p	hsa-miR-7107-3p
hsa-miR-214-5p	hsa-miR-449c-3p	hsa-miR-627-5p	hsa-miR-7107-3p
hsa-miR-214-5p	hsa-miR-449c-3p	hsa-miR-629-3p	hsa-miR-7107-3p
hsa-miR-214-5p	hsa-miR-449c-3p	hsa-miR-629-5p	hsa-miR-7107-5p
hsa-miR-218-1-3p	hsa-miR-449c-3p	hsa-miR-629-5p	hsa-miR-7107-5p
hsa-miR-218-1-3p	hsa-miR-449c-5p	hsa-miR-629-5p	hsa-miR-7107-5p
hsa-miR-218-1-3p	hsa-miR-449c-5p	hsa-miR-630 E	hsa-miR-7107-5p
hsa-miR-218-1-3p	hsa-miR-449c-5p	hsa-miR-632 E	hsa-miR-7108-5p
hsa-miR-218-2-3p	hsa-miR-449c-5p	hsa-miR-632 E	hsa-miR-7108-5p
hsa-miR-218-2-3p	hsa-miR-449c-5p	hsa-miR-632 E	hsa-miR-7108-5p
hsa-miR-218-2-3p	hsa-miR-4500	hsa-miR-632 E	hsa-miR-7109-5p
hsa-miR-219a-1-3	hsa-miR-4500	hsa-miR-636 E	hsa-miR-7109-5p
hsa-miR-219b-5p	hsa-miR-4502	hsa-miR-636 E	hsa-miR-7109-5p
hsa-miR-221-3p	hsa-miR-4502	hsa-miR-636 E	hsa-miR-7109-5p
hsa-miR-221-5p	hsa-miR-4502	hsa-miR-637 E	hsa-miR-7109-5p
hsa-miR-223-3p	hsa-miR-4502	hsa-miR-637 E	hsa-miR-7109-5p
hsa-miR-2277-3p	hsa-miR-4505	hsa-miR-637 E	hsa-miR-7109-5p
hsa-miR-2277-5p	hsa-miR-4505	hsa-miR-637 E	hsa-miR-7110-3p
hsa-miR-2277-5p	hsa-miR-4505	hsa-miR-637 E	hsa-miR-7110-3p
hsa-miR-2277-5p	hsa-miR-4505	hsa-miR-637 E	hsa-miR-7110-5p
hsa-miR-2277-5p	hsa-miR-4505	hsa-miR-638 E	hsa-miR-7110-5p
hsa-miR-2277-5p	hsa-miR-4506	hsa-miR-638 E	hsa-miR-7110-5p
hsa-miR-2278	hsa-miR-4507	hsa-miR-638 E	hsa-miR-7110-5p
hsa-miR-2278	hsa-miR-4507	hsa-miR-638 E	hsa-miR-7110-5p
hsa-miR-2278	hsa-miR-4507	hsa-miR-639 E	hsa-miR-7110-5p

hsa-miR-2278	hsa-miR-4507	hsa-miR-639 E	hsa-miR-7110-5p
hsa-miR-2278	hsa-miR-4507	hsa-miR-643 E	hsa-miR-7110-5p
hsa-miR-2355-3p	hsa-miR-4507	hsa-miR-645 E	hsa-miR-7110-5p
hsa-miR-2355-3p	hsa-miR-4507	hsa-miR-645 E	hsa-miR-7110-5p
hsa-miR-2355-3p	hsa-miR-4507	hsa-miR-645 E	hsa-miR-7110-5p
hsa-miR-2355-3p	hsa-miR-4507	hsa-miR-647 E	hsa-miR-7111-5p
hsa-miR-2355-3p	hsa-miR-4507	hsa-miR-647 E	hsa-miR-7111-5p
hsa-miR-2392	hsa-miR-4508	hsa-miR-647 E	hsa-miR-7111-5p
hsa-miR-2392	hsa-miR-4508	hsa-miR-648 E	hsa-miR-7111-5p
hsa-miR-23a-5p	hsa-miR-4508	hsa-miR-648 E	hsa-miR-7112-3p
hsa-miR-23a-5p	hsa-miR-4508	hsa-miR-648 E	hsa-miR-7112-3p
hsa-miR-23a-5p	hsa-miR-450b-3p	hsa-miR-6499-3p	hsa-miR-7112-5p
hsa-miR-23b-5p	hsa-miR-450b-3p	hsa-miR-6499-3p	hsa-miR-7113-3p
hsa-miR-23b-5p	hsa-miR-4510	hsa-miR-6499-5p	hsa-miR-7113-5p
hsa-miR-2467-3p	hsa-miR-4510	hsa-miR-6499-5p	hsa-miR-7114-5p
hsa-miR-2467-3p	hsa-miR-4510	hsa-miR-6499-5p	hsa-miR-7114-5p
hsa-miR-2467-3p	hsa-miR-4510	hsa-miR-6500-3p	hsa-miR-7114-5p
hsa-miR-2467-5p	hsa-miR-4514	hsa-miR-6500-3p	hsa-miR-7114-5p
hsa-miR-2467-5p	hsa-miR-4515	hsa-miR-6500-3p	hsa-miR-7114-5p
hsa-miR-2467-5p	hsa-miR-4518	hsa-miR-6500-3p	hsa-miR-7114-5p
hsa-miR-2467-5p	hsa-miR-4518	hsa-miR-6500-5p	hsa-miR-711 E
hsa-miR-24-1-5p	hsa-miR-4518	hsa-miR-6501-3p	hsa-miR-711 E
hsa-miR-24-3p	hsa-miR-4519	hsa-miR-6501-3p	hsa-miR-711 E
hsa-miR-25-5p	hsa-miR-4520-3p	hsa-miR-6501-5p	hsa-miR-7150
hsa-miR-25-5p	hsa-miR-4520-5p	hsa-miR-6501-5p	hsa-miR-7150
hsa-miR-25-5p	hsa-miR-4520-5p	hsa-miR-6501-5p	hsa-miR-7150
hsa-miR-25-5p	hsa-miR-4520-5p	hsa-miR-6503-5p	hsa-miR-7151-3p
hsa-miR-25-5p	hsa-miR-4521	hsa-miR-6504-5p	hsa-miR-7151-3p
hsa-miR-25-5p	hsa-miR-4521	hsa-miR-6504-5p	hsa-miR-7151-3p
hsa-miR-25-5p	hsa-miR-4524a-3p	hsa-miR-6504-5p	hsa-miR-7152-3p
hsa-miR-25-5p	hsa-miR-4524a-3p	hsa-miR-6505-3p	hsa-miR-7152-3p
hsa-miR-2682-5p	hsa-miR-4524b-3p	hsa-miR-6506-5p	hsa-miR-7152-3p
hsa-miR-2682-5p	hsa-miR-4525	hsa-miR-6508-5p	hsa-miR-7152-3p
hsa-miR-2682-5p	hsa-miR-4525	hsa-miR-650 E	hsa-miR-7152-5p
hsa-miR-2682-5p	hsa-miR-4525	hsa-miR-650 E	hsa-miR-7152-5p
hsa-miR-26a-5p	hsa-miR-4525	hsa-miR-650 E	hsa-miR-7152-5p
hsa-miR-26b-3p	hsa-miR-4525	hsa-miR-650 E	hsa-miR-7152-5p
hsa-miR-26b-3p	hsa-miR-4525	hsa-miR-6510-3p	hsa-miR-7154-3p
hsa-miR-26b-3p	hsa-miR-4525	hsa-miR-6510-5p	hsa-miR-7154-3p
hsa-miR-27a-5p	hsa-miR-4529-3p	hsa-miR-6511a-5p	hsa-miR-7154-3p
hsa-miR-2861	hsa-miR-4530	hsa-miR-6511a-5p	hsa-miR-7154-5p
hsa-miR-2861	hsa-miR-4530	hsa-miR-6513-5p	hsa-miR-7154-5p
hsa-miR-2861	hsa-miR-4532	hsa-miR-6513-5p	hsa-miR-7155-3p

hsa-miR-2861	hsa-miR-4532	hsa-miR-6515-3p	hsa-miR-7155-3p
hsa-miR-2861	hsa-miR-4534	hsa-miR-6515-5p	hsa-miR-7155-5p
hsa-miR-28-3p	hsa-miR-4534	hsa-miR-6515-5p	hsa-miR-7155-5p
hsa-miR-296-3p	hsa-miR-4534	hsa-miR-6515-5p	hsa-miR-7155-5p
hsa-miR-296-3p	hsa-miR-4534	hsa-miR-6516-3p	hsa-miR-7155-5p
hsa-miR-296-3p	hsa-miR-4535	hsa-miR-653-3p	hsa-miR-7155-5p
hsa-miR-296-3p	hsa-miR-4535	hsa-miR-653-3p	hsa-miR-7155-5p
hsa-miR-296-3p	hsa-miR-4536-5p	hsa-miR-654-5p	hsa-miR-7156-3p
hsa-miR-296-3p	hsa-miR-4537	hsa-miR-654-5p	hsa-miR-7157-3p
hsa-miR-296-3p	hsa-miR-4538	hsa-miR-654-5p	hsa-miR-7157-3p
hsa-miR-296-3p	hsa-miR-4538	hsa-miR-654-5p	hsa-miR-7157-5p
hsa-miR-297 E	hsa-miR-4538	hsa-miR-654-5p	hsa-miR-7157-5p
hsa-miR-297 E	hsa-miR-4538	hsa-miR-654-5p	hsa-miR-7157-5p
hsa-miR-298 E	hsa-miR-455-3p	hsa-miR-654-5p	hsa-miR-7160-3p
hsa-miR-298 E	hsa-miR-455-3p	hsa-miR-656-5p	hsa-miR-7160-3p
hsa-miR-299-3p	hsa-miR-455-5p	hsa-miR-656-5p	hsa-miR-7160-3p
hsa-miR-299-5p	hsa-miR-4632-3p	hsa-miR-656-5p	hsa-miR-7160-5p
hsa-miR-29a-5p	hsa-miR-4632-5p	hsa-miR-656-5p	hsa-miR-7160-5p
hsa-miR-29b-1-5p	hsa-miR-4632-5p	hsa-miR-656-5p	hsa-miR-7161-3p
hsa-miR-29b-1-5p	hsa-miR-4632-5p	hsa-miR-657 E	hsa-miR-7161-5p
hsa-miR-29b-1-5p	hsa-miR-4632-5p	hsa-miR-657 E	hsa-miR-7162-3p
hsa-miR-29b-1-5p	hsa-miR-4632-5p	hsa-miR-658 E	hsa-miR-7162-5p
hsa-miR-29b-1-5p	hsa-miR-4632-5p	hsa-miR-658 E	hsa-miR-7162-5p
hsa-miR-29b-2-5p	hsa-miR-4633-3p	hsa-miR-658 E	hsa-miR-718 E
hsa-miR-29b-2-5p	hsa-miR-4634	hsa-miR-658 E	hsa-miR-744-3p
hsa-miR-29c-5p	hsa-miR-4634	hsa-miR-658 E	hsa-miR-744-3p
hsa-miR-301b-5p	hsa-miR-4634	hsa-miR-658 E	hsa-miR-744-5p
hsa-miR-301b-5p	hsa-miR-4635	hsa-miR-658 E	hsa-miR-744-5p
hsa-miR-301b-5p	hsa-miR-4638-3p	hsa-miR-658 E	hsa-miR-744-5p
hsa-miR-3064-5p	hsa-miR-4638-3p	hsa-miR-658 E	hsa-miR-744-5p
hsa-miR-3064-5p	hsa-miR-4638-5p	hsa-miR-658 E	hsa-miR-7515
hsa-miR-3064-5p	hsa-miR-4640-5p	hsa-miR-658 E	hsa-miR-758-3p
hsa-miR-3064-5p	hsa-miR-4640-5p	hsa-miR-659-3p	hsa-miR-758-3p
hsa-miR-3064-5p	hsa-miR-4640-5p	hsa-miR-659-3p	hsa-miR-758-3p
hsa-miR-3064-5p	hsa-miR-4641	hsa-miR-659-3p	hsa-miR-758-5p
hsa-miR-3074-5p	hsa-miR-4642	hsa-miR-659-5p	hsa-miR-760 E
hsa-miR-3074-5p	hsa-miR-4642	hsa-miR-660-3p	hsa-miR-760 E
hsa-miR-3074-5p	hsa-miR-4642	hsa-miR-661 E	hsa-miR-760 E
hsa-miR-3074-5p	hsa-miR-4646-3p	hsa-miR-661 E	hsa-miR-760 E
hsa-miR-3074-5p	hsa-miR-4646-3p	hsa-miR-661 E	hsa-miR-760 E
hsa-miR-30b-3p	hsa-miR-4646-5p	hsa-miR-662 E	hsa-miR-760 E
hsa-miR-30b-3p	hsa-miR-4647	hsa-miR-663a	hsa-miR-760 E
hsa-miR-30b-3p	hsa-miR-4648	hsa-miR-663a	hsa-miR-761 E

hsa-miR-30b-3p	hsa-miR-4648	hsa-miR-663a	hsa-miR-761 E
hsa-miR-30b-3p	hsa-miR-4648	hsa-miR-663a	hsa-miR-761 E
hsa-miR-30b-3p	hsa-miR-4649-3p	hsa-miR-663a	hsa-miR-762 E
hsa-miR-30b-3p	hsa-miR-4649-3p	hsa-miR-663a	hsa-miR-762 E
hsa-miR-30c-1-3p	hsa-miR-4649-5p	hsa-miR-663b	hsa-miR-762 E
hsa-miR-30c-1-3p	hsa-miR-4649-5p	hsa-miR-663b	hsa-miR-762 E
hsa-miR-30c-1-3p	hsa-miR-4649-5p	hsa-miR-663b	hsa-miR-762 E
hsa-miR-30c-1-3p	hsa-miR-4649-5p	hsa-miR-663b	hsa-miR-762 E
hsa-miR-30c-1-3p	hsa-miR-4649-5p	hsa-miR-664a-5p	hsa-miR-762 E
hsa-miR-30c-2-3p	hsa-miR-4649-5p	hsa-miR-664b-3p	hsa-miR-762 E
hsa-miR-30c-2-3p	hsa-miR-4649-5p	hsa-miR-664b-3p	hsa-miR-764 E
hsa-miR-30c-2-3p	hsa-miR-4649-5p	hsa-miR-664b-5p	hsa-miR-764 E
hsa-miR-30c-2-3p	hsa-miR-4649-5p	hsa-miR-668-3p	hsa-miR-764 E
hsa-miR-30d-3p	hsa-miR-4649-5p	hsa-miR-668-5p	hsa-miR-764 E
hsa-miR-30d-3p	hsa-miR-4651	hsa-miR-668-5p	hsa-miR-764 E
hsa-miR-3116	hsa-miR-4651	hsa-miR-668-5p	hsa-miR-764 E
hsa-miR-3116	hsa-miR-4651	hsa-miR-670-5p	hsa-miR-765 E
hsa-miR-3116	hsa-miR-4651	hsa-miR-670-5p	hsa-miR-765 E
hsa-miR-3117-3p	hsa-miR-4651	hsa-miR-670-5p	hsa-miR-766-5p
hsa-miR-3120-5p	hsa-miR-4651	hsa-miR-6715a-3p	hsa-miR-766-5p
hsa-miR-3120-5p	hsa-miR-4651	hsa-miR-6715b-5p	hsa-miR-767-3p
hsa-miR-3120-5p	hsa-miR-4651	hsa-miR-6716-5p	hsa-miR-767-5p
hsa-miR-3121-3p	hsa-miR-4651	hsa-miR-6716-5p	hsa-miR-767-5p
hsa-miR-3121-5p	hsa-miR-4651	hsa-miR-6717-5p	hsa-miR-767-5p
hsa-miR-3122	hsa-miR-4652-3p	hsa-miR-6717-5p	hsa-miR-767-5p
hsa-miR-3122	hsa-miR-4652-3p	hsa-miR-6717-5p	hsa-miR-769-3p
hsa-miR-3122	hsa-miR-4652-3p	hsa-miR-6717-5p	hsa-miR-769-3p
hsa-miR-3122	hsa-miR-4653-3p	hsa-miR-6717-5p	hsa-miR-7703
hsa-miR-3124-3p	hsa-miR-4653-3p	hsa-miR-6717-5p	hsa-miR-7703
hsa-miR-3124-5p	hsa-miR-4653-3p	hsa-miR-6718-5p	hsa-miR-7703
hsa-miR-3127-3p	hsa-miR-4653-3p	hsa-miR-6718-5p	hsa-miR-7704
hsa-miR-3127-3p	hsa-miR-4653-5p	hsa-miR-671-3p	hsa-miR-7704
hsa-miR-3127-5p	hsa-miR-4653-5p	hsa-miR-671-5p	hsa-miR-7704
hsa-miR-3128	hsa-miR-4653-5p	hsa-miR-6720-3p	hsa-miR-7704
hsa-miR-3129-5p	hsa-miR-4653-5p	hsa-miR-6720-3p	hsa-miR-7704
hsa-miR-3129-5p	hsa-miR-4654	hsa-miR-6720-5p	hsa-miR-7704
hsa-miR-3129-5p	hsa-miR-4654	hsa-miR-6721-5p	hsa-miR-7704
hsa-miR-3130-3p	hsa-miR-4654	hsa-miR-6721-5p	hsa-miR-7706
hsa-miR-3130-3p	hsa-miR-4654	hsa-miR-6721-5p	hsa-miR-7706
hsa-miR-3131	hsa-miR-4655-3p	hsa-miR-6721-5p	hsa-miR-770-5p
hsa-miR-3131	hsa-miR-4655-5p	hsa-miR-6721-5p	hsa-miR-770-5p
hsa-miR-3132	hsa-miR-4656	hsa-miR-6721-5p	hsa-miR-770-5p
hsa-miR-3132	hsa-miR-4656	hsa-miR-6721-5p	hsa-miR-7843-5p

hsa-miR-3132	hsa-miR-4656	hsa-miR-6722-3p	hsa-miR-7843-5p
hsa-miR-3132	hsa-miR-4657	hsa-miR-6722-3p	hsa-miR-7843-5p
hsa-miR-3135a	hsa-miR-4658	hsa-miR-6722-3p	hsa-miR-7843-5p
hsa-miR-3135a	hsa-miR-4658	hsa-miR-6722-3p	hsa-miR-7843-5p
hsa-miR-3135a	hsa-miR-4658	hsa-miR-6722-3p	hsa-miR-7845-5p
hsa-miR-3135a	hsa-miR-4658	hsa-miR-6722-3p	hsa-miR-7845-5p
hsa-miR-3135a	hsa-miR-4659a-3p	hsa-miR-6722-3p	hsa-miR-7846-3p
hsa-miR-3135b	hsa-miR-4659b-3p	hsa-miR-6722-5p	hsa-miR-7846-3p
hsa-miR-3135b	hsa-miR-4660	hsa-miR-6723-5p	hsa-miR-7847-3p
hsa-miR-3135b	hsa-miR-4660	hsa-miR-6724-5p	hsa-miR-7847-3p
hsa-miR-3135b	hsa-miR-4660	hsa-miR-6724-5p	hsa-miR-7847-3p
hsa-miR-3135b	hsa-miR-4661-3p	hsa-miR-6724-5p	hsa-miR-7847-3p
hsa-miR-3135b	hsa-miR-4664-5p	hsa-miR-6724-5p	hsa-miR-7847-3p
hsa-miR-3136-5p	hsa-miR-4664-5p	hsa-miR-6724-5p	hsa-miR-7847-3p
hsa-miR-3137	hsa-miR-4664-5p	hsa-miR-6726-3p	hsa-miR-7849-3p
hsa-miR-3137	hsa-miR-4664-5p	hsa-miR-6726-5p	hsa-miR-7850-5p
hsa-miR-3137	hsa-miR-4664-5p	hsa-miR-6726-5p	hsa-miR-7850-5p
hsa-miR-3137	hsa-miR-4664-5p	hsa-miR-6726-5p	hsa-miR-7850-5p
hsa-miR-3137	hsa-miR-4665-5p	hsa-miR-6727-3p	hsa-miR-7850-5p
hsa-miR-3138	hsa-miR-4665-5p	hsa-miR-6727-5p	hsa-miR-7850-5p
hsa-miR-3138	hsa-miR-4665-5p	hsa-miR-6727-5p	hsa-miR-7850-5p
hsa-miR-3139	hsa-miR-4665-5p	hsa-miR-6727-5p	hsa-miR-7850-5p
hsa-miR-3140-3p	hsa-miR-4665-5p	hsa-miR-6727-5p	hsa-miR-7851-3p
hsa-miR-3140-3p	hsa-miR-4665-5p	hsa-miR-6727-5p	hsa-miR-7854-3p
hsa-miR-3140-3p	hsa-miR-4665-5p	hsa-miR-6727-5p	hsa-miR-7854-3p
hsa-miR-3141	hsa-miR-4665-5p	hsa-miR-6728-3p	hsa-miR-7856-5p
hsa-miR-3141	hsa-miR-4666b	hsa-miR-6728-3p	hsa-miR-7974
hsa-miR-3144-3p	hsa-miR-4667-3p	hsa-miR-6728-3p	hsa-miR-7974
hsa-miR-3146	hsa-miR-4667-5p	hsa-miR-6728-3p	hsa-miR-7974
hsa-miR-3147	hsa-miR-4667-5p	hsa-miR-6728-5p	hsa-miR-7974
hsa-miR-3147	hsa-miR-4669	hsa-miR-6728-5p	hsa-miR-7974
hsa-miR-3147	hsa-miR-4669	hsa-miR-6729-5p	hsa-miR-7975
hsa-miR-3147	hsa-miR-4669	hsa-miR-6729-5p	hsa-miR-7976
hsa-miR-3147	hsa-miR-4669	hsa-miR-6729-5p	hsa-miR-7976
hsa-miR-3147	hsa-miR-4669	hsa-miR-6730-5p	hsa-miR-7976
hsa-miR-3147	hsa-miR-4670-5p	hsa-miR-6731-3p	hsa-miR-7978
hsa-miR-3147	hsa-miR-4671-5p	hsa-miR-6731-5p	hsa-miR-7978
hsa-miR-3147	hsa-miR-4672	hsa-miR-6731-5p	hsa-miR-7978
hsa-miR-3149	hsa-miR-4674	hsa-miR-6731-5p	hsa-miR-7978
hsa-miR-3150a-3p	hsa-miR-4674	hsa-miR-6731-5p	hsa-miR-7-5p
hsa-miR-3150a-3p	hsa-miR-4674	hsa-miR-6732-5p	hsa-miR-8052
hsa-miR-3150a-3p	hsa-miR-4675	hsa-miR-6732-5p	hsa-miR-8052
hsa-miR-3150b-3p	hsa-miR-4675	hsa-miR-6733-3p	hsa-miR-8052

hsa-miR-3150b-3p	hsa-miR-4675	hsa-miR-6733-3p	hsa-miR-8056
hsa-miR-3151-3p	hsa-miR-4675	hsa-miR-6733-3p	hsa-miR-8056
hsa-miR-3151-3p	hsa-miR-4675	hsa-miR-6734-3p	hsa-miR-8056
hsa-miR-3151-5p	hsa-miR-4675	hsa-miR-6734-5p	hsa-miR-8057
hsa-miR-3151-5p	hsa-miR-4675	hsa-miR-6734-5p	hsa-miR-8057
hsa-miR-3151-5p	hsa-miR-4675	hsa-miR-6734-5p	hsa-miR-8057
hsa-miR-3151-5p	hsa-miR-4675	hsa-miR-6734-5p	hsa-miR-8058
hsa-miR-3151-5p	hsa-miR-4676-3p	hsa-miR-6735-3p	hsa-miR-8059
hsa-miR-3151-5p	hsa-miR-4676-5p	hsa-miR-6735-3p	hsa-miR-8060
hsa-miR-3152-3p	hsa-miR-4677-3p	hsa-miR-6735-3p	hsa-miR-8062
hsa-miR-3154	hsa-miR-4677-5p	hsa-miR-6735-3p	hsa-miR-8064
hsa-miR-3157-3p	hsa-miR-4677-5p	hsa-miR-6735-5p	hsa-miR-8064
hsa-miR-3158-3p	hsa-miR-4677-5p	hsa-miR-6736-5p	hsa-miR-8064
hsa-miR-3158-5p	hsa-miR-4677-5p	hsa-miR-6736-5p	hsa-miR-8065
hsa-miR-3160-5p	hsa-miR-4678	hsa-miR-6736-5p	hsa-miR-8065
hsa-miR-3165	hsa-miR-4682	hsa-miR-6736-5p	hsa-miR-8068
hsa-miR-3165	hsa-miR-4682	hsa-miR-6736-5p	hsa-miR-8069
hsa-miR-3165	hsa-miR-4683	hsa-miR-6736-5p	hsa-miR-8069
hsa-miR-3166	hsa-miR-4684-3p	hsa-miR-6736-5p	hsa-miR-8069
hsa-miR-3167	hsa-miR-4684-5p	hsa-miR-6736-5p	hsa-miR-8069
hsa-miR-3168	hsa-miR-4684-5p	hsa-miR-6737-3p	hsa-miR-8069
hsa-miR-3169	hsa-miR-4684-5p	hsa-miR-6737-5p	hsa-miR-8069
hsa-miR-3169	hsa-miR-4685-3p	hsa-miR-6737-5p	hsa-miR-8069
hsa-miR-3169	hsa-miR-4685-3p	hsa-miR-6737-5p	hsa-miR-8069
hsa-miR-3170	hsa-miR-4685-5p	hsa-miR-6737-5p	hsa-miR-8071
hsa-miR-3170	hsa-miR-4685-5p	hsa-miR-6737-5p	hsa-miR-8071
hsa-miR-3170	hsa-miR-4686	hsa-miR-6737-5p	hsa-miR-8071
hsa-miR-3171	hsa-miR-4686	hsa-miR-6737-5p	hsa-miR-8071
hsa-miR-3173-5p	hsa-miR-4687-3p	hsa-miR-6738-3p	hsa-miR-8071
hsa-miR-3173-5p	hsa-miR-4687-3p	hsa-miR-6738-5p	hsa-miR-8071
hsa-miR-3173-5p	hsa-miR-4687-3p	hsa-miR-6738-5p	hsa-miR-8072
hsa-miR-3173-5p	hsa-miR-4687-3p	hsa-miR-6738-5p	hsa-miR-8072
hsa-miR-3174	hsa-miR-4687-3p	hsa-miR-6738-5p	hsa-miR-8072
hsa-miR-3175	hsa-miR-4687-5p	hsa-miR-6740-3p	hsa-miR-8072
hsa-miR-3176	hsa-miR-4688	hsa-miR-6740-3p	hsa-miR-8072
hsa-miR-3176	hsa-miR-4689	hsa-miR-6740-5p	hsa-miR-8072
hsa-miR-3177-3p	hsa-miR-4689	hsa-miR-6740-5p	hsa-miR-8072
hsa-miR-3177-5p	hsa-miR-4689	hsa-miR-6740-5p	hsa-miR-8072
hsa-miR-3177-5p	hsa-miR-4689	hsa-miR-6741-5p	hsa-miR-8072
hsa-miR-3178	hsa-miR-4689	hsa-miR-6741-5p	hsa-miR-8072
hsa-miR-3178	hsa-miR-4690-5p	hsa-miR-6741-5p	hsa-miR-8072
hsa-miR-3178	hsa-miR-4690-5p	hsa-miR-6741-5p	hsa-miR-8073
hsa-miR-3179	hsa-miR-4691-5p	hsa-miR-6741-5p	hsa-miR-8073

hsa-miR-3180-3p	hsa-miR-4691-5p	hsa-miR-6741-5p	hsa-miR-8073
hsa-miR-3180-3p	hsa-miR-4691-5p	hsa-miR-6741-5p	hsa-miR-8074
hsa-miR-3180-3p	hsa-miR-4691-5p	hsa-miR-6741-5p	hsa-miR-8075
hsa-miR-3180-3p	hsa-miR-4691-5p	hsa-miR-6741-5p	hsa-miR-8077
hsa-miR-3180-3p	hsa-miR-4692	hsa-miR-6741-5p	hsa-miR-8077
hsa-miR-3180-3p	hsa-miR-4692	hsa-miR-6741-5p	hsa-miR-8077
hsa-miR-3180-3p	hsa-miR-4692	hsa-miR-6741-5p	hsa-miR-8078
hsa-miR-3180-3p	hsa-miR-4695-5p	hsa-miR-6741-5p	hsa-miR-8078
hsa-miR-3180-5p	hsa-miR-4697-3p	hsa-miR-6742-3p	hsa-miR-8078
hsa-miR-3180	hsa-miR-4697-3p	hsa-miR-6742-5p	hsa-miR-8078
hsa-miR-3180	hsa-miR-4697-5p	hsa-miR-6742-5p	hsa-miR-8080
hsa-miR-3180	hsa-miR-4697-5p	hsa-miR-6743-3p	hsa-miR-8081
hsa-miR-3180	hsa-miR-4697-5p	hsa-miR-6743-5p	hsa-miR-8083
hsa-miR-3180	hsa-miR-4697-5p	hsa-miR-6743-5p	hsa-miR-8083
hsa-miR-3180	hsa-miR-4697-5p	hsa-miR-6744-3p	hsa-miR-8085
hsa-miR-3180	hsa-miR-4697-5p	hsa-miR-6744-5p	hsa-miR-8086
hsa-miR-3180	hsa-miR-4700-5p	hsa-miR-6744-5p	hsa-miR-8086
hsa-miR-3181	hsa-miR-4700-5p	hsa-miR-6744-5p	hsa-miR-8086
hsa-miR-3182	hsa-miR-4700-5p	hsa-miR-6745	hsa-miR-8089
hsa-miR-3182	hsa-miR-4700-5p	hsa-miR-6745	hsa-miR-8089
hsa-miR-3183	hsa-miR-4701-3p	hsa-miR-6745	hsa-miR-8089
hsa-miR-3184-5p	hsa-miR-4701-3p	hsa-miR-6745	hsa-miR-8089
hsa-miR-3184-5p	hsa-miR-4701-3p	hsa-miR-6745	hsa-miR-873-3p
hsa-miR-3186-3p	hsa-miR-4701-3p	hsa-miR-6745	hsa-miR-873-3p
hsa-miR-3186-5p	hsa-miR-4701-3p	hsa-miR-6745	hsa-miR-873-3p
hsa-miR-3187-3p	hsa-miR-4701-5p	hsa-miR-6745	hsa-miR-873-5p
hsa-miR-3187-3p	hsa-miR-4703-3p	hsa-miR-6746-5p	hsa-miR-874-5p
hsa-miR-3187-3p	hsa-miR-4705	hsa-miR-6746-5p	hsa-miR-875-3p
hsa-miR-3187-3p	hsa-miR-4706	hsa-miR-6746-5p	hsa-miR-875-3p
hsa-miR-3187-5p	hsa-miR-4706	hsa-miR-6747-3p	hsa-miR-876-3p
hsa-miR-3187-5p	hsa-miR-4706	hsa-miR-6747-5p	hsa-miR-876-5p
hsa-miR-3187-5p	hsa-miR-4706	hsa-miR-6747-5p	hsa-miR-885-3p
hsa-miR-3187-5p	hsa-miR-4706	hsa-miR-6747-5p	hsa-miR-885-3p
hsa-miR-3187-5p	hsa-miR-4707-5p	hsa-miR-6747-5p	hsa-miR-885-3p
hsa-miR-3187-5p	hsa-miR-4707-5p	hsa-miR-6747-5p	hsa-miR-885-3p
hsa-miR-3187-5p	hsa-miR-4708-5p	hsa-miR-6747-5p	hsa-miR-885-3p
hsa-miR-3187-5p	hsa-miR-4710	hsa-miR-6748-3p	hsa-miR-885-5p
hsa-miR-3187-5p	hsa-miR-4710	hsa-miR-6748-5p	hsa-miR-887-5p
hsa-miR-3188	hsa-miR-4710	hsa-miR-6748-5p	hsa-miR-887-5p
hsa-miR-3188	hsa-miR-4711-3p	hsa-miR-6748-5p	hsa-miR-889-5p
hsa-miR-3188	hsa-miR-4711-3p	hsa-miR-6748-5p	hsa-miR-891a-3p
hsa-miR-3189-3p	hsa-miR-4711-5p	hsa-miR-6748-5p	hsa-miR-891a-3p
hsa-miR-3189-3p	hsa-miR-4712-5p	hsa-miR-6748-5p	hsa-miR-891a-3p

hsa-miR-3189-5p	hsa-miR-4713-3p	hsa-miR-6748-5p	hsa-miR-891a-3p
hsa-miR-3190-3p	hsa-miR-4713-3p	hsa-miR-6748-5p	hsa-miR-892c-3p
hsa-miR-3190-3p	hsa-miR-4713-5p	hsa-miR-6749-3p	hsa-miR-892c-3p
hsa-miR-3190-5p	hsa-miR-4715-5p	hsa-miR-6749-5p	hsa-miR-920 E
hsa-miR-3190-5p	hsa-miR-4716-3p	hsa-miR-6749-5p	hsa-miR-920 E
hsa-miR-3191-3p	hsa-miR-4716-3p	hsa-miR-6749-5p	hsa-miR-920 E
hsa-miR-3191-3p	hsa-miR-4717-3p	hsa-miR-6749-5p	hsa-miR-920 E
hsa-miR-3191-3p	hsa-miR-4717-3p	hsa-miR-6749-5p	hsa-miR-920 E
hsa-miR-3191-3p	hsa-miR-4717-3p	hsa-miR-6750-5p	hsa-miR-920 E
hsa-miR-3191-3p	hsa-miR-4717-3p	hsa-miR-6751-5p	hsa-miR-920 E
hsa-miR-3191-5p	hsa-miR-4718	hsa-miR-6751-5p	hsa-miR-920 E
hsa-miR-3191-5p	hsa-miR-4721	hsa-miR-6751-5p	hsa-miR-920 E
hsa-miR-3191-5p	hsa-miR-4721	hsa-miR-6751-5p	hsa-miR-921 E
hsa-miR-3192-3p	hsa-miR-4721	hsa-miR-6751-5p	hsa-miR-921 E
hsa-miR-3192-3p	hsa-miR-4722-5p	hsa-miR-6751-5p	hsa-miR-921 E
hsa-miR-3192-5p	hsa-miR-4722-5p	hsa-miR-6751-5p	hsa-miR-922 E
hsa-miR-3192-5p	hsa-miR-4722-5p	hsa-miR-6751-5p	hsa-miR-92a-1-5p
hsa-miR-3192-5p	hsa-miR-4722-5p	hsa-miR-6751-5p	hsa-miR-92a-1-5p
hsa-miR-3192-5p	hsa-miR-4722-5p	hsa-miR-6751-5p	hsa-miR-92a-1-5p
hsa-miR-3193	hsa-miR-4722-5p	hsa-miR-6751-5p	hsa-miR-92a-1-5p
hsa-miR-3193	hsa-miR-4722-5p	hsa-miR-6751-5p	hsa-miR-92a-1-5p
hsa-miR-3193	hsa-miR-4722-5p	hsa-miR-6752-3p	hsa-miR-92a-1-5p
hsa-miR-3193	hsa-miR-4723-3p	hsa-miR-6752-3p	hsa-miR-92a-1-5p
hsa-miR-3193	hsa-miR-4723-5p	hsa-miR-6752-5p	hsa-miR-92a-1-5p
hsa-miR-3194-3p	hsa-miR-4723-5p	hsa-miR-6752-5p	hsa-miR-92a-2-5p
hsa-miR-3194-3p	hsa-miR-4723-5p	hsa-miR-6752-5p	hsa-miR-92a-2-5p
hsa-miR-3194-3p	hsa-miR-4723-5p	hsa-miR-6752-5p	hsa-miR-92a-2-5p
hsa-miR-3194-3p	hsa-miR-4723-5p	hsa-miR-6752-5p	hsa-miR-92a-2-5p
hsa-miR-3194-5p	hsa-miR-4725-3p	hsa-miR-6752-5p	hsa-miR-92a-2-5p
hsa-miR-3195	hsa-miR-4725-3p	hsa-miR-6752-5p	hsa-miR-92a-2-5p
hsa-miR-3195	hsa-miR-4725-3p	hsa-miR-6752-5p	hsa-miR-92a-2-5p
hsa-miR-3196	hsa-miR-4726-3p	hsa-miR-6752-5p	hsa-miR-92a-2-5p
hsa-miR-3196	hsa-miR-4726-3p	hsa-miR-6752-5p	hsa-miR-92b-5p
hsa-miR-3196	hsa-miR-4726-5p	hsa-miR-6752-5p	hsa-miR-92b-5p
hsa-miR-3196	hsa-miR-4726-5p	hsa-miR-6752-5p	hsa-miR-92b-5p
hsa-miR-3196	hsa-miR-4728-3p	hsa-miR-6753-3p	hsa-miR-933 E
hsa-miR-3196	hsa-miR-4728-3p	hsa-miR-6753-3p	hsa-miR-937-5p
hsa-miR-3196	hsa-miR-4728-3p	hsa-miR-6753-3p	hsa-miR-937-5p
hsa-miR-3197	hsa-miR-4728-5p	hsa-miR-6753-3p	hsa-miR-937-5p
hsa-miR-3197	hsa-miR-4728-5p	hsa-miR-6753-3p	hsa-miR-937-5p
hsa-miR-3197	hsa-miR-4728-5p	hsa-miR-6753-3p	hsa-miR-938 E
hsa-miR-3197	hsa-miR-4728-5p	hsa-miR-6753-5p	hsa-miR-939-3p
hsa-miR-3197	hsa-miR-4728-5p	hsa-miR-6753-5p	hsa-miR-939-3p

hsa-miR-3197	hsa-miR-4728-5p	hsa-miR-6754-5p	hsa-miR-939-3p
hsa-miR-3198	hsa-miR-4730	hsa-miR-6754-5p	hsa-miR-939-3p
hsa-miR-3198	hsa-miR-4730	hsa-miR-6754-5p	hsa-miR-939-5p
hsa-miR-3198	hsa-miR-4731-5p	hsa-miR-6754-5p	hsa-miR-939-5p
hsa-miR-3198	hsa-miR-4731-5p	hsa-miR-6755-3p	hsa-miR-939-5p
hsa-miR-3198	hsa-miR-4731-5p	hsa-miR-6755-5p	hsa-miR-939-5p
hsa-miR-3199	hsa-miR-4731-5p	hsa-miR-6756-3p	hsa-miR-939-5p
hsa-miR-3199	hsa-miR-4732-3p	hsa-miR-6756-5p	hsa-miR-93-3p
hsa-miR-31-3p	hsa-miR-4732-5p	hsa-miR-6756-5p	hsa-miR-93-3p
hsa-miR-31-5p	hsa-miR-4732-5p	hsa-miR-6756-5p	hsa-miR-942-5p
hsa-miR-3200-3p	hsa-miR-4734	hsa-miR-6756-5p	hsa-miR-942-5p
hsa-miR-3200-3p	hsa-miR-4734	hsa-miR-6756-5p	hsa-miR-942-5p
hsa-miR-3202	hsa-miR-4734	hsa-miR-6756-5p	hsa-miR-9500
hsa-miR-320a	hsa-miR-4734	hsa-miR-6756-5p	hsa-miR-96-5p
hsa-miR-320b	hsa-miR-4736	hsa-miR-6757-5p	hsa-miR-98-5p
hsa-miR-323a-5p	hsa-miR-4736	hsa-miR-6757-5p	hsa-miR-98-5p
hsa-miR-323a-5p	hsa-miR-4736	hsa-miR-6758-3p	hsa-miR-99b-3p
hsa-miR-323a-5p	hsa-miR-4736	hsa-miR-6758-5p	hsa-miR-9-5p
hsa-miR-323a-5p	hsa-miR-4737	hsa-miR-6759-5p	hsa-miR-9-5p
hsa-miR-323a-5p	hsa-miR-4738-5p	hsa-miR-6759-5p	hsa-miR-9-5p
hsa-miR-323a-5p	hsa-miR-4739	hsa-miR-6759-5p	hsa-miR-708-5p
hsa-miR-323a-5p	hsa-miR-4740-3p	hsa-miR-6759-5p	hsa-miR-708-3p
hsa-miR-323a-5p	hsa-miR-4740-5p	hsa-miR-6759-5p	hsa-miR-455-5p
hsa-miR-323b-5p	hsa-miR-4741	hsa-miR-6759-5p	hsa-miR-409-3p
hsa-miR-323b-5p	hsa-miR-4741	hsa-miR-6759-5p	hsa-miR-297
hsa-miR-323b-5p	hsa-miR-4741	hsa-miR-6759-5p	hsa-miR-206
hsa-miR-323b-5p	hsa-miR-4741	hsa-miR-6759-5p	hsa-miR-145-3p
hsa-miR-323b-5p	hsa-miR-4742-3p	hsa-miR-675-3p	hsa-miR-143-5p
hsa-miR-323b-5p	hsa-miR-4742-3p	hsa-miR-675-3p	hsa-miR-138-5p
hsa-miR-323b-5p	hsa-miR-4742-3p	hsa-miR-675-5p	hsa-miR-143-3p
hsa-miR-323b-5p	hsa-miR-4743-3p	hsa-miR-675-5p	hsa-miR-199a-3p
hsa-miR-323b-5p	hsa-miR-4743-3p	hsa-miR-675-5p	hsa-miR-199b-3p
hsa-miR-324-3p	hsa-miR-4743-5p	hsa-miR-675-5p	hsa-miR-146a-5p
hsa-miR-324-3p	hsa-miR-4743-5p	hsa-miR-675-5p	hsa-miR-129-5p
hsa-miR-324-5p	hsa-miR-4743-5p	hsa-miR-675-5p	hsa-miR-155-5p
hsa-miR-325 E	hsa-miR-4743-5p	hsa-miR-675-5p	hsa-miR-129-2-3p
hsa-miR-325 E	hsa-miR-4743-5p	hsa-miR-675-5p	hsa-miR-10b-3p
hsa-miR-325 E	hsa-miR-4745-3p	hsa-miR-675-5p	hsa-miR-450a-1-3p
hsa-miR-328-5p	hsa-miR-4745-3p	hsa-miR-6760-3p	hsa-let-7g-3p
hsa-miR-328-5p	hsa-miR-4745-3p	hsa-miR-6760-3p	hsa-miR-29a-5p
hsa-miR-328-5p	hsa-miR-4745-5p	hsa-miR-6760-5p	
hsa-miR-328-5p	hsa-miR-4745-5p	hsa-miR-6761-3p	hsa-let-7c-3p
hsa-miR-328-5p	hsa-miR-4745-5p	hsa-miR-6761-3p	hsa-miR-33a-5p

hsa-miR-329-5p	hsa-miR-4746-3p	hsa-miR-6761-5p	hsa-miR-4510
hsa-miR-329-5p	hsa-miR-4746-3p	hsa-miR-6762-3p	hsa-miR-9-5p
hsa-miR-329-5p	hsa-miR-4746-3p	hsa-miR-6762-3p	hsa-miR-877-3p
hsa-miR-329-5p	hsa-miR-4746-3p	hsa-miR-6762-3p	hsa-miR-653-3p
hsa-miR-330-5p	hsa-miR-4746-5p	hsa-miR-6762-3p	
hsa-miR-330-5p	hsa-miR-4746-5p	hsa-miR-6762-3p	hsa-miR-31-3p
hsa-miR-330-5p	hsa-miR-4747-3p	hsa-miR-6762-3p	hsa-miR-15a-5p
hsa-miR-330-5p	hsa-miR-4747-5p	hsa-miR-6762-5p	hsa-miR-182-3p
hsa-miR-331-5p	hsa-miR-4747-5p	hsa-miR-6762-5p	hsa-miR-125a-3p
hsa-miR-335-3p	hsa-miR-4747-5p	hsa-miR-6762-5p	hsa-miR-195-5p
hsa-miR-338-3p	hsa-miR-4748	hsa-miR-6762-5p	hsa-miR-107
hsa-miR-339-3p	hsa-miR-4748	hsa-miR-6763-3p	hsa-miR-130a-3p
hsa-miR-339-3p	hsa-miR-4748	hsa-miR-6763-3p	hsa-miR-514a-3p
hsa-miR-339-3p	hsa-miR-4749-5p	hsa-miR-6763-3p	hsa-miR-424-5p
hsa-miR-339-5p	hsa-miR-4749-5p	hsa-miR-6763-5p	hsa-miR-16-5p
hsa-miR-339-5p	hsa-miR-4749-5p	hsa-miR-6764-3p	hsa-miR-146b-5p
hsa-miR-339-5p	hsa-miR-4749-5p	hsa-miR-6764-3p	hsa-miR-940
hsa-miR-339-5p	hsa-miR-4750-3p	hsa-miR-6764-3p	hsa-miR-9-3p
hsa-miR-33a-5p	hsa-miR-4750-5p	hsa-miR-6764-5p	hsa-miR-627-3p
hsa-miR-33a-5p	hsa-miR-4750-5p	hsa-miR-6764-5p	hsa-miR-605-5p
hsa-miR-33b-3p	hsa-miR-4750-5p	hsa-miR-6764-5p	
hsa-miR-33b-3p	hsa-miR-4750-5p	hsa-miR-6765-3p	hsa-miR-378e
hsa-miR-33b-5p	hsa-miR-4751	hsa-miR-6765-3p	hsa-miR-24-2-5p
hsa-miR-33b-5p	hsa-miR-4751	hsa-miR-6765-5p	hsa-miR-103a-3p
hsa-miR-33b-5p	hsa-miR-4753-3p	hsa-miR-6766-3p	hsa-miR-30d-3p
hsa-miR-340-3p	hsa-miR-4753-3p	hsa-miR-6766-5p	hsa-miR-550a-3p
hsa-miR-342-5p	hsa-miR-4753-3p	hsa-miR-6766-5p	hsa-miR-15b-5p
hsa-miR-342-5p	hsa-miR-4753-3p	hsa-miR-6766-5p	hsa-miR-548n
hsa-miR-345-5p	hsa-miR-4753-5p	hsa-miR-6767-3p	hsa-let-7a-2-3p
hsa-miR-346 E	hsa-miR-4754	hsa-miR-6767-5p	hsa-miR-615-5p
hsa-miR-34a-5p	hsa-miR-4755-3p	hsa-miR-6768-3p	hsa-miR-542-3p
hsa-miR-34a-5p	hsa-miR-4755-3p	hsa-miR-6768-5p	hsa-miR-196b-5p
hsa-miR-34a-5p	hsa-miR-4755-5p	hsa-miR-6768-5p	hsa-miR-301a-5p
hsa-miR-34a-5p	hsa-miR-4755-5p	hsa-miR-6769a-5p	hsa-miR-24-1-5p
hsa-miR-34a-5p	hsa-miR-4756-3p	hsa-miR-6769a-5p	hsa-miR-1260a
hsa-miR-34a-5p	hsa-miR-4756-3p	hsa-miR-6769a-5p	hsa-miR-5100
hsa-miR-34a-5p	hsa-miR-4756-5p	hsa-miR-6769a-5p	hsa-miR-503-5p
hsa-miR-34b-5p	hsa-miR-4757-3p	hsa-miR-6769a-5p	hsa-miR-1260b
hsa-miR-34c-5p	hsa-miR-4757-5p	hsa-miR-6769a-5p	hsa-miR-27a-5p
hsa-miR-34c-5p	hsa-miR-4757-5p	hsa-miR-6769a-5p	hsa-miR-378g
hsa-miR-34c-5p	hsa-miR-4757-5p	hsa-miR-6769a-5p	hsa-miR-6501-5p
hsa-miR-34c-5p	hsa-miR-4758-3p	hsa-miR-6769b-3p	hsa-miR-346
hsa-miR-34c-5p	hsa-miR-4758-5p	hsa-miR-6769b-5p	hsa-miR-125b-2-3p

hsa-miR-3529-5p	hsa-miR-4758-5p	hsa-miR-6769b-5p	hsa-miR-196a-5p
hsa-miR-3605-3p	hsa-miR-4758-5p	hsa-miR-6769b-5p	hsa-miR-6720-5p
hsa-miR-3605-5p	hsa-miR-4758-5p	hsa-miR-6769b-5p	hsa-miR-548ab
hsa-miR-3607-5p	hsa-miR-4761-3p	hsa-miR-6769b-5p	hsa-miR-141-3p
hsa-miR-3610	hsa-miR-4761-5p	hsa-miR-6769b-5p	hsa-miR-342-5p
hsa-miR-3612	hsa-miR-4763-3p	hsa-miR-6769b-5p	hsa-miR-1306-5p
hsa-miR-3614-3p	hsa-miR-4763-3p	hsa-miR-676-3p	hsa-miR-183-3p
hsa-miR-3614-5p	hsa-miR-4763-3p	hsa-miR-676-3p	hsa-miR-324-3p
hsa-miR-3614-5p	hsa-miR-4763-3p	hsa-miR-676-3p	hsa-miR-378d
hsa-miR-3614-5p	hsa-miR-4763-3p	hsa-miR-676-3p	
hsa-miR-3615	hsa-miR-4763-3p	hsa-miR-6770-3p	
hsa-miR-3615	hsa-miR-4763-3p	hsa-miR-6770-3p	
hsa-miR-3615	hsa-miR-4763-3p	hsa-miR-6770-3p	
hsa-miR-3615	hsa-miR-4763-5p	hsa-miR-6770-3p	
hsa-miR-3616-3p	hsa-miR-4764-5p	hsa-miR-6770-5p	
hsa-miR-3616-3p	hsa-miR-4764-5p	hsa-miR-6771-5p	
hsa-miR-3616-3p	hsa-miR-4764-5p	hsa-miR-6771-5p	
hsa-miR-3619-3p	hsa-miR-4764-5p	hsa-miR-6771-5p	
hsa-miR-3619-3p	hsa-miR-4765	hsa-miR-6771-5p	
hsa-miR-3619-5p	hsa-miR-4767	hsa-miR-6771-5p	
hsa-miR-3619-5p	hsa-miR-4767	hsa-miR-6771-5p	
hsa-miR-361-3p	hsa-miR-4767	hsa-miR-6772-3p	
hsa-miR-3620-3p	hsa-miR-4767	hsa-miR-6772-3p	

d. HMUCC cohort

hsa-miR-708-5p	1.3703442	5.184001418	4.316832869	0	#DIV/0!
hsa-miR-708-3p	5.93815819	10.13236641	2.158416435	0	#DIV/0!
hsa-miR-516b-5p	1.3703442	0.471272856	0	0	#DIV/0!
hsa-miR-455-5p	81.7638705	208.066966	35.88367323	0	#DIV/0!
hsa-miR-455-3p	6.39493959	13.19563997	2.967822598	0	#DIV/0!
hsa-miR-409-3p	1.3703442	0.706909284	1.88861438	0	#DIV/0!
hsa-miR-34c-5p	218.79829	312.6895401	87.4158656	0	#DIV/0!
hsa-miR-34c-3p	3.6542512	2.827637137	0.539604109	0	#DIV/0!
hsa-miR-34b-3p	9.5924093	22.3854606	4.04703081	0	#DIV/0!

	9	7	5		
hsa-miR-297	4.56781399	3.298909993	0.269802054	0	#DIV/0!
hsa-miR-214-5p	38.3696376	82.94402268	11.87129039	0	#DIV/0!
hsa-miR-214-3p	100.948689	164.7098632	28.59901776	0	#DIV/0!
hsa-miR-206	97.7512195	177.9055032	30.48763214	0	#DIV/0!
hsa-miR-145-5p	44.3077958	98.49602693	19.15594586	0	#DIV/0!
hsa-miR-145-3p	112.825006	218.9062417	32.37624652	0	#DIV/0!
hsa-miR-143-5p	38.826419	69.74838271	14.02970682	0	#DIV/0!
hsa-miR-138-5p	7.30850239	14.84509497	3.237624652	0	#DIV/0!
hsa-miR-143-3p	5959.62692	13032.34393	2245.8323	4.886409308	1219.633
hsa-miR-199a-3p	4323.89273	8202.739698	2279.287755	8.144015513	530.9288
hsa-miR-199b-3p	2159.66246	4097.246211	1138.564669	4.072007756	530.368
hsa-miR-199a-5p	1663.59786	3654.485363	601.6585811	4.886409308	340.4541
hsa-miR-146a-5p	687.912788	1088.404661	423.5892253	3.257606205	211.1713
hsa-miR-16-1-3p	74.9121495	112.6342126	16.72772737	2.443204654	30.66143
hsa-miR-199b-5p	10.5059722	25.2130978	6.745051358	0.407200776	25.80047
hsa-miR-129-5p	909.908548	1290.580717	286.7995837	47.23528997	19.26332
hsa-miR-155-5p	52.9866423	77.28874841	24.821789	2.850405429	18.58916
hsa-miR-129-2-3p	15.0737862	25.68437066	4.047030815	0.814401551	18.50903
hsa-miR-181a-3p	4.1110326	10.13236641	1.88861438	0.407200776	10.09584
hsa-miR-6877-5p	3.6542512	3.770182849	2.967822598	0.407200776	8.974077
hsa-miR-10b-3p	66.2333029	98.02475408	29.94802803	7.736814737	8.560797
hsa-miR-450a-1-3	6.8517209	15.5520042	3.50742670	0.81440155	8.41319

p	9	5	6	1	7
hsa-let-7g-3p	3.1974698	3.770182849	1.079208217	0.407200776	7.852318
hsa-miR-26a-2-3p	12.3330978	21.20727853	13.22030066	1.628803103	7.571878
hsa-miR-29a-5p	5.93815819	7.069092842	4.316832869	0.814401551	7.291438
hsa-miR-6842-3p	2.7406884	1.17818214	1.88861438	0.407200776	6.730558
hsa-miR-6087	7.76528379	4.241455705	5.665843141	1.221602327	6.356638
hsa-miR-10b-5p	3642.83166	5943.221989	2278.748151	748.8422264	4.864618
hsa-miR-345-3p	9.59240939	12.72436712	7.824259575	2.036003878	4.711391
hsa-let-7c-3p	13.2466606	12.01745783	9.98267601	2.850405429	4.64729
hsa-miR-6500-3p	1.8271256	0.706909284	1.079208217	0.407200776	4.487039
hsa-miR-3648	3.6542512	1.413818568	2.158416435	0.814401551	4.487039
hsa-let-7c-5p	3648.76982	6395.172658	2811.87701	842.4984048	4.330892
hsa-miR-33a-5p	10.9627536	14.60945854	8.363863684	2.850405429	3.846033
hsa-miR-4510	12.3330978	23.56364281	7.554457521	3.257606205	3.785939
hsa-miR-9-5p	41.110326	67.156382	33.18565268	10.99442094	3.739199
hsa-miR-222-5p	16.4441304	14.84509497	7.014853412	4.479208532	3.671213
hsa-miR-877-3p	12.7898792	11.7818214	5.396041087	3.664806981	3.489919
hsa-miR-653-3p	1.3703442	1.885091425	2.428218489	0.407200776	3.365279
hsa-miR-3607-3p	1.3703442	2.592000709	3.777228761	0.407200776	3.365279
hsa-miR-31-3p	13.703442	9.661093551	8.363863684	4.072007756	3.365279
hsa-miR-184	2.7406884	0.235636428	0.539604109	0.814401551	3.365279
hsa-miR-328-3p	152.108206	262.9702537	122.4901327	45.60648687	3.335232
hsa-miR-15a-5p	7.7652837	12.7243671	10.2524780	2.44320465	3.17831

	9	2	6	4	9
hsa-miR-182-3p	12.7898792	26.15564352	9.712873956	4.072007756	3.140927
hsa-miR-125a-3p	123.787759	152.456769	76.35398137	40.31287679	3.070675
hsa-miR-195-5p	5.93815819	17.20145925	12.4108945	2.036003878	2.916575
hsa-miR-107	147.540392	227.6247895	141.6460785	51.30729773	2.875622
hsa-miR-130a-3p	95.9240939	155.5200425	65.02229509	33.79766438	2.838187
hsa-miR-514a-3p	6.85172099	4.94836499	4.047030815	2.443204654	2.804399
hsa-miR-4449	2.283907	0.706909284	1.349010272	0.814401551	2.804399
hsa-miR-424-5p	38.826419	67.156382	39.39109993	13.84482637	2.804399
hsa-miR-99b-5p	11632.3951	16559.58562	7913.564055	4150.597506	2.802583
hsa-let-7e-3p	27.8636654	47.8341949	22.93317462	10.58722017	2.631821
hsa-miR-653-5p	3.1974698	2.356364281	3.777228761	1.221602327	2.617439
hsa-miR-212-3p	12.7898792	17.67273211	15.10891504	4.886409308	2.617439
hsa-miR-16-5p	598.840415	921.1027973	707.6907885	230.8828398	2.593698
hsa-miR-522-3p	11.419535	16.49454997	7.014853412	4.479208532	2.549454
hsa-miR-146b-5p	143.429359	218.1993324	138.948058	61.08011635	2.348217
hsa-miR-99b-3p	852.354091	1124.692671	430.8738808	365.2590957	2.33356
hsa-miR-6510-3p	0.9135628	0.942545712	0.269802054	0.407200776	2.243519
hsa-miR-4440	0.9135628	1.413818568	3.507426706	0.407200776	2.243519
hsa-miR-3180-3p	9.13562799	12.96000354	9.443071901	4.072007756	2.243519
hsa-miR-3180	9.13562799	12.96000354	9.443071901	4.072007756	2.243519
hsa-miR-2682-5p	0.9135628	0.471272856	0	0.407200776	2.243519
hsa-miR-940	2.7406884	1.88509142	1.61881232	1.22160232	2.24351

		5	6	7	9
hsa-miR-9-3p	2.7406884	5.655274274	4.856436978	1.221602327	2.243519
hsa-miR-627-3p	1.8271256	1.885091425	4.316832869	0.814401551	2.243519
hsa-miR-605-5p	1.8271256	1.413818568	1.618812326	0.814401551	2.243519
hsa-miR-5009-5p	1.8271256	1.413818568	2.698020543	0.814401551	2.243519
hsa-miR-378e	14.6170048	20.26473281	18.8861438	6.51521241	2.243519
hsa-miR-149-5p	348.524208	444.1746669	272.7698769	156.3650978	2.228913
hsa-miR-24-2-5p	668.271187	836.2736832	450.2996287	302.9573771	2.205826
hsa-miR-147b	22.3822886	27.09818923	14.29950888	10.18001939	2.198649
hsa-miR-103a-3p	1807.484	2842.246595	2032.418875	823.7671691	2.194169
hsa-miR-30d-3p	41.5671074	58.20219773	39.12129788	19.13843645	2.171918
hsa-miR-125b-1-3p	372.276841	486.589224	322.1436529	171.4315265	2.171577
hsa-miR-212-5p	52.9866423	57.02401559	48.56436978	24.43204654	2.168735
hsa-miR-550a-3p	23.2958514	25.44873423	25.09159105	10.99442094	2.118879
hsa-miR-15b-5p	101.862252	139.4967654	117.9034977	48.86409308	2.084603
hsa-miR-31-5p	303.759631	441.5826662	298.9406762	145.7778777	2.083716
hsa-miR-548n	5.02459539	5.184001418	5.665843141	2.443204654	2.056559
hsa-miR-125b-5p	6616.93535	10814.7695	8257.831477	3263.307016	2.027678
hsa-miR-99a-5p	11258.2912	18718.95785	13602.07057	5571.728213	2.02061
hsa-miR-3909	14.6170048	19.79345996	19.69554997	7.329613961	1.994239
hsa-miR-34a-5p	36.0857306	66.44947272	71.76734645	18.3240349	1.969311
hsa-let-7a-2-3p	9.59240939	7.540365698	8.633665738	4.886409308	1.963079
hsa-miR-615-5p	22.83907	51.8400141	29.6782259	11.8088224	1.93406

		8	8	9	8
hsa-miR-542-3p	103.232596	169.1869554	103.6039889	53.75050238	1.920588
hsa-miR-196b-5p	223.366104	396.8117449	299.2104782	116.4594218	1.917974
hsa-miR-652-3p	110.99788	167.5375004	106.3020094	58.22971092	1.906207
hsa-let-7e-5p	1233.30978	1949.184533	1324.728087	649.8924379	1.897714
hsa-miR-132-5p	153.021769	183.0895046	160.5322223	81.03295435	1.888389
hsa-miR-3614-5p	2.283907	0.471272856	1.618812326	1.221602327	1.869599
hsa-miR-301a-5p	15.987349	25.68437066	9.443071901	8.551216288	1.869599
hsa-miR-24-1-5p	34.258605	58.90910702	32.10644446	18.3240349	1.869599
hsa-miR-1260a	282.290905	342.6153664	503.1808313	151.0714878	1.868592
hsa-miR-5100	42.9374516	40.52946563	55.84902525	23.21044421	1.849919
hsa-miR-503-5p	105.516503	170.1295011	111.6980505	57.41530936	1.837776
hsa-miR-1306-3p	16.4441304	15.55200425	11.60148834	8.958417064	1.835607
hsa-miR-1260b	303.759631	380.5528313	549.0471806	168.9883219	1.797518
hsa-miR-101-3p	3616.79512	4840.914778	4152.253616	2025.009457	1.786063
hsa-miR-27a-5p	954.673125	951.4998966	1312.04739	534.6546184	1.785588
hsa-miR-378g	24.6661956	34.63855493	28.59901776	13.84482637	1.781618
hsa-miR-6501-5p	5.02459539	1.413818568	0.809406163	2.850405429	1.762765
hsa-miR-346	6.39493959	9.425457123	4.047030815	3.664806981	1.744959
hsa-miR-125b-2-3p	79.9367449	136.6691283	120.3317162	46.01368765	1.737238
hsa-miR-196a-5p	1487.73702	2615.093079	2476.782859	861.6368412	1.72664
hsa-miR-30b-3p	13.2466606	15.0807314	31.2970383	7.736814737	1.712159
hsa-miR-1246	15.987349	16.4945499	11.0618842	9.36561784	1.70702

		7	3		6
hsa-miR-203a-3p	118.306382	234.9295188	142.9950888	70.03853341	1.689161
hsa-miR-6720-5p	1.3703442	3.063273565	5.126239032	0.814401551	1.682639
hsa-miR-548ab	2.7406884	1.649454997	2.428218489	1.628803103	1.682639
hsa-miR-449c-5p	1.3703442	2.356364281	3.237624652	0.814401551	1.682639
hsa-miR-141-3p	2.7406884	1.649454997	1.349010272	1.628803103	1.682639
hsa-miR-135b-5p	1.3703442	3.063273565	1.079208217	0.814401551	1.682639
hsa-miR-140-3p	1093.07789	1745.594659	1334.710763	651.1140402	1.678781
hsa-miR-22-5p	94.0969683	134.7840369	130.8539963	56.60090781	1.662464
hsa-miR-6720-3p	15.5305676	22.62109709	17.80693559	9.36561784	1.658253
hsa-miR-132-3p	159.416708	213.4866038	190.4802504	96.9137846	1.644933
hsa-miR-342-5p	11.8763164	9.425457123	11.33168628	7.329613961	1.620319
hsa-miR-1306-5p	10.5059722	12.96000354	13.22030066	6.51521241	1.612529
hsa-miR-125a-5p	3225.33346	5544.760789	3704.112404	2003.835017	1.60958
hsa-miR-183-3p	6.39493959	15.55200425	7.554457521	4.072007756	1.570464
hsa-miR-324-3p	32.4314794	45.00655776	33.99505885	20.76723956	1.561665
hsa-miR-625-3p	22.83907	22.85673352	29.94802803	14.65922792	1.558
hsa-miR-615-3p	1553.05676	2386.290107	1551.361812	1008.22912	1.540381
hsa-miR-378d	849.156622	1195.3836	964.2725422	555.0146572	1.529972
hsa-miR-26b-5p	771.047002	1239.447612	955.6388764	511.851375	1.506388

e. Overlapped miR

hsa-miR-103a-3p	hsa-miR-324-3p	hsa-miR-143-3p	hsa-miR-15b-5p
hsa-miR-129-5p	hsa-miR-33a-5p	hsa-miR-146a-5p	hsa-miR-542-3p
hsa-miR-155-5p	hsa-miR-409-3p	hsa-miR-107	hsa-miR-503-5p
hsa-miR-15a-5p	hsa-miR-455-5p	hsa-miR-424-5p	hsa-miR-141-3p
hsa-miR-16-5p	hsa-miR-206	hsa-miR-146b-5p	hsa-miR-199a-5p
hsa-miR-195-5p	hsa-miR-138-5p	hsa-miR-9-3p	
