# Supplementary material for: LncRNA TINCR favors tumorigenesis via STAT3–TINCR–EGFR-feedback loop by recruiting DNMT1 and acting as a competing endogenous RNA in human breast cancer
Source: Cell Death Dis. 2021 Jan 14;12(1):83. doi: 10.1038/s41419-020-03188-0 (PMC7809450; doi:10.1038/s41419-020-03188-0)
Supplement: Supplementary file 13 — Supplementary Table S6 [file 41419_2020_3188_MOESM13_ESM.pdf]

Target	Position	Chr	GenomePosition	Distance2TSS	Type	UACC812			MDA-MB-231		
						812-NC	812-Si TINCR #1	812-Si TINCR #2	NC	Si TINCR #1	Si TINCR #2
MIR503_1	92	X	133680280	148	CG	0.981412639	0.975693362	0.98074662	0.983446449	0.981616879	0.980378981
MIR503_1	203	X	133680391	37	CG	0.981618887	0.978372491	0.977246036	0.983956335	0.981651376	0.981871895
